# Supplementary material for: Proteomic Landscape Has Revealed Small Rubber Particles Are Crucial Rubber Biosynthetic Machines for Ethylene-Stimulation in Natural Rubber Production
Source: Int J Mol Sci. 2019 Oct 14;20(20):5082. doi: 10.3390/ijms20205082 (PMC6829444; doi:10.3390/ijms20205082)

Figure S2: MS identification of differentially expressed proteins in small rubber particles from 2D DIGE gels

Spot No.: **1**

The accumulation patterns of target protein spot on the DIGE gels:

(from left to right, the gels for D24, E24, merged for D24 and E24, D48, E48, merged for D48 and E48; arrows indicate the detail position of the target protein spot)

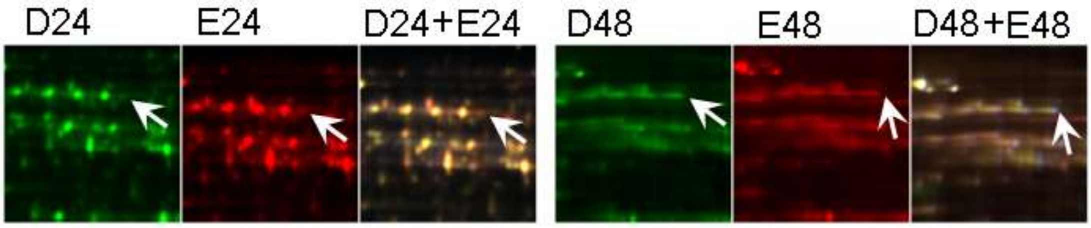

Accession No.: **scaffold1000_300919.mRNA1**

Plant species: ***Hevea brasiliensis***

Protein name：**Probable nucleoredoxin 1**

Peptide sequences: **R.HFTPNLVEVYEK.L；K.GDFEVVFISSDR.D；K.GDFEVVFISSDRDDESFNGYFSK.M；K.MPWLAIPFSDQEIR.K；R.DYGAEGYPFTPER.L；R.LDYFRQEEENAK.K；R.ALPTLVIIGQDGK.T；K.TLNPNVAELIEDHGIEAYPFTPEK.L；R.LEAQTLESVLVHGDKDFVIEK.S；K.LIEAYHEIK.A；R.EHLAAYGADAYPFTEDHLK.Q；K.LKHELHSQHELTR.T；K.HELHSQHELTR.T；R.QCDFDLHPK.C**

PFF Mascot score: **[737]**   Sequence coverage %: **[32]**

Matched peptides No.: **[14]**

Calculated Mr: **65399**   Calculated *p*I: **5.62**

Annotated PFF **spectra:**

Ions score is -10*Log(P), where P is the probability that the observed match is a random event.
Individual ions scores > 31 indicate identity or extensive homology (p<0.05).
Protein scores are derived from ions scores as a non-probabilistic basis for ranking protein hits.

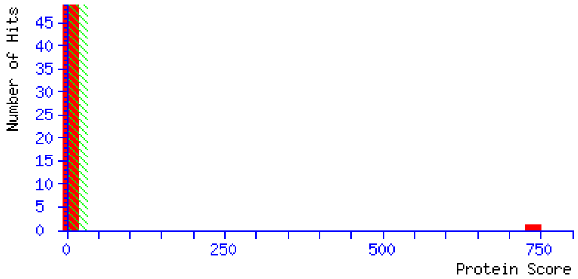

Matched peptide sequences: shown in **Bold Red**

```
  1 MANELVGDVS HDLFSLLSAE DRDFLIRNNG DQVKISSLVG KIVGLYFSAS
 51 WCGPCRHFTP NLVEVYEKLS SKGDFEVVFI SSDRDDESFN GYFSKMPWLA
101 IPFSDQEIRK RLKELFKVRG IPSLVILDTD GKVSCDQGVK IVRDYGAEGY
151 PFTPERLDYF RQEEENAKKN QTLSSILVSS SRDYLISKDG TKVAVSEIEG
201 KMVGLYFSVA SHRRCLEFTA KLVDVYKKLK EKGENFEVVL ISIDYDEKEF
251 KQSLETIPWL AIPFEDKCRE KLARYFELRA LPTLVIIGQD GKTLNPNVAE
301 LIEDHGIEAY PFTPEKLVEL AEIEKARLEA QTLESVLVHG DKDFVIEKSG
351 SKVAVSELVG KNVLLYFSAK WCPPCRAFLP KLIEAYHEIK AKDNAFEVIF
401 ISSDRDQSSF DEFYSEMPWL ALPFGDERKT ILQRKFKIKG IPAAIAISPK
451 GQTITKEARE HLAAYGADAY PFTEDHLKQL EEKLEETAKA WPEKLKHELH
501 SQHELTRTKR SGYVCNGCRE MGYLYSFYCR QCDFDLHPKC ALKKEEKGEA
551 EKGKEGWTCD GDVCRKA
```

Matched (bold red) stretches: 57–68 HFTPNLVEVYEK; 73–95 GDFEVVFISSDRDDESFNGYFSK; 96–109 MPWLAIPFSDQEIR; 144–168 DYGAEGYPFTPERLDYFRQEEENAK; 280–316 ALPTLVIIGQDGKTLNPNVAELIEDHGIEAYPFTPEK; 328–348 LEAQTLESVLVHGDKDFVIEK; 382–390 LIEAYHEIK; 460–478 EHLAAYGADAYPFTEDHLK; 495–507 LKHELHSQHELTR; 531–539 QCDFDLHPK.

Matched peptide information:

Start - End	Observed	Mr(expt)	Mr(calc)	ppm	Miss	Sequence
57 - 68	1475.8568	1474.8495	1474.7456	70	0	R.HFTPNLVEVYEK.L (Ions score 61)
73 - 84	1370.7523	1369.7450	1369.6514	68	0	K.GDFEVVFISSDR.D (Ions score 30)
73 - 95	2660.3564	2659.3491	2659.1715	67	1	K.GDFEVVFISSDRDDESFNGYFSK.M (Ions score 75)
96 - 109	1718.9772	1717.9699	1717.8498	70	0	K.MPWLAIPFSDQEIR.K Oxidation (M) (Ions score 39)
144 - 156	1501.7694	1500.7621	1500.6521	73	0	R.DYGAEGYPFTPER.L (Ions score 85)
157 - 168	1541.8330	1540.8257	1540.7157	71	1	R.LDYFRQEEENAK.K (Ions score 18)
280 - 292	1324.8561	1323.8488	1323.7762	55	0	R.ALPTLVIIGQDGK.T (Ions score 14)
293 - 316	2697.5322	2696.5249	2696.3333	71	0	K.TLNPNVAELIEDHGIEAYPFTPEK.L (Ions score 10)
328 - 348	2370.4248	2369.4175	2369.2478	72	1	R.LEAQTLESVLVHGDKDFVIEK.S (Ions score 67)
382 - 390	1115.6797	1114.6724	1114.6022	63	0	K.LIEAYHEIK.A (Ions score 12)
460 - 478	2148.1594	2147.1521	2146.9960	73	0	R.EHLAAYGADAYPFTEDHLK.Q (Ions score 134)
495 - 507	1627.9839	1626.9766	1626.8590	72	1	K.LKHELHSQHELTR.T (Ions score 87)
497 - 507	1386.7867	1385.7794	1385.6800	72	0	K.HELHSQHELTR.T (Ions score 56)
531 - 539	1159.5912	1158.5839	1158.5128	61	0	R.QCDFDLHPK.C (Ions score 45)

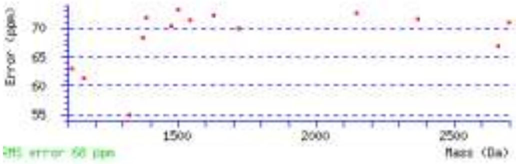

Spot No.: 2

The accumulation patterns of target protein spot on the DIGE gels:

(from left to right, the gels for D24, E24, merged for D24 and E24, D48, E48, merged for D48 and E48; arrows indicate the detail position of the target protein spot)

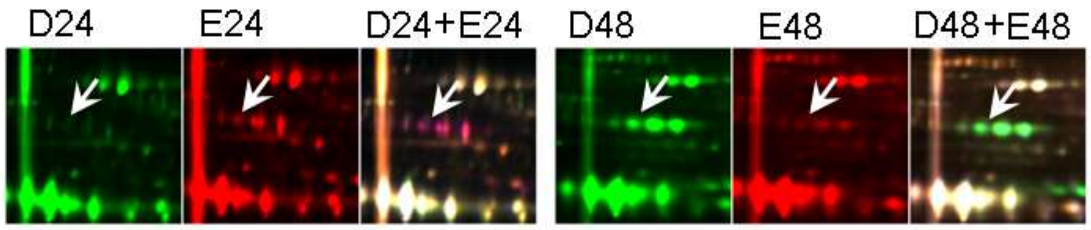

Accession No.: **scaffold0560_404767.mRNA1**

Plant species: ***Hevea brasiliensis***

Protein name： **Metacaspase-4**

Peptide sequences: **R.SAEPGDLLFVHYSGHGTR.L；R.EFVDQVPHGCR.I；K.QTIQDAFESR.G；R.VVEEDYGDSGYVK.S；K.LRPTLFDMFGDDASPK.V**

PFF Mascot score: **[137]** Sequence coverage %: **[16]**

Matched peptides No.: **[5]**

Calculated Mr: **46443** Calculated *p*I: **5.01**

Annotated PFF **spectra:**

Ions score is -10*Log(P), where P is the probability that the observed match is a random event.
Individual ions scores > 31 indicate identity or extensive homology (p<0.05).
Protein scores are derived from ions scores as a non-probabilistic basis for ranking protein hits.

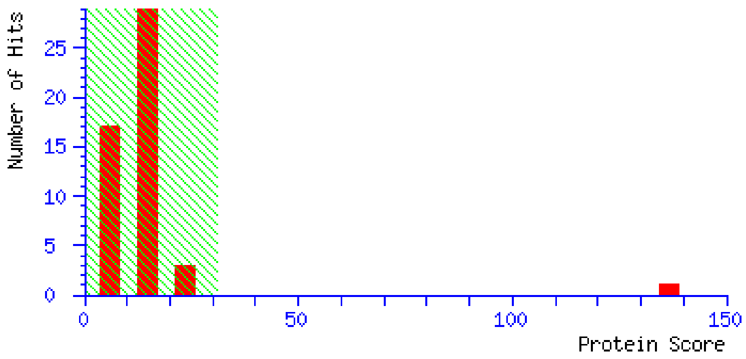

Matched peptide sequences: shown in **Bold Red**

1 MAKKAVLIGI NYPGTKAELK GCINDVKRMY RCLVDRYGFS EEDITVLIDI
51 DESYIQPTGK NIRRVLTDLV R**SAEPGDLLF VHYSGHGTR**L PAETGEDDDT
101 GFDECIVPCD MNLITDDDFR **EFVDQVPHGC R**ITVVSDSCH SGGLIDEAKE
151 QIGESTKRKE EESESGFGFK SFLK**QTIQDA FESR**GVHLPS DLHHHHGHRD
201 EEDFDNR**VVE EDYGDSGYVK** SKSLPLSTLI EILKQKTGKD DIDVGK**LRPT**
251 **LFDMFGDDAS PK**VKKFMKVI LNKLRHGDGE SGGGGFLGMV GSLAQEFLKH
301 KLDENDESYV KPALETEVDS KQEVYAGKTK RSLPDGGILI SGCQTDQTSA
351 DASPSGKSSE AYGALSNAIQ TIIAETDGAV TNQELVLKAR KMLKKQGFTQ
401 KPGLYCSDDH VEASFVC

Matched peptide information:

Start - End	Observed	Mr(expt)	Mr(calc)	ppm	Miss	Sequence
72 - 89	1943.0193	1942.0120	1941.9333	41	0	R.SAEPGDLLFVHYSGHGTR.L (Ions score 24)
121 - 131	1343.6781	1342.6708	1342.6088	46	0	R.EFVDQVPHGCR.I (Ions score 45)
175 - 184	1194.6357	1193.6284	1193.5677	51	0	K.QTIQDAFESR.G (Ions score 33)
208 - 220	1459.7251	1458.7178	1458.6515	45	0	R.VVEEDYGDSGYVK.S (Ions score 10)
247 - 262	1825.9493	1824.9420	1824.8716	39	1	K.LRPTLFDMFGDDASPK.V Oxidation (M) (Ions score 25)

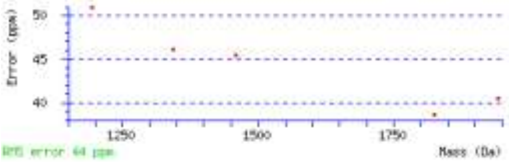

Spot No.: 3

The accumulation patterns of target protein spot on the DIGE gels:

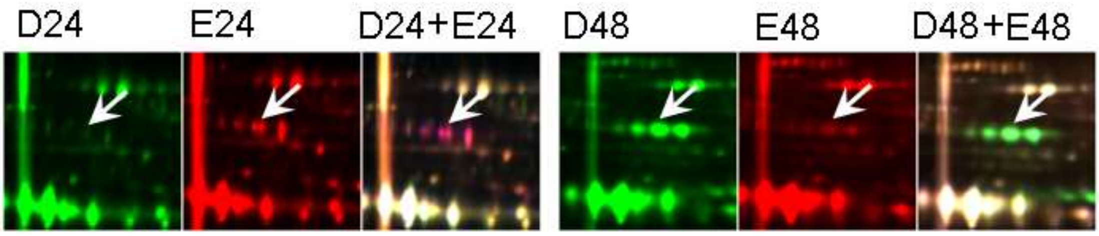

Accession No.: **scaffold0560_404767.mRNA1**

Plant species: ***Hevea brasiliensis***

Protein name：**Metacaspase-4**

Peptide sequences: **R.VLTDLVR.S；R.SAEPGDLLFVHYSGHGTR.L；R.EFVDQVPHGCR.I；K.QTIQDAFESR.G；K.LRPTLFDMFGDDASPK.V；K.LRPTLFDMFGDDASPK.V**

PFF Mascot score: **[338]** Sequence coverage %: **[14]**

Matched peptides No.: **[6]**

Calculated Mr: **46443** Calculated *p*I: **5.01**

Annotated PFF **spectra:**

Ions score is -10*Log(P), where P is the probability that the observed match is a random event.
Individual ions scores > 32 indicate identity or extensive homology (p<0.05).
Protein scores are derived from ions scores as a non-probabilistic basis for ranking protein hits.

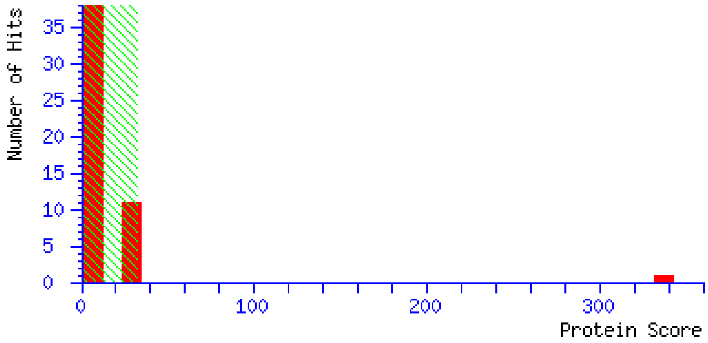

Matched peptide sequences: shown in **Bold Red**

1	MAKKAVLIGI	NYPGTKAELK	GCINDVKRMY	RCLVDRYGFS	EEDITVLIDI
51	DESYIQPTGK	NIRR**VLTDLV**	**RSAEPGDLLF**	**VHYSGHGTR**L	PAETGEDDDT
101	GFDECIVPCD	MNLITDDDFR	**EFVDQVPHGC**	**R**ITVVSDSCH	SGGLIDEAKE
151	QIGESTKRKE	EESESGFGFK	SFLK**QTIQDA**	**FESR**GVHLPS	DLHHHHGHRD
201	EEDFDNRVVE	EDYGDSGYVK	SKSLPLSTLI	EILKQKTGKD	DIDVGK**LRPT**
251	**LFDMFGDDAS**	**PK**VKKFMKVI	LNKLRHGDGE	SGGGGFLGMV	GSLAQEFLKH
301	KLDENDESYV	KPALETEVDS	KQEVYAGKTK	RSLPDGGILI	SGCQTDQTSA
351	DASPSGKSSE	AYGALSNAIQ	TIIAETDGAV	TNQELVLKAR	KMLKKQGFTQ
401	KPGLYCSDDH	VEASFVC			

Matched peptide information:

Start - End	Observed	Mr(expt)	Mr(calc)	ppm	Miss	Sequence
65 - 71	815.4955	814.4883	814.4913	-4	0	R.VLTDLVR.S (Ions score 27)
72 - 89	1942.9210	1941.9137	1941.9333	-10	0	R.SAEPGDLLFVHYSGHGTR.L (Ions score 104)
121 - 131	1343.6027	1342.5954	1342.6088	-10	0	R.EFVDQVPHGCR.I (Ions score 77)
175 - 184	1194.5636	1193.5563	1193.5677	-9	0	K.QTIQDAFESR.G (Ions score 57)
247 - 262	1809.8607	1808.8534	1808.8767	-13	1	K.LRPTLFDMFGDDASPK.V (Ions score 72)
247 - 262	1825.8291	1824.8218	1824.8716	-27	1	K.LRPTLFDMFGDDASPK.V Oxidation (M) (Ions score 18)

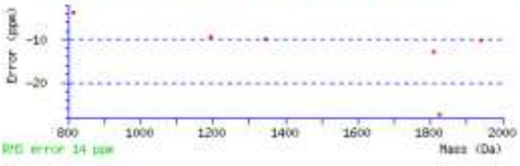

Spot No.: 4

The accumulation patterns of target protein spot on the DIGE gels:

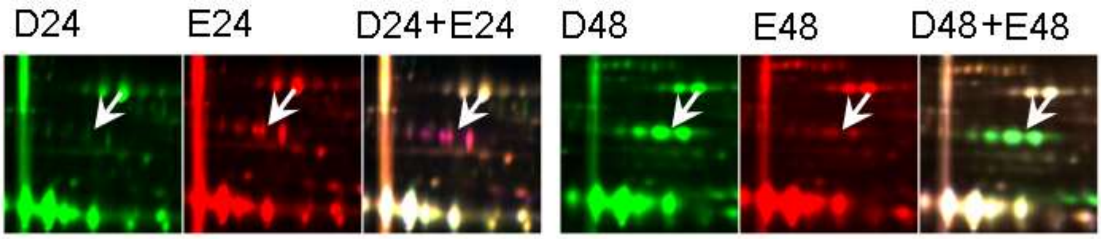

Accession No.: **scaffold0560_404767.mRNA1**

Plant species: ***Hevea brasiliensis***

Protein name： **Metacaspase-4**

Peptide sequences: **R.SAEPGDLLFVHYSGHGTR.L；K.QTIQDAFESR.G ； R.LPAETGEDDDTGFDECIVPCDMNLITDDDFR.E；R.EFVDQVPHGCR.I； R.GVHLPSDLHHHHGHR.D；R.GVHLPSDLHHHHGHRDEEDFDNR.V； K.LRPTLFDMFGDDASPK.V；R.HGDGESGGGGFLGMVGSLAQEFLK.H； K.SSEAYGALSNAIQTIIAETDGAVTNQELVLK.A**

PFF Mascot score: **[855]**

Sequence coverage %: **[39]**

Matched peptides No.: **[9]**

Calculated Mr: **46443**

Calculated *p*I: **5.01**

Annotated PFF **spectra:**

Ions score is -10*Log(P), where P is the probability that the observed match is a random event.
Individual ions scores > 30 indicate identity or extensive homology (p<0.05).
Protein scores are derived from ions scores as a non-probabilistic basis for ranking protein hits.

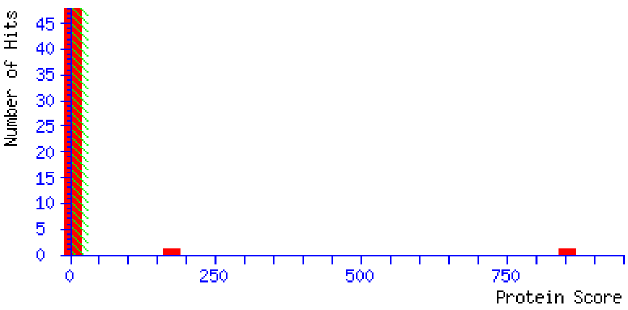

Matched peptide sequences: shown in Bold Red

```
  1 MAKKAVLIGI NYPGTKAELK GCINDVKRMY RCLVDRYGFS EEDITVLIDI
 51 DESYIQPTGK NIRRVLTDLV RSAEPGDLLF VHYSGHGTRL PAETGEDDDT
101 GFDECIVPCD MNLITDDDFR EFVDQVPHGC RITVVSDSCH SGGLIDEAKE
151 QIGESTKRKE EESESGFGFK SFLKQTIQDA FESRGVHLPS DLHHHHGHRD
201 EEDFDNRVVE EDYGDSGYVK SKSLPLSTLI EILKQKTGKD DIDVGKLRPT
251 LFDMFGDDAS PKVKKFMKVI LNKLRHGDGE SGGGGFLGMV GSLAQEFLKH
301 KLDENDESYV KPALETEVDS KQEVYAGKTK RSLPDGGILI SGCQTDQTSA
351 DASPSGKSSE AYGALSNAIQ TIIAETDGAV TNQELVLKAR KMLKKQGFTQ
401 KPGLYCSDDH VEASFVC
```

Matched peptide information:

Start - End	Observed	Mr(expt)	Mr(calc)	ppm	Miss	Sequence
72 - 89	1942.8949	1941.8876	1941.9333	-24	0	R.SAEPGDLLFVHYSGHGTR.L (Ions score 139)
90 - 120	3575.3813	3574.3740	3574.4690	-27	0	R.LPAETGEDDDTGFDECIVPCDMNLITDDDFR.E (Ions score 146)
121 - 131	1343.5896	1342.5823	1342.6088	-20	0	R.EFVDQVPHGCR.I (Ions score 71)
175 - 184	1194.5493	1193.5420	1193.5677	-21	0	K.QTIQDAFESR.G (Ions score 65)
185 - 199	1735.8248	1734.8175	1734.8564	-22	0	R.GVHLPSDLHHHHGHR.D (Ions score 87)
185 - 207	2756.1868	2755.1795	2755.2348	-20	1	R.GVHLPSDLHHHHGHRDEEDFDNR.V (Ions score 53)
247 - 262	1809.8430	1808.8357	1808.8767	-23	1	K.LRPTLFDMFGDDASPK.V (Ions score 95)
276 - 299	2350.0518	2349.0445	2349.1060	-26	0	R.HGDGESGGGGFLGMVGSLAQEFLK.H (Ions score 118)
358 - 388	3206.5674	3205.5601	3205.6354	-23	0	K.SSEAYGALSNAIQTIIAETDGAVTNQELVLK.A (Ions score 81)

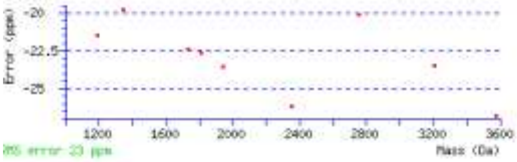

Spot No.: **5**

The accumulation patterns of target protein spot on the DIGE gels:

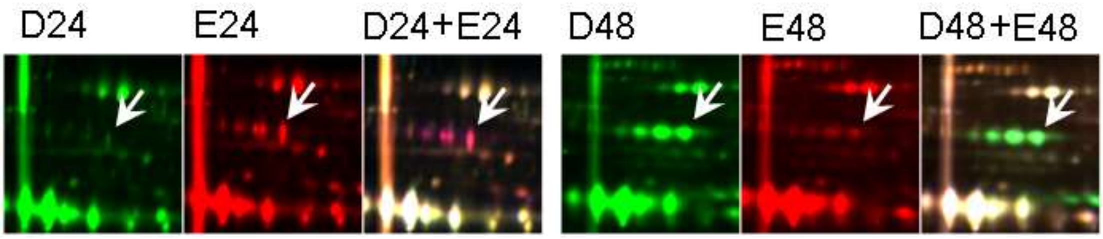

Accession No.: **scaffold0560_404767.mRNA1**

Plant species: ***Hevea brasiliensis***

Protein name： **Metacaspase-4**

Peptide sequences: R.SAEPGDLLFVHYSGHGTR.L; R.EFVDQVPHGCR.I; R.LPAETGEDDDTGFDECIVPCDMNLITDDDFR.E; K.QTIQDAFESR.G; R.GVHLPSDLHHHHGHR.D; R.DEEDFDNRVVEEDYGDSGYVK.S; R.VVEEDYGDSGYVK.S; K.LRPTLFDMFGDDASPK.V; R.HGDGESGGGGFLGMVGSLAQEFLK.H

PFF Mascot score: **[786]** Sequence coverage %: **[35]**

Matched peptides No.: **[9]**

Calculated Mr: **46443** Calculated *p*I: **5.01**

Annotated PFF **spectra:**

Ions score is -10*Log(P), where P is the probability that the observed match is a random event.
Individual ions scores > 30 indicate identity or extensive homology (p<0.05).
Protein scores are derived from ions scores as a non-probabilistic basis for ranking protein hits.

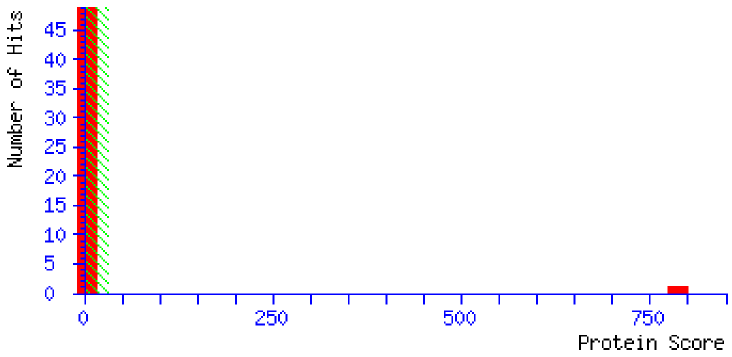

Matched peptide sequences: shown in **Bold Red**

```
  1 MAKKAVLIGI NYPGTKAELK GCINDVKRMY RCLVDRYGFS EEDITVLIDI
 51 DESYIQPTGK NIRRVLTDLV RSAEPGDLLF VHYSGHGTRL PAETGEDDDT
101 GFDECIVPCD MNLITDDDFR EFVDQVPHGC RITVVSDSCH SGGLIDEAKE
151 QIGESTKRKE EESESGFGFK SFLKQTIQDA FESRGVHLPS DLHHHHGHRD
201 EEDFDNRVVE EDYGDSGYVK SKSLPLSTLI EILKQKTGKD DIDVGKLRPT
251 LFDMFGDDAS PKVKKFMKVI LNKLRHGDGE SGGGGFLGMV GSLAQEFLKH
301 KLDENDESYV KPALETEVDS KQEVYAGKTK RSLPDGGILI SGCQTDQTSA
351 DASPSGKSSE AYGALSNAIQ TIIAETDGAV TNQELVLKAR KMLKKQGFTQ
401 KPGLYCSDDH VEASFVC
```

Matched peptide information:

Start - End	Observed	Mr(expt)	Mr(calc)	ppm	Miss	Sequence
72 - 89	1942.9321	1941.9248	1941.9333	-4	0	R.SAEPGDLLFVHYSGHGTR.L (Ions score 121)
90 - 120	3575.4709	3574.4636	3574.4698	-2	0	R.LPAETGEDDDTGFDECIVPCDMNLITDDDFR.E (Ions score 105)
121 - 131	1343.6149	1342.6076	1342.6088	-1	0	R.EFVDQVPHGCR.I (Ions score 66)
175 - 184	1194.5731	1193.5658	1193.5677	-2	0	K.QTIQDAFESR.G (Ions score 92)
185 - 199	1735.8535	1734.8462	1734.8564	-6	0	R.GVHLPSDLHHHHGHR.D (Ions score 77)
200 - 220	2480.0283	2479.0210	2479.0299	-4	1	R.DEEDFDNRVVEEDYGDSGYVK.S (Ions score 18)
208 - 220	1459.6484	1458.6411	1458.6515	-7	0	R.VVEEDYGDSGYVK.S (Ions score 83)
247 - 262	1809.8781	1808.8708	1808.8767	-3	1	K.LRPTLFDMFGDDASPK.V (Ions score 90)
276 - 299	2350.1011	2349.0938	2349.1060	-5	0	R.HGDGESGGGGFLGMVGSLAQEFLK.H (Ions score 132)

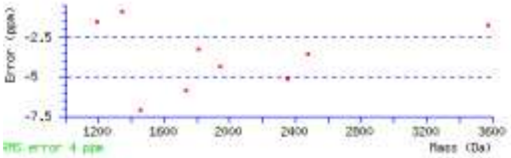

Spot No.: **6**

The accumulation patterns of target protein spot on the DIGE gels:

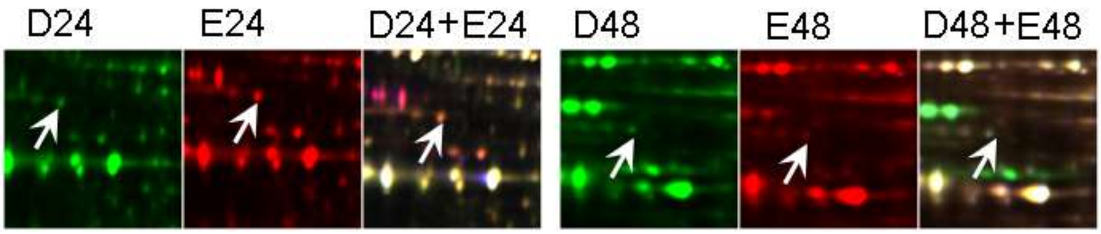

Accession No.: **scaffold0829_389953.mRNA1**

Plant species: ***Hevea brasiliensis***

Protein name：**Tubulin alpha-3 chain**

Peptide sequences: **R.AVFVDLEPTVIDEVR.T；R.QLFHPEQLISGK.E；R.FDGAINVDVTEFQTNLVPYPR.I；K.CGINYQPPTVVPGGDLAR.V；R.AFVHWYVGEGMEEGEFSEAR.E**

PFF Mascot score: **[451]**

Sequence coverage %: **[19]**

Matched peptides No.: **[5]**

Calculated Mr: **50244**

Calculated *p*I: **5.00**

Annotated PFF **spectra:**

Ions score is -10*Log(P), where P is the probability that the observed match is a random event.
Individual ions scores > 31 indicate identity or extensive homology (p<0.05).
Protein scores are derived from ions scores as a non-probabilistic basis for ranking protein hits.

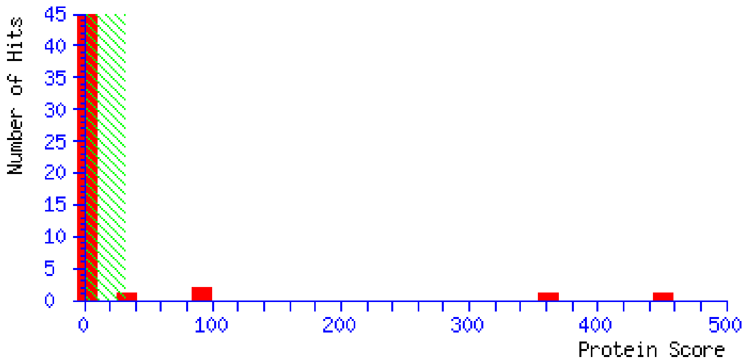

Matched peptide sequences: shown in Bold Red

1 MREIISIHIG QAGIQVGNSC WELYCLEHGI HPDGMMPSDT SVGVAHDAFN
51 TFFSETGSGK HVPR**AVFVDL EPTVIDEVR**T GPYR**QLFHPE QLISGK**EDAA
101 NNFARGHYTV GKEIVDLCLD RVRKLADNCT GLQGFLVFNA VGGGTGSGLG
151 SLLLERLSVD YGKKSKLGFT IYPSPQVSTA VVEPYNSVLS THSLLEHTDV
201 AVLLDNEAIY DICRRSLDIE RPTYTNLNRL ISQIISSLTT SLR**FDGAINV**
251 **DVTEFQTNLV PYPR**IHFMLS SYAPVISAEK AYHEQLSVPE ITNAVFEPSS
301 MMAKCDPRHG KYMACCLMYR GDVVPKDVNA AVATIKTKRT VQFVDWCPTG
351 FK**CGINYQPP TVVPGGDLAR** VQRAVCMISN NTAVAEVFSR IDHKFDLMYA
401 KR**AFVHWYVG EGMEEGEFSE AR**EDLAALEK DYEEVGAEGG DDEEEGEDY

Matched peptide information:

Start - End	Observed	Mr(expt)	Mr(calc)	ppm	Miss	Sequence
65 - 79	1701.8055	1700.7982	1700.8985	-59	0	R.AVFVDLEPTVIDEVR.T (Ions score 89)
85 - 96	1396.6731	1395.6658	1395.7510	-61	0	R.QLFHPEQLISGK.E (Ions score 64)
244 - 264	2395.0469	2394.0396	2394.1856	-61	0	R.FDGAINVDVTEFQTNLVPYPR.I (Ions score 100)
353 - 370	1913.8384	1912.8311	1912.9465	-60	0	K.CGINYQPPTVVPGGDLAR.V (Ions score 90)
403 - 422	2329.8765	2328.8692	2329.0110	-61	0	R.AFVHWYVGEGMEEGEFSEAR.E (Ions score 109)

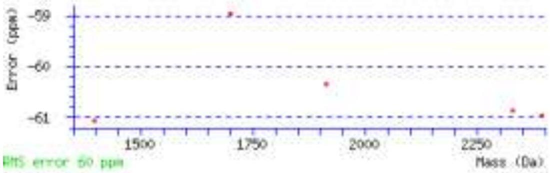

Spot No.: **7**

The accumulation patterns of target protein spot on the DIGE gels:

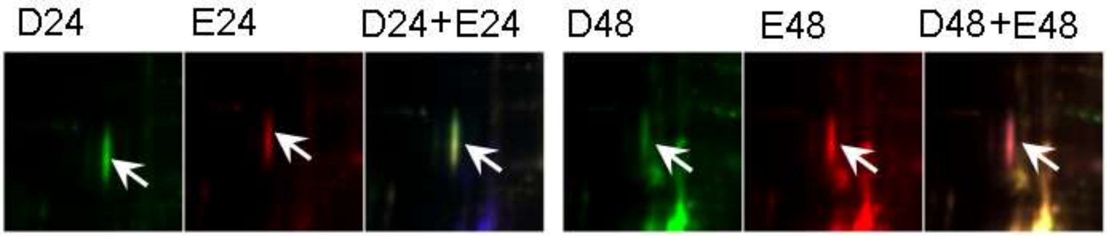

Accession No.: **scaffold5971_1039.mRNA1**

Plant species: ***Hevea brasiliensis***

Protein name：**Inactive GDSL esterase/lipase-like protein 25**

Peptide sequences: **K.YLPSYHHPYGTTFFDYPTGR.F；R.TVVDFVAENVSLPR.I；K.AVYLISFGADDYLNYEIPSEASR.E；K.ELYDFGAR.K；R.EQLESIVDVVLGNISDR.I；R.IKELYDFGAR.K；K.EIIDAPGEHGFK.Y**

PFF Mascot score: **[419]**

Sequence coverage %: **[26]**

Matched peptides No.: **[7]**

Calculated Mr: **41480**

Calculated *p*I: **4.60**

Annotated PFF **spectra:**

Ions score is -10*Log(P), where P is the probability that the observed match is a random event.
Individual ions scores > 31 indicate identity or extensive homology (p<0.05).
Protein scores are derived from ions scores as a non-probabilistic basis for ranking protein hits.

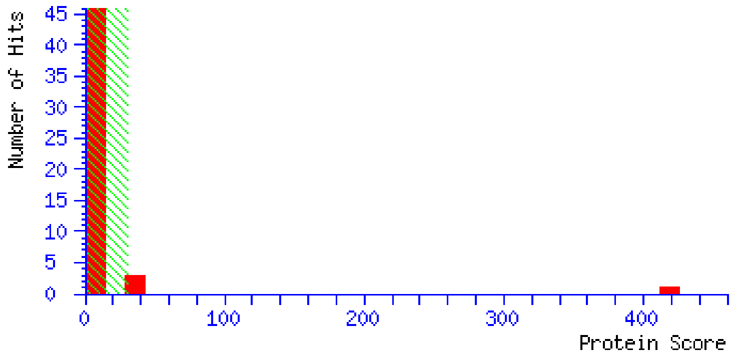

Matched peptide sequences: shown in Bold Red

```
  1 MASLAYSLFI LSLFTFTLLN PVCSELDEYL FSFGDGLYDA GNAKFIYPDK
 51 YLPSYHHPYG TTFFDYPTGR FSDGRTVVDF VAENVSLPRI PPFKNKEANF
101 TYGANFASEG ATASDSNPLI DFRSQIRDFG ELKLEWAVQL VNVTELARRL
151 KKAVYLISFG ADDYLNYEIP SEASREQLES IVDVVLGNIS DRIKELYDFG
201 ARKFVVENVA PLGLIPFIKQ TSDNSTLFYE LASLHAMKLP QILEKIQDGY
251 LFPEFNYTVF NYFGIIKEII DAPGEHGFKY GDIACCGNST YRGQACGFLD
301 YEFCVCGNKT EYLFFDGTHN TDAANNLLAE LMWDKESGFI SPYGVKDFFP
351 SPTTIQTLLT EATALG
```

Bold red (matched) residues: 51–70 YLPSYHHPYG TTFFDYPTGR; 76–89 TVVDF VAENVSLPR; 153–202 AVYLISFG ADDYLNYEIP SEASREQLES IVDVVLGNIS DRIKELYDFG AR; 268–279 EII DAPGEHGFK.

Matched peptide information:

Start - End	Observed	Mr(expt)	Mr(calc)	ppm	Miss	Sequence
51 - 70	2419.2244	2418.2171	2418.1069	46	0	K.YLPSYHHPYGTTFFDYPTGR.F (Ions score 50)
76 - 89	1545.9088	1544.9015	1544.8199	53	0	R.TVVDFVAENVSLPR.I (Ions score 48)
153 - 175	2593.3601	2592.3528	2592.2384	44	0	K.AVYLISFGADDYLNYEIPSEASR.E (Ions score 70)
176 - 192	1886.0894	1885.0821	1884.9793	55	0	R.EQLESIVDVVLGNISDR.I (Ions score 100)
193 - 202	1211.7180	1210.7107	1210.6346	63	1	R.IKELYDFGAR.K (Ions score 62)
195 - 202	970.5330	969.5257	969.4556	72	0	K.ELYDFGAR.K (Ions score 5)
268 - 279	1312.7219	1311.7146	1311.6459	52	0	K.EIIDAPGEHGFK.Y (Ions score 85)

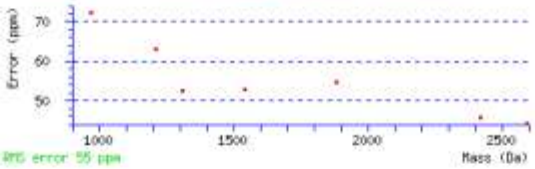

Spot No.: 8

The accumulation patterns of target protein spot on the DIGE gels:

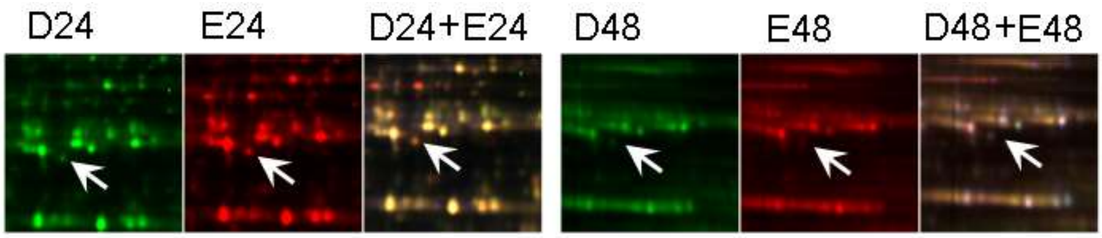

Accession No.: **scaffold2091_1808.mRNA1**

Plant species: ***Hevea brasiliensis***

Protein name：**Elongation factor 1-gamma**

Peptide sequences: **K.VPVLETPDGPLFESNAIAR.Y；R.MGFAPYLPPAEEAAISALKR.G；K.SFTSEFPHVER.Y；R.YFWTMVNQPNFK.K；K.MILDEWKR.L；K.YNDENTVSFVTLNK.V；R.GQEIPQFVIDECYDMELYEWK.K；R.VNQMIEDQEPFEGEPLLDAK.C**

PFF Mascot score: **[523]** Sequence coverage %: **[29]**

Matched peptides No.: **[8]**

Calculated Mr: **48685** Calculated *p*I: **5.69**

Annotated PFF **spectra:**

Ions score is -10*Log(P), where P is the probability that the observed match is a random event.
Individual ions scores > 31 indicate identity or extensive homology (p<0.05).
Protein scores are derived from ions scores as a non-probabilistic basis for ranking protein hits.

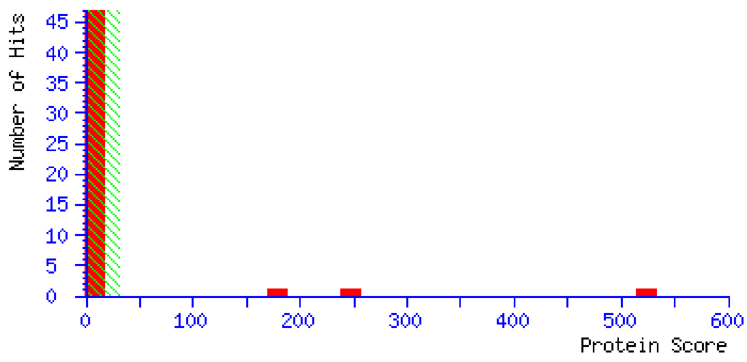

Matched peptide sequences: shown in Bold Red

1 MALVLHAGKT NKNAFKSLIA AEYSGVKVEL VENFEMGVSN KTPEFLKMNP
51 IGK**VPVLETP DGPLFESNAI AR**YVTRLKAD NPLYDSSLID YAHIEQWIDF
101 ASLEIDANLM AWLRPR**MGFA PYLPPAEEAA ISALKR**GLGA LNTHLASNTY
151 LVGHSVTLAD IITTCNLYMG FALLMTK**SFT SEFPHVERYF WTMVNQPNFK**
201 KILGEVKQTE SVLPVQSTKK PAQPKESAKS KRKDEPKKEA KTEKEPAKPK
251 EAAEEEEEAP KPKPKNPLDL LPPSK**MILDE WKR**LYSNTKT NFREVAIKGF
301 WDMYDPEGYS LWFCDYK**YND ENTVSFVTLN K**VSGFLQRMD LARKYAFGKM
351 LVIGENPPYK VKGLWLFR**GQ EIPQFVIDEC YDMELYEWK**K VDISDEAQKE
401 R**VNQMIEDQE PFEGEPLLDA K**CFK

Matched peptide information:

Start - End	Observed	Mr(expt)	Mr(calc)	ppm	Miss	Sequence
54 - 72	2025.0549	2024.0476	2024.0578	-5	0	K.VPVLETPDGPLFESNAIAR.Y (Ions score 109)
117 - 136	2132.1096	2131.1023	2131.1135	-5	1	R.MGFAPYLPPAEEAAISALKR.G (Ions score 141)
178 - 188	1335.6278	1334.6205	1334.6255	-4	0	K.SFTSEFPHVER.Y (Ions score 59)
189 - 200	1574.7664	1573.7591	1573.7388	13	0	R.YFWTMVNQPNFK.K (Ions score 50)
276 - 283	1090.5739	1089.5666	1089.5641	2	1	K.MILDEWKR.L (Ions score 42)
318 - 331	1643.8140	1642.8067	1642.7839	14	0	K.YNDENTVSFVTLNK.V (Ions score 32)
369 - 389	2692.1909	2691.1836	2691.1873	-1	0	R.GQEIPQFVIDECYDMELYEWK.K (Ions score 37)
402 - 421	2302.1262	2301.1189	2301.0835	15	0	R.VNQMIEDQEPFEGEPLLDAK.C (Ions score 54)

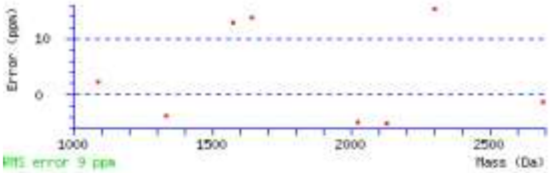

Spot No.: 9

The accumulation patterns of target protein spot on the DIGE gels:

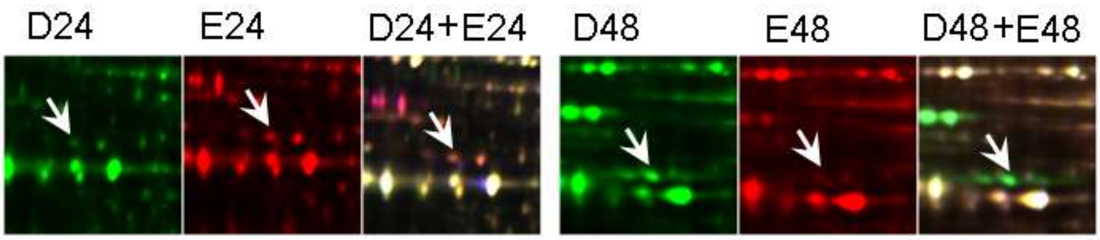

Accession No.: **scaffold1072_242122.mRNA1**

Plant species: ***Hevea brasiliensis***

Protein name： **Phospholipase A1-IIdelta**

Peptide sequences: **K.VMLEDAANYQVCSFIYGTAR.V；R.EIYIAFR.G；K.LESAEPLLR.G；K.IDVIPHYPGR.L；R.YTGTELEIDTR.K**

PFF Mascot score: **[242]** Sequence coverage %: **[13]**

Matched peptides No.: **[5]**

Calculated Mr: **47415** Calculated *p*I: **5.47**

Annotated PFF **spectra:**

Ions score is -10*Log(P), where P is the probability that the observed match is a random event.
Individual ions scores > 31 indicate identity or extensive homology (p<0.05).
Protein scores are derived from ions scores as a non-probabilistic basis for ranking protein hits.

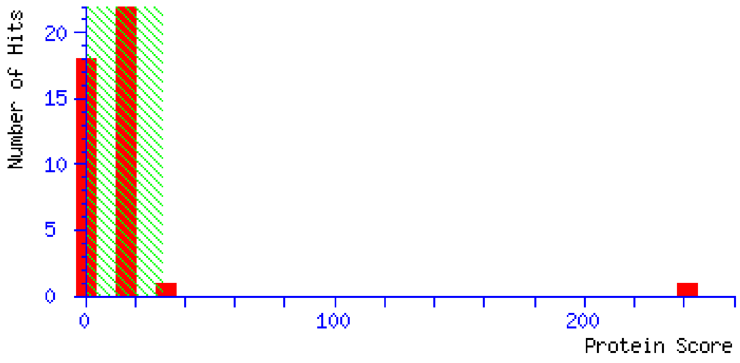

Matched peptide sequences: shown in **Bold Red**

1 METPSTQEPT WPEILGSKNW ENLLDPLDLT VRKLILRCGD FCQATYDSFN
51 NDENSKYCGT SRYGKKDFFQ K**VMLEDAANY** **QVCSFIYGTA** **R**VSLPEAILL
101 HSMTRDSWDR ESNWIGYIAV TSDEYTKVIG RR**EIYIAFR**G TTRNYEWVNI
151 LGAK**LESAEP** **LLR**GSSQNVE EANSGSDSDN EDNRKVPKVM LGWLTIYISD
201 NQESPFTKLS ARQQLVTKIN QLKEQYRDEN LSIILTGHSL GATLSILAAF
251 DLVENGITDI PVAAFVFGSP QVGNKAFNSR LNKYPNLKVL HIKNK**IDVIP**
301 **HYPGR**LLGYR **YTGTELEIDT** **R**KSPNLKDSK HTGDWHNLQA MLHIVAGWNG
351 EKGEFMLRVK RSLALVNKSC EFLKDECMVP GSWWIEKNKG MVRGEDGEWT
401 MASPDEEDQP VPEF

Matched peptide information:

Start - End	Observed	Mr(expt)	Mr(calc)	ppm	Miss	Sequence
72 - 91	2307.9888	2306.9815	2307.0664	-37	0	K.VMLEDAANYQVCSFIYGTAR.V (Ions score 89)
133 - 139	911.4761	910.4689	910.4912	-25	0	R.EIYIAFR.G (Ions score 60)
155 - 163	1027.5502	1026.5429	1026.5709	-27	0	K.LESAEPLLR.G (Ions score 19)
296 - 305	1166.6019	1165.5946	1165.6244	-26	0	K.IDVIPHYPGR.L (Ions score 44)
311 - 321	1297.6023	1296.5950	1296.6198	-19	0	R.YTGTELEIDTR.K (Ions score 30)

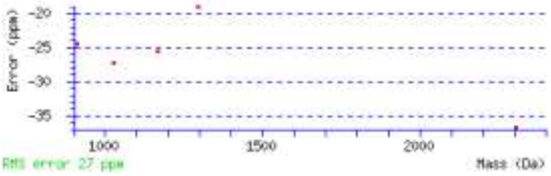

Spot No.: **10**

The accumulation patterns of target protein spot on the DIGE gels:

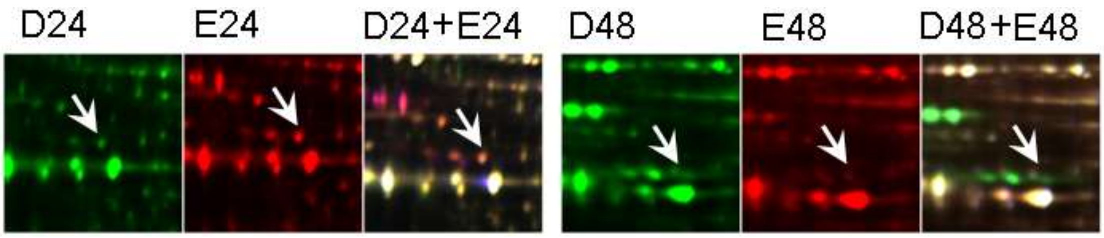

Accession No.: **scaffold1072_242122.mRNA1**

Plant species: ***Hevea brasiliensis***

Protein name：**Phospholipase A1-IIdelta**

Peptide sequences: **K.NWENLLDPLDLTVR.K；R.EIYIAFR.G；K.VMLEDAANYQVCSFIYGTAR.V；R.VSLPEAILLHSMTR.D；K.LESAEPLLR.G；K.NKIDVIPHYPGR.L；K.IDVIPHYPGR.L；R.YTGTELEIDTR.K**

PFF Mascot score: **[703]**

Sequence coverage %: **[21]**

Matched peptides No.: **[8]**

Calculated Mr: **47415**

Calculated *p*I: **5.47**

Annotated PFF **spectra:**

Ions score is -10*Log(P), where P is the probability that the observed match is a random event.
Individual ions scores > 31 indicate identity or extensive homology (p<0.05).
Protein scores are derived from ions scores as a non-probabilistic basis for ranking protein hits.

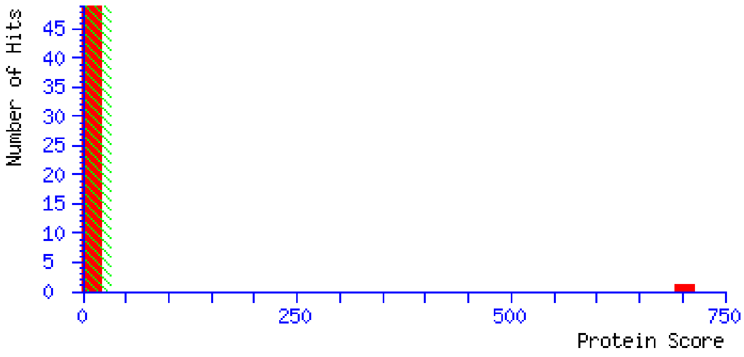

Matched peptide sequences: shown in Bold Red

1 METPSTQEPT WPEILGSK**NW ENLLDPLDLT VR**KLILRCGD FCQATYDSFN
51 NDENSKYCGT SRYGKKDFFQ K**VMLEDAANY QVCSFIYGTA RVSLPEAILL**
101 **HSMTR**DSWDR ESNWIGYIAV TSDEYTKVIG RR**EIYIAFR**G TTRNYEWVNI
151 LGAK**LESAEP LLR**GSSQNVE EANSGSDSDN EDNRKVPKVM LGWLTIYISD
201 NQESPFTKLS ARQQLVTKIN QLKEQYRDEN LSIILTGHSL GATLSILAAF
251 DLVENGITDI PVAAFVFGSP QVGNKAFNSR LNKYPNLKVL HIK**NKIDVIP**
301 **HYPGR**LLGYR **YTGTELEIDT R**KSPNLKDSK HTGDWHNLQA MLHIVAGWNG
351 EKGEFMLRVK RSLALVNKSC EFLKDECMVP GSWWIEKNKG MVRGEDGEWT
401 MASPDEEDQP VPEF

Matched peptide information:

Start - End	Observed	Mr(expt)	Mr(calc)	ppm	Miss	Sequence
19 - 32	1697.8680	1696.8607	1696.8784	-10	0	K.NWENLLDPLDLTVR.K (Ions score 123)
72 - 91	2308.0400	2307.0327	2307.0664	-15	0	K.VMLEDAANYQVCSFIYGTAR.V (Ions score 140)
92 - 105	1566.8573	1565.8500	1565.8599	-6	0	R.VSLPEAILLHSMTR.D (Ions score 119)
133 - 139	911.4960	910.4887	910.4912	-3	0	R.EIYIAFR.G (Ions score 47)
155 - 163	1027.5784	1026.5711	1026.5709	0	0	K.LESAEPLLR.G (Ions score 70)
294 - 305	1408.7640	1407.7567	1407.7623	-4	1	K.NKIDVIPHYPGR.L (Ions score 67)
296 - 305	1166.6240	1165.6167	1165.6244	-7	0	K.IDVIPHYPGR.L (Ions score 61)
311 - 321	1297.6312	1296.6239	1296.6198	3	0	R.YTGTELEIDTR.K (Ions score 77)

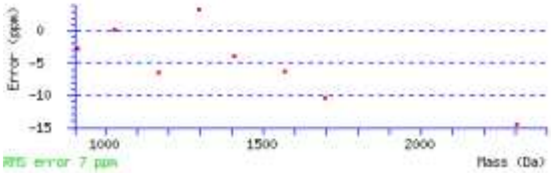

Spot No.: **11**

The accumulation patterns of target protein spot on the DIGE gels:

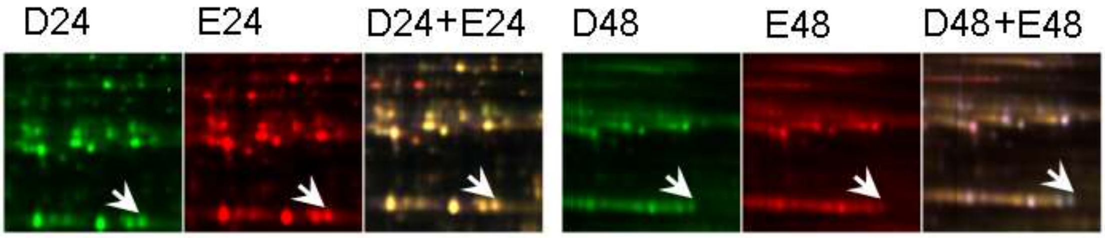

Accession No.: **scaffold0947_141222.mRNA1**

Plant species: ***Hevea brasiliensis***

Protein name： **Serpin-ZX**

Peptide sequences: **K.GPTLHQLLSFLNSK.S ；**

K.SNDHLSSFFSELVSVVFVDGSASGGPR.L；R.LSFANGVWVDR.S；K.EVLPFGSVDNTTR.L；R.LIFANALYFK.G；K.QFICAFDGFK.V；K.RQFSMYFFLPDAK.N；R.QFSMYFFLPDAK.N；K.VGSDSGFLDHHLPQQK.V；K.LDFVADHPFLFLIR.E；R.EDISGMVLFIGQVLDPSQAK.-

PFF Mascot score: **[812]** Sequence coverage %: **[37]**

Matched peptides No.: **[11]**

Calculated Mr: **42822** Calculated *p*I: **5.94**

Annotated PFF **spectra:**

Ions score is -10*Log(P), where P is the probability that the observed match is a random event.
Individual ions scores > 31 indicate identity or extensive homology (p<0.05).
Protein scores are derived from ions scores as a non-probabilistic basis for ranking protein hits.

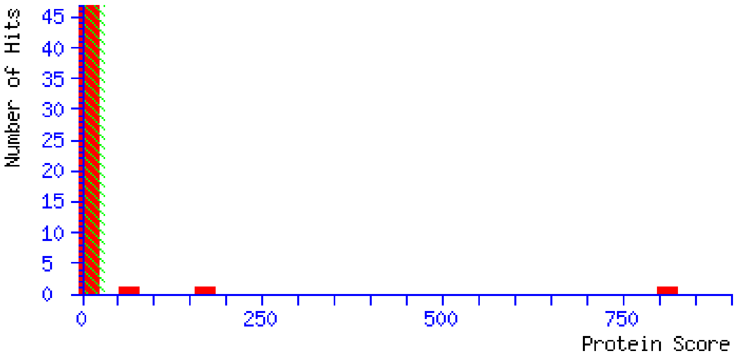

Matched peptide sequences: shown in **Bold Red**

```
  1 MDLRESIVNQ NAVTLGLSKH VLLTEAKTSN SVLSPLSIQV VLGLIAAGSK
 51 GPTLHQLLSF LNSKSNDHLS SFFSELVSVV FVDGSASGGP RLSFANGVWV
101 DRSLLLKPSF KQVVENVYKA ASNQVDFQNK AVEVTNEVNA WAEKETSGLI
151 KEVLPFGSVD NTTRLIFANA LYFKGAWNEK FNASTTKDYD FYLLNGSSVH
201 VSFMTSKKKQ FICAFDGFKV LGLPYKQGEA KRQFSMYFFL PDAKNGLPAL
251 VEKVGSDSGF LDHHLPQQKV EVGDFRIPRF KVSFGFEASK TLKGLGLVLP
301 FSDKGDLTEM VDSSVSQNLY VSSIFHKSFI EVNEEGTEAA AASAGVVNLE
351 SLTLADKLDF VADHPFLFLI REDISGMVLF IGQVLDPSQA K
```

Matched peptide information:

Start - End	Observed	Mr(expt)	Mr(calc)	ppm	Miss	Sequence
51 - 64	1554.8429	1553.8356	1553.8566	-13	0	K.GPTLHQLLSFLNSK.S (Ions score 79)
65 - 91	2797.3127	2796.3054	2796.3355	-11	0	K.SNDHLSSFFSELVSVVFVDGSASGGPR.L (Ions score 177)
92 - 102	1263.6348	1262.6275	1262.6408	-10	0	R.LSFANGVWVDR.S (Ions score 60)
152 - 164	1434.7125	1433.7052	1433.7151	-7	0	K.EVLPFGSVDNTTR.L (Ions score 96)
165 - 174	1199.6628	1198.6555	1198.6750	-16	0	R.LIFANALYFK.G (Ions score 72)
210 - 219	1232.5619	1231.5546	1231.5696	-12	0	K.QFICAFDGFK.V (Ions score 58)
232 - 244	1649.8020	1648.7947	1648.8072	-8	1	K.RQFSMYFFLPDAK.N (Ions score 16)
233 - 244	1493.6919	1492.6846	1492.7061	-14	0	R.QFSMYFFLPDAK.N (Ions score 47)
254 - 269	1764.8425	1763.8352	1763.8591	-14	0	K.VGSDSGFLDHHLPQQK.V (Ions score 62)
358 - 371	1702.9149	1701.9076	1701.9243	-10	0	K.LDFVADHPFLFLIR.E (Ions score 72)
372 - 391	2147.0908	2146.0835	2146.0980	-7	0	R.EDISGMVLFIGQVLDPSQAK.- (Ions score 73)

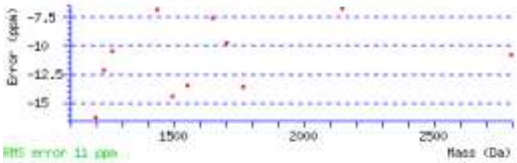

Spot No.: **12**

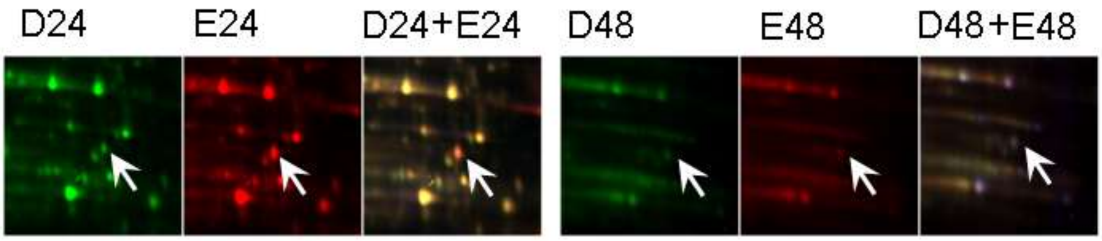

Accession No.: **scaffold1479_76107.mRNA1**

Plant species: ***Hevea brasiliensis***

Protein name： **Acetyl-CoA acetyltransferase, cytosolic 1**

Peptide sequences: **M.APVAAAEIKPR.D；R.EDQDNYAIHSFER.G；K.ITGYADAAQAPELFTTAPALAIPK.A；K.VNVHGGAVSLGHPLGCSGAR.I；R.ILVTLLGVLR.Q**

PFF Mascot score: **[109]** Sequence coverage %: **[19]**

Matched peptides No.: **[5]**

Calculated Mr: **41646** Calculated *p*I: **6.01**

Annotated PFF **spectra:**

Ions score is -10*Log(P), where P is the probability that the observed match is a random event.
Individual ions scores > 30 indicate identity or extensive homology (p<0.05).
Protein scores are derived from ions scores as a non-probabilistic basis for ranking protein hits.

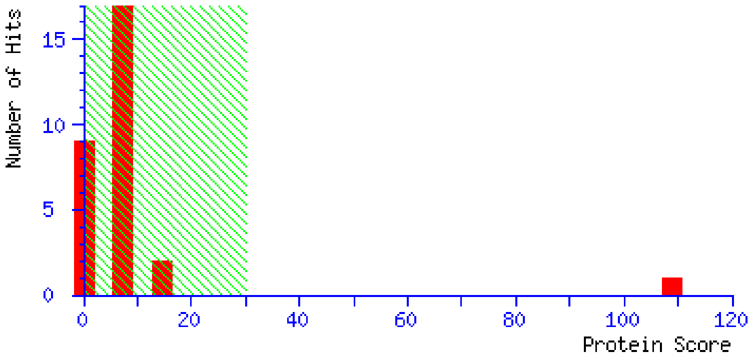

Matched peptide sequences: shown in **Bold Red**

1 M**APVAAAEIK PR**DVCIVGVA RTPMGGFLGL LSTLPATKLG SIAIEAALKR
51 ANVDPSLVQE VFFGNVLSAN LGQAPARQAA LGAGIPNSVV CTTVNKVCAS
101 GMKATMLAAQ SIQLGINDVV VAGGMESMSN APKYLAEARK GSRLGHDSVV
151 DGMLKDGLWD VFNDVGMGSC AEICADNHSI TR**EDQDNYAI HSFER**GIAAQ
201 DSGAFAWEIV PVEVSGGRGK PSTIVDKDEG LGKFDPVKLR KLRPSFKENG
251 GTVTAGNASS ISDGAAALVL VSGETALKLG LQVIAK**ITGY ADAAQAPELF**
301 **TTAPALAIPK** AVSNAGLDAS QVDYYEINEA FAVVALANQK LLGLNPEK**VN**
351 **VHGGAVSLGH PLGCSGARIL VTLLGVLR**QK NGKYGVGGVC NGGGGASALV
401 VELL

Matched peptide information:

Start - End	Observed	Mr(expt)	Mr(calc)	ppm	Miss	Sequence
2 - 12	1122.6364	1121.6291	1121.6557	-24	1	M.APVAAAEIKPR.D (Ions score 58)
183 - 195	1623.6702	1622.6629	1622.6961	-20	0	R.EDQDNYAIHSFER.G (Ions score 14)
287 - 310	2430.2498	2429.2425	2429.2842	-17	0	K.ITGYADAAQAPELFTTAPALAIPK.A (Ions score 3)
349 - 368	1944.9457	1943.9384	1943.9748	-19	0	K.VNVHGGAVSLGHPLGCSGAR.I (Ions score 5)
369 - 378	1096.7192	1095.7119	1095.7380	-24	0	R.ILVTLLGVLR.Q (Ions score 28)

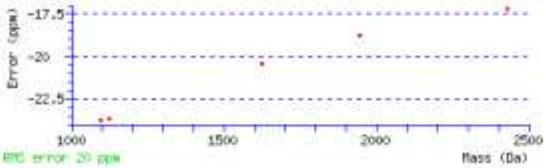

Spot No.: **13**

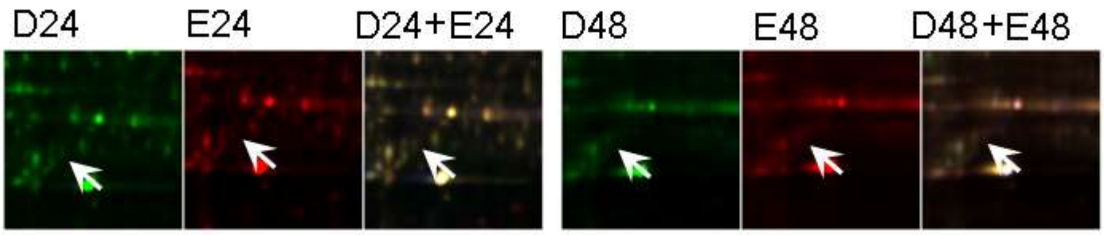

Accession No.: **scaffold0755_254783.mRNA1**

Plant species: ***Hevea brasiliensis***

Protein name: **2-alkenal reductase (NADP(+)-dependent)**

Peptide sequences: **K.IDEVLVAFGVGR.V; R.MEGFLVGSYLNR.F; R.YSEGDIVLNPYFPIAEYCVMPSDSLIR.K**

PFF Mascot score: **[124]** Sequence coverage %: **[13]**

Matched peptides No.: **[3]**

Calculated Mr: **42640** Calculated *p*I: **5.95**

Annotated PFF **spectra:**

Ions score is -10*Log(P), where P is the probability that the observed match is a random event.
Individual ions scores > 30 indicate identity or extensive homology (p<0.05).
Protein scores are derived from ions scores as a non-probabilistic basis for ranking protein hits.

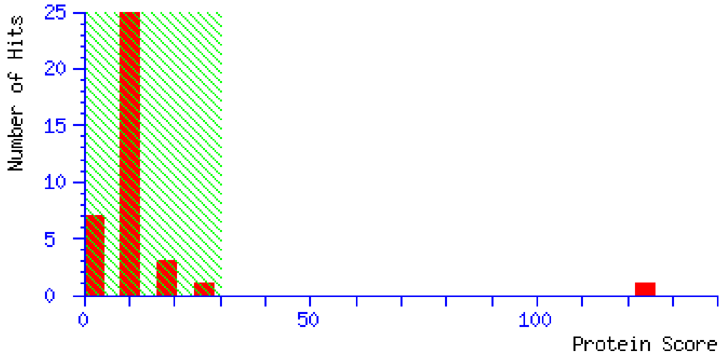

Matched peptide sequences: shown in Bold Red

```
  1 MYFKRQSGES ISNRFYKKVC QAILTEQSTM AGDGTEKATV IEGKEWYMAA
 51 YAPQGVPSSD HLKLRTVTIS LADDSIPDGH VAVELLWISV DPYLRSRMTG
101 HQDGLYMPQF KIDEVLVAFG VGRVIKSKDS RYSEGDIVLN PYFPIAEYCV
151 MPSDSLIRKI DVTAGIAIPD YLNALGAPGF AAWVGIVVLG EAKPGLNVFV
201 SAAAGGVGMF AGQLAKLRGC RVIGSTGSDD KVKLLKEEFG YDDAFNYKRE
251 TDYDAALSKY FPNGIDVYLD NVGGKMLEAV LNHINIGGRI PLCGMISEYN
301 KSWKERDGVR NLLNLVGKNV RMEGFLVGSY LNRFQDFLKE MEDCLLQGKI
351 ISKTKTYNGI DSFLESLGSL FSSTNNGKVV IQVKE
```

Matched peptide information:

Start - End	Observed	Mr(expt)	Mr(calc)	ppm	Miss	Sequence
112 - 123	1274.7250	1273.7177	1273.7030	12	0	K.IDEVLVAFGVGR.V (Ions score 41)
132 - 158	3148.5266	3147.5193	3147.4933	8	0	R.YSEGDIVLNPYFPIAEYCVMPSDSLIR.K (Ions score 24)
322 - 333	1385.7046	1384.6973	1384.6809	12	0	R.MEGFLVGSYLNR.F (Ions score 58)

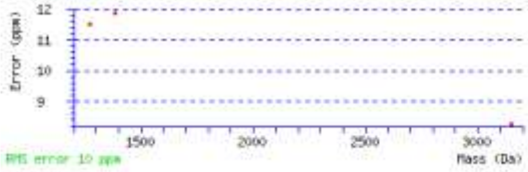

Spot No.: **14**

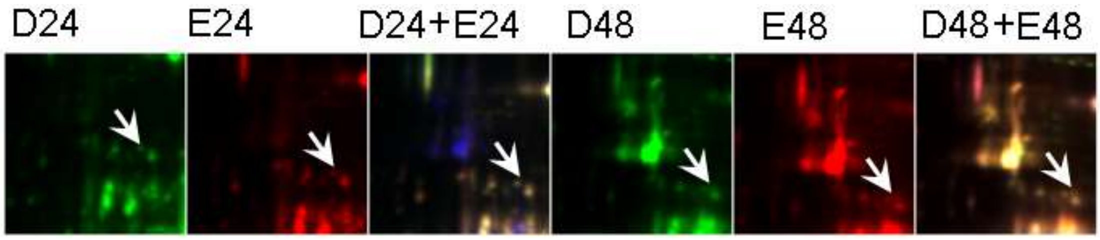

Accession No.: **scaffold0008_570089.mRNA1**

Plant species: ***Hevea brasiliensis***

Protein name：**Proteasome subunit alpha type-1-B**

Peptide sequences: **R.NQYDTDVTTWSPAGR.L；**
R.SECINYSFTYESPLPVGR.L；R.RFENFADSSR.D；R.FENFADSSR.D

PFF Mascot score: **[196]** Sequence coverage %: **[15]**

Matched peptides No.: **[4]**

Calculated Mr: **30852** Calculated *p*I: **4.76**

Annotated PFF **spectra:**

Ions score is -10*Log(P), where P is the probability that the observed match is a random event.
Individual ions scores > 31 indicate identity or extensive homology (p<0.05).
Protein scores are derived from ions scores as a non-probabilistic basis for ranking protein hits.

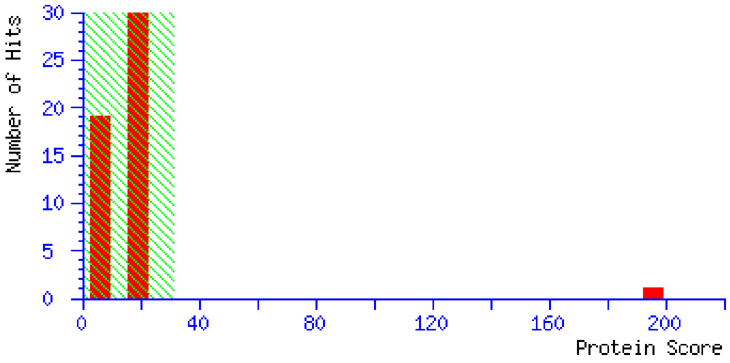

Matched peptide sequences: shown in Bold Red

```
  1 MFRNQYDTDV TTWSPAGRLF QVEYAMEAVK QGSAAIGLRS KTHVVLACVN
 51 KANSELSSHQ KKIFKVDDHI GVAIAGLTAD GRVLSRYMRS ECINYSFTYE
101 SPLPVGRLVV QLADKAQVCT QRSWKRPYGV GLLVAGLDES GAHLYYNCPS
151 GNYFEYQAFA IGSRSQAAKT YLERRFENFA DSSRDDLIKD ALIAVRETLQ
201 GETLKSSICT VAVVGVGEAF HVLDQDTVQQ LIDAFEIVGE PEGPAAEPEP
251 EPEPEPDAAA EEGTTADQGA APDQGVAPMD I
```

Matched peptide information:

Start - End	Observed	Mr(expt)	Mr(calc)	ppm	Miss	Sequence
4 - 18	1710.7490	1709.7417	1709.7645	-13	0	R.NQYDTDVTTWSPAGR.L (Ions score 105)
90 - 107	2118.9480	2117.9407	2117.9728	-15	0	R.SECINYSFTYESPLPVGR.L (Ions score 35)
175 - 184	1228.5477	1227.5404	1227.5632	-19	1	R.RFENFADSSR.D (Ions score 15)
176 - 184	1072.4532	1071.4459	1071.4621	-15	0	R.FENFADSSR.D (Ions score 42)

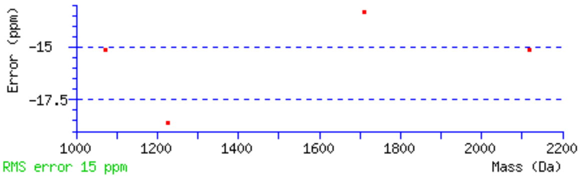

Spot No.: 15

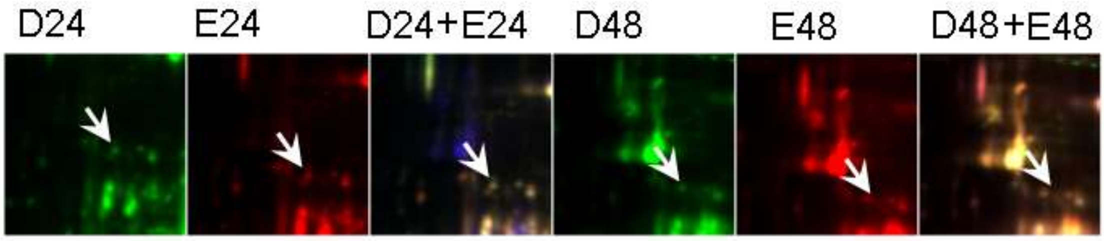

Accession No.: **scaffold0242_1127191.mRNA1**

Plant species: ***Hevea brasiliensis***

Protein name：**Ran-binding protein 1 homolog b**

Peptide sequences: **K.LEEVALTTGEEDEDPILDLK.A; R.FDKEGNQWK.E; K.SCVWHAADFADGELKDELFCIR.F**

PFF Mascot score: **[223]** Sequence coverage %: **[22]**

Matched peptides No.: **[3]**

Calculated Mr: **25725** Calculated *p*I: **4.77**

Annotated PFF **spectra:**

Ions score is -10*Log(P), where P is the probability that the observed match is a random event.
Individual ions scores > 30 indicate identity or extensive homology (p<0.05).
Protein scores are derived from ions scores as a non-probabilistic basis for ranking protein hits.

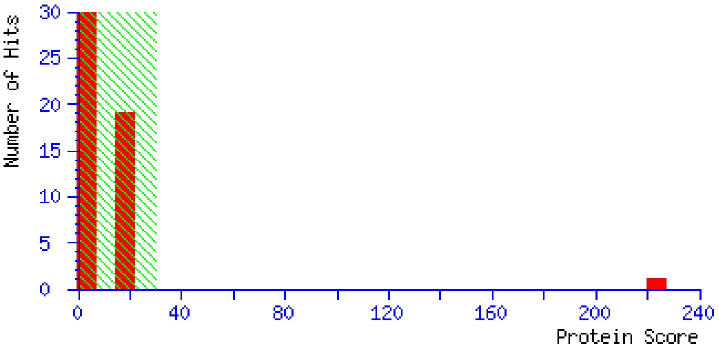

Matched peptide sequences: shown in **Bold Red**

```
  1 MASTASAADP EHNTKDREED ENAPAADDED TGAQVAPIVK LEEVALTTGE
 51 EDEDPILDLK AKLYRFDKEG NQWKERGVGN VKLLKHKESG KVRLVMRQSK
101 TLKICANHLV VPSINVQEHH GNDKSCVWHA ADFADGELKD ELFCIRFPSV
151 ENCKTFMETM QEVAETQGKK EESKEAADAA GLLEKLSVVD GKTDEKEKEE
201 GPVEAKEQHN EAEGEKAKAD AKKEDEIALS A
```

Matched peptide information:

Start - End	Observed	Mr(expt)	Mr(calc)	ppm	Miss	Sequence
41 - 60	2229.0774	2228.0701	2228.0947	-11	0	K.LEEVALTTGEEDEDPILDLK.A (Ions score 30)
66 - 74	1151.5190	1150.5117	1150.5407	-25	1	R.FDKEGNQWK.E (Ions score 52)
125 - 146	2639.2180	2638.2107	2638.1944	6	1	K.SCVWHAADFADGELKDELFCIR.F (Ions score 142)

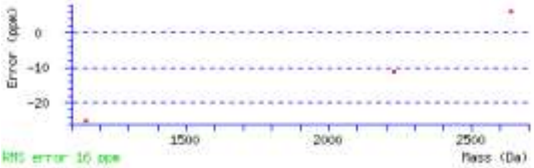

Spot No.: **16**

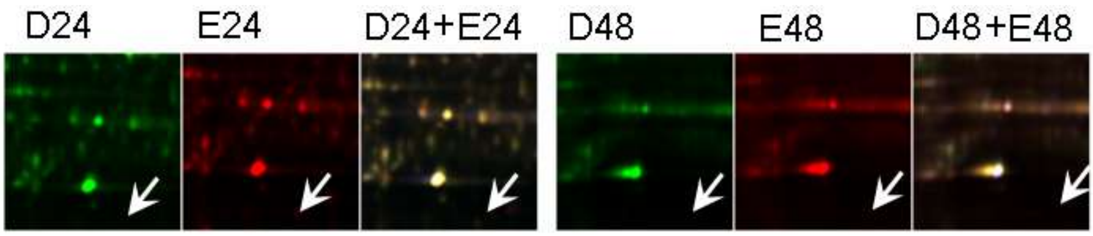

Accession No.: **scaffold0160_1784930.mRNA1**

Plant species: ***Hevea brasiliensis***

Protein name：**Vacuolar protein sorting-associated protein 24 homolog 1**

Peptide sequences: **K.AQMNSISMHLGESVAIAR.T；K.VLTAIAGETAAQLPEAVR.K；R.TAEEEEAIAEGVDDEEELEEIR.A**

PFF Mascot score: **[165]** Sequence coverage %: **[25]**

Matched peptides No.: **[3]**

Calculated Mr: **26063** Calculated *p*I: **5.34**

Annotated PFF **spectra:**

Ions score is -10*Log(P), where P is the probability that the observed match is a random event.
Individual ions scores > 31 indicate identity or extensive homology (p<0.05).
Protein scores are derived from ions scores as a non-probabilistic basis for ranking protein hits.

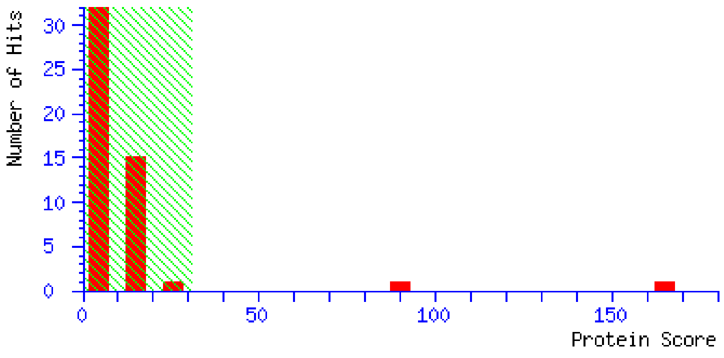

Matched peptide sequences: shown in **Bold Red**

```
  1 MEKMMNMIKP KQNPQQQLRD WQRRLRQECR NIERQIRDIQ KEEKSVQKAI
 51 RDAAKRNDMG SAKSLAKEIV RSRRTVNRLY ENKAQMNSIS MHLGESVAIA
101 RTVGHLSKSS EVMKLVNNLM KAPEVAATMQ EFSKEMTKAG VIEEFVNDAV
151 DNALDSEDIE EEIEEEVDKV LTAIAGETAA QLPEAVRKER VKQSAGTART
201 AEEEEAIAEG VDDEEELEEI RARLAKVRS
```

Bold red (matched) residues: 84–101 (AQMNSIS MHLGESVAIA R), 170–187 (V LTAIAGETAA QLPEAVR), 200–221 (T AEEEEAIAEG VDDEEELEEI R).

Matched peptide information:

Start - End	Observed	Mr(expt)	Mr(calc)	ppm	Miss	Sequence	
84 - 101	1914.9628	1913.9555	1913.9451	5	0	K.AQMNSISMHLGESVAIAR.T	(Ions score 14)
170 - 187	1810.0226	1809.0153	1808.9996	9	0	K.VLTAIAGETAAQLPEAVR.K	(Ions score 50)
200 - 221	2505.1316	2504.1243	2504.0925	13	0	R.TAEEEEAIAEGVDDEEELEEIR.A	(Ions score 100)

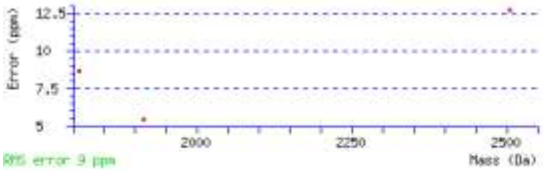

Spot No.: 17

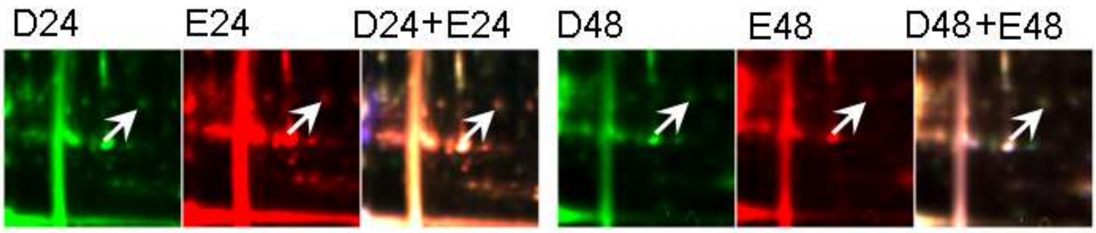

Accession No.: scaffold0457_538964.mRNA1

Plant species: *Hevea brasiliensis*

Protein name：Alpha-soluble NSF attachment protein 2

Peptide sequences: K.HEAAQAYVDAAHCYK.K；K.KTSTNEAISCLGQAVDLFCDIGR.I；K.TSTNEAISCLGQAVDLFCDIGR.I；K.AADFFQGEEVTTSANQCK.Q；K.AIEIYEEIAR.Q；R.YQDLDPTFSGTR.D

PFF Mascot score: [565]

Sequence coverage %: [26]

Matched peptides No.: [6]

Calculated Mr: 32767

Calculated *p*I: 5.05

Annotated PFF **spectra:**

Ions score is -10*Log(P), where P is the probability that the observed match is a random event.
Individual ions scores > 31 indicate identity or extensive homology (p<0.05).
Protein scores are derived from ions scores as a non-probabilistic basis for ranking protein hits.

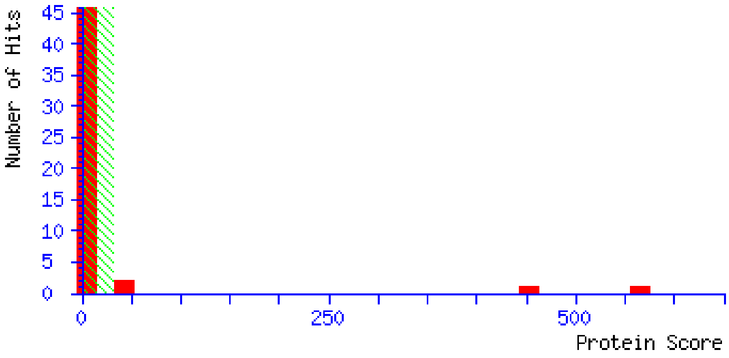

Matched peptide sequences: shown in **Bold Red**

```
  1 MGDQIARGEE FEKKAEKKLN GWGLFGSKFE DAADLFDKAA NSFKLAKSWD
 51 KAGSTYVKLA NCHLKLDSKH EAAQAYVDAA HCYKKTSTNE AISCLGQAVD
101 LFCDIGRISM AARYYKEIGE LYESEANFEK AIDFYEKAAD FFQGEEVTTS
151 ANQCKQKVAQ FAAQLEQYQK AIEIYEEIAR QSLGNNLLKY GVKGHLLNAG
201 ICHLCKGDVV AITNALERYQ DLDPTFSGTR DYKLLADIAA AIDEEDIAKF
251 TDVVKEFDSM TPLDSWKTTL LLRVKEKLKA KELEEDDLT
```

Bold red (matched) stretches: 70–84 (HEAAQAYVDAAHCYK), 86–107 (TSTNEAISCLGQAVDLFCDIGR), 138–155 (AADFFQGEEVTTSANQCK), 171–180 (AIEIYEEIAR), 219–230 (YQDLDPTFSGTR).

Matched peptide information:

Start - End	Observed	Mr(expt)	Mr(calc)	ppm	Miss	Sequence
70 - 84	1733.6727	1732.6654	1732.7627	-56	0	K.HEAAQAYVDAAHCYK.K (Ions score 131)
85 - 107	2555.0713	2554.0640	2554.2156	-59	1	K.KTSTNEAISCLGQAVDLFCDIGR.I (Ions score 29)
86 - 107	2426.9995	2425.9922	2426.1206	-53	0	K.TSTNEAISCLGQAVDLFCDIGR.I (Ions score 129)
138 - 155	2002.7664	2001.7591	2001.8738	-57	0	K.AADFFQGEEVTTSANQCK.Q (Ions score 113)
171 - 180	1206.5730	1205.5657	1205.6292	-53	0	K.AIEIYEEIAR.Q (Ions score 71)
219 - 230	1399.5734	1398.5661	1398.6416	-54	0	R.YQDLDPTFSGTR.D (Ions score 93)

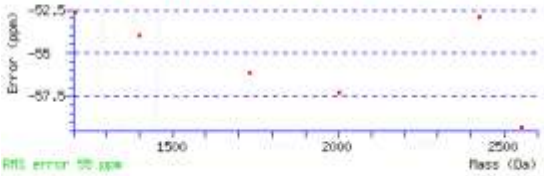

Spot No.: **18**

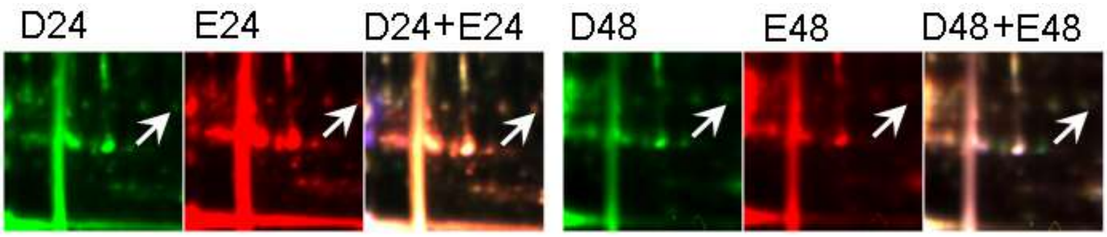

Accession No.: **scaffold0041_2570834.mRNA1**

Plant species: ***Hevea brasiliensis***

Protein name： **Aspartic proteinase A1**

Peptide sequences: **K.NYMDAQYFGEIGIGTPPQK.F；K.CYFSVACYFHSR.Y；K.SADIHYGTGAISGFFSQDHVK.V；K.EPVFSFWFNR.N；R.NADEDEGGEIVFGGMDPK.H；K.GEHTYVPVTQK.G**

PFF Mascot score: **[178]** Sequence coverage %: **[17]**

Matched peptides No.: **[6]**

Calculated Mr: **56526** Calculated *p*I: **5.73**

Annotated PFF **spectra:**

Ions score is -10*Log(P), where P is the probability that the observed match is a random event.
Individual ions scores > 31 indicate identity or extensive homology (p<0.05).
Protein scores are derived from ions scores as a non-probabilistic basis for ranking protein hits.

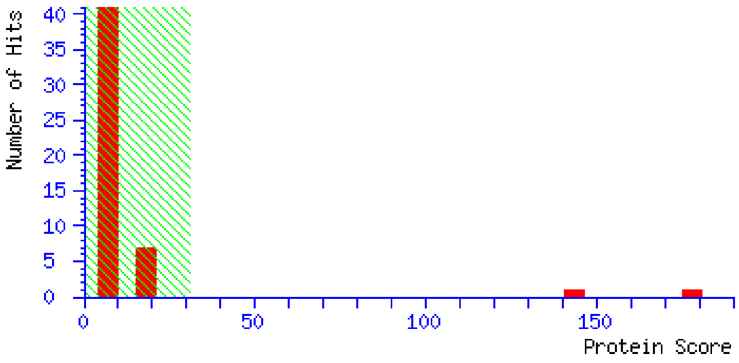

Matched peptide sequences: shown in **Bold Red**

```
  1 MGTRSKPITT ALFLCFLLLP LVFAAHNDGL VRIGLKKRKF DQNNLVAAQF
 51 ESKEGESLRT SFKKYHFHGN LGDAEDIDII SLKNYMDAQY FGEIGIGTPP
101 QKFTVIFDTG SSNLWVPSSK CYFSVACYFH SRYKSGHSST YKKNGKSADI
151 HYGTGAISGF FSQDHVKVGG LVVKDQEFIE ATKEPSITFL VAKFDGILGL
201 GFEEISVGKA VPVWYNMVNQ GLVKEPVFSF WFNRNADEDE GGEIVFGGMD
251 PKHYKGEHTY VPVTQKGYWQ FNMGDVLIDG KTTGICGSGC AAIADSGTSL
301 LAGPTGIIAE VNHAIGATGI VSQECKAVVA QYGETIIEML LAKDQPQKIC
351 SQIGLCTFDG SRGVSVGIKS VVNENIQGVA SGLRDAMCST CEMAVVWMQN
401 QLKLNNTQER ILNYANELCE RLPSPMGESA VDCGGLSTMP NVSFTIGGKV
451 FDLSPEQYVL KVGEGEAAQC ISGFTALDVP PPRGPLWILG DVFMGRFHTV
501 FDYGNKRVGF AEAA
```

Matched peptide information:

Start - End	Observed	Mr(expt)	Mr(calc)	ppm	Miss	Sequence
84 - 102	2145.0759	2144.0686	2143.9884	37	0	K.NYMDAQYFGEIGIGTPPQK.F Oxidation (M) (Ions score 35)
121 - 132	1596.7429	1595.7356	1595.6650	44	0	K.CYFSVACYFHSR.Y (Ions score 25)
147 - 167	2237.1594	2236.1521	2236.0549	43	0	K.SADIHYGTGAISGFFSQDHVK.V (Ions score 20)
225 - 234	1328.7057	1327.6984	1327.6350	48	0	K.EPVFSFWFNR.N (Ions score 53)
235 - 252	1895.8605	1894.8532	1894.7891	34	0	R.NADEDEGGEIVFGGMDPK.H Oxidation (M) (Ions score 16)
256 - 266	1258.6754	1257.6681	1257.6354	26	0	K.GEHTYVPVTQK.G (Ions score 29)

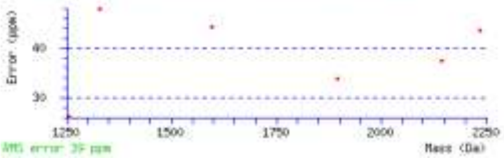

Spot No.: **19**

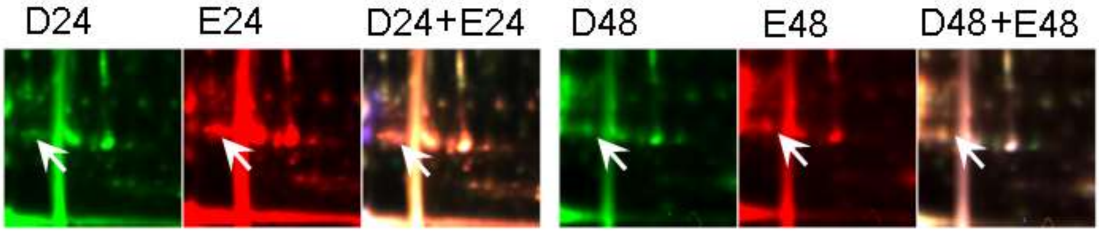

Accession No.: **scaffold0153_1834215.mRNA1**

Plant species: ***Hevea brasiliensis***

Protein name： **26S proteasome non-ATPase regulatory subunit 8 homolog A**

Peptide sequences: **R.SLPPLFEDTPNAIHELTLAR.D；R.DFFQLKPYYTDASGR.L；R.LPPSSQEYMILGLNLLR.L；R.IAEFHTELELLSPTALENPCIK.H；K.EIPSLQLINQTLSYAR.E**

PFF Mascot score: **[525]** Sequence coverage %: **[33]**

Matched peptides No.: **[5]**

Calculated Mr: **30835** Calculated *p*I: **4.87**

Annotated PFF **spectra:**

Ions score is -10*Log(P), where P is the probability that the observed match is a random event.
Individual ions scores > 31 indicate identity or extensive homology (p<0.05).
Protein scores are derived from ions scores as a non-probabilistic basis for ranking protein hits.

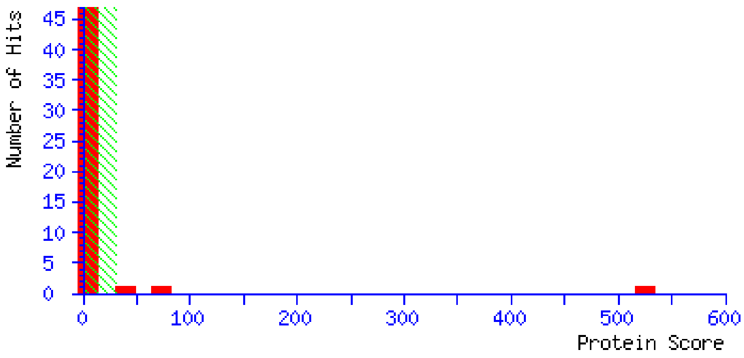

Matched peptide sequences: shown in **Bold Red**

```
  1 MDPKLTEVSQ LFERFKAACV REDVDTGSNL LSKLKVMLTG FRSLPPLFED
 51 TPNAIHELTL ARDIYEHAVL LSVKIGDQDA FERDFFQLKP YYTDASGRLP
101 PSSQEYMILG LNLLRLLVQN RIAEFHTELE LLSPTALENP CIKHAVELEQ
151 SFMEGAYNRV LSAKQTVPYK TYDYFMDLLA KTVRDEIAGC SEKAYDYLSI
201 SDARQMLLFS SDDELLQYIN EEHPEWEIKN GVVIFQKAKE SAPCKEIPSL
251 QLINQTLSYA RELERIV
```

Matched peptide information:

Start - End	Observed	Mr(expt)	Mr(calc)	ppm	Miss	Sequence
43 - 62	2234.1008	2233.0935	2233.1743	-36	0	R.SLPPLFEDTPNAIHELTLAR.D (Ions score 106)
84 - 98	1807.8010	1806.7937	1806.8577	-35	1	R.DFFQLKPYYTDASGR.L (Ions score 117)
99 - 115	1943.9922	1942.9849	1943.0550	-36	0	R.LPPSSQEYMILGLNLLR.L (Ions score 104)
122 - 143	2525.2039	2524.1966	2524.2883	-36	0	R.IAEFHTELELLSPTALENPCIK.H (Ions score 67)
246 - 261	1845.9424	1844.9351	1844.9996	-35	0	K.EIPSLQLINQTLSYAR.E (Ions score 190)

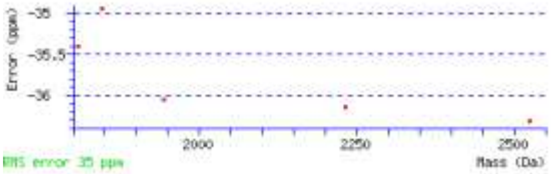

Spot No.: **20**

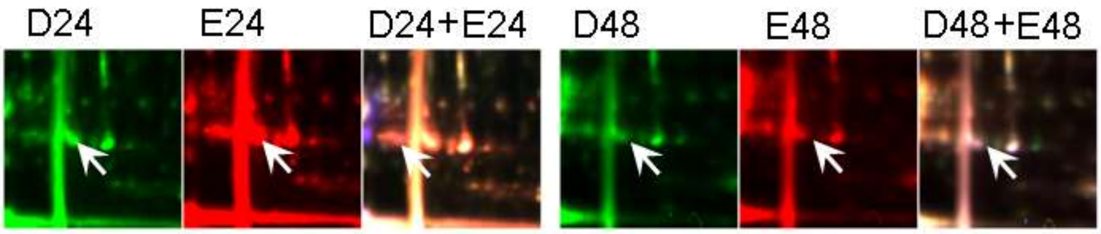

Accession No.: **scaffold1222_175215.mRNA1**

Plant species: ***Hevea brasiliensis***

Protein name：**Rubber elongation factor protein**

Peptide sequences: **K.SVVRPFYYR.F；K.FADNQVDASVTLVLR.Y；R.AALALVSYLPLPTNR.L**

PFF Mascot score: **[274]**

Sequence coverage %: **[17]**

Matched peptides No.: **[3]**

Calculated Mr: **23621**

Calculated *p*I: **5.33**

Annotated PFF **spectra:**

Ions score is -10*Log(P), where P is the probability that the observed match is a random event.
Individual ions scores > 31 indicate identity or extensive homology (p<0.05).
Protein scores are derived from ions scores as a non-probabilistic basis for ranking protein hits.

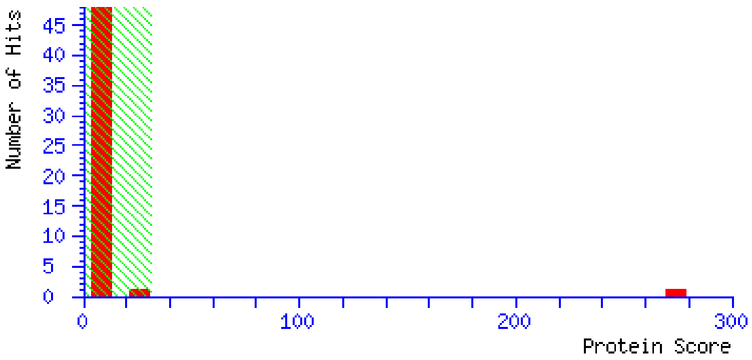

Matched peptide sequences: shown in **Bold Red**

```
  1 MASLLGAASN VINAASNVVE EAVKGVGNAQ QEVANAVSNP SNIVKDVASA
 51 ATDIVEEAAK VVDNVQQGVV SAASNVVEEA AKGVGNIQEK VDDEEEDTLK
101 YLDIVQAALV LALVSSSKLY LFVKDKSGPL KPGVDTAEVT IKSVVRPFYY
151 RFHDVPNKVL KFADNQVDAS VTLVLRYAPP VVKQVSTRAY SVARNAPRAA
201 LALVSYLPLP TNRLCKLLSE DK
```

Bold red (matched): 143–151 SVVRPFYYR; 162–176 FADNQVDASVTLVLR; 199–213 AALALVSYLPLPTNR

Matched peptide information:

Start - End	Observed	Mr(expt)	Mr(calc)	ppm	Miss	Sequence	
143 - 151	1186.5686	1185.5613	1185.6295	-57	1	K.SVVRPFYYR.F	(Ions score 38)
162 - 176	1647.7781	1646.7708	1646.8628	-56	0	K.FADNQVDASVTLVLR.Y	(Ions score 130)
199 - 213	1598.8372	1597.8299	1597.9191	-56	0	R.AALALVSYLPLPTNR.L	(Ions score 106)

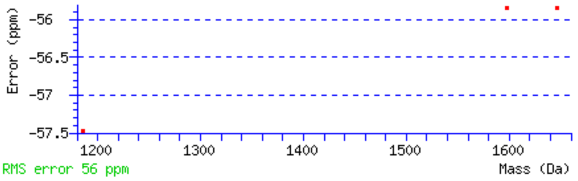

Spot No.: **21**

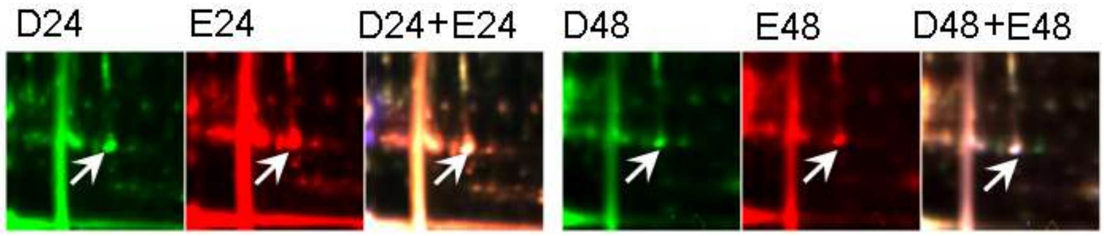

Accession No.: **scaffold1222_175215.mRNA1**

Plant species: ***Hevea brasiliensis***

Protein name：**Rubber elongation factor protein**

Peptide sequences: **K.VVDNVQQGVVSAASNVVEEAAK.G；K.YLDIVQAALVLALVSSSK.L；K.SVVRPFYYR.F；K.FADNQVDASVTLVLR.Y；R.AALALVSYLPLPTNR.L**

PFF Mascot score: **[524]**　Sequence coverage %: **[35]**

Matched peptides No.: **[5]**

Calculated Mr: **23621**　Calculated *p*I: **5.33**

Annotated PFF **spectra:**

Ions score is -10*Log(P), where P is the probability that the observed match is a random event.
Individual ions scores > 31 indicate identity or extensive homology (p<0.05).
Protein scores are derived from ions scores as a non-probabilistic basis for ranking protein hits.

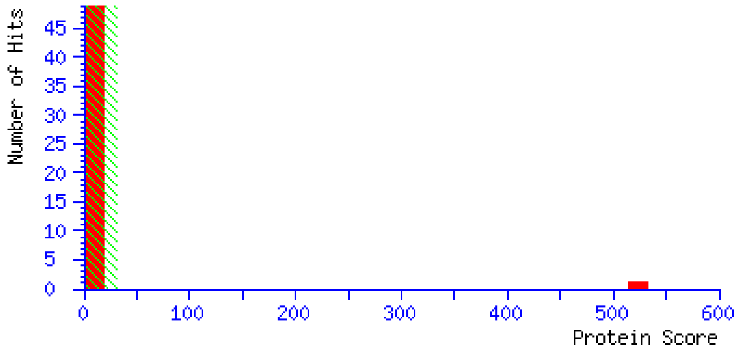

Matched peptide sequences: shown in **Bold Red**

```
  1 MASLLGAASN VINAASNVVE EAVKGVGNAQ QEVANAVSNP SNIVKDVASA
 51 ATDIVEEAAK VVDNVQQGVV SAASNVVEEA AKGVGNIQEK VDDEEEDTLK
101 YLDIVQAALV LALVSSSKLY LFVKDKSGPL KPGVDTAEVT IKSVVRPFYY
151 RFHDVPNKVL KFADNQVDAS VTLVLRYAPP VVKQVSTRAY SVARNAPRAA
201 LALVSYLPLP TNRLCKLLSE DK
```

Matched peptide information:

Start - End	Observed	Mr(expt)	Mr(calc)	ppm	Miss	Sequence
61 - 82	2212.9980	2211.9907	2212.1335	-65	0	K.VVDNVQQGVVSAASNVVEEAAK.G (Ions score 161)
101 - 118	1889.9652	1888.9579	1889.0873	-69	0	K.YLDIVQAALVLALVSSSK.L (Ions score 66)
143 - 151	1186.5707	1185.5634	1185.6295	-56	1	K.SVVRPFYYR.F (Ions score 39)
162 - 176	1647.7740	1646.7667	1646.8628	-58	0	K.FADNQVDASVTLVLR.Y (Ions score 150)
199 - 213	1598.8350	1597.8277	1597.9191	-57	0	R.AALALVSYLPLPTNR.L (Ions score 109)

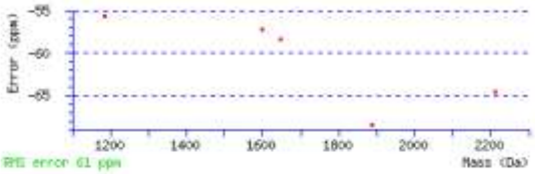

Spot No.: 22

D24 E24 D24+E24 D48 E48 D48+E48

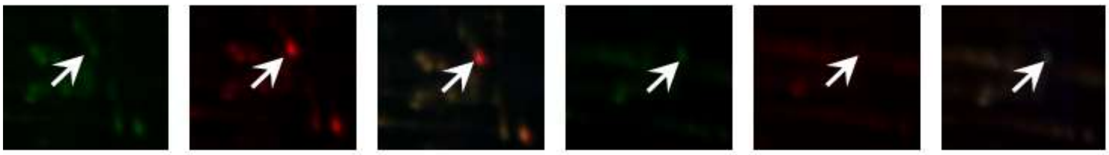

Accession No.: scaffold2538_3915.mRNA1

Plant species: *Hevea brasiliensis*

Protein name：REF/SRPP-like protein At3g05500

Peptide sequences: K.AGPLKPGVETVEGTVK.S；K.AEQCAVTAWR.R；R.RLNQLPLFPQVAQVVVPTAAYCSEK.Y；R.VSSYLPLVPTER.I

PFF Mascot score: [267] Sequence coverage %: [25]

Matched peptides No.: [4]

Calculated Mr: 27100 Calculated *p*I: 6.36

Annotated PFF **spectra:**

Ions score is -10*Log(P), where P is the probability that the observed match is a random event.
Individual ions scores > 31 indicate identity or extensive homology ($p<0.05$).
Protein scores are derived from ions scores as a non-probabilistic basis for ranking protein hits.

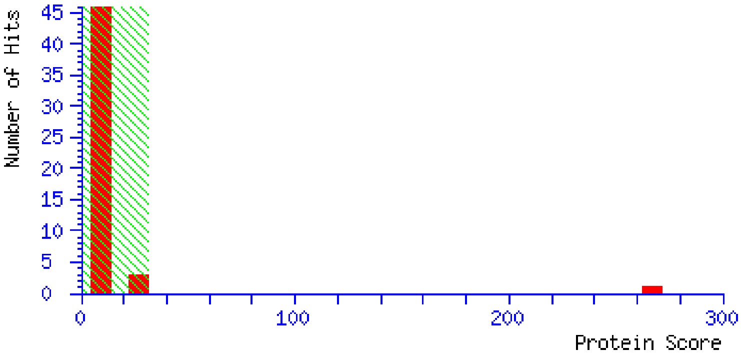

Matched peptide sequences: shown in **Bold Red**

```
  1 MAEGEGNVNM QQQMENEEEE RLKYLEFVQV AVIHAVVTFT NLYLYAKEKA
 51 GPLKPGVETV EGTVKSVVGP VYYKFHDVPN EVLKFVDRKV DESVTSLDSR
101 VPPVVKQVSA QAYSVAREAP VAARAVASEV HQSGVKETAS GLAKTLYTKY
151 EPKAKELYSK YEPKAEQCAV TAWRRLNQLP LFPQVAQVVV PTAAYCSEKY
201 NQTVLSTFEK GYRVSSYLPL VPTERIAKVF SDDVAQSMPL VSS
```

Matched peptide information:

Start - End	Observed	Mr(expt)	Mr(calc)	ppm	Miss	Sequence
50 - 65	1581.8340	1580.8267	1580.8774	-32	1	K.AGPLKPGVETVEGTVK.S (Ions score 96)
165 - 174	1191.5222	1190.5149	1190.5502	-30	0	K.AEQCAVTAWR.R (Ions score 55)
175 - 199	2828.4136	2827.4063	2827.5055	-35	1	R.RLNQLPLFPQVAQVVVPTAAYCSEK.Y (Ions score 34)
214 - 225	1360.7096	1359.7023	1359.7398	-28	0	R.VSSYLPLVPTER.I (Ions score 84)

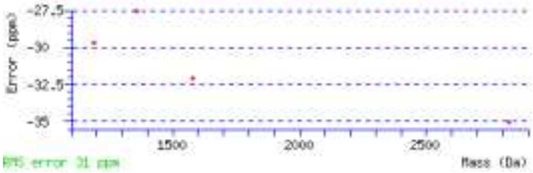

Spot No.: **23**

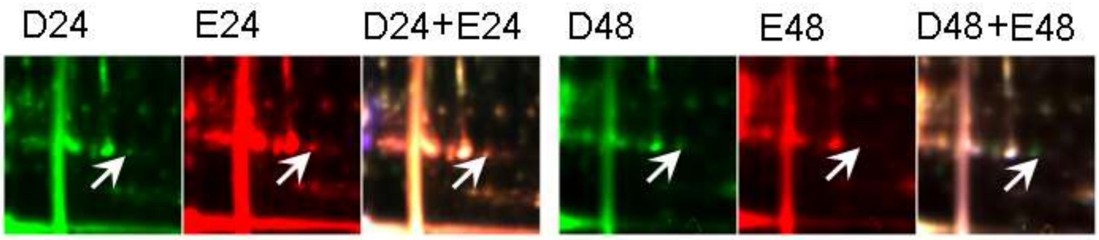

Accession No.: **scaffold1222_175215.mRNA1**

Plant species: ***Hevea brasiliensis***

Protein name： **Rubber elongation factor protein**

Peptide sequences: **K.DVASAATDIVEEAAK.V； R.AALALVSYLPLPTNR.L； K.VVDNVQQGVVSAASNVVEEAAK.G；K.SVVRPFYYR.F；K.FADNQVDASVTLVLR.Y；**

PFF Mascot score: **[483]**

Sequence coverage %: **[34]**

Matched peptides No.: **[5]**

Calculated Mr: **23621**

Calculated *p*I: **5.33**

Annotated PFF **spectra:**

Ions score is -10*Log(P), where P is the probability that the observed match is a random event.
Individual ions scores > 31 indicate identity or extensive homology (p<0.05).
Protein scores are derived from ions scores as a non-probabilistic basis for ranking protein hits.

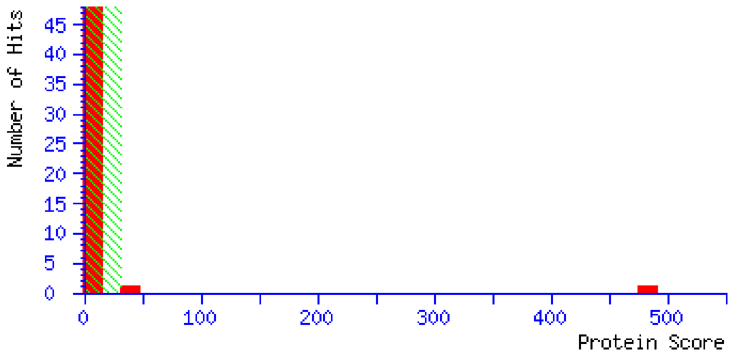

Matched peptide sequences: shown in **Bold Red**

1 MASLLGAASN VINAASNVVE EAVKGVGNAQ QEVANAVSNP SNIVK**DVASA**

51 **ATDIVEEAAK VVDNVQQGVV SAASNVVEEA AK**GVGNIQEK VDDEEEDTLK

101 YLDIVQAALV LALVSSSKLY LFVKDKSGPL KPGVDTAEVT IK**SVVRPFYY**

151 **R**FHDVPNKVL K**FADNQVDAS VTLVLR**YAPP VVKQVSTRAY SVARNAPR**AA**

201 **LALVSYLPLP TNR**LCKLLSE DK

Matched peptide information:

Start - End	Observed	Mr(expt)	Mr(calc)	ppm	Miss	Sequence
46 - 60	1489.6531	1488.6458	1488.7307	-57	0	K.DVASAATDIVEEAAK.V (Ions score 88)
61 - 82	2212.9885	2211.9812	2212.1335	-69	0	K.VVDNVQQGVVSAASNVVEEAAK.G (Ions score 119)
143 - 151	1186.5667	1185.5594	1185.6295	-59	1	K.SVVRPFYYR.F (Ions score 35)
162 - 176	1647.7722	1646.7649	1646.8628	-59	0	K.FADNQVDASVTLVLR.Y (Ions score 137)
199 - 213	1598.8346	1597.8273	1597.9191	-57	0	R.AALALVSYLPLPTNR.L (Ions score 104)

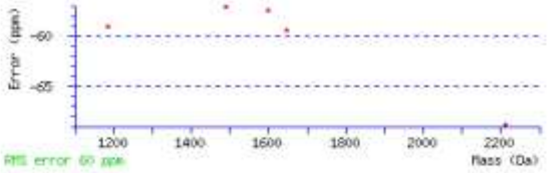

Spot No.: **24**

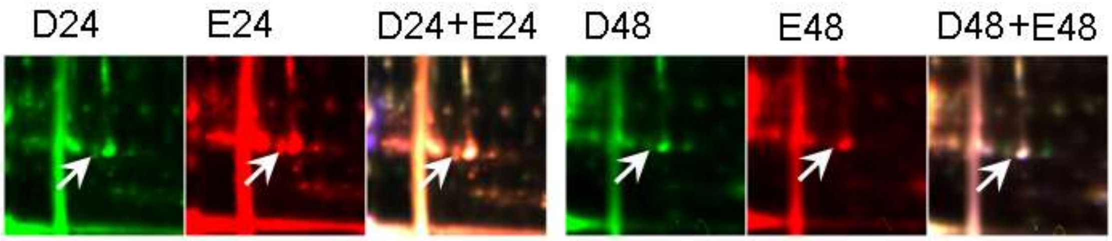

Accession No.: **scaffold1222_175215.mRNA1**

Plant species: ***Hevea brasiliensis***

Protein name：**Rubber elongation factor protein**

Peptide sequences: **K.SVVRPFYYR.F；K.FADNQVDASVTLVLR.Y；R.AALALVSYLPLPTNR.L**

PFF Mascot score: **[277]** Sequence coverage %: **[17]**

Matched peptides No.: **[3]**

Calculated Mr: **23621** Calculated *p*I: **5.33**

Annotated PFF **spectra:**

Ions score is -10*Log(P), where P is the probability that the observed match is a random event.
Individual ions scores > 31 indicate identity or extensive homology (p<0.05).
Protein scores are derived from ions scores as a non-probabilistic basis for ranking protein hits.

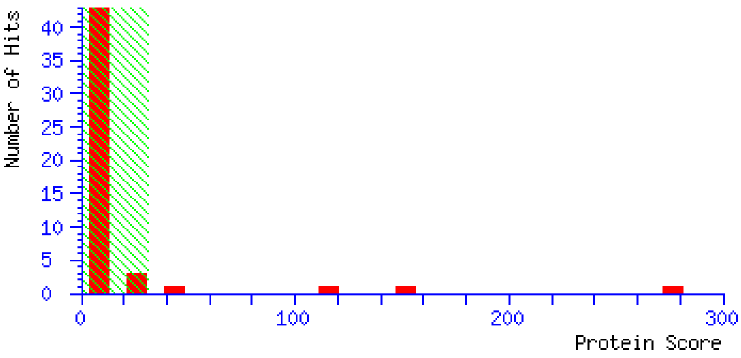

Matched peptide sequences: shown in **Bold Red**

```
  1 MASLLGAASN VINAASNVVE EAVKGVGNAQ QEVANAVSNP SNIVKDVASA
 51 ATDIVEEAAK VVDNVQQGVV SAASNVVEEA AKGVGNIQEK VDDEEEDTLK
101 YLDIVQAALV LALVSSSKLY LFVKDKSGPL KPGVDTAEVT IKSVVRPFYY
151 RFHDVPNKVL KFADNQVDAS VTLVLRYAPP VVKQVSTRAY SVARNAPRAA
201 LALVSYLPLP TNRLCKLLSE DK
```

Matched (bold red) residues: 143–151 (SVVRPFYYR), 162–176 (FADNQVDASVTLVLR), 199–213 (AALALVSYLPLPTNR).

Matched peptide information:

Start – End	Observed	Mr(expt)	Mr(calc)	ppm	Miss	Sequence	
143 – 151	1186.5507	1185.5434	1185.6295	-73	1	K.SVVRPFYYR.F	(Ions score 42)
162 – 176	1647.7527	1646.7454	1646.8628	-71	0	K.FADNQVDASVTLVLR.Y	(Ions score 142)
199 – 213	1598.8119	1597.8046	1597.9191	-72	0	R.AALALVSYLPLPTNR.L	(Ions score 93)

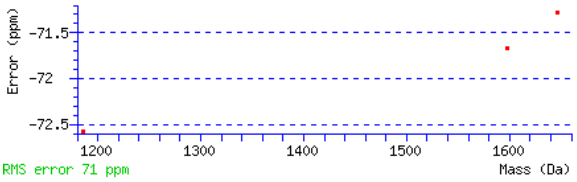

Spot No.: **25**

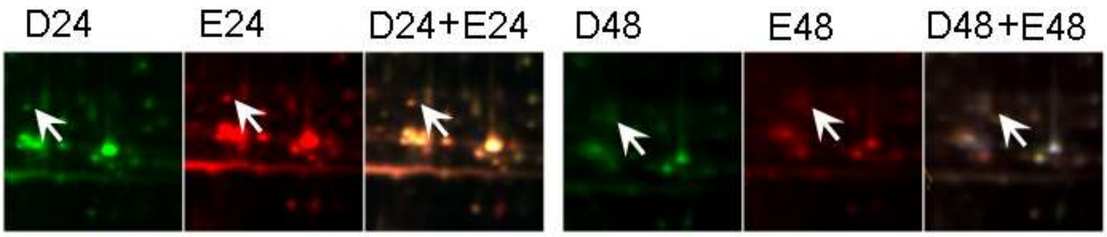

Accession No.: **scaffold0086_1407649.mRNA1**

Plant species: ***Hevea brasiliensis***

Protein name：**Glutathione S-transferase L3**

Peptide sequences: **K.IHLVPLNLQSR.P；K.YLDSNFEGQSLLPDDPAK.K；K.YLDSNFEGQSLLPDDPAKK.E；K.EFAEELFSYTDTFNK.T；K.EAGPAFDYLENALHK.F；K.FDDGPFLLGQFSLVDIAYIPFVER.F；R.FQIFLSEVFK.Y；K.YDITAGRPK.L；K.LAAWIEEINK.L**

PFF Mascot score: **[426]** Sequence coverage %: **[24]**

Matched peptides No.: **[9]**

Calculated Mr: **53345** Calculated *p*I: **5.95**

Annotated PFF **spectra:**

Ions score is -10*Log(P), where P is the probability that the observed match is a random event.
Individual ions scores > 31 indicate identity or extensive homology (p<0.05).
Protein scores are derived from ions scores as a non-probabilistic basis for ranking protein hits.

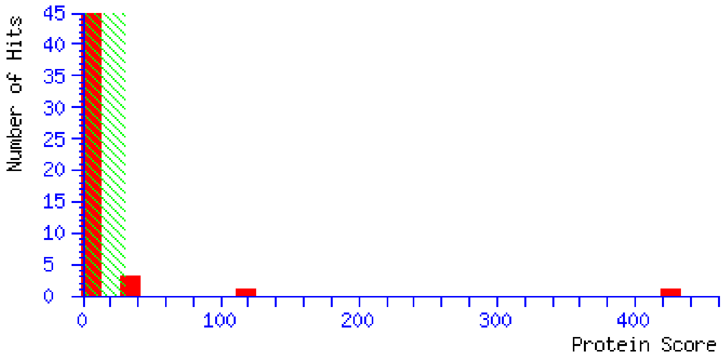

Matched peptide sequences: shown in **Bold Red**

```
  1 MAELQGLQDK **IHLVPLNLQS R**PSWYGEKVY SVNRVPALEH NGKIIGESLD
 51 LIK**YLDSNFE GQSLLPDDPA KKEFAEELFS YTDTFNK**TVF TSFKGDVAK**E**
101 **AGPAFDYLEN ALHKFDDGPF LLGQFSLVDI AYIPFVERFQ IFLSEVFKYD**
151 **ITAGRPKLAA WIEEINK**LGA YKQTKTDPKE LVEFYKKRIL GHLDKSVPEK
201 LPPVLDPTAE QPPLFDGTIR LYIAYICPYA QRVWIIRNYK GLQDKIKLIP
251 LNLQSRPAWY GEKVYPPNKV PSLEHNGKII GESLDLIKYL DSNFEGQSLL
301 PEDPAKREFA EELLAYSDTF NKIVFTSFKG DPAKEAGPAF DYLENALHKF
351 DDGPFLLGQF SLVDIAYIPF VERFHVFLLE VFKYDIIAGR PKLEAWIEEI
401 NKIEAYKQTK IDPKENVEAF KKRFLAIQYR RMGCALIRRK LRQCFIGLDP
451 QLCQRFVVSW V
```

Matched peptide information:

Start - End	Observed	Mr(expt)	Mr(calc)	ppm	Miss	Sequence	
11 - 21	1289.8518	1288.8445	1288.7615	64	0	K.IHLVPLNLQSR.P	(Ions score 51)
54 - 71	2009.0750	2008.0677	2007.9425	62	0	K.YLDSNFEGQSLLPDDPAK.K	(Ions score 43)
54 - 72	2137.1724	2136.1651	2136.0375	60	1	K.YLDSNFEGQSLLPDDPAKK.E	(Ions score 70)
73 - 87	1840.9390	1839.9317	1839.8203	61	0	K.EFAEELFSYTDTFNK.T	(Ions score 80)
100 - 114	1674.9232	1673.9159	1673.8049	66	0	K.EAGPAFDYLENALHK.F	(Ions score 85)
115 - 138	2758.5637	2757.5564	2757.4054	55	0	K.FDDGPFLLGQFSLVDIAYIPFVER.F	(Ions score 48)
139 - 148	1257.7480	1256.7407	1256.6805	48	0	R.FQIFLSEVFK.Y	(Ions score 29)
149 - 157	1020.6074	1019.6001	1019.5400	59	1	K.YDITAGRPK.L	(Ions score 7)
158 - 167	1186.7015	1185.6942	1185.6393	46	0	K.LAAWIEEINK.L	(Ions score 18)

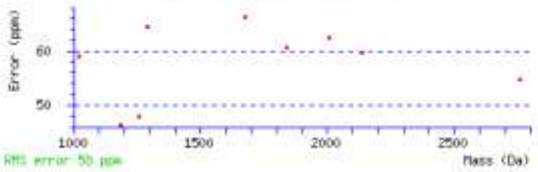

Spot No.: **26**

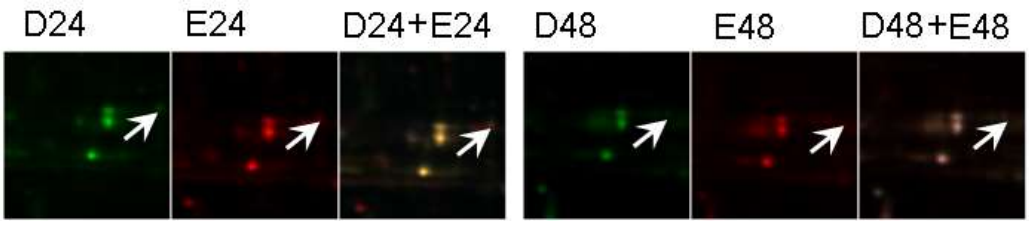

Accession No.: **scaffold0181_54255.mRNA1**

Plant species: ***Hevea brasiliensis***

Protein name：**Proteasome subunit alpha type-3**

Peptide sequences: **R.HSGMAVAGLAADGR.Q；**

K.SEATNYESVYGEPIPVNELAGR.V；R.DGPQLYMIEPSGISYR.Y；
K.AFELEMSWVCDESKR.L

PFF Mascot score: **[412]** Sequence coverage %: **[26]**

Matched peptides No.: **[4]**

Calculated Mr: **27595** Calculated p*I*: **6.11**

Annotated PFF **spectra:**

Ions score is -10*Log(P), where P is the probability that the observed match is a random event.
Individual ions scores > 31 indicate identity or extensive homology ($p<0.05$).
Protein scores are derived from ions scores as a non-probabilistic basis for ranking protein hits.

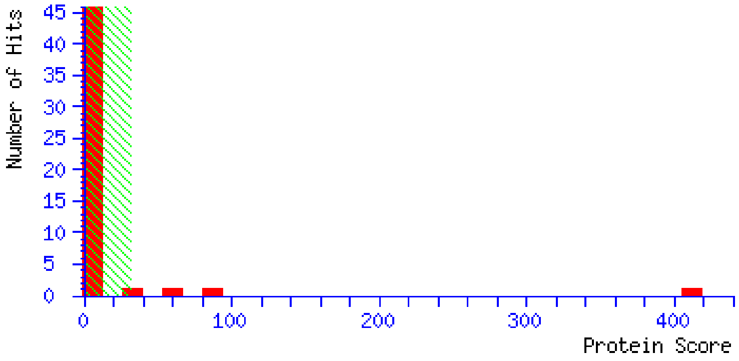

Matched peptide sequences: shown in Bold Red

1 MSSIGTGYDL SVTTFSPDGR VFQIEYAAKA VDNSGTVIGI KCKDGIVMGV
51 EKLIASKMML PGSNRRIHSV HR**HSGMAVAG LAADGR**QIVA RAK**SEATNYE**
101 **SVYGEPIPVN ELAGR**VASYV HLCTLYWWLR PFGCGVILGG YDR**DGPQLYM**
151 **IEPSGISYR**Y FGAAIGKGKQ AAKTEIEKLK LSEMTCREGV IEVAKIIYKV
201 HDEAKDK**AFE LEMSWVCDES KR**LHQKVPDE LLEEAKAAAR TALEEMDAD

Matched peptide information:

Start - End	Observed	Mr(expt)	Mr(calc)	ppm	Miss	Sequence
73 - 86	1312.6466	1311.6393	1311.6354	3	0	R.HSGMAVAGLAADGR.Q (Ions score 86)
94 - 115	2395.1338	2394.1265	2394.1339	-3	0	K.SEATNYESVYGEPIPVNELAGR.V (Ions score 126)
144 - 159	1825.8751	1824.8678	1824.8716	-2	0	R.DGPQLYMIEPSGISYR.Y (Ions score 93)
208 - 222	1886.8341	1885.8268	1885.8338	-4	1	K.AFELEMSWVCDESKR.L (Ions score 107)

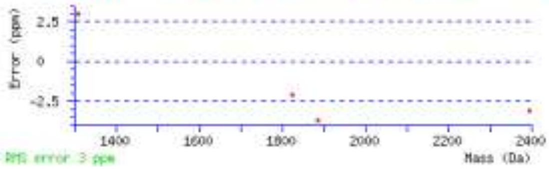

Spot No.: **27**

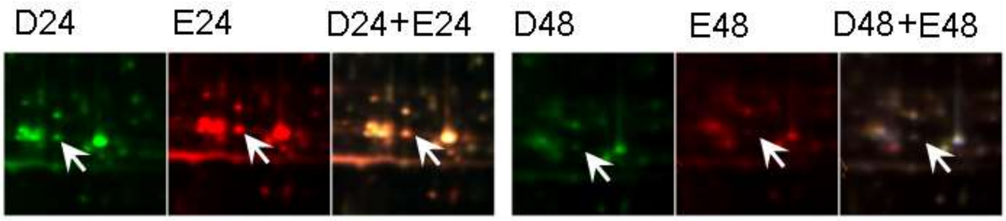

Accession No.: **scaffold1222_175215.mRNA1**

Plant species: ***Hevea brasiliensis***

Protein name：**Rubber elongation factor protein**

Peptide sequences: **K.GVGNAQQEVANAVSNPSNIVK.D；K.VVDNVQQGVVSAASNVVEEAAK.G；K.SVVRPFYYR.F；K.FADNQVDASVTLVLR.Y；R.AALALVSYLPLPTNR.L**

PFF Mascot score: **[467]**

Sequence coverage %: **[36]**

Matched peptides No.: **[5]**

Calculated Mr: **23621**

Calculated *p*I: **5.33**

Annotated PFF **spectra:**

Ions score is -10*Log(P), where P is the probability that the observed match is a random event.
Individual ions scores > 30 indicate identity or extensive homology (p<0.05).
Protein scores are derived from ions scores as a non-probabilistic basis for ranking protein hits.

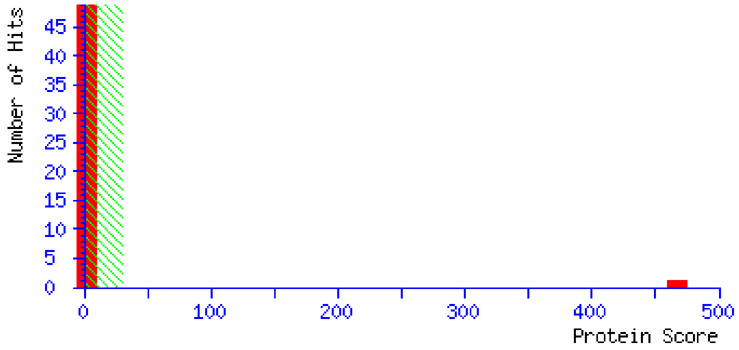

Matched peptide sequences: shown in **Bold Red**

1 MASLLGAASN VINAASNVVE EAVK**GVGNAQ** **QEVANAVSNP** **SNIVK**DVASA
51 ATDIVEEAAK **VVDNVQQGVV** **SAASNVVEEA** **AK**GVGNIQEK VDDEEEDTLK
101 YLDIVQAALV LALVSSSKLY LFVKDKSGPL KPGVDTAEVT IK**SVVRPFYY**
151 **R**FHDVPNKVL K**FADNQVDAS** **VTLVLR**YAPP VVKQVSTRAY SVARNAPR**AA**
201 **LALVSYLPLP** **TNR**LCKLLSE DK

Matched peptide information:

Start - End	Observed	Mr(expt)	Mr(calc)	ppm	Miss	Sequence
25 - 45	2096.0068	2094.9995	2095.0658	-32	0	K.GVGNAQQEVANAVSNPSNIVK.D (Ions score 78)
61 - 82	2213.0596	2212.0523	2212.1335	-37	0	K.VVDNVQQGVVSAASNVVEEAAK.G (Ions score 95)
143 - 151	1186.5925	1185.5852	1185.6295	-37	1	K.SVVRPFYYR.F (Ions score 36)
162 - 176	1647.8125	1646.8052	1646.8628	-35	0	K.FADNQVDASVTLVLR.Y (Ions score 153)
199 - 213	1598.8693	1597.8620	1597.9191	-36	0	R.AALALVSYLPLPTNR.L (Ions score 106)

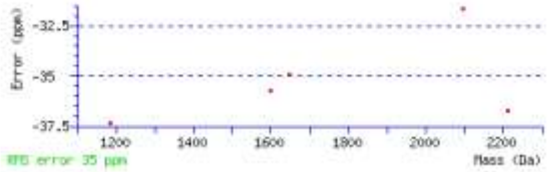

Spot No.: **28**

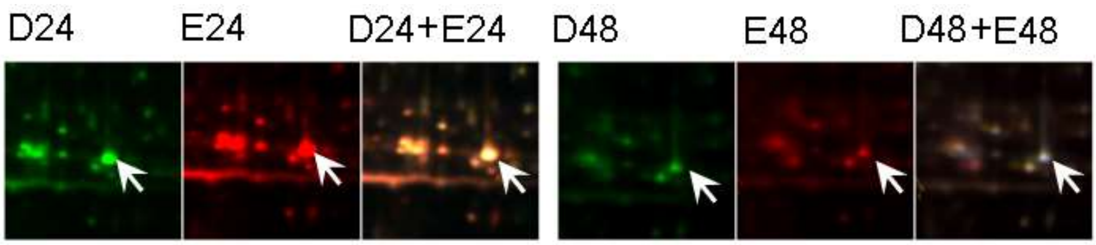

Accession No.: **scaffold0135_1578719.mRNA1**

Plant species: ***Hevea brasiliensis***

Protein name：**Probable glutathione S-transferase**

Peptide sequences: **K.YEYREEDLR.N；K.SPLLLQMNPVHK.K；R.FWADFIDK.K；R.FWADFIDKK.I；K.VYEFVLVLK.K**

PFF Mascot score: **[192]** Sequence coverage %: **[17]**

Matched peptides No.: **[5]**

Calculated Mr: **25563** Calculated *p*I: **6.23**

Annotated PFF **spectra:**

Ions score is -10*Log(P), where P is the probability that the observed match is a random event.
Individual ions scores > 31 indicate identity or extensive homology (p<0.05).
Protein scores are derived from ions scores as a non-probabilistic basis for ranking protein hits.

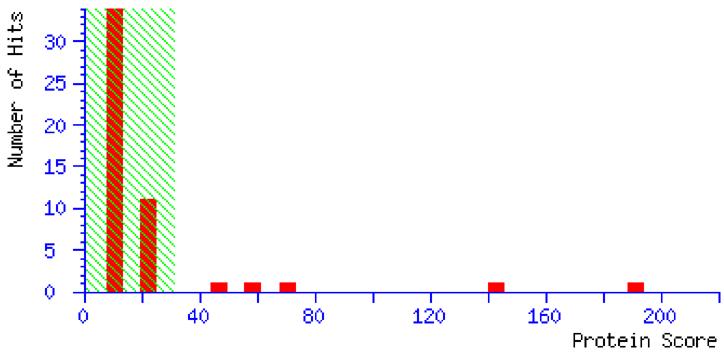

Matched peptide sequences: shown in **Bold Red**

```
  1 MAEEVILLDF WSSPFGMRVR IALAEKGVKY EYREEDLRNK SPLLLQMNPV
 51 HKKIPVLIHN GKPICESLIA VQYVDEVWKD KSPLLPSDPY QRAQARFWAD
101 FIDKKIYDIG RKIWTTKGDE QEAAKKEFIE ALKLLEGELG NKPYFGGESM
151 GYVDVALIPF YSWFYAYETC GNFSIEPECP VLIAWAKRCL QKESVSKSLP
201 DPQKVYEFVL VLKKKFGIE
```

Matched peptide information:

Start - End	Observed	Mr(expt)	Mr(calc)	ppm	Miss	Sequence
30 - 38	1272.6665	1271.6592	1271.5782	64	1	K.YEYREEDLR.N (Ions score 48)
41 - 52	1392.8492	1391.8419	1391.7595	59	0	K.SPLLLQMNPVHK.K Oxidation (M) (Ions score 47)
97 - 104	1041.5602	1040.5529	1040.4967	54	0	R.FWADFIDK.K (Ions score 21)
97 - 105	1169.6688	1168.6615	1168.5917	60	1	R.FWADFIDKK.I (Ions score 51)
205 - 213	1109.7219	1108.7146	1108.6532	55	0	K.VYEFVLVLK.K (Ions score 25)

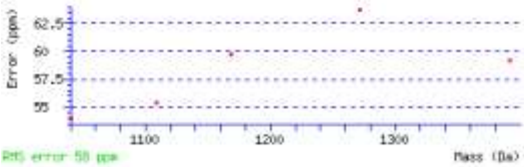

Spot No.: **29**

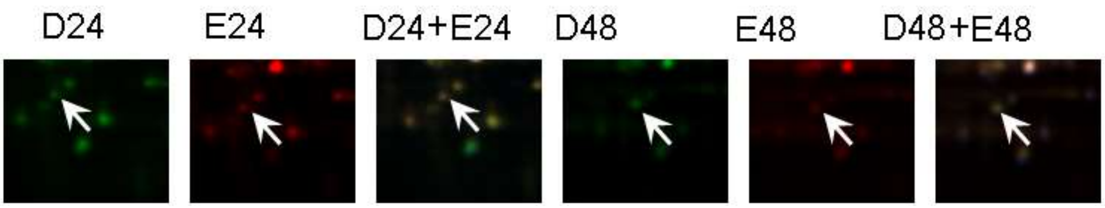

Accession No.: **scaffold0135_1578719.mRNA1**

Plant species: ***Hevea brasiliensis***

Protein name: **Probable glutathione S-transferase**

Peptide sequences: **K.YEYREEDLR.N; R.FWADFIDK.K; R.FWADFIDKK.I; K.VYEFVLVLK.K**

PFF Mascot score: **[185]** Sequence coverage %: **[12]**

Matched peptides No.: **[4]**

Calculated Mr: **25563** Calculated *p*I: **6.23**

Annotated PFF **spectra:**

Ions score is -10*Log(P), where P is the probability that the observed match is a random event.
Individual ions scores > 31 indicate identity or extensive homology (p<0.05).
Protein scores are derived from ions scores as a non-probabilistic basis for ranking protein hits.

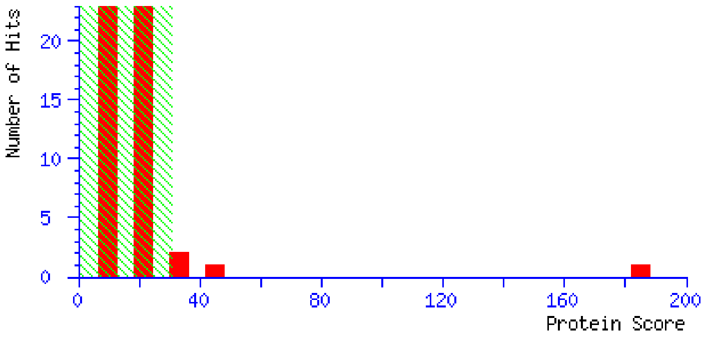

Matched peptide sequences: shown in **Bold Red**

```
  1 MAEEVILLDF WSSPFGMRVR IALAEKGVKY EYREEDLRNK SPLLLQMNPV
 51 HKKIPVLIHN GKPICESLIA VQYVDEVWKD KSPLLPSDPY QRAQARFWAD
101 FIDKKIYDIG RKIWTTKGDE QEAAKKEFIE ALKLLEGELG NKPYFGGESM
151 GYVDVALIPF YSWFYAYETC GNFSIEPECP VLIAWAKRCL QKESVSKSLP
201 DPQKVYEFVL VLKKKFGIE
```

Matched (bold red) residues: 1 … IALAEKGVK**Y** **EYREEDLR**NK …; 51 … QRAQAR**FWAD**; 101 **FIDKK**IYDIG …; 201 DPQK**VYEFVL** **VLK**KKFGIE

Matched peptide information:

Start - End	Observed	Mr(expt)	Mr(calc)	ppm	Miss	Sequence	
30 - 38	1272.6244	1271.6171	1271.5782	31	1	K.YEYREEDLR.N	(Ions score 28)
97 - 104	1041.4972	1040.4899	1040.4967	-7	0	R.FWADFIDK.K	(Ions score 61)
97 - 105	1169.5955	1168.5882	1168.5917	-3	1	R.FWADFIDKK.I	(Ions score 56)
205 - 213	1109.6362	1108.6289	1108.6532	-22	0	K.VYEFVLVLK.K	(Ions score 41)

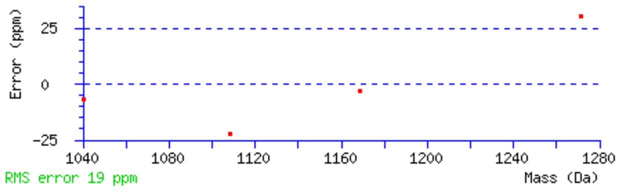

Spot No.: **30**

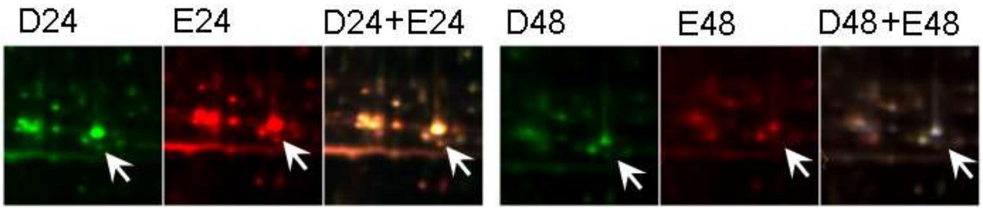

Accession No.: **scaffold0135_1578719.mRNA1**

Plant species: ***Hevea brasiliensis***

Protein name：**Probable glutathione S-transferase**

Peptide sequences: **K.YEYREEDLR.N；R.FWADFIDKK.I**

PFF Mascot score: **[110]** Sequence coverage %: **[8]**

Matched peptides No.: **[2]**

Calculated Mr: **25563** Calculated *p*I: **6.23**

Annotated PFF **spectra:**

Ions score is -10*Log(P), where P is the probability that the observed match is a random event.
Individual ions scores > 31 indicate identity or extensive homology (p<0.05).
Protein scores are derived from ions scores as a non-probabilistic basis for ranking protein hits.

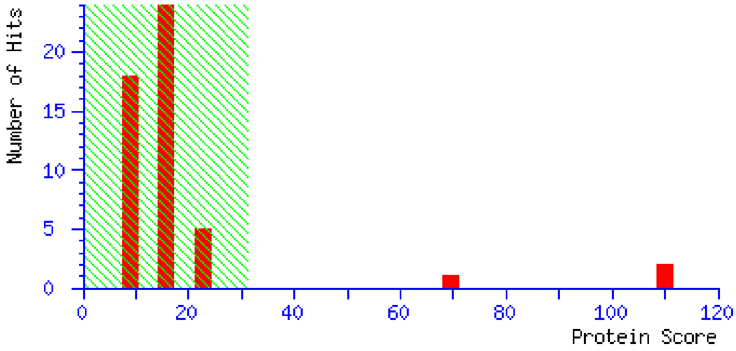

Matched peptide sequences: shown in Bold Red

```
  1 MAEEVILLDF WSSPFGMRVR IALAEKGVKY EYREEDLRNK SPLLLQMNPV
 51 HKKIPVLIHN GKPICESLIA VQYVDEVWKD KSPLLPSDPY QRAQARFWAD
101 FIDKKIYDIG RKIWTTKGDE QEAAKKEFIE ALKLLEGELG NKPYFGGESM
151 GYVDVALIPF YSWFYAYETC GNFSIEPECP VLIAWAKRCL QKESVSKSLP
201 DPQKVYEFVL VLKKKFGIE
```

(Shown in bold red: Y EYREEDLR (30–38); FWAD FIDKK (97–105))

Matched peptide information:

Start – End	Observed	Mr(expt)	Mr(calc)	ppm	Miss	Sequence	
30 – 38	1272.5231	1271.5158	1271.5782	-49	1	K.YEYREEDLR.N	(Ions score 50)
97 – 105	1169.5366	1168.5293	1168.5917	-53	1	R.FWADFIDKK.I	(Ions score 61)

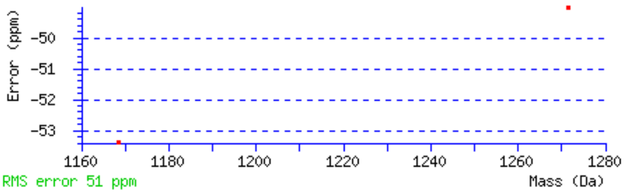

Spot No.: **31**

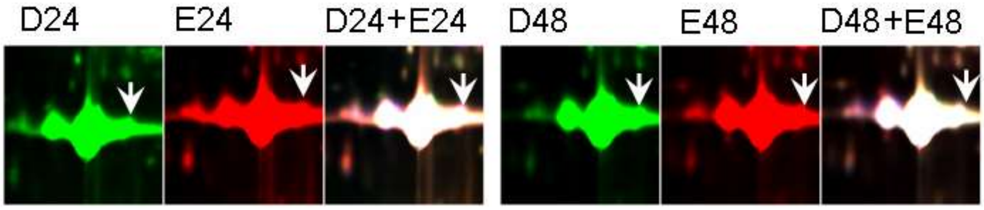

Accession No.: **scaffold1222_60641.mRNA1**

Plant species: ***Hevea brasiliensis***

Protein name：**Small rubber particle protein**

Peptide sequences: **K.DISGPLKPGVDTIENVVK.T；**
K.TVVTPVYYIPLEAVK.F ； K.FVDKTVDVSVTSLDGVVPPVIK.Q； K.QVSAQTYSVAQDAPR.I； R.IVLDVASSVFNTGVQEGAK.A； K.ALYANLEPK.A； K.AEQYAVITWR.A； R.ALNKLPLVPQVANVVVPTAVYFSEK.Y； K.LPLVPQVANVVVPTAVYFSEK.Y； K.LPLVPQVANVVVPTAVYFSEKYNDVVR.G； R.VSSYLPLLPTEK.I

PFF Mascot score: **[739]**

Sequence coverage %: **[74]**

Matched peptides No.: **[11]**

Calculated Mr: **22331**

Calculated *p*I: **4.80**

Annotated PFF **spectra:**

Ions score is -10*Log(P), where P is the probability that the observed match is a random event.
Individual ions scores > 31 indicate identity or extensive homology (p<0.05).
Protein scores are derived from ions scores as a non-probabilistic basis for ranking protein hits.

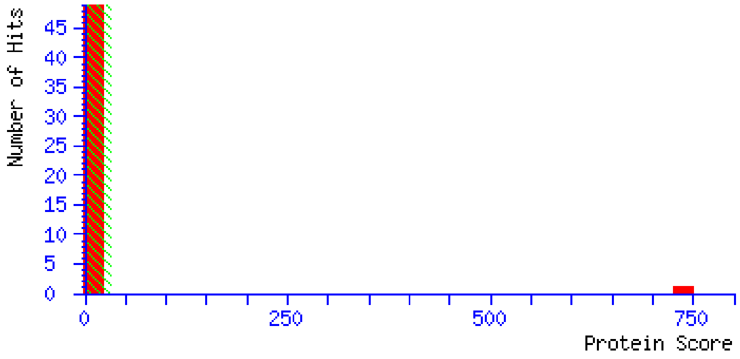

Matched peptide sequences: shown in **Bold Red**

```
  1 MAEEVEEERL KYLDFVRAAG VYAVDSFSTL YLYAKDISGP LKPGVDTIEN
 51 VVKTVVTPVY YIPLEAVKFV DKTVDVSVTS LDGVVPPVIK QVSAQTYSVA
101 QDAPRIVLDV ASSVFNTGVQ EGAKALYANL EPKAEQYAVI TWRALNKLPL
151 VPQVANVVVP TAVYFSEKYN DVVRGTTEQG YRVSSYLPLL PTEKITKVFG
201 DEAS
```

Matched peptide information:

Start - End	Observed	Mr(expt)	Mr(calc)	ppm	Miss	Sequence
36 - 53	1881.0472	1880.0399	1880.0255	8	1	K.DISGPLKPGVDTIENVVK.T (Ions score 115)
54 - 68	1691.9594	1690.9521	1690.9546	-1	0	K.TVVTPVYYIPLEAVK.F (Ions score 68)
69 - 90	2314.2991	2313.2918	2313.2832	4	1	K.FVDKTVDVSVTSLDGVVPPVIK.Q (Ions score 18)
91 - 105	1620.8217	1619.8144	1619.7903	15	0	K.QVSAQTYSVAQDAPR.I (Ions score 142)
106 - 124	1934.0342	1933.0269	1933.0157	6	0	R.IVLDVASSVFNTGVQEGAK.A (Ions score 112)
125 - 133	1018.5529	1017.5456	1017.5495	-4	0	K.ALYANLEPK.A (Ions score 49)
134 - 143	1236.6542	1235.6469	1235.6299	14	0	K.AEQYAVITWR.A (Ions score 88)
144 - 168	2696.5498	2695.5425	2695.5313	4	1	R.ALNKLPLVPQVANVVVPTAVYFSEK.Y (Ions score 84)
148 - 168	2270.2610	2269.2537	2269.2722	-8	0	K.LPLVPQVANVVVPTAVYFSEK.Y (Ions score 16)
148 - 174	3016.6633	3015.6560	3015.6434	4	1	K.LPLVPQVANVVVPTAVYFSEKYNDVVR.G (Ions score 17)
183 - 194	1346.7416	1345.7343	1345.7493	-11	0	R.VSSYLPLLPTEK.I (Ions score 29)

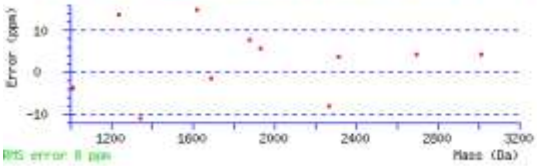

Spot No.: 32

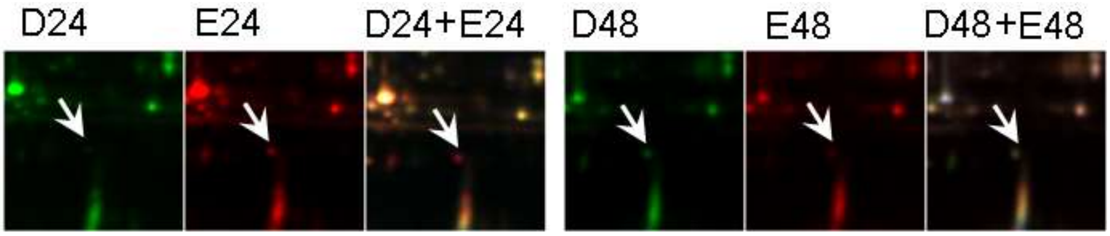

Accession No.: **scaffold1222_100110.mRNA1**

Plant species: ***Hevea brasiliensis***

Protein name：**Rubber elongation factor protein**

Peptide sequences: **K.SGPFKPGVNTVESR.F；R.RVDAYVTVLDR.I**

PFF Mascot score: **[112]** Sequence coverage %: **[14]**

Matched peptides No.: **[2]**

Calculated Mr: **19612** Calculated *p*I: **5.28**

Annotated PFF **spectra:**

Ions score is -10*Log(P), where P is the probability that the observed match is a random event.
Individual ions scores > 30 indicate identity or extensive homology (p<0.05).
Protein scores are derived from ions scores as a non-probabilistic basis for ranking protein hits.

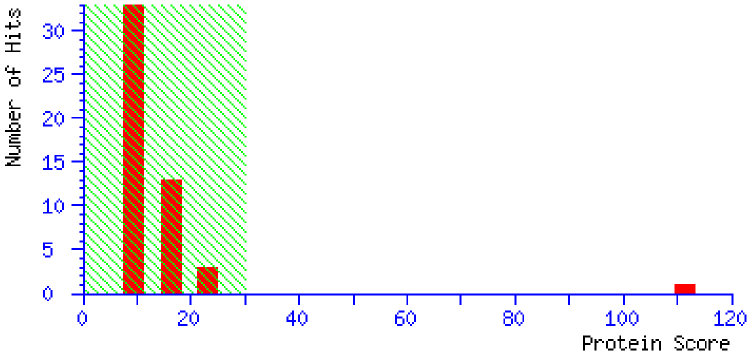

Matched peptide sequences: shown in **Bold Red**

```
  1 MAEGEEEVNI QEEANKGEEN PQEEANIQEE TNKGEENIQE EANIQEEANK
 51 EEESLKYLDF VQAATVYARA SFSKLYLFAK DKSGPFKPGV NTVESRFKSV
101 VRPVYNKFQP VPNKVLKFAD RRVDAYVTVL DRIVPPIVKR ASIQAYSVAP
151 GAARAVASYL PLHTKRLSKV LYGDG
```

Matched peptide information:

Start - End	Observed	Mr(expt)	Mr(calc)	ppm	Miss	Sequence
83 - 96	1474.6901	1473.6828	1473.7576	-51	1	K.SGPFKPGVNTVESR.F (Ions score 57)
122 - 132	1306.6473	1305.6400	1305.7041	-49	1	R.RVDAYVTVLDR.I (Ions score 55)

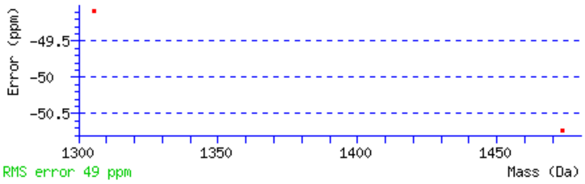

Spot No.: 33

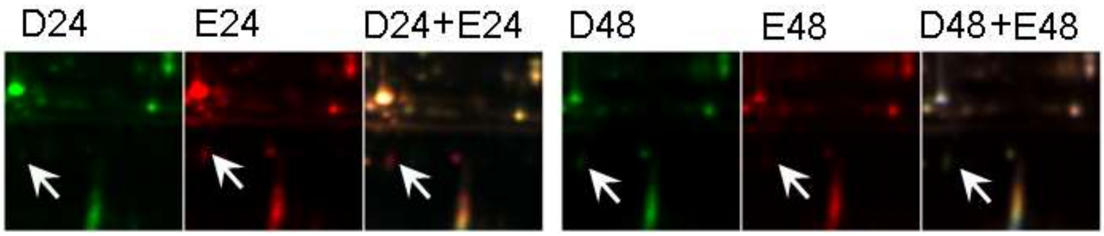

Accession No.: **scaffold0959_355995.mRNA1**

Plant species: ***Hevea brasiliensis***

Protein name：**Probable glutathione peroxidase 2**

Peptide sequences: **K.AEFPIFDK.I; K.GGLFVDAIK.W**

PFF Mascot score: **[111]** Sequence coverage %: **[8]**

Matched peptides No.: **[2]**

Calculated Mr: **22791** Calculated *p*I: **8.29**

Annotated PFF **spectra:**

Ions score is -10*Log(P), where P is the probability that the observed match is a random event.
Individual ions scores > 31 indicate identity or extensive homology (p<0.05).
Protein scores are derived from ions scores as a non-probabilistic basis for ranking protein hits.

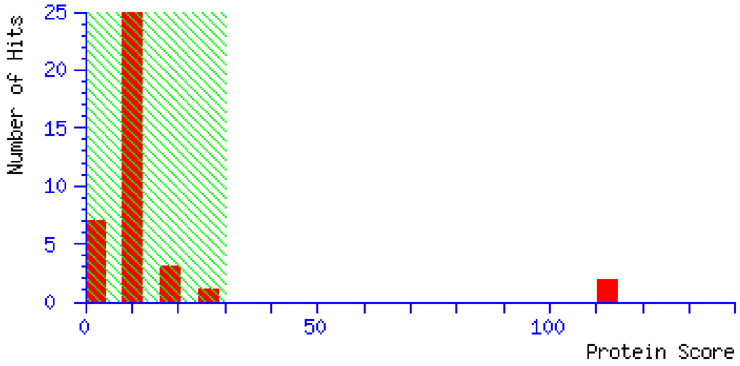

Matched peptide sequences: shown in Bold Red

```
  1 MHFTNWVSLA FLVFAVLLFY SYQTPLFFSS RNMAQESPKS IYDFTVKDIQ
 51 GNDVSLSKYS GKVLLIVNVA SKCGFTQSNY KELNVLYEKY KNQGFEILAF
101 PCNQFAGQEP GSNEEIQEVA CTMFKAEFPI FDKIEVNGKN TAPLYKYLKS
151 EKGGLFVDAI KWNFTKFLVN KEGKVVERYA PTTSPLKIEK DIQNLLGSS
```

(Bold red: residues 126–133 AEFPIFDK and 153–161 GGLFVDAIK)

Matched peptide information:

Start - End	Observed	Mr(expt)	Mr(calc)	ppm	Miss	Sequence	
126 - 133	966.5029	965.4956	965.4858	10	0	K.AEFPIFDK.I	(Ions score 55)
153 - 161	919.5311	918.5239	918.5175	7	0	K.GGLFVDAIK.W	(Ions score 57)

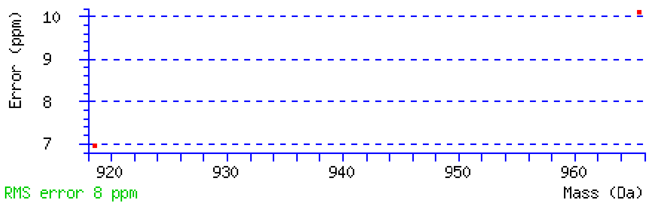

Spot No.: **34**

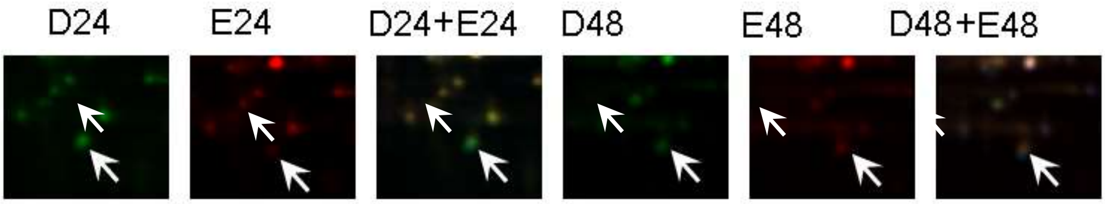

Accession No.: **scaffold0427_434785.mRNA1**

Plant species: ***Hevea brasiliensis***

Protein name： **Superoxide dismutase [Mn], mitochondrial**

Peptide sequences: **K.HHQTYVTNFNTALEQLNDAMEKGDPAAVVK.L；R.EGGGEPPHSSLGWAIDTDFGSLEK.L；K.INAEGAALQGSGWVWLALDK.E；K.KLVVETTANQDPLVTK.G；K.NVRPDYLK.N；K.YASEVYAK.E**

PFF Mascot score: **[356]**

Sequence coverage %: **[45]**

Matched peptides No.: **[6]**

Calculated Mr: **26153**

Calculated *p*I: **7.82**

Annotated PFF **spectra:**

Ions score is -10*Log(P), where P is the probability that the observed match is a random event.
Individual ions scores > 30 indicate identity or extensive homology (p<0.05).
Protein scores are derived from ions scores as a non-probabilistic basis for ranking protein hits.

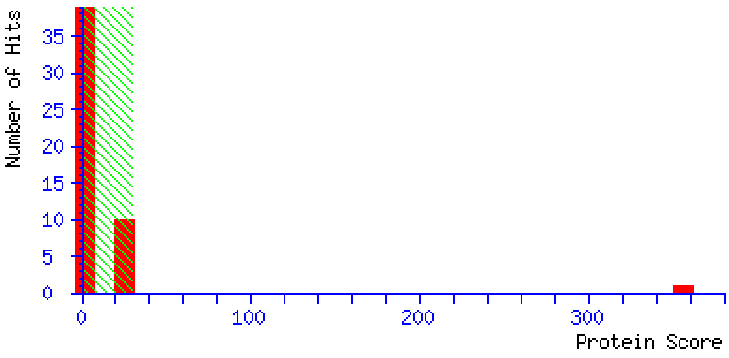

Matched peptide sequences: shown in **Bold Red**

```
  1 MALRSLVARK TISSAFKAAT GLRLGQLRGI QTYSLPDLPY DYGALEPVIS
 51 GEIMQLHHQK HHQTYVTNFN TALEQLNDAM EKGDPAAVVK LQSAIKFNGG
101 GHVNHSIFWK NLAPVREGGG EPPHSSLGWA IDTDFGSLEK LIQKINAEGA
151 ALQGSGWVWL ALDKELKKLV VETTANQDPL VTKGPSLIPL LGIDVWEHAY
201 YLQYKNVRPD YLKNIWKVMN WKYASEVYAK ECPSS
```

Matched peptide information:

Start – End	Observed	Mr(expt)	Mr(calc)	ppm	Miss	Sequence
61 – 90	3342.5107	3341.5034	3340.6146	266	1	K.HHQTYVTNFNTALEQLNDAMEKGDPAAVVK.L (Ions score 3)
117 – 140	2486.0547	2485.0474	2485.1398	-37	0	R.EGGGEPPHSSLGWAIDTDFGSLEK.L (Ions score 114)
145 – 164	2099.0100	2098.0027	2098.0847	-39	0	K.INAEGAALQGSGWVWLALDK.E (Ions score 87)
168 – 183	1755.8998	1754.8925	1754.9778	-49	1	K.KLVVETTANQDPLVTK.G (Ions score 62)
206 – 213	1004.5160	1003.5087	1003.5451	-36	1	K.NVRPDYLK.N (Ions score 48)
223 – 230	930.4247	929.4175	929.4494	-34	0	K.YASEVYAK.E (Ions score 42)

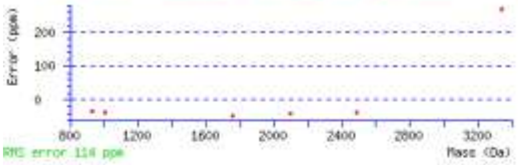

Spot No.: 35

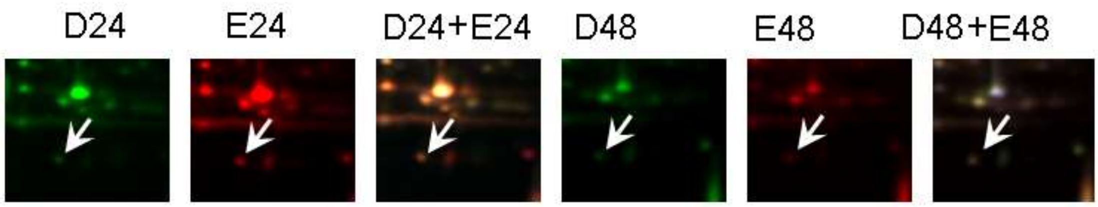

Accession No.: **scaffold1222_100110.mRNA1**

Plant species: ***Hevea brasiliensis***

Protein name：**Rubber elongation factor protein**

Peptide sequences: **K.YLDFVQAATVYAR.A；K.SGPFKPGVNTVESR.F；K.SVVRPVYNK.F；R.RVDAYVTVLDR.I；R.VDAYVTVLDR.I；R.ASIQAYSVAPGAAR.A**

PFF Mascot score: **[507]** Sequence coverage %: **[34]**

Matched peptides No.: **[6]**

Calculated Mr: **19612** Calculated *p*I: **5.28**

Annotated PFF **spectra:**

Ions score is -10*Log(P), where P is the probability that the observed match is a random event.
Individual ions scores > 31 indicate identity or extensive homology (p<0.05).
Protein scores are derived from ions scores as a non-probabilistic basis for ranking protein hits.

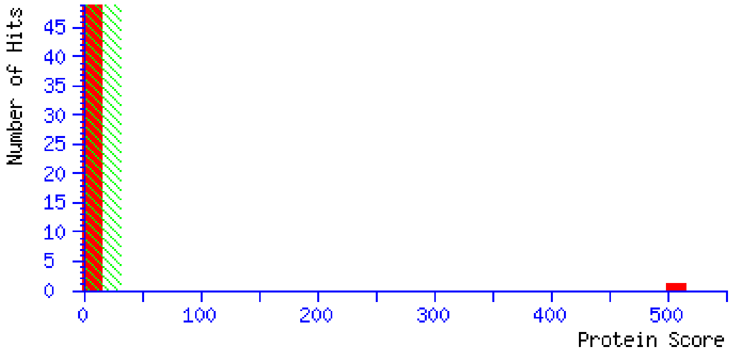

Matched peptide sequences: shown in Bold Red

1 MAEGEEEVNI QEEANKGEEN PQEEANIQEE TNKGEENIQE EANIQEEANK
51 EEESLK**YLDF** **VQAATVYAR**A SFSKLYLFAK DK**SGPFKPGV** **NTVESR**FK**SV**
101 **VRPVYNK**FQP VPNKVLKFAD R**RVDAYVTVL** **DR**IVPPIVKR **ASIQAYSVAP**
151 **GAAR**AVASYL PLHTKRLSKV LYGDG

Matched peptide information:

Start - End	Observed	Mr(expt)	Mr(calc)	ppm	Miss	Sequence
57 - 69	1516.7640	1515.7567	1515.7722	-10	0	K.YLDFVQAATVYAR.A (Ions score 109)
83 - 96	1474.7479	1473.7406	1473.7576	-12	1	K.SGPFKPGVNTVESR.F (Ions score 113)
99 - 107	1061.5902	1060.5829	1060.6029	-19	1	K.SVVRPVYNK.F (Ions score 28)
122 - 132	1306.6814	1305.6741	1305.7041	-23	1	R.RVDAYVTVLDR.I (Ions score 72)
123 - 132	1150.5931	1149.5858	1149.6030	-15	0	R.VDAYVTVLDR.I (Ions score 87)
141 - 154	1361.6993	1360.6920	1360.7099	-13	0	R.ASIQAYSVAPGAAR.A (Ions score 99)

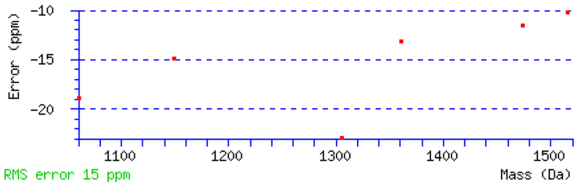

Spot No.: **36**

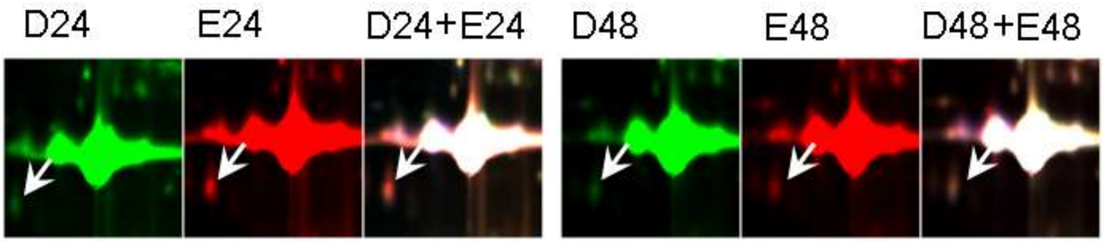

Accession No.: **scaffold0625_591792.mRNA1**

Plant species: ***Hevea brasiliensis***

Protein name：**Translationally-controlled tumor protein homolog**

Peptide sequences: **K.EIHNGILWEVEGK.W；R.EGATDPTFLYFAYALK.E；K.WVVQGAVDVDIGANPSAEGADEDEGVDDQAVK.V；K.VVDIVDTFR.L；R.LQEQPAFDKK.Q；K.LSDLQFFVGESMHDDGSLVFAYYR.E；**

PFF Mascot score: **[376]** Sequence coverage %: **[61]**

Matched peptides No.: **[6]**

Calculated Mr: **19156** Calculated *p*I: **4.47**

Annotated PFF **spectra:**

Ions score is -10*Log(P), where P is the probability that the observed match is a random event.
Individual ions scores > 31 indicate identity or extensive homology (p<0.05).
Protein scores are derived from ions scores as a non-probabilistic basis for ranking protein hits.

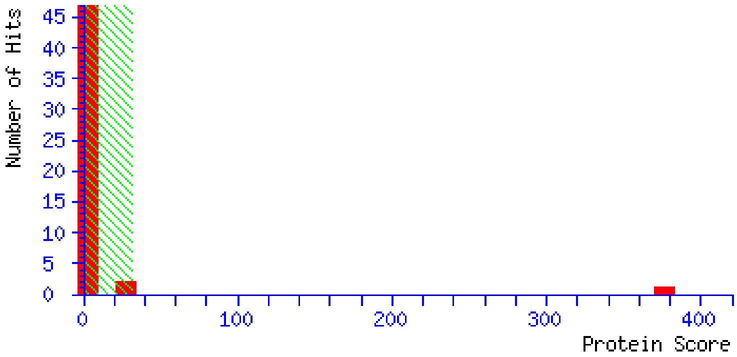

Matched peptide sequences: shown in **Bold Red**

```
  1 MLVYQDLLTG DELLSDSFPY KEIHNGILWE VEGKWVVQGA VDVDIGANPS
 51 AEGADEDEGV DDQAVKVVDI VDTFRLQEQP AFDKKQFVTY MKRFIKLLTP
101 KLDEEKQESF KKNIEGATKF LLSKLSDLQF FVGESMHDDG SLVFAYYREG
151 ATDPTFLYFA YALKEVKC
```

Matched peptide information:

Start	-	End	Observed	Mr(expt)	Mr(calc)	ppm	Miss	Sequence
22	-	34	1523.7277	1522.7204	1522.7780	-38	0	K.EIHNGILWEVEGK.W (Ions score 38)
35	-	66	3255.3733	3254.3660	3254.4852	-37	0	K.WVVQGAVDVDIGANPSAEGADEDEGVDDQAVK.V (Ions score 51)
67	-	75	1063.5427	1062.5354	1062.5710	-33	0	K.VVDIVDTFR.L (Ions score 68)
76	-	85	1203.5939	1202.5866	1202.6295	-36	1	R.LQEQPAFDKK.Q (Ions score 36)
125	-	148	2796.1936	2795.1863	2795.2901	-37	0	K.LSDLQFFVGESMHDDGSLVFAYYR.E (Ions score 121)
149	-	164	1806.8230	1805.8157	1805.8876	-40	0	R.EGATDPTFLYFAYALK.E (Ions score 62)

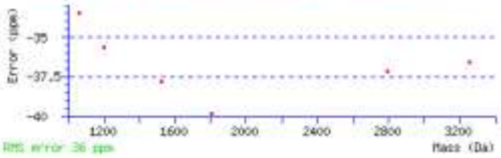

Spot No.: **37**

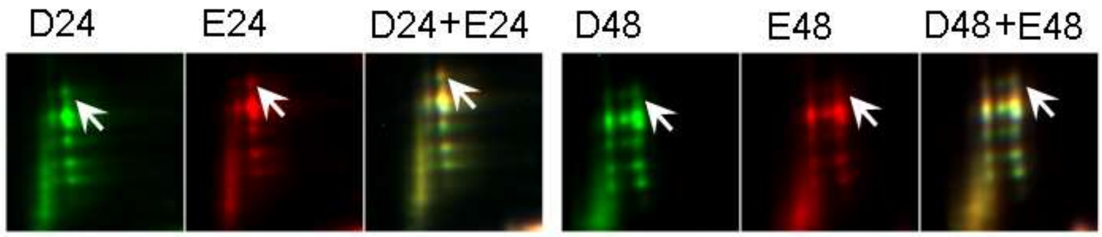

Accession No.: **scaffold3444_7763.mRNA1**

Plant species: ***Hevea brasiliensis***

Protein name: **Elicitor-responsive protein 3**

Peptide sequences: **M.PLGTVEVLLVGAK.G; K.GLENTDFLNGVDPYVVLACR.T; K.FSFEVSDGDTELTLK.I**

PFF Mascot score: **[190]**

Sequence coverage %: **[34]**

Matched peptides No.: **[3]**

Calculated Mr: **15249**

Calculated *p*I: **4.06**

Annotated PFF **spectra:**

Ions score is -10*Log(P), where P is the probability that the observed match is a random event.
Individual ions scores > 31 indicate identity or extensive homology (p<0.05).
Protein scores are derived from ions scores as a non-probabilistic basis for ranking protein hits.

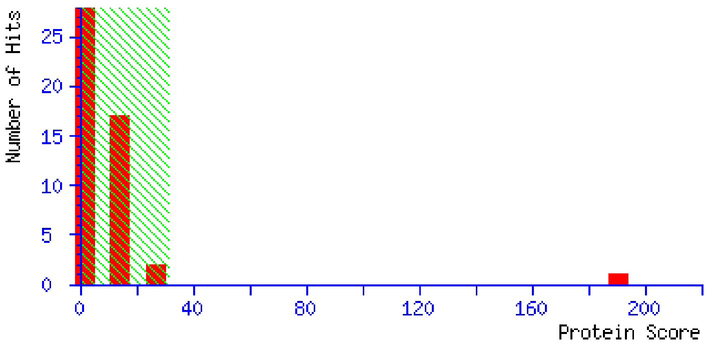

Matched peptide sequences: shown in **Bold Red**

```
  1 MPLGTVEVLL VGAKGLENTD FLNGVDPYVV LACRTQEQKS SVASGKGSEP
 51 EWNEKFSFEV SDGDTELTLK IMDSDVGAAD DFVGEATIPL EPLFLEGNLP
101 STAYKVVKEQ EYKGEITVGL TFTPEVEMDN VGVDGYDFRL
```

Matched: M**PLGTVEVLL VGAKGLENTD FLNGVDPYVV LACR** (2–34); EWNEK**FSFEV SDGDTELTLK** (56–70)

Matched peptide information:

Start - End	Observed	Mr(expt)	Mr(calc)	ppm	Miss	Sequence
2 - 14	1295.7573	1294.7500	1294.7860	-28	0	M.PLGTVEVLLVGAK.G (Ions score 45)
15 - 34	2252.1482	2251.1409	2251.0943	21	0	K.GLENTDFLNGVDPYVVLACR.T (Ions score 74)
56 - 70	1687.7842	1686.7769	1686.7989	-13	0	K.FSFEVSDGDTELTLK.I (Ions score 72)

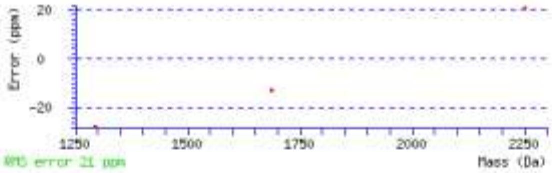

Spot No.: 38

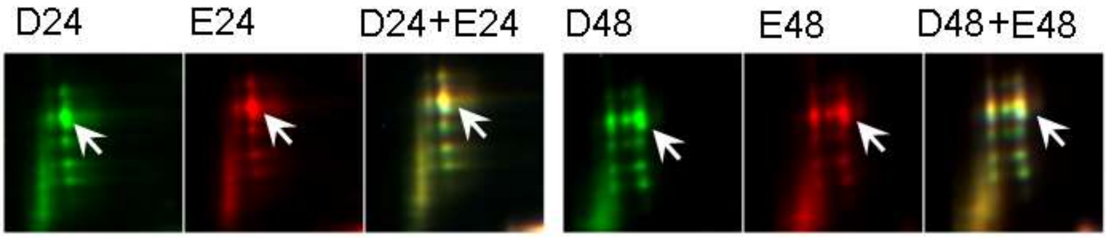

Accession No.: **scaffold3444_7763.mRNA1**

Plant species: ***Hevea brasiliensis***

Protein name： **Elicitor-responsive protein 3**

Peptide sequences: **M.PLGTVEVLLVGAK.G；K.GLENTDFLNGVDPYVVLACR.T；K.FSFEVSDGDTELTLK.I；K.GEITVGLTFTPEVEMDNVGVDGYDFR.L**

PFF Mascot score: **[446]** Sequence coverage %: **[52]**

Matched peptides No.: **[4]**

Calculated Mr: **15249** Calculated *p*I: **4.06**

Annotated PFF **spectra:**

Ions score is -10*Log(P), where P is the probability that the observed match is a random event.
Individual ions scores > 31 indicate identity or extensive homology (p<0.05).
Protein scores are derived from ions scores as a non-probabilistic basis for ranking protein hits.

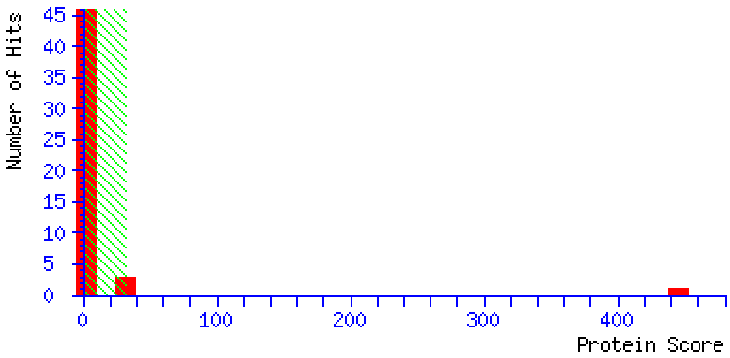

Matched peptide sequences: shown in **Bold Red**

```
  1 MPLGTVEVLL VGAKGLENTD FLNGVDPYVV LACRTQEQKS SVASGKGSEP
 51 EWNEKFSFEV SDGDTELTLK IMDSDVGAAD DFVGEATIPL EPLFLEGNLP
101 STAYKVVKEQ EYKGEITVGL TFTPEVEMDN VGVDGYDFRL
```

Matched peptide information:

Start - End	Observed	Mr(expt)	Mr(calc)	ppm	Miss	Sequence
2 - 14	1295.8602	1294.8529	1294.7860	52	0	M.PLGTVEVLLVGAK.G (Ions score 103)
15 - 34	2252.2053	2251.1980	2251.0943	46	0	K.GLENTDFLNGVDPYVVLACR.T (Ions score 56)
56 - 70	1687.8864	1686.8791	1686.7989	48	0	K.FSFEVSDGDTELTLK.I (Ions score 160)
114 - 139	2876.4487	2875.4414	2875.3223	41	0	K.GEITVGLTFTPEVEMDNVGVDGYDFR.L Oxidation (M) (Ions score 97)

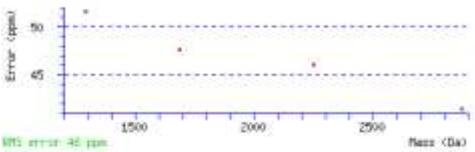

Spot No.: **39**

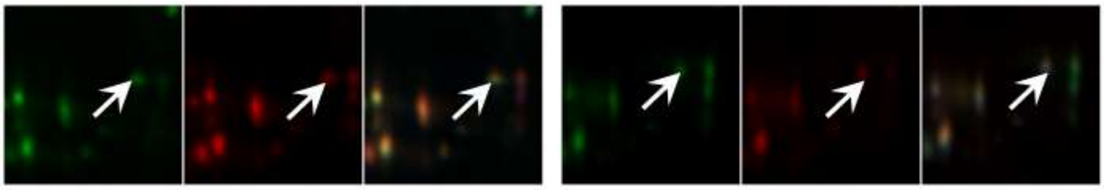

Accession No.: **scaffold0155_515853.mRNA1**

Plant species: ***Hevea brasiliensis***

Protein name： **Pro-hevein**

Peptide sequences: **K.YGWTAFCGPVGAHGQPSCGK.C; R.IVDQCSNGGLDLDVNVFR.Q; R.QLDTDGKGYER.G**

PFF Mascot score: **[190]** Sequence coverage %: **[23]**

Matched peptides No.: **[3]**

Calculated Mr: **23042** Calculated *p*I: **8.15**

Annotated PFF **spectra:**

Ions score is -10*Log(P), where P is the probability that the observed match is a random event.
Individual ions scores > 30 indicate identity or extensive homology (p<0.05).
Protein scores are derived from ions scores as a non-probabilistic basis for ranking protein hits.

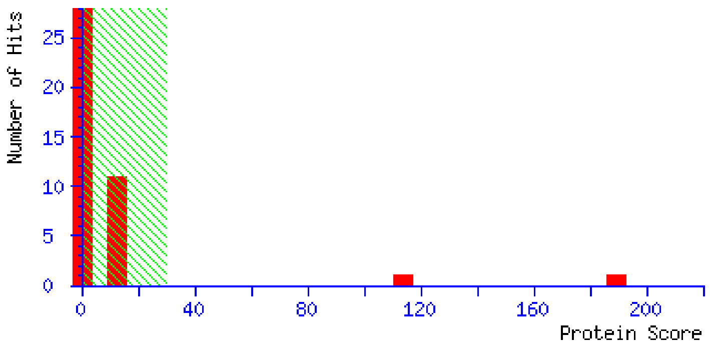

Matched peptide sequences: shown in Bold Red

```
  1 MGRVMNIFIV VLLCLTGVAI AEQCGRQAGG KLCPNNLCCS QYGWCGSSDD
 51 YCSPSKNCQS NCKGGGGGGG GGGGSASNVL ATYHLYNPQQ HGWDLNAVSA
101 YCSTWDANKP YSWRSKYGWT AFCGPVGAHG QPSCGKCLSV TNTGTGAKTT
151 VRIVDQCSNG GLDLDVNVFR QLDTDGKGYE RGHLTVNYQF VNCGDSFNPL
201 FSIMKSSVIN
```

(Bold red in original: 117–136 YGWT AFCGPVGAHG QPSCGK; 153–181 IVDQCSNG GLDLDVNVFR QLDTDGKGYE R)

Matched peptide information:

Start - End	Observed	Mr(expt)	Mr(calc)	ppm	Miss	Sequence	
117 - 136	2136.8660	2135.8587	2135.9306	-34	0	K.YGWTAFCGPVGAHGQPSCGK.C	(Ions score 58)
153 - 170	2020.9030	2019.8957	2019.9684	-36	0	R.IVDQCSNGGLDLDVNVFR.Q	(Ions score 97)
171 - 181	1281.5587	1280.5514	1280.5997	-38	1	R.QLDTDGKGYER.G	(Ions score 35)

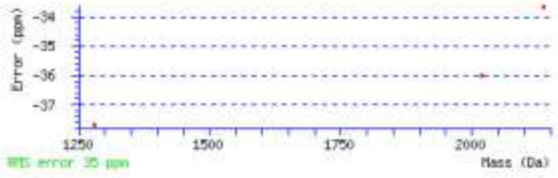

Spot No.: **40**

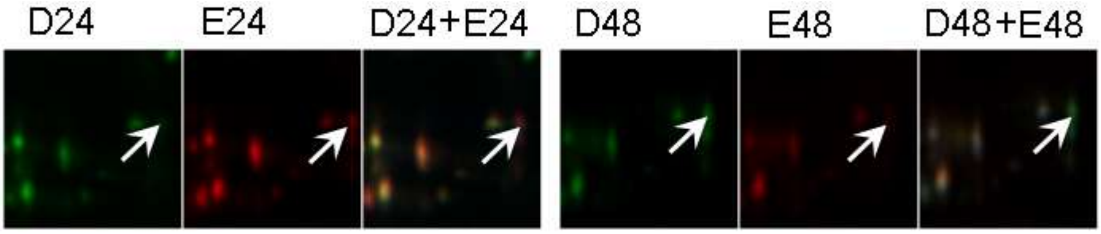

Accession No.: **scaffold0645_687748.mRNA1**

Plant species: ***Hevea brasiliensis***

Protein name： **18.5 kDa class I heat shock protein**

Peptide sequences: **R.FEFANETSAFANTR.I；**

K.EEVKVEIEEGNVLQISGER.S； **K.VEIEEGNVLQISGER.S；** **R.FRLPENAK.V**

PFF Mascot score: **[397]** Sequence coverage %: **[25]**

Matched peptides No.: **[4]**

Calculated Mr: **18335** Calculated *p*I: **6.00**

Annotated PFF **spectra:**

Ions score is -10*Log(P), where P is the probability that the observed match is a random event.
Individual ions scores > 30 indicate identity or extensive homology ($p<0.05$).
Protein scores are derived from ions scores as a non-probabilistic basis for ranking protein hits.

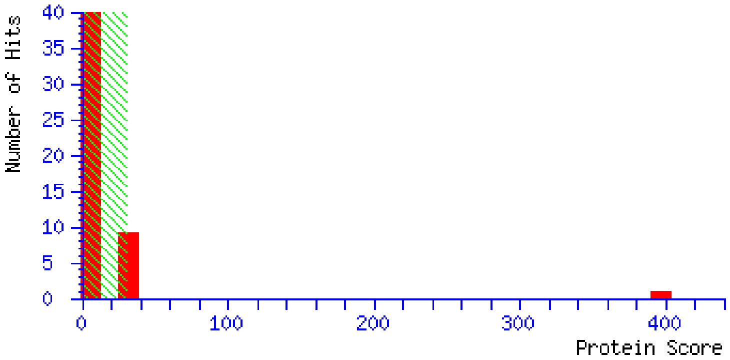

Matched peptide sequences: shown in **Bold Red**

```
  1 MSLIPSSLFG GRRTNIFDPF SLDVWDPFHD FPFPSTALSA PRFEFANETS
 51 AFANTRIDWK ETPEAHVFKA DLPGLKKEEV KVEIEEGNVL QISGERSKEK
101 EEKNDKLHRV ERSSGKFLRR FRLPENAKVD QVKASMESGV LTVTVPKEEV
151 KKPDVKAIDI SG
```

Matched peptide information:

Start - End	Observed	Mr(expt)	Mr(calc)	ppm	Miss	Sequence	
43 - 56	1604.7509	1603.7436	1603.7267	11	0	R.FEFANETSAFANTR.I	(Ions score 116)
78 - 96	2157.1218	2156.1145	2156.0961	9	1	K.EEVKVEIEEGNVLQISGER.S	(Ions score 104)
82 - 96	1671.8698	1670.8625	1670.8475	9	0	K.VEIEEGNVLQISGER.S	(Ions score 137)
121 - 128	974.5561	973.5488	973.5345	15	1	R.FRLPENAK.V	(Ions score 40)

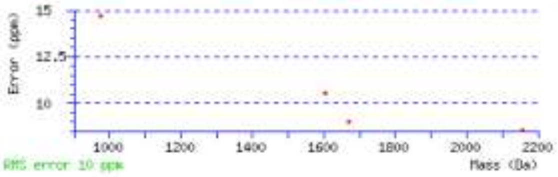

Spot No.: **41**

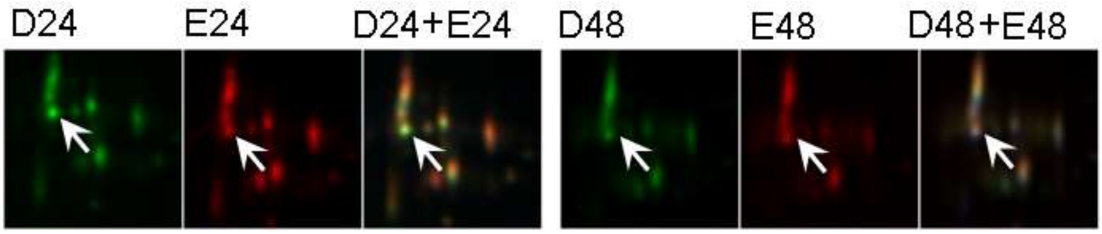

Accession No.: **scaffold0155_515853.mRNA1**

Plant species: ***Hevea brasiliensis***

Protein name: **Pro-hevein**

Peptide sequences: **K.YGWTAFCGPVGAHGQPSCGK.C; R.IVDQCSNGGLDLDVNVFR.Q; R.QLDTDGKGYER.G**

PFF Mascot score: **[132]**

Sequence coverage %: **[23]**

Matched peptides No.: **[3]**

Calculated Mr: **23042**

Calculated pI: **8.15**

Annotated PFF spectra:

Ions score is -10*Log(P), where P is the probability that the observed match is a random event.
Individual ions scores > 31 indicate identity or extensive homology (p<0.05).
Protein scores are derived from ions scores as a non-probabilistic basis for ranking protein hits.

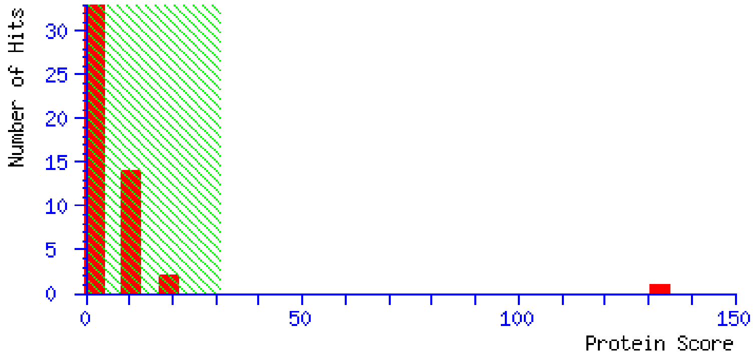

Matched peptide sequences: shown in Bold Red

```
  1 MGRVMNIFIV VLLCLTGVAI AEQCGRQAGG KLCPNNLCCS QYGWCGSSDD
 51 YCSPSKNCQS NCKGGGGGGG GGGGSASNVL ATYHLYNPQQ HGWDLNAVSA
101 YCSTWDANKP YSWRSKYGWT AFCGPVGAHG QPSCGKCLSV TNTGTGAKTT
151 VRIVDQCSNG GLDLDVNVFR QLDTDGKGYE RGHLTVNYQF VNCGDSFNPL
201 FSIMKSSVIN
```

Bold red residues: 117–136 (YGWT AFCGPVGAHG QPSCGK) and 153–181 (IVDQCSNG GLDLDVNVFR QLDTDGKGYE R).

Matched peptide information:

Start - End	Observed	Mr(expt)	Mr(calc)	ppm	Miss	Sequence
117 - 136	2137.0818	2136.0745	2135.9306	67	0	K.YGWTAFCGPVGAHGQPSCGK.C (Ions score 3)
153 - 170	2021.1167	2020.1094	2019.9684	70	0	R.IVDQCSNGGLDLDVNVFR.Q (Ions score 123)
171 - 181	1281.6882	1280.6809	1280.5997	63	1	R.QLDTDGKGYER.G (Ions score 6)

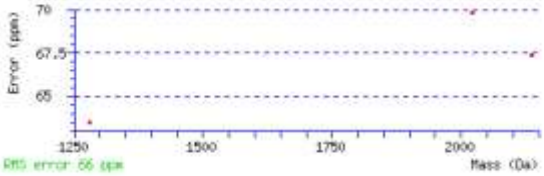

Spot No.: **42**

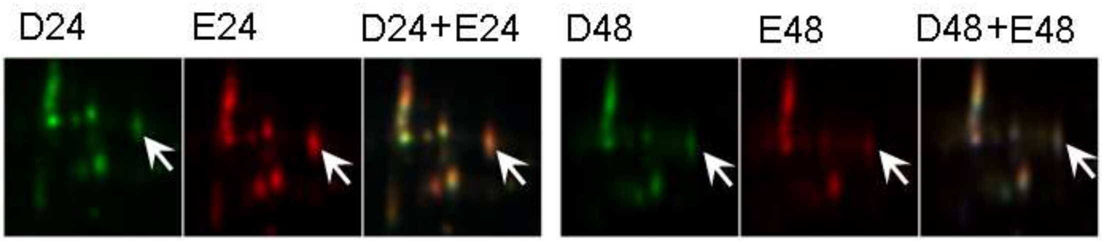

Accession No.: **scaffold0872_395492.mRNA1**

Plant species: ***Hevea brasiliensis***

Protein name：**Eukaryotic translation initiation factor 5A**

Peptide sequences: **K.TYPQQAGTIR.K；K.CHFVGIDIFNAK.K；K.KLEDIVPSSHNCDVPHVTR.T；R.TDYQLIDISEDGFVSLLTENGNTK.D；K.DDLRLPTDENLLSQIK.D；R.LPTDENLLSQIK.D；K.DLVVTVMSSMGEEQICALK.D**

PFF Mascot score: **[483]**

Sequence coverage %: **[62]**

Matched peptides No.: **[7]**

Calculated Mr: **17689**

Calculated *p*I: **5.60**

Annotated PFF spectra:

Ions score is -10*Log(P), where P is the probability that the observed match is a random event.
Individual ions scores > 30 indicate identity or extensive homology (p<0.05).
Protein scores are derived from ions scores as a non-probabilistic basis for ranking protein hits.

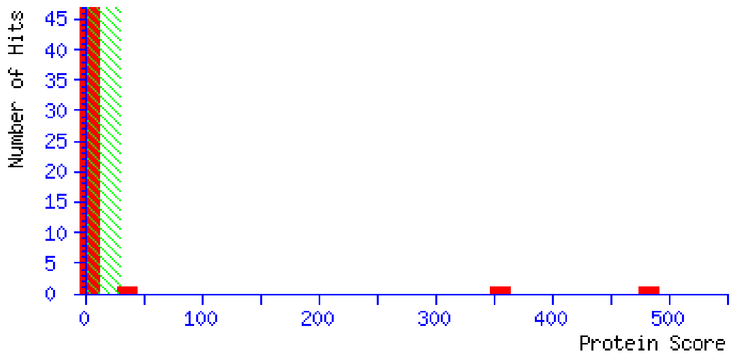

Matched peptide sequences: shown in Bold Red

1 MSDEEHHFES KADAGASK**TY PQQAGTIR**KN GYIVIKNRPC KVVDVSTSKT
51 GKHGHAK**CHF VGIDIFNAKK LEDIVPSSHN CDVPHVTRTD YQLIDISEDG**
101 **FVSLLTENGN TKDDLRLPTD ENLLSQIK**DG FAEGK**DLVVT VMSSMGEEQI**
151 **CALK**DIGPK

Matched peptide information:

Start - End	Observed	Mr(expt)	Mr(calc)	ppm	Miss	Sequence
19 - 28	1134.6040	1133.5967	1133.5829	12	0	K.TYPQQAGTIR.K (Ions score 63)
58 - 69	1420.7086	1419.7013	1419.6969	3	0	K.CHFVGIDIFNAK.K (Ions score 74)
70 - 88	2203.1091	2202.1018	2202.0852	8	1	K.KLEDIVPSSHNCDVPHVTR.T (Ions score 121)
89 - 112	2672.3103	2671.3030	2671.2865	6	0	R.TDYQLIDISEDGFVSLLTENGNTK.D (Ions score 65)
113 - 128	1870.0052	1868.9979	1868.9843	7	1	K.DDLRLPTDENLLSQIK.D (Ions score 78)
117 - 128	1370.7583	1369.7510	1369.7453	4	0	R.LPTDENLLSQIK.D (Ions score 37)
136 - 154	2110.0283	2109.0210	2109.0156	3	0	K.DLVVTVMSSMGEEQICALK.D (Ions score 46)

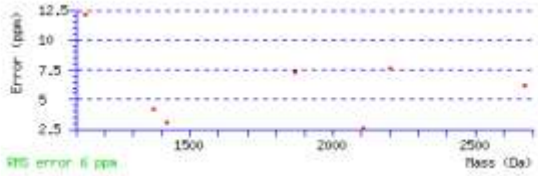

Spot No.: **43**

D24 E24 D24+E24 D48 E48 D48+E48

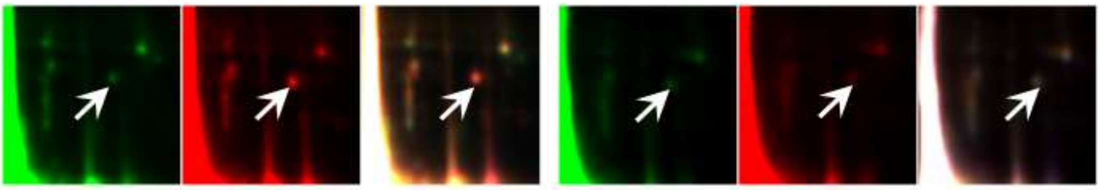

Accession No.: **scaffold1222_181260.mRNA1**

Plant species: ***Hevea brasiliensis***

Protein name： **Small rubber particle protein**

Peptide sequences: **K.EVDVEEER.L；K.YLDFVQTGAVFALVSFSK.L；K.DVSGPFKPYVENAGGR.F；K.SVVRPIYYK.F；K.FQPVSNEILK.F；K.FADHKVDESVTILDLFVPPIVK.Q；K.VDESVTILDLFVPPIVK.Q；K.QLCTQAYSVAR.D；R.DAPVVACALTYYLLSPNEK.F**

PFF Mascot score: **[868]** Sequence coverage %: **[73]**

Matched peptides No.: **[9]**

Calculated Mr: **17567** Calculated *p*I: **4.98**

Annotated PFF **spectra:**

Ions score is -10*Log(P), where P is the probability that the observed match is a random event.
Individual ions scores > 31 indicate identity or extensive homology (p<0.05).
Protein scores are derived from ions scores as a non-probabilistic basis for ranking protein hits.

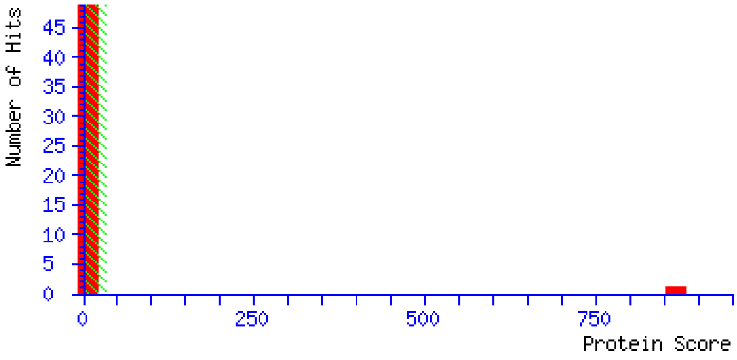

Matched peptide sequences: shown in **Bold Red**

```
  1 MGEGEENKNI QEEVNKEANI QKEVDVEEER LKYLDFVQTG AVFALVSFSK
 51 LYLFAKDVSG PFKPYVENAG GRFKSVVRPI YYKFQPVSNE ILKFADHKVD
101 ESVTILDLFV PPIVKQLCTQ AYSVARDAPV VACALTYYLL SPNEKFYMVL
151 YGDG
```

Matched peptide information:

Start - End	Observed	Mr(expt)	Mr(calc)	ppm	Miss	Sequence
23 - 30	1004.4271	1003.4198	1003.4458	-26	0	K.EVDVEEER.L (Ions score 48)
33 - 50	1991.9822	1990.9749	1991.0404	-33	0	K.YLDFVQTGAVFALVSFSK.L (Ions score 124)
57 - 72	1692.7847	1691.7774	1691.8267	-29	1	K.DVSGPFKPYVENAGGR.F (Ions score 127)
75 - 83	1124.6118	1123.6045	1123.6390	-31	1	K.SVVRPIYYK.F (Ions score 42)
84 - 93	1174.6091	1173.6018	1173.6394	-32	0	K.FQPVSNEILK.F (Ions score 65)
94 - 115	2482.2817	2481.2744	2481.3519	-31	1	K.FADHKVDESVTILDLFVPPIVK.Q (Ions score 153)
99 - 115	1884.0078	1883.0005	1883.0656	-35	0	K.VDESVTILDLFVPPIVK.Q (Ions score 94)
116 - 126	1296.6011	1295.5938	1295.6292	-27	0	K.QLCTQAYSVAR.D (Ions score 84)
127 - 145	2124.0017	2122.9944	2123.0609	-31	0	R.DAPVVACALTYYLLSPNEK.F (Ions score 131)

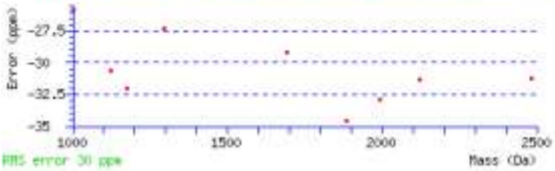

Spot No.: 44

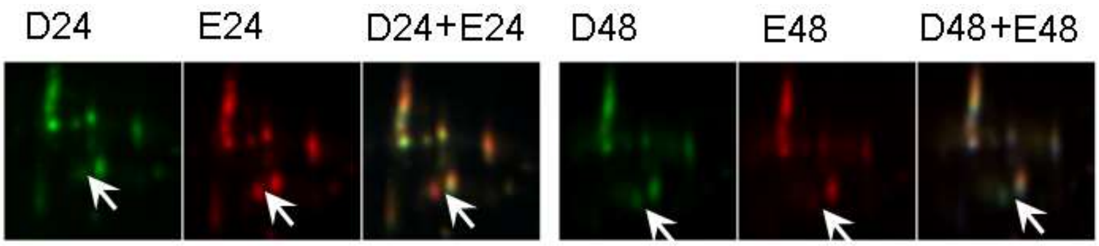

Accession No.: **scaffold0464_317117.mRNA1**

Plant species: ***Hevea brasiliensis***

Protein name: **Actin-depolymerizing factor 2**

Peptide sequences: **K.LGEPTQSYEDFTASLPADECR.Y; R.YAVYDFDFVTEENCQK.S; R.IFFIAWSPDTSR.V**

PFF Mascot score: **[244]** Sequence coverage %: **[35]**

Matched peptides No.: **[3]**

Calculated Mr: **16220** Calculated *p*I: **5.92**

Annotated PFF **spectra:**

Ions score is -10*Log(P), where P is the probability that the observed match is a random event.
Individual ions scores > 30 indicate identity or extensive homology (p<0.05).
Protein scores are derived from ions scores as a non-probabilistic basis for ranking protein hits.

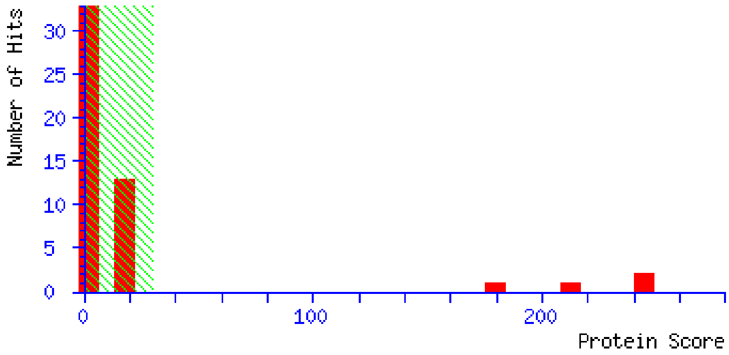

Matched peptide sequences: shown in **Bold Red**

```
  1 MANAASGMAV HDDCKLRFLE LKAKRTYRYI VFKIEEKAKQ VIVEKLGEPT
 51 QSYEDFTASL PADECRYAVY DFDFVTEENC QKSRIFFIAW SPDTSRVRSK
101 MIYASSKDRF KRELDGIQVE LQATDPTEMG LDVFKSRAS
```

Matched peptide information:

Start - End	Observed	Mr(expt)	Mr(calc)	ppm	Miss	Sequence
46 - 66	2386.0156	2385.0083	2385.0431	-15	0	K.LGEPTQSYEDFTASLPADECR.Y (Ions score 116)
67 - 82	2027.8496	2026.8423	2026.8619	-10	0	R.YAVYDFDFVTEENCQK.S (Ions score 54)
85 - 96	1439.7250	1438.7177	1438.7245	-5	0	R.IFFIAWSPDTSR.V (Ions score 73)

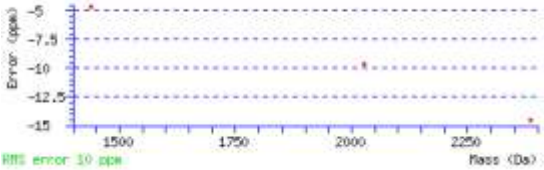

Spot No.: **45**

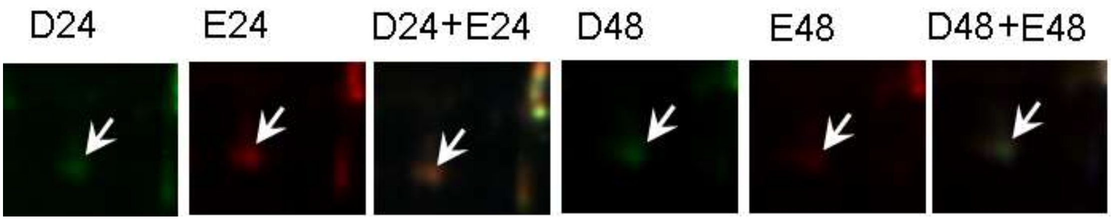

Accession No.: **scaffold0464_317117.mRNA1**

Plant species: ***Hevea brasiliensis***

Protein name： **Actin-depolymerizing factor 2**

Peptide sequences: **K.LGEPTQSYEDFTASLPADECR.Y；**
R.YAVYDFDFVTEENCQK.S； **R.IFFIAWSPDTSR.V；**
R.ELDGIQVELQATDPTEMGLDVFK.S

PFF Mascot score: **[365]** Sequence coverage %: **[51]**

Matched peptides No.: **[4]**

Calculated Mr: **16220** Calculated *p*I: **5.92**

Annotated PFF spectra:

Ions score is -10*Log(P), where P is the probability that the observed match is a random event.
Individual ions scores > 31 indicate identity or extensive homology ($p<0.05$).
Protein scores are derived from ions scores as a non-probabilistic basis for ranking protein hits.

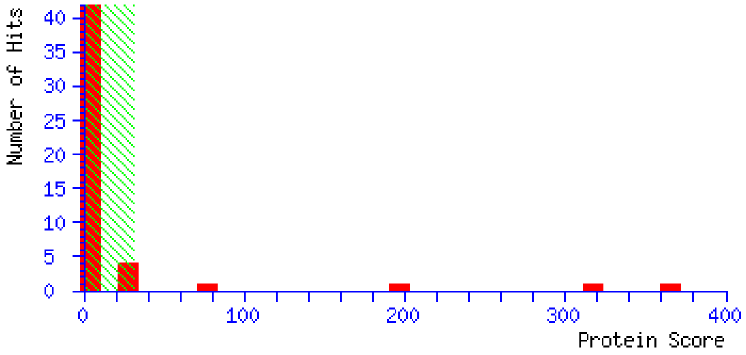

Matched peptide sequences: shown in **Bold Red**

```
  1 MANAASGMAV HDDCKLRFLE LKAKRTYRYI VFKIEEKAKQ VIVEKLGEPT
 51 QSYEDFTASL PADECRYAVY DFDFVTEENC QKSRIFFIAW SPDTSRVRSK
101 MIYASSKDRF KRELDGIQVE LQATDPTEMG LDVFKSRAS
```

1 MANAASGMAV HDDCKLRFLE LKAKRTYRYI VFKIEEKAKQ VIVEK**LGEPT**
51 **QSYEDFTASL** **PADECRYAVY** **DFDFVTEENC** **QK**SR**IFFIAW** **SPDTSR**VRSK
101 MIYASSKDRF KR**ELDGIQVE** **LQATDPTEMG** **LDVFK**SRAS

Matched peptide information:

Start - End	Observed	Mr(expt)	Mr(calc)	ppm	Miss	Sequence	
46 - 66	2385.9663	2384.9590	2385.0431	-35	0	K.LGEPTQSYEDFTASLPADECR.Y	(Ions score 130)
67 - 82	2027.7893	2026.7820	2026.8619	-39	0	R.YAVYDFDFVTEENCQK.S	(Ions score 89)
85 - 96	1439.6851	1438.6778	1438.7245	-32	0	R.IFFIAWSPDTSR.V	(Ions score 97)
113 - 135	2548.1475	2547.1402	2547.2414	-40	0	R.ELDGIQVELQATDPTEMGLDVFK.S	(Ions score 45)

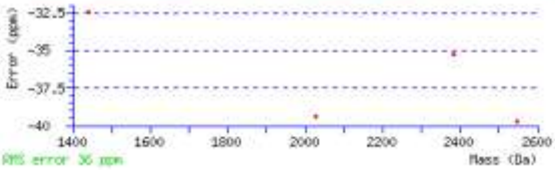

Spot No.: **46**

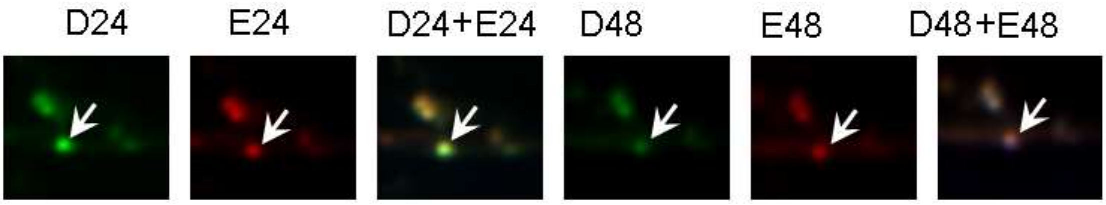

Accession No.: **scaffold0155_515853.mRNA1**

Plant species: ***Hevea brasiliensis***

Protein name： **Pro-hevein**

Peptide sequences: **K.YGWTAFCGPVGAHGQPSCGK.C；R.IVDQCSNGGLDLDVNVFR.Q；R.QLDTDGKGYER.G**

PFF Mascot score: **[329]** Sequence coverage %: **[23]**

Matched peptides No.: **[3]**

Calculated Mr: **23042** Calculated *p*I: **8.15**

Annotated PFF **spectra:**

Ions score is -10*Log(P), where P is the probability that the observed match is a random event.
Individual ions scores > 30 indicate identity or extensive homology (p<0.05).
Protein scores are derived from ions scores as a non-probabilistic basis for ranking protein hits.

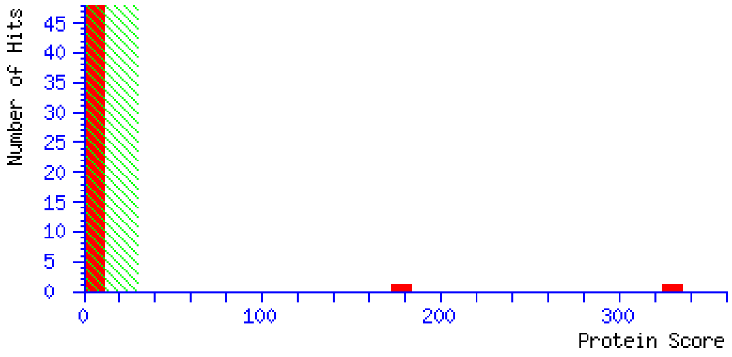

Matched peptide sequences: shown in Bold Red

```
  1 MGRVMNIFIV VLLCLTGVAI AEQCGRQAGG KLCPNNLCCS QYGWCGSSDD
 51 YCSPSKNCQS NCKGGGGGGG GGGGSASNVL ATYHLYNPQQ HGWDLNAVSA
101 YCSTWDANKP YSWRSKYGWT AFCGPVGAHG QPSCGKCLSV TNTGTGAKTT
151 VRIVDQCSNG GLDLDVNVFR QLDTDGKGYE RGHLTVNYQF VNCGDSFNPL
201 FSIMKSSVIN
```

Matched (bold red): 101 … YSWRSK**YGWT AFCGPVGAHG QPSCGK**CLSV …; 151 VR**IVDQCSNG GLDLDVNVFR QLDTDGKGYE R**GHLTVNYQF …

Matched peptide information:

Start - End	Observed	Mr(expt)	Mr(calc)	ppm	Miss	Sequence
117 - 136	2136.8838	2135.8765	2135.9306	-25	0	K.YGWTAFCGPVGAHGQPSCGK.C (Ions score 88)
153 - 170	2020.9237	2019.9164	2019.9684	-26	0	R.IVDQCSNGGLDLDVNVFR.Q (Ions score 150)
171 - 181	1281.5817	1280.5744	1280.5997	-20	1	R.QLDTDGKGYER.G (Ions score 91)

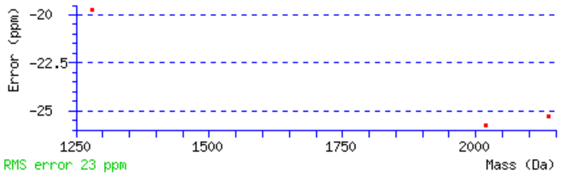

Spot No.: **47**

D24 E24 D24+E24 D48 E48 D48+E48

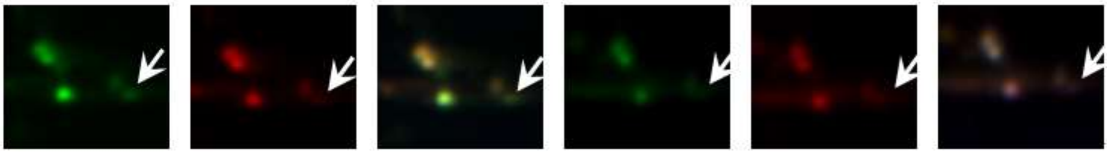

Accession No.: **scaffold0696_83618.mRNA1**

Plant species: ***Hevea brasiliensis***

Protein name：**14 kDa zinc-binding protein**

Peptide sequences: **K.AAALTAVPSDSPTIFDK.I；K.EIPCNVVYEDDKVLAFK.D；K.DISPQAPTHILIIPK.V；K.QEGLEDGFR.I；R.IVINDGPNGCQSVYHLHIHLLGGR.Q**

PFF Mascot score: **[144]**

Sequence coverage %: **[56]**

Matched peptides No.: **[5]**

Calculated Mr: **16097**

Calculated *p*I: **6.29**

Annotated PFF **spectra:**

Ions score is -10*Log(P), where P is the probability that the observed match is a random event.
Individual ions scores > 30 indicate identity or extensive homology (p<0.05).
Protein scores are derived from ions scores as a non-probabilistic basis for ranking protein hits.

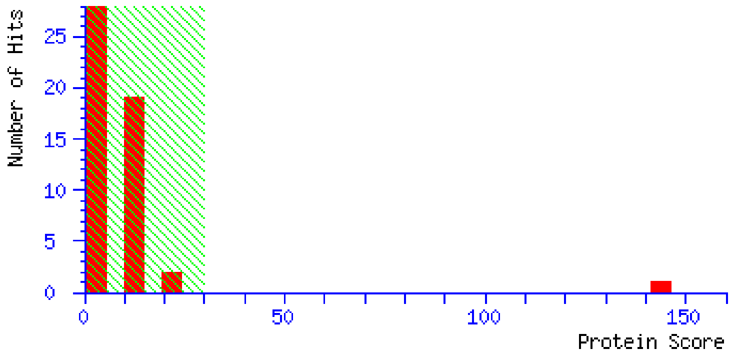

Matched peptide sequences: shown in **Bold Red**

1 MEENIKGRIS VLSSHIMASE K**AAALTAVPS DSPTIFDK**II NK**EIPCNVVY**
51 **EDDKVLAFKD ISPQAPTHIL IIPK**VKDGLT GLSKAEERHC DILGRLLYTA
101 KLIAK**QEGLE DGFRIVINDG PNGCQSVYHL HIHLLGGR**QM NWPPG

Matched peptide information:

Start - End	Observed	Mr(expt)	Mr(calc)	ppm	Miss	Sequence
22 - 38	1703.8137	1702.8064	1702.8778	-42	0	K.AAALTAVPSDSPTIFDK.I (Ions score 29)
43 - 59	2038.9080	2037.9007	2038.0081	-53	1	K.EIPCNVVYEDDKVLAFK.D (Ions score 9)
60 - 74	1642.8879	1641.8806	1641.9454	-39	0	K.DISPQAPTHILIIPK.V (Ions score 34)
106 - 114	1050.4457	1049.4384	1049.4778	-38	0	K.QEGLEDGFR.I (Ions score 46)
115 - 138	2669.2742	2668.2669	2668.3656	-37	0	R.IVINDGPNGCQSVYHLHIHLLGGR.Q (Ions score 25)

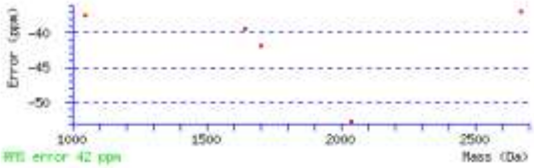

Spot No.: **48**

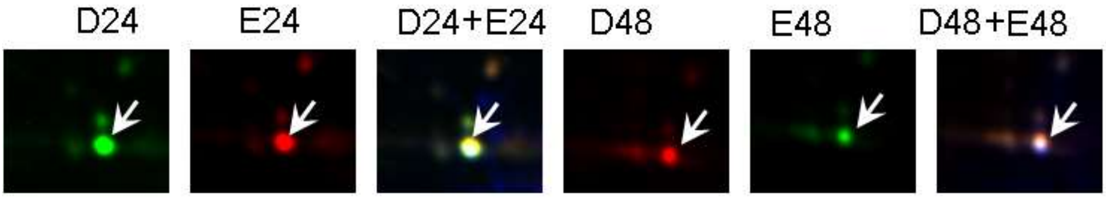

Accession No.: **scaffold0155_515853.mRNA1**

Plant species: ***Hevea brasiliensis***

Protein name： **Pro-hevein**

Peptide sequences: **K.YGWTAFCGPVGAHGQPSCGK.C；R.IVDQCSNGGLDLDVNVFR.Q；R.QLDTDGKGYER.G**

PFF Mascot score: **[348]** Sequence coverage %: **[23]**

Matched peptides No.: **[3]**

Calculated Mr: **23042** Calculated *p*I: **8.15**

Annotated PFF **spectra:**

Ions score is -10*Log(P), where P is the probability that the observed match is a random event.
Individual ions scores > 31 indicate identity or extensive homology (p<0.05).
Protein scores are derived from ions scores as a non-probabilistic basis for ranking protein hits.

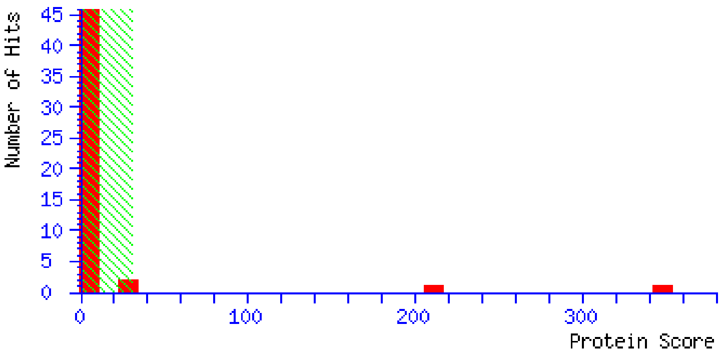

Matched peptide sequences: shown in Bold Red

1 MGRVMNIFIV VLLCLTGVAI AEQCGRQAGG KLCPNNLCCS QYGWCGSSDD
51 YCSPSKNCQS NCKGGGGGGG GGGGSASNVL ATYHLYNPQQ HGWDLNAVSA
101 YCSTWDANKP YSWRSK**YGWT AFCGPVGAHG QPSCGK**CLSV TNTGTGAKTT
151 VR**IVDQCSNG GLDLDVNVFR QLDTDGKGYE R**GHLTVNYQF VNCGDSFNPL
201 FSIMKSSVIN

Matched peptide information:

Start - End	Observed	Mr(expt)	Mr(calc)	ppm	Miss	Sequence
117 - 136	2137.0835	2136.0762	2135.9306	68	0	K.YGWTAFCGPVGAHGQPSCGK.C (Ions score 101)
153 - 170	2021.1191	2020.1118	2019.9684	71	0	R.IVDQCSNGGLDLDVNVFR.Q (Ions score 156)
171 - 181	1281.6837	1280.6764	1280.5997	60	1	R.QLDTDGKGYER.G (Ions score 91)

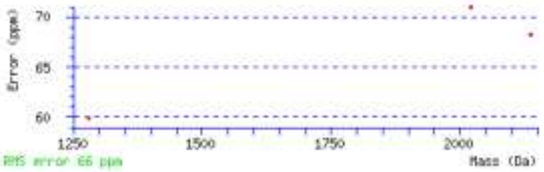

Spot No.: **49**

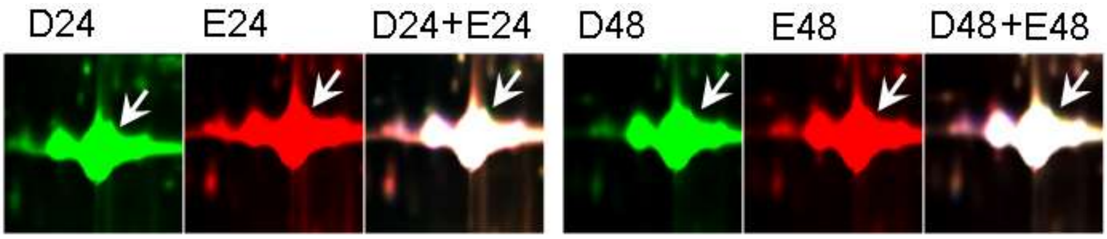

Accession No.: **scaffold1222_60641.mRNA1**

Plant species: ***Hevea brasiliensis***

Protein name： **Small rubber particle protein**

Peptide sequences: **K.DISGPLKPGVDTIENVVK.T; K.TVVTPVYYIPLEAVK.F; K.FVDKTVDVSVTSLDGVVPPVIK.Q; K.TVDVSVTSLDGVVPPVIK.Q; K.QVSAQTYSVAQDAPR.I; R.IVLDVASSVFNTGVQEGAK.A; K.ALYANLEPK.A; K.AEQYAVITWR.A; R.ALNKLPLVPQVANVVVPTAVYFSEK.Y; K.LPLVPQVANVVVPTAVYFSEK.Y ; K.LPLVPQVANVVVPTAVYFSEKYNDVVR.G; R.VSSYLPLLPTEK.I**

PFF Mascot score: **[919]**

Sequence coverage %: **[74]**

Matched peptides No.: **[12]**

Calculated Mr: **22331**

Calculated *p*I: **4.80**

Annotated PFF **spectra:**

Ions score is -10*Log(P), where P is the probability that the observed match is a random event.
Individual ions scores > 31 indicate identity or extensive homology (p<0.05).
Protein scores are derived from ions scores as a non-probabilistic basis for ranking protein hits.

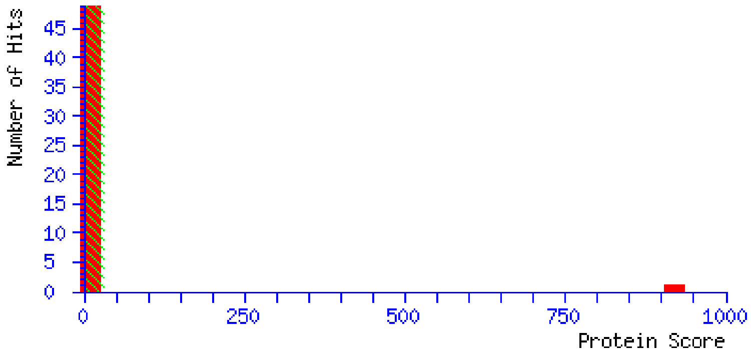

Matched peptide sequences: shown in Bold Red

```
  1 MAEEVEEERL KYLDFVRAAG VYAVDSFSTL YLYAKDISGP LKPGVDTIEN
 51 VVKTVVTPVY YIPLEAVKFV DKTVDVSVTS LDGVVPPVIK QVSAQTYSVA
101 QDAPRIVLDV ASSVFNTGVQ EGAKALYANL EPKAEQYAVI TWRALNKLPL
151 VPQVANVVVP TAVYFSEKYN DVVRGTTEQG YRVSSYLPLL PTEKITKVFG
201 DEAS
```

Matched peptide information:

Start - End	Observed	Mr(expt)	Mr(calc)	ppm	Miss	Sequence
36 - 53	1881.0944	1880.0871	1880.0255	33	1	K.DISGPLKPGVDTIENVVK.T (Ions score 82)
54 - 68	1692.0026	1690.9953	1690.9546	24	0	K.TVVTPVYYIPLEAVK.F (Ions score 85)
69 - 90	2314.3591	2313.3518	2313.2832	30	1	K.FVDKTVDVSVTSLDGVVPPVIK.Q (Ions score 27)
73 - 90	1825.0756	1824.0683	1824.0245	24	0	K.TVDVSVTSLDGVVPPVIK.Q (Ions score 23)
91 - 105	1620.8538	1619.8465	1619.7903	35	0	K.QVSAQTYSVAQDAPR.I (Ions score 139)
106 - 124	1934.0851	1933.0778	1933.0157	32	0	R.IVLDVASSVFNTGVQEGAK.A (Ions score 152)
125 - 133	1018.5795	1017.5722	1017.5495	22	0	K.ALYANLEPK.A (Ions score 53)
134 - 143	1236.6801	1235.6728	1235.6299	35	0	K.AEQYAVITWR.A (Ions score 88)
144 - 168	2696.6306	2695.6233	2695.5313	34	1	R.ALNKLPLVPQVANVVVPTAVYFSEK.Y (Ions score 55)
148 - 168	2270.3418	2269.3345	2269.2722	27	0	K.LPLVPQVANVVVPTAVYFSEK.Y (Ions score 28)
148 - 174	3016.7451	3015.7378	3015.6434	31	1	K.LPLVPQVANVVVPTAVYFSEKYNDVVR.G (Ions score 168)
183 - 194	1346.7797	1345.7724	1345.7493	17	0	R.VSSYLPLLPTEK.I (Ions score 20)

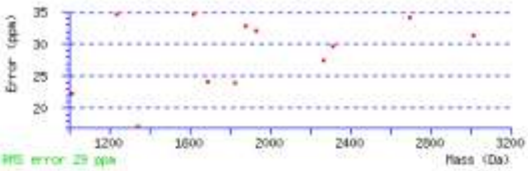

Spot No.: **50**

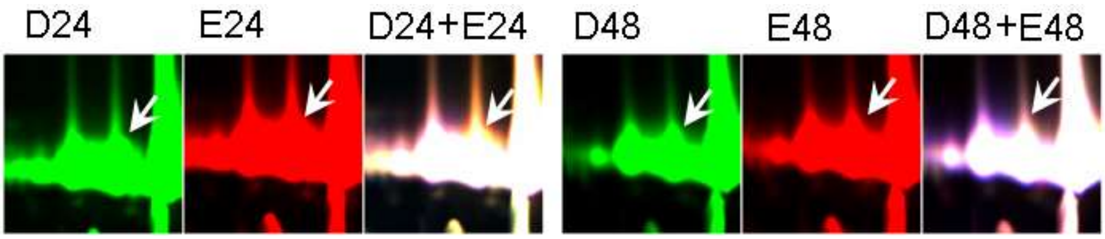

Accession No.: **scaffold1222_136753.mRNA1**

Plant species: ***Hevea brasiliensis***

Protein name：**Rubber elongation factor protein**

Peptide sequences: **K.DKSGPLQPGVDIIEGPVK.N ； K.NVAVPLYNR.F； K.FVDSTVVASVTIIDR.S； K.DASIQVVSAIR.A； R.SLASSLPGQTK.I**

PFF Mascot score: **[479]** Sequence coverage %: **[46]**

Matched peptides No.: **[5]**

Calculated Mr: **14713** Calculated p*I*: **5.04**

Annotated PFF **spectra:**

Ions score is -10*Log(P), where P is the probability that the observed match is a random event.
Individual ions scores > 31 indicate identity or extensive homology (p<0.05).
Protein scores are derived from ions scores as a non-probabilistic basis for ranking protein hits.

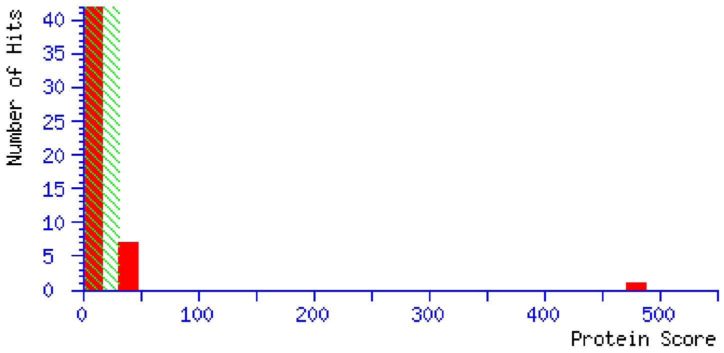

Matched peptide sequences: shown in Bold Red

```
  1 MAEDEDNQQG QGEGLKYLGF VQDAATYAVT TFSNVYLFAK DKSGPLQPGV
 51 DIIEGPVKNV AVPLYNRFSY IPNGALKFVD STVVASVTII DRSLPPIVKD
101 ASIQVVSAIR AAPEAARSLA SSLPGQTKIL AKVFYGEN
```

1 MAEDEDNQQG QGEGLKYLGF VQDAATYAVT TFSNVYLFAK **DKSGPLQPGV**
51 **DIIEGPVKNV AVPLYNR**FSY IPNGALK**FVD STVVASVTII DR**SLPPIVK**D**
101 **ASIQVVSAIR** AAPEAAR**SLA SSLPGQTK**IL AKVFYGEN

Matched peptide information:

Start - End	Observed	Mr(expt)	Mr(calc)	ppm	Miss	Sequence
41 - 58	1849.0110	1848.0037	1847.9993	2	1	K.DKSGPLQPGVDIIEGPVK.N (Ions score 142)
59 - 67	1045.5704	1044.5631	1044.5716	-8	0	K.NVAVPLYNR.F (Ions score 58)
78 - 92	1621.8785	1620.8712	1620.8723	-1	0	K.FVDSTVVASVTIIDR.S (Ions score 139)
100 - 110	1158.6448	1157.6375	1157.6404	-3	0	K.DASIQVVSAIR.A (Ions score 110)
118 - 128	1088.5717	1087.5644	1087.5873	-21	0	R.SLASSLPGQTK.I (Ions score 30)

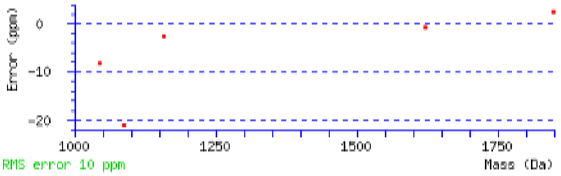

Spot No.: **51**

D24 E24 D24+E24 D48 E48 D48+E48

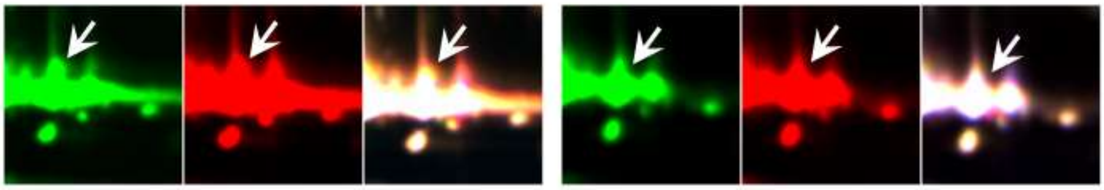

Accession No.: **scaffold1222_136753.mRNA1**

Plant species: ***Hevea brasiliensis***

Protein name：**Rubber elongation factor protein**

Peptide sequences: **K.YLGFVQDAATYAVTTFSNVYLFAK.D；K.DKSGPLQPGVDIIEGPVK.N；K.NVAVPLYNR.F；K.FVDSTVVASVTIIDR.S；K.DASIQVVSAIR.A；R.SLASSLPGQTK.I**

PFF Mascot score: **[560]**

Sequence coverage %: **[63]**

Matched peptides No.: **[6]**

Calculated Mr: **14713**

Calculated *p*I: **5.04**

Annotated PFF **spectra:**

Ions score is -10*Log(P), where P is the probability that the observed match is a random event.
Individual ions scores > 31 indicate identity or extensive homology (p<0.05).
Protein scores are derived from ions scores as a non-probabilistic basis for ranking protein hits.

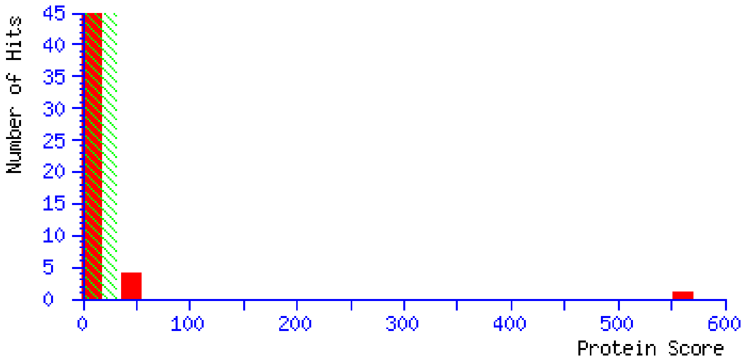

Matched peptide sequences: shown in Bold Red

```
  1 MAEDEDNQQG QGEGLKYLGF VQDAATYAVT TFSNVYLFAK DKSGPLQPGV
 51 DIIEGPVKNV AVPLYNRFSY IPNGALKFVD STVVASVTII DRSLPPIVKD
101 ASIQVVSAIR AAPEAARSLA SSLPGQTKIL AKVFYGEN
```

Bold red: 1 MAEDEDNQQG QGEGLK**YLGF VQDAATYAVT TFSNVYLFAK DKSGPLQPGV**
51 **DIIEGPVKNV AVPLYNR**FSY IPNGALK**FVD STVVASVTII DR**SLPPIVK**D**
101 **ASIQVVSAIR** AAPEAAR**SLA SSLPGQTK**IL AKVFYGEN

Matched peptide information:

Start - End	Observed	Mr(expt)	Mr(calc)	ppm	Miss	Sequence
17 - 40	2689.3093	2688.3020	2688.3476	-17	0	K.YLGFVQDAATYAVTTFSNVYLFAK.D (Ions score 89)
41 - 58	1848.9688	1847.9615	1847.9993	-20	1	K.DKSGPLQPGVDIIEGPVK.N (Ions score 147)
59 - 67	1045.5529	1044.5456	1044.5716	-25	0	K.NVAVPLYNR.F (Ions score 41)
78 - 92	1621.8500	1620.8427	1620.8723	-18	0	K.FVDSTVVASVTIIDR.S (Ions score 119)
100 - 110	1158.6237	1157.6164	1157.6404	-21	0	K.DASIQVVSAIR.A (Ions score 110)
118 - 128	1088.5576	1087.5503	1087.5873	-34	0	R.SLASSLPGQTK.I (Ions score 53)

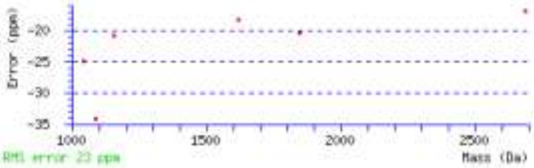

Spot No.: 52

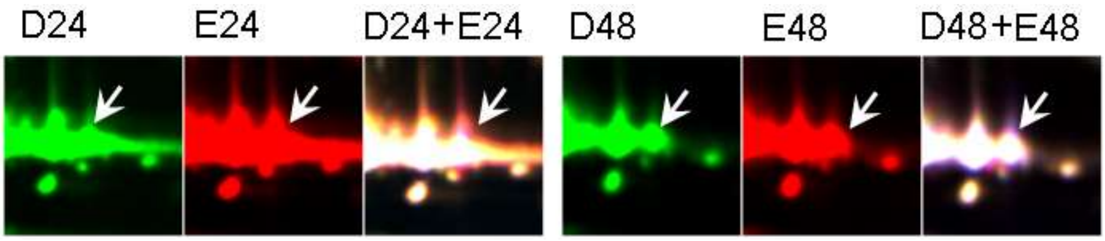

Accession No.: **scaffold1222_136753.mRNA1**

Plant species: ***Hevea brasiliensis***

Protein name：**Rubber elongation factor protein**

Peptide sequences: **K.DKSGPLQPGVDIIEGPVK.N; K.NVAVPLYNR.F; K.FVDSTVVASVTIIDR.S; K.DASIQVVSAIR.A; R.SLASSLPGQTK.I**

PFF Mascot score: **[358]** Sequence coverage %: **[46]**

Matched peptides No.: **[5]**

Calculated Mr: **14713** Calculated *p*I: **5.04**

Annotated PFF **spectra:**

Ions score is -10*Log(P), where P is the probability that the observed match is a random event.
Individual ions scores > 31 indicate identity or extensive homology ($p<0.05$).
Protein scores are derived from ions scores as a non-probabilistic basis for ranking protein hits.

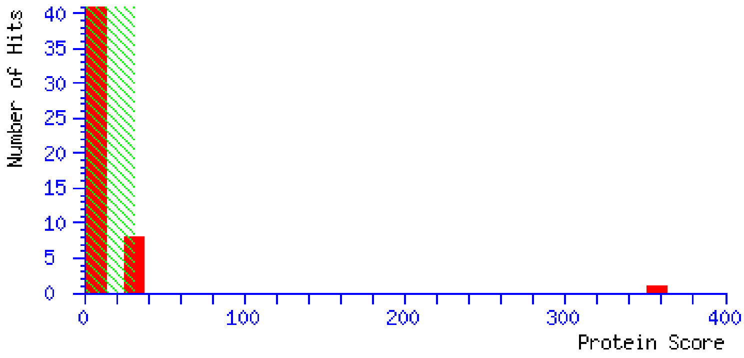

Matched peptide sequences: shown in Bold Red

1 MAEDEDNQQG QGEGLKYLGF VQDAATYAVT TFSNVYLFAK **DKSGPLQPGV**
51 **DIIEGPVKNV AVPLYNR**FSY IPNGALK**FVD STVVASVTII DR**SLPPIVK**D**
101 **ASIQVVSAIR** AAPEAAR**SLA SSLPGQTK**IL AKVFYGEN

Matched peptide information:

Start - End	Observed	Mr(expt)	Mr(calc)	ppm	Miss	Sequence
41 - 58	1849.0087	1848.0014	1847.9993	1	1	K.DKSGPLQPGVDIIEGPVK.N (Ions score 62)
59 - 67	1045.5721	1044.5648	1044.5716	-6	0	K.NVAVPLYNR.F (Ions score 63)
78 - 92	1621.8833	1620.8760	1620.8723	2	0	K.FVDSTVVASVTIIDR.S (Ions score 125)
100 - 110	1158.6415	1157.6342	1157.6404	-5	0	K.DASIQVVSAIR.A (Ions score 97)
118 - 128	1088.5811	1087.5738	1087.5873	-12	0	R.SLASSLPGQTK.I (Ions score 10)

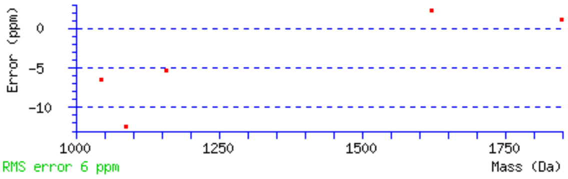

Spot No.: **53**

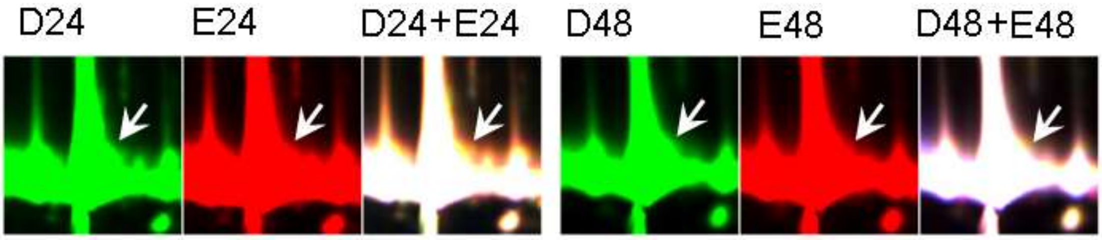

Accession No.: **scaffold1222_136753.mRNA1**

Plant species: ***Hevea brasiliensis***

Protein name: **Rubber elongation factor protein**

Peptide sequences: **K.DKSGPLQPGVDIIEGPVK.N; K.NVAVPLYNR.F; K.FVDSTVVASVTIIDR.S; K.DASIQVVSAIR.A; R.SLASSLPGQTK.I**

PFF Mascot score: **[433]**

Sequence coverage %: **[46]**

Matched peptides No.: **[5]**

Calculated Mr: **14713**

Calculated *p*I: **5.04**

Annotated PFF **spectra:**

Ions score is -10*Log(P), where P is the probability that the observed match is a random event.
Individual ions scores > 31 indicate identity or extensive homology (p<0.05).
Protein scores are derived from ions scores as a non-probabilistic basis for ranking protein hits.

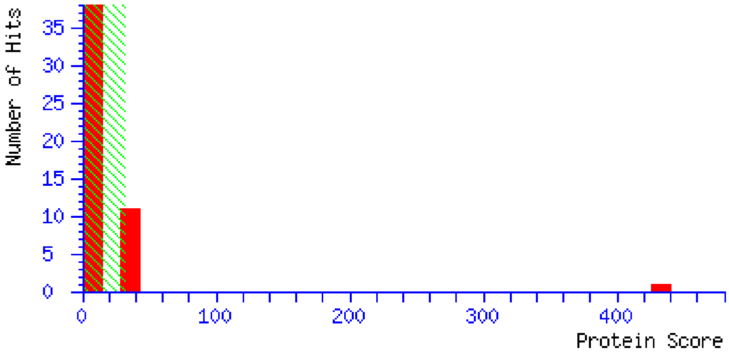

Matched peptide sequences: shown in Bold Red

```
  1 MAEDEDNQQG QGEGLKYLGF VQDAATYAVT TFSNVYLFAK DKSGPLQPGV
 51 DIIEGPVKNV AVPLYNRFSY IPNGALKFVD STVVASVTII DRSLPPIVKD
101 ASIQVVSAIR AAPEAARSLA SSLPGQTKIL AKVFYGEN
```

(Red in the original: 41–58 DKSGPLQPGV DIIEGPVK; 59–67 NV AVPLYNR; 78–92 FVD STVVASVTII DR; 100–110 D ASIQVVSAIR; 118–128 SLA SSLPGQTK)

Matched peptide information:

Start	-	End	Observed	Mr(expt)	Mr(calc)	ppm	Miss	Sequence
41	-	58	1848.9884	1847.9811	1847.9993	-10	1	K.DKSGPLQPGVDIIEGPVK.N (Ions score 132)
59	-	67	1045.5653	1044.5580	1044.5716	-13	0	K.NVAVPLYNR.F (Ions score 38)
78	-	92	1621.8668	1620.8595	1620.8723	-8	0	K.FVDSTVVASVTIIDR.S (Ions score 146)
100	-	110	1158.6339	1157.6266	1157.6404	-12	0	K.DASIQVVSAIR.A (Ions score 114)
118	-	128	1088.5704	1087.5631	1087.5873	-22	0	R.SLASSLPGQTK.I (Ions score 3)

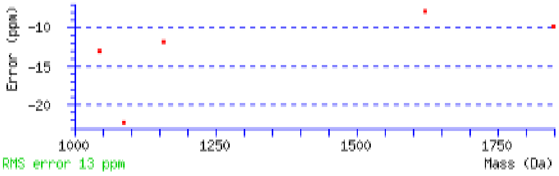

Spot No.: **54**

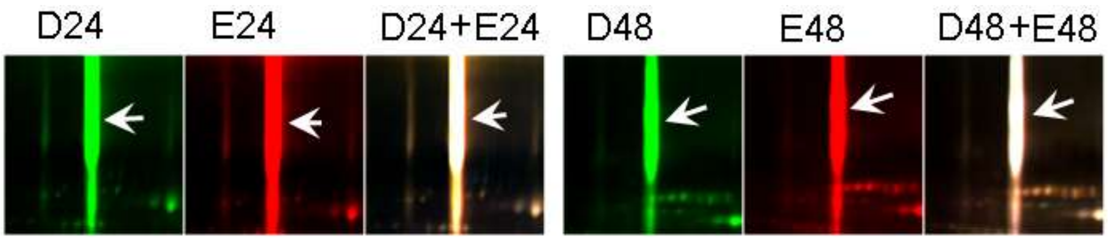

Accession No.: **scaffold1222_136753.mRNA1**

Plant species: ***Hevea brasiliensis***

Protein name: **Rubber elongation factor protein**

Peptide sequences: **K.DKSGPLQPGVDIIEGPVK.N; K.NVAVPLYNR.F; K.FVDSTVVASVTIIDR.S; K.DASIQVVSAIR.A; K.YLGFVQDAATYAVTTFSNVYLFAK.D**

PFF Mascot score: **[654]**

Sequence coverage %: **[55]**

Matched peptides No.: **[5]**

Calculated Mr: **14713**

Calculated *p*I: **5.04**

Annotated PFF **spectra:**

Ions score is -10*Log(P), where P is the probability that the observed match is a random event.
Individual ions scores > 31 indicate identity or extensive homology (p<0.05).
Protein scores are derived from ions scores as a non-probabilistic basis for ranking protein hits.

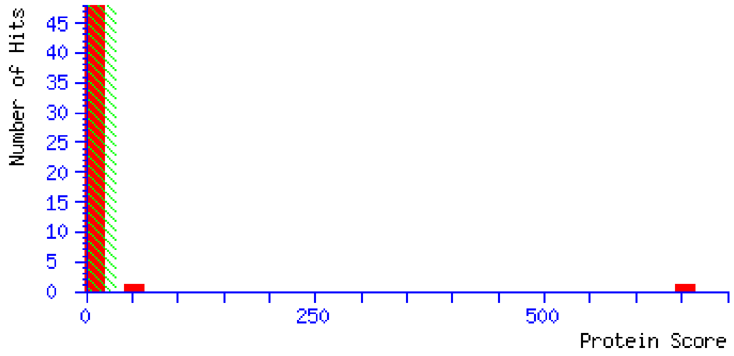

Matched peptide sequences: shown in **Bold Red**

```
  1 MAEDEDNQQG QGEGLKYLGF VQDAATYAVT TFSNVYLFAK DKSGPLQPGV
 51 DIIEGPVKNV AVPLYNRFSY IPNGALKFVD STVVASVTII DRSLPPIVKD
101 ASIQVVSAIR AAPEAARSLA SSLPGQTKIL AKVFYGEN
```

Bold red residues: 17–40 (**YLGF VQDAATYAVT TFSNVYLFAK**), 41–58 (**DKSGPLQPGV DIIEGPVK**), 59–67 (**NV AVPLYNR**), 78–92 (**FVD STVVASVTII DR**), 101–110 (**ASIQVVSAIR**).

Matched peptide information:

Start - End	Observed	Mr(expt)	Mr(calc)	ppm	Miss	Sequence
17 - 40	2689.2146	2688.2073	2688.3476	-52	0	K.YLGFVQDAATYAVTTFSNVYLFAK.D (Ions score 199)
41 - 58	1848.9199	1847.9126	1847.9993	-47	1	K.DKSGPLQPGVDIIEGPVK.N (Ions score 141)
59 - 67	1045.5411	1044.5338	1044.5716	-36	0	K.NVAVPLYNR.F (Ions score 72)
78 - 92	1621.8131	1620.8058	1620.8723	-41	0	K.FVDSTVVASVTIIDR.S (Ions score 132)
100 - 110	1158.6027	1157.5954	1157.6404	-39	0	K.DASIQVVSAIR.A (Ions score 109)

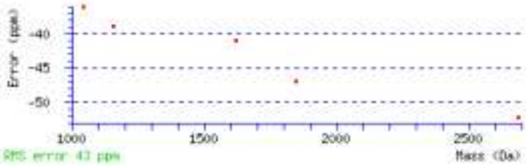

Spot No.: **55**

D24 E24 D24+E24 D48 E48 D48+E48

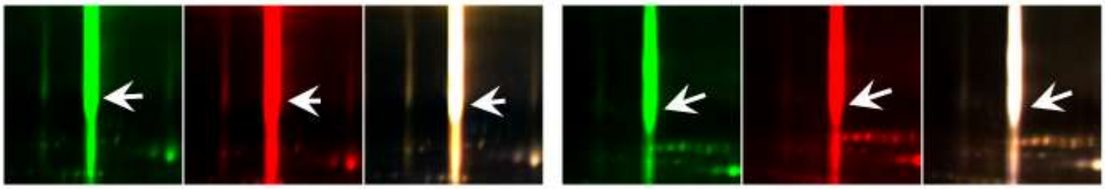

Accession No.: **scaffold1222_136753.mRNA1**

Plant species: ***Hevea brasiliensis***

Protein name：**Rubber elongation factor protein**

Peptide sequences: **K.DKSGPLQPGVDIIEGPVK.N; K.NVAVPLYNR.F; K.FVDSTVVASVTIIDR.S; K.DASIQVVSAIR.A; K.YLGFVQDAATYAVTTFSNVYLFAK.D**

PFF Mascot score: **[611]** Sequence coverage %: **[55]**

Matched peptides No.: **[5]**

Calculated Mr: **14713** Calculated *p*I: **5.04**

Annotated PFF **spectra:**

Ions score is -10*Log(P), where P is the probability that the observed match is a random event.
Individual ions scores > 31 indicate identity or extensive homology (p<0.05).
Protein scores are derived from ions scores as a non-probabilistic basis for ranking protein hits.

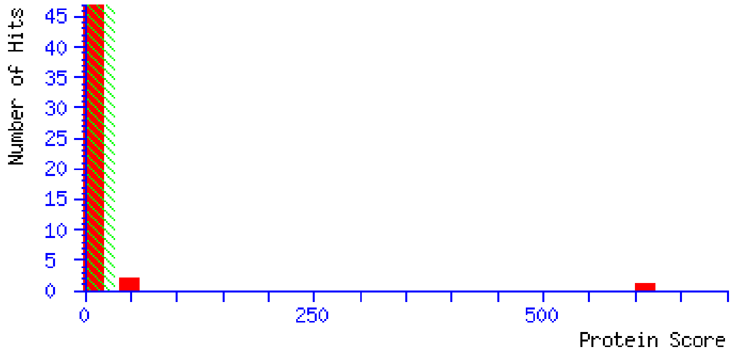

Matched peptide sequences: shown in Bold Red

```
  1 MAEDEDNQQG QGEGLKYLGF VQDAATYAVT TFSNVYLFAK DKSGPLQPGV
 51 DIIEGPVKNV AVPLYNRFSY IPNGALKFVD STVVASVTII DRSLPPIVKD
101 ASIQVVSAIR AAPEAARSLA SSLPGQTKIL AKVFYGEN
```

Bold red: 1 MAEDEDNQQG QGEGLK**YLGF VQDAATYAVT TFSNVYLFAK DKSGPLQPGV**
51 **DIIEGPVKNV AVPLYNR**FSY IPNGALK**FVD STVVASVTII DR**SLPPIVKD
101 **ASIQVVSAIR** AAPEAARSLA SSLPGQTKIL AKVFYGEN

Matched peptide information:

Start - End	Observed	Mr(expt)	Mr(calc)	ppm	Miss	Sequence
17 - 40	2689.2568	2688.2495	2688.3476	-36	0	K.YLGFVQDAATYAVTTFSNVYLFAK.D (Ions score 180)
41 - 58	1848.9453	1847.9380	1847.9993	-33	1	K.DKSGPLQPGVDIIEGPVK.N (Ions score 121)
59 - 67	1045.5551	1044.5478	1044.5716	-23	0	K.NVAVPLYNR.F (Ions score 64)
78 - 92	1621.8289	1620.8216	1620.8723	-31	0	K.FVDSTVVASVTIIDR.S (Ions score 137)
100 - 110	1158.6204	1157.6131	1157.6404	-24	0	K.DASIQVVSAIR.A (Ions score 109)

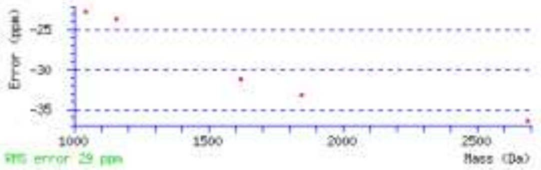

Spot No.: **56**

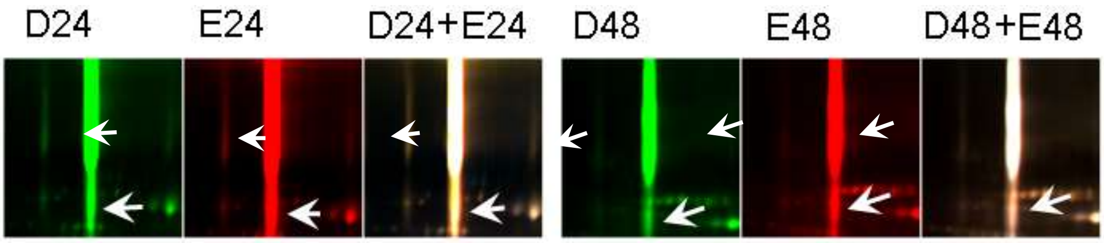

Accession No.: **scaffold1222_136753.mRNA1**

Plant species: ***Hevea brasiliensis***

Protein name：**Rubber elongation factor protein**

Peptide sequences: **K.SGPLQPGVDIIEGPVK.N; K.NVAVPLYNR.F; K.FVDSTVVASVTIIDR.S; K.DASIQVVSAIR.A**

PFF Mascot score: **[220]** Sequence coverage %: **[36]**

Matched peptides No.: **[4]**

Calculated Mr: **14713** Calculated *p*I: **5.04**

Annotated PFF **spectra:**

Ions score is -10*Log(P), where P is the probability that the observed match is a random event.
Individual ions scores > 32 indicate identity or extensive homology ($p<0.05$).
Protein scores are derived from ions scores as a non-probabilistic basis for ranking protein hits.

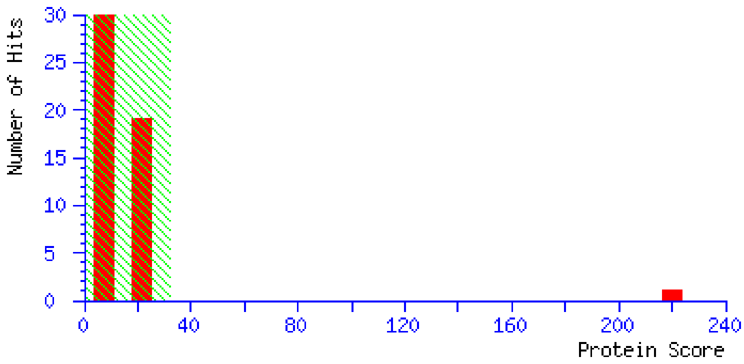

Matched peptide sequences: shown in Bold Red

```
  1 MAEDEDNQQG QGEGLKYLGF VQDAATYAVT TFSNVYLFAK DKSGPLQPGV
 51 DIIEGPVKNV AVPLYNRFSY IPNGALKFVD STVVASVTII DRSLPPIVKD
101 ASIQVVSAIR AAPEAARSLA SSLPGQTKIL AKVFYGEN
```

Matched peptide information:

Start - End	Observed	Mr(expt)	Mr(calc)	ppm	Miss	Sequence
43 - 58	1605.8586	1604.8513	1604.8774	-16	0	K.SGPLQPGVDIIEGPVK.N (Ions score 53)
59 - 67	1045.5825	1044.5752	1044.5716	3	0	K.NVAVPLYNR.F (Ions score 39)
78 - 92	1621.8805	1620.8732	1620.8723	1	0	K.FVDSTVVASVTIIDR.S (Ions score 81)
100 - 110	1158.6493	1157.6420	1157.6404	1	0	K.DASIQVVSAIR.A (Ions score 47)

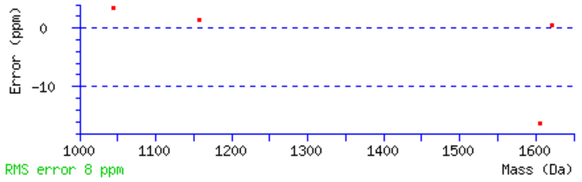

Spot No.: **57**

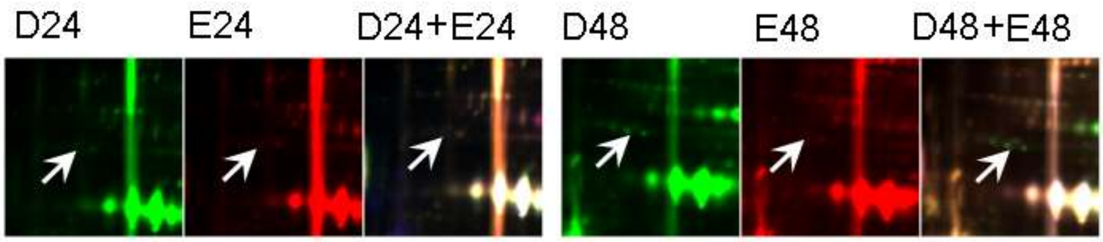

Accession No.: **scaffold1535_19427.mRNA1**

Plant species: ***Hevea brasiliensis***

Protein name：**Serine/threonine-protein phosphatase 2A 65 kDa regulatory subunit A beta isoform**

Peptide sequences: **R.LAAGEWFTAR.V；K.TDIMSIFEDLTQDDQDSVR.L；K.LLEPQDCVAHILPVIVNFSQDK.S；R.YMVANQLYELCEAVGPELTSSDLVPAYVR.L；R.ILNPELAIQR.I；K.LDQVNQVIGIDLLSQSLLPAIVELAEDR.H**

PFF Mascot score: **[621]**

Sequence coverage %: **[20]**

Matched peptides No.: **[6]**

Calculated Mr: **66211**

Calculated *p*I: **4.84**

Annotated PFF **spectra:**

Ions score is -10*Log(P), where P is the probability that the observed match is a random event.
Individual ions scores > 30 indicate identity or extensive homology (p<0.05).
Protein scores are derived from ions scores as a non-probabilistic basis for ranking protein hits.

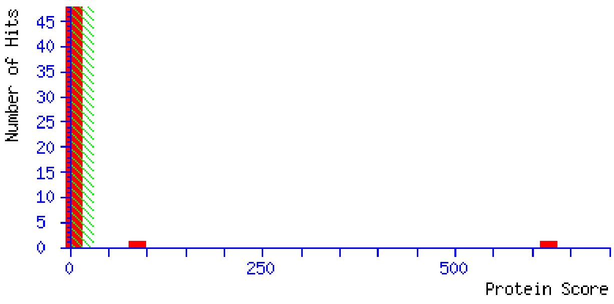

Matched peptide sequences: shown in **Bold Red**

1 MATIDEPLYP IAVLIDELKN EDIQLRLNSI RKLSTIARAL GEERTRKELI
51 PFLSENNDDD DEVLIAMAEE LGVFIPYIGG VEHANVLLPP LETLCTVEET
101 CVRDKAVESL CRIGVQIREQ DLVEYFIPLV KR**LAAGEWFT AR**VSSCGLFH
151 IAYPSAPETL KTELRAIYSQ LCQDDMPMVR RSAATNLGKF AATIEPAHLK
201 **TDIMSIFEDL TQDDQDSVR**L LAVEGCAALG K**LLEPQDCVA HILPVIVNFS**
251 **QDK**SWRVR**YM VANQLYELCE AVGPELTSSD LVPAYVR**LLC DNEAEVRIAA
301 AGKVTKFCR**I LNPELAIQR**I IPCVKELSTD SSQHVRSALA SVIMGMAPIL
351 GKDATIEQLL PIFLSLLKDE FPDVRLNIIS K**LDQVNQVIG IDLLSQSLLP**
401 **AIVELAEDR**H WRVRLAIIEY VPLLASQLGV GFFDDKLGAL CMQWLKDKVY
451 SIRDAAANNV KRLAEEFGPD WAMQHIVPQV LDMINNPHYL YRMTILHAIS
501 LLAPVMGSEI TCSTLLPVVV NTSKDRVPNI KFNVAKVLQS LIPIVDQSVV
551 DKTIRPCLVE LSEDPDVDVR FFATQALQSS DQVMMST

Matched peptide information:

Start - End	Observed	Mr(expt)	Mr(calc)	ppm	Miss	Sequence
133 - 142	1121.5145	1120.5072	1120.5665	-53	0	R.LAAGEWFTAR.V (Ions score 64)
201 - 219	2227.8699	2226.8626	2226.9951	-59	0	K.TDIMSIFEDLTQDDQDSVR.L (Ions score 109)
232 - 253	2535.1812	2534.1739	2534.3203	-58	0	K.LLEPQDCVAHILPVIVNFSQDK.S (Ions score 72)
259 - 287	3287.4021	3286.3948	3286.5890	-59	0	R.YMVANQLYELCEAVGPELTSSDLVPAYVR.L (Ions score 173)
310 - 319	1166.6260	1165.6187	1165.6819	-54	0	R.ILNPELAIQR.I (Ions score 54)
382 - 409	3061.4941	3060.4868	3060.6707	-60	0	K.LDQVNQVIGIDLLSQSLLPAIVELAEDR.H (Ions score 148)

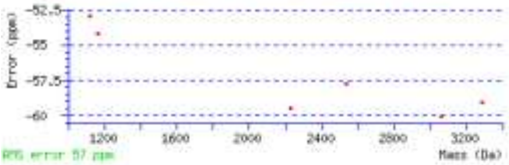

Spot No.: 58

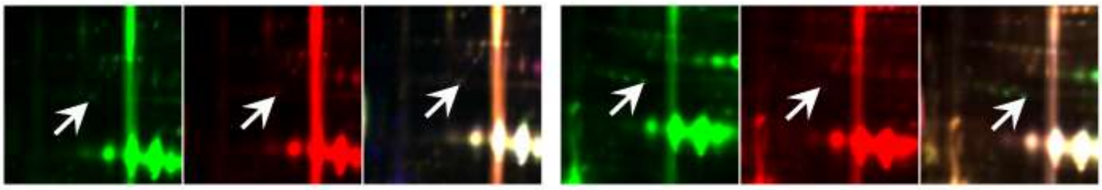

Accession No.: **scaffold0199_1207598.mRNA1**

Plant species: ***Hevea brasiliensis***

Protein name：**Ubiquitin receptor RAD23c**

Peptide sequences: **R.AAYNNPER.A；R.NSQQFQALR.A；R.LIQEHQADFLR.M**

PFF Mascot score: **[154]** Sequence coverage %: **[7]**

Matched peptides No.: **[3]**

Calculated Mr: **40862** Calculated *p*I: **4.71**

Annotated PFF **spectra:**

Ions score is -10*Log(P), where P is the probability that the observed match is a random event.
Individual ions scores > 32 indicate identity or extensive homology (p<0.05).
Protein scores are derived from ions scores as a non-probabilistic basis for ranking protein hits.

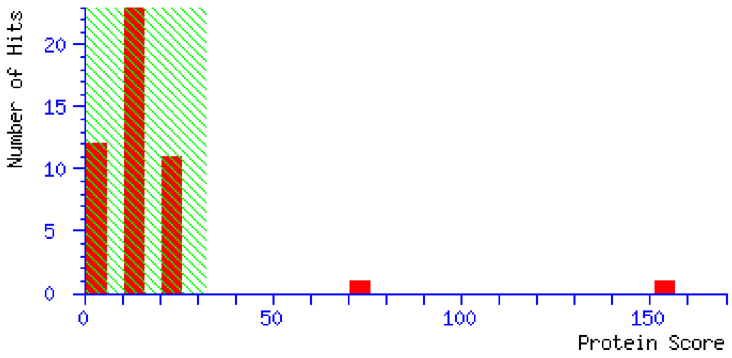

Matched peptide sequences: shown in Bold Red

```
  1 MKVYVKTLKG TNFEIEVKLE DTVADVKKII ETIQGVDIYP AAQQMLIHQG
 51 KVLKDGTTLE ESKVAENSFI VVMLSKSKVS SGGTSTASAA PPSQAQPASS
101 LPSSATRPST TTQASAPTAA PLQPAIESTP AVVNPVSSES DIYGQAASNL
151 VAGSNLEATI QQILDMGGGS WDHETVVRAL RAAYNNPERA VEYLYSGIPE
201 QAEARPVAQV PASGQAANPP AQAEQPAAPT SDGPNANPLD LFPQGLPSMG
251 SNASAGTLDF LRNSQQFQAL RAMVQANPQI LQPMLQELGK QNPHLVRLIQ
301 EHQADFLRMI NEPVEGEGNP LSQLASAMPQ AVTVTPEERE AIERLEAMGF
351 DRALVLEVFF ACNKNEELAA NYLLDHIHEF EE
```

Bold red: R**AAYNNPER**A (line 151); LR**NSQQFQAL** **R**AMVQANPQI and QNPHLVR**LIQ** (line 251); **EHQADFLR**MI (line 301)

Matched peptide information:

Start - End	Observed	Mr(expt)	Mr(calc)	ppm	Miss	Sequence	
182 - 189	934.4064	933.3992	933.4304	-33	0	R.AAYNNPER.A	(Ions score 23)
263 - 271	1091.5298	1090.5225	1090.5519	-27	0	R.NSQQFQALR.A	(Ions score 48)
298 - 308	1369.6824	1368.6751	1368.7150	-29	0	R.LIQEHQADFLR.M	(Ions score 83)

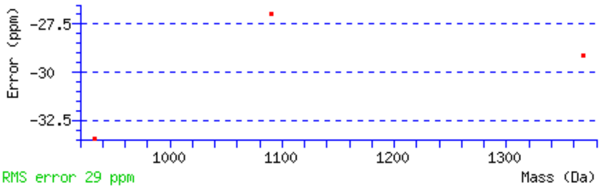

Spot No.: **59**

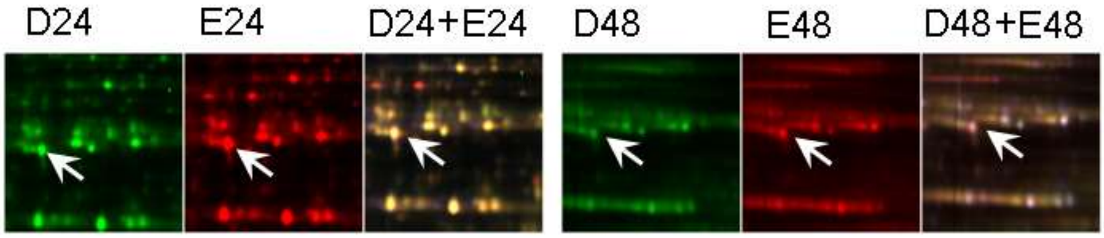

Accession No.: **scaffold0172_1138898.mRNA1**

Plant species: ***Hevea brasiliensis***

Protein name： **Guanosine nucleotide diphosphate dissociation inhibitor 2**

Peptide sequences: **K.YGLDDNTVDFIGHSLALHR.D；R.FQGGSPYIYPLYGLGELPQAFAR.L；K.VVCDPSYLPNK.V；R.AIAIMSHPIPNTNDSHSVQVILPQK.Q；R.RSDMYLFCCSYTHNVAPK.G；R.SDMYLFCCSYTHNVAPK.G**

PFF Mascot score: **[497]**

Sequence coverage %: **[21]**

Matched peptides No.: **[6]**

Calculated Mr: **50142**

Calculated *p*I: **5.54**

Annotated PFF **spectra:**

Ions score is -10*Log(P), where P is the probability that the observed match is a random event.
Individual ions scores > 30 indicate identity or extensive homology (p<0.05).
Protein scores are derived from ions scores as a non-probabilistic basis for ranking protein hits.

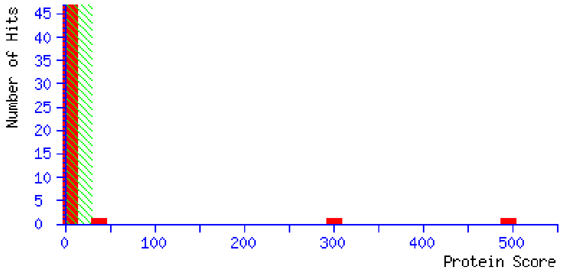

Matched peptide sequences: shown in Bold Red

```
  1 MDEEYDVIVL GTGLKECILS GLLSVDGLKV LHMDRNDYYG GESASLNLIQ
 51 LWKRFRGNDK PPAHLGSSRD YNVDMIPKFM MANGNLVRVL IHTDVTKYLY
101 FKAVDGSFVY NKGKVHKVPA TDIEALKSPL MGIFEKRRAR KFFIYVQDYN
151 ENDPKTHEGM DLTRVTTREL IAKYGLDDNT VDFIGHSLAL HRDDQYLDEP
201 ALDTVMRMKL YAESLARFQG GSPYIYPLYG LGELPQAFAR LSAVYGGTYM
251 LNKPECKVEF NEEGKVVGVT SEGETARCKK VVCDPSYLPN KVRKVGRVAR
301 AIAIMSHPIP NTNDSHSVQV ILPQKQLGRR SDMYLFCCSY THNVAPKGKY
351 IAFVSSEAET DHPEVELKPG IDLLGPVDEI FFEIYDRYEP VNEPSLDNCF
401 ISTSYDATTH FESTVTDVLN MYTLITGKVL DLSVDLSAAS AAE
```

Matched peptide information:

Start - End	Observed	Mr(expt)	Mr(calc)	ppm	Miss	Sequence
174 - 192	2142.9622	2141.9549	2142.0494	-44	0	K.YGLDDNTVDFIGHSLALHR.D (Ions score 117)
218 - 240	2544.1755	2543.1682	2543.2849	-46	0	R.FQGGSPYIYPLYGLGELPQAFAR.L (Ions score 182)
281 - 291	1291.5847	1290.5774	1290.6278	-39	0	K.VVCDPSYLPNK.V (Ions score 52)
301 - 325	2710.3091	2709.3018	2709.4272	-46	0	R.AIAIMSHPIPNTNDSHSVQVILPQK.Q (Ions score 75)
330 - 347	2248.8921	2247.8848	2247.9864	-45	1	R.RSDMYLFCCSYTHNVAPK.G (Ions score 39)
331 - 347	2092.8501	2091.8428	2091.8853	-20	0	R.SDMYLFCCSYTHNVAPK.G (Ions score 31)

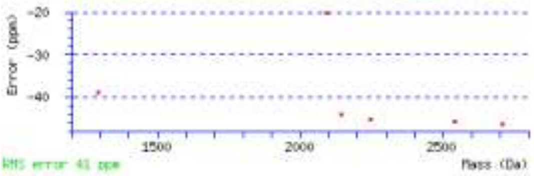

Spot No.: 60

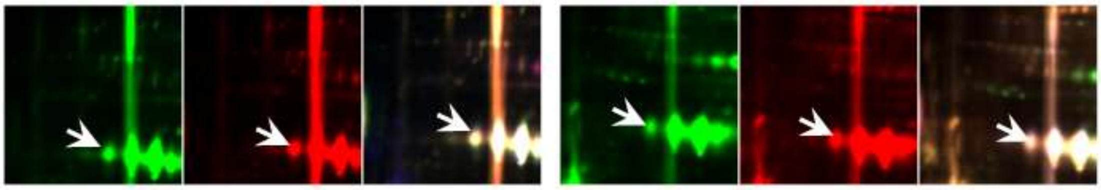

Accession No.: **scaffold0560_404767.mRNA1**

Plant species: ***Hevea brasiliensis***

Protein name: **Metacaspase-4**

Peptide sequences: **R.SAEPGDLLFVHYSGHGTR.L; R.EFVDQVPHGCR.I; K.QTIQDAFESR.G; R.GVHLPSDLHHHHGHR.D; K.LRPTLFDMFGDDASPK.V**

PFF Mascot score: **[346]** Sequence coverage %: **[16]**

Matched peptides No.: **[5]**

Calculated Mr: **46443** Calculated *p*I: **5.01**

Annotated PFF **spectra:**

Ions score is -10*Log(P), where P is the probability that the observed match is a random event.
Individual ions scores > 31 indicate identity or extensive homology (p<0.05).
Protein scores are derived from ions scores as a non-probabilistic basis for ranking protein hits.

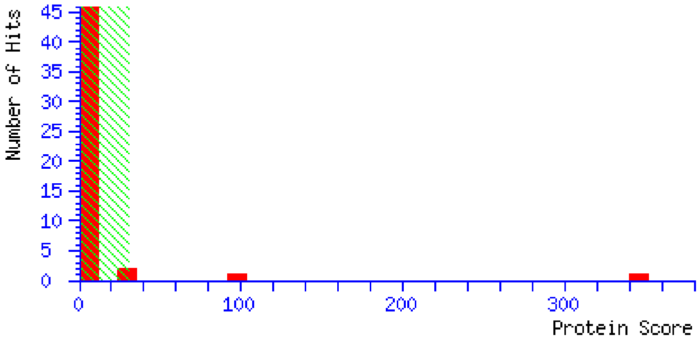

Matched peptide sequences: shown in **Bold Red**

1 MAKKAVLIGI NYPGTKAELK GCINDVKRMY RCLVDRYGFS EEDITVLIDI
51 DESYIQPTGK NIRRVLTDLV R**SAEPGDLLF VHYSGHGTR**L PAETGEDDDT
101 GFDECIVPCD MNLITDDDFR **EFVDQVPHGC R**ITVVSDSCH SGGLIDEAKE
151 QIGESTKRKE EESESGFGFK SFLK**QTIQDA FESRGVHLPS DLHHHHGHR**D
201 EEDFDNRVVE EDYGDSGYVK SKSLPLSTLI EILKQKTGKD DIDVGK**LRPT**
251 **LFDMFGDDAS PK**VKKFMKVI LNKLRHGDGE SGGGGFLGMV GSLAQEFLKH
301 KLDENDESYV KPALETEVDS KQEVYAGKTK RSLPDGGILI SGCQTDQTSA
351 DASPSGKSSE AYGALSNAIQ TIIAETDGAV TNQELVLKAR KMLKKQGFTQ
401 KPGLYCSDDH VEASFVC

Matched peptide information:

Start - End	Observed	Mr(expt)	Mr(calc)	ppm	Miss	Sequence
72 - 89	1942.8282	1941.8209	1941.9333	-58	0	R.SAEPGDLLFVHYSGHGTR.L (Ions score 125)
121 - 131	1343.5459	1342.5386	1342.6088	-52	0	R.EFVDQVPHGCR.I (Ions score 67)
175 - 184	1194.5095	1193.5022	1193.5677	-55	0	K.QTIQDAFESR.G (Ions score 75)
185 - 199	1735.7604	1734.7531	1734.8564	-60	0	R.GVHLPSDLHHHHGHR.D (Ions score 47)
247 - 262	1809.7789	1808.7716	1808.8767	-58	1	K.LRPTLFDMFGDDASPK.V (Ions score 32)

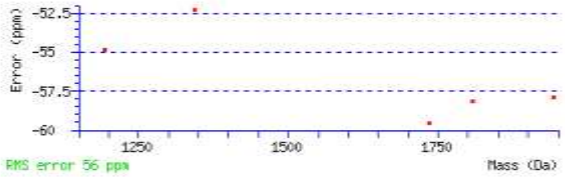

Spot No.: **61**

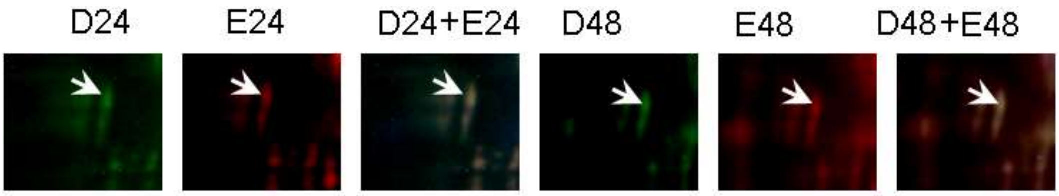

Accession No.: **scaffold0233_1484662.mRNA1**

Plant species: ***Hevea brasiliensis***

Protein name： **26S proteasome non-ATPase regulatory subunit 4 homolog**

Peptide sequences: **M.VLEATMICIDNSEWMR.N; R.FQAQADAVNLICGAK.T; R.IIVFAGSPIK.H; R.NDLTDEENALLQQALAMSMDEPASSHELR.D**

PFF Mascot score: **[231]**

Sequence coverage %: **[17]**

Matched peptides No.: **[4]**

Calculated Mr: **42727**

Calculated *p*I: **4.57**

Annotated PFF **spectra:**

Ions score is -10*Log(P), where P is the probability that the observed match is a random event.
Individual ions scores > 31 indicate identity or extensive homology (p<0.05).
Protein scores are derived from ions scores as a non-probabilistic basis for ranking protein hits.

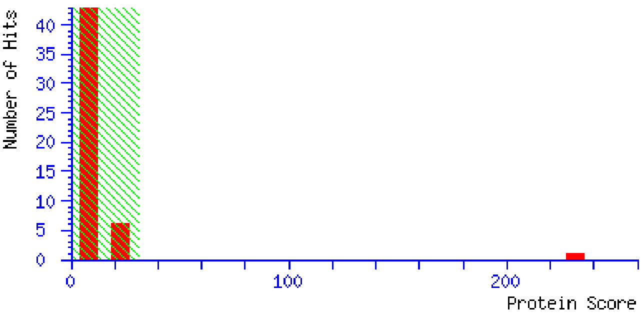

Matched peptide sequences: shown in **Bold Red**

```
  1 MVLEATMICI DNSEWMRNGD YSPSRFQAQA DAVNLICGAK TQSNPENTVG
 51 VLTMAGKGVR VLVTPTSDLG KILACMHGLE IGGEMNLAAG IQVAQLALKH
101 RQNKKQQQRI IVFAGSPIKH DKKTLEMIGR KLKKSNVALD IVDFGEEEDG
151 KAEKLEALLA AVNTNDTSHI VHVPPGPNAL SDVLISTPIF TGDGEGGSGF
201 AAAAAAAAAG GVSGFEFGVD PNLDPELALA LRVSMEEERA RQEAAAKKAA
251 EEGSKQEKGG EQPSSSQDAT MTESASVAAS EADNKRNDLT DEENALLQQA
301 LAMSMDEPAS SHELRDTDMS EAAADDPDLA LALQLSVQDS TKDSGSQTDM
351 SKLLADQSFV SSILASLPGV DPNDPSVKDL LASMQSQSEP PEKKDEDKPK
401 EEK
```

Bold red residues: 2–17 (VLEATMICIDNSEWMR), 26–40 (FQAQADAVNLICGAK), 110–119 (IIVFAGSPIK), 287–315 (NDLTDEENALLQQALAMSMDEPASSHELR).

Matched peptide information:

Start - End	Observed	Mr(expt)	Mr(calc)	ppm	Miss	Sequence
2 - 17	1967.8317	1966.8244	1966.8951	-36	0	M.VLEATMICIDNSEWMR.N (Ions score 72)
26 - 40	1605.7445	1604.7372	1604.7981	-38	0	R.FQAQADAVNLICGAK.T (Ions score 80)
110 - 119	1044.6050	1043.5977	1043.6379	-39	0	R.IIVFAGSPIK.H (Ions score 47)
287 - 315	3228.3879	3227.3806	3227.4710	-28	0	R.NDLTDEENALLQQALAMSMDEPASSHELR.D (Ions score 33)

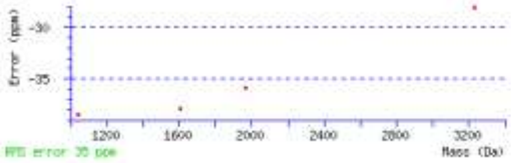

Spot No.: **62**

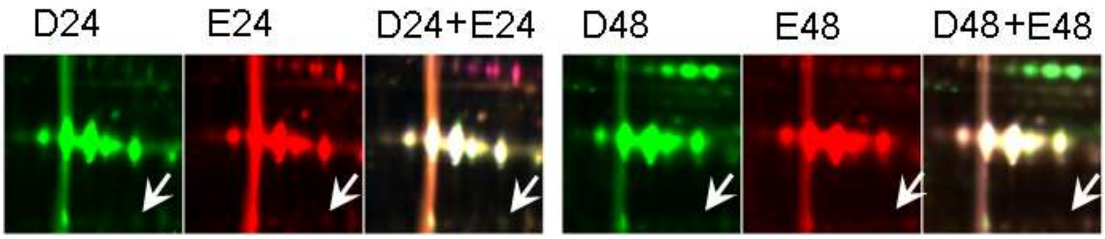

Accession No.: **scaffold0677_45450.mRNA1**

Plant species: ***Hevea brasiliensis***

Protein name：**Probable carboxylesterase 6**

Peptide sequences: **K.LVEEVSGWLR.L; R.LYDDGSVDR.T; K.FMADPVPAHQDFIDGVATR.D; R.LPAACDDGFSALLWLR.S; R.SSEPWLNDYADFNR.V; R.VFLIGDSSGGNLVHEVAAR.A; R.LAGGIPVHPGFVR.S**

PFF Mascot score: **[677]** Sequence coverage %: **[29]**

Matched peptides No.: **[7]**

Calculated Mr: **37612** Calculated *p*I: **5.02**

Annotated PFF spectra:

Ions score is -10*Log(P), where P is the probability that the observed match is a random event.
Individual ions scores > 31 indicate identity or extensive homology (p<0.05).
Protein scores are derived from ions scores as a non-probabilistic basis for ranking protein hits.

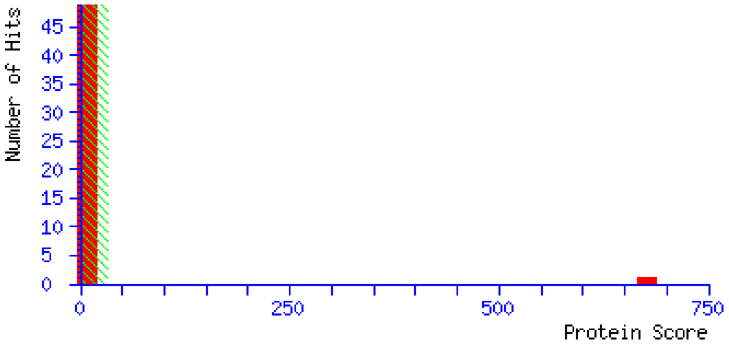

Matched peptide sequences: shown in **Bold Red**

```
  1 MVRERK**LVEE VSGWLRLYDD GSVDR**TWTGS PQVK**FMADPV PAHQDFIDGV**
 51 **ATR**DVTIDEN SGLRVRIYLP EPNSEDLNKL PVILHFHGGG FCISQADWYM
101 YYNIYTRLAK SVRAICVSVY LRLAPEHR**LP AACDDGFSAL LWLR**SLAQGR
151 **SSEPWLNDYA DFNRVFLIGD SSGGNLVHEV AAR**AGNVDLS PLR**LAGGIPV**
201 **HPGFVR**SERS KSEMEQPESP FLTLDMVDKF LGLALPVGCT KDHPITCPMG
251 TAAPQLDSLN LPPLLLCVAE MDLIKDTEME YYEATKKANK DVELLISPGM
301 GHSFYLNKIA LDMDPPTAEQ TTALIEGIQE FINKH
```

Matched peptide information:

Start - End	Observed	Mr(expt)	Mr(calc)	ppm	Miss	Sequence
7 - 16	1187.5850	1186.5777	1186.6346	-48	0	K.LVEEVSGWLR.L (Ions score 48)
17 - 25	1039.4208	1038.4135	1038.4618	-46	0	R.LYDDGSVDR.T (Ions score 63)
35 - 53	2086.8975	2085.8902	2085.9942	-50	0	K.FMADPVPAHQDFIDGVATR.D (Ions score 108)
129 - 144	1804.8143	1803.8070	1803.8978	-50	0	R.LPAACDDGFSALLWLR.S (Ions score 125)
151 - 164	1713.6616	1712.6543	1712.7430	-52	0	R.SSEPWLNDYADFNR.V (Ions score 98)
165 - 183	1940.9210	1939.9137	1940.0116	-50	0	R.VFLIGDSSGGNLVHEVAAR.A (Ions score 154)
194 - 206	1319.6923	1318.6850	1318.7510	-50	0	R.LAGGIPVHPGFVR.S (Ions score 81)

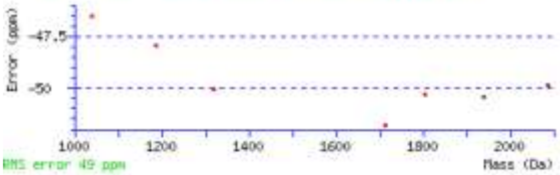

Spot No.: **63**

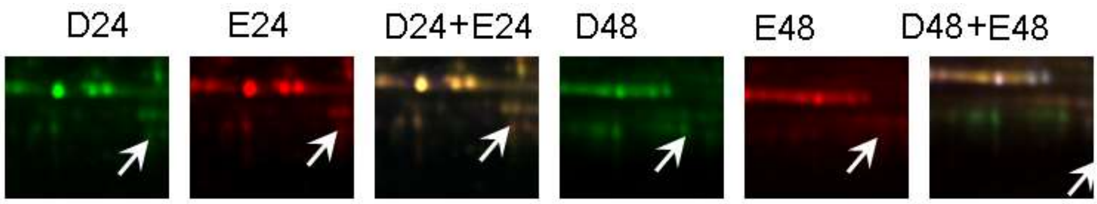

Accession No.: **scaffold0347_688965.mRNA1**

Plant species: ***Hevea brasiliensis***

Protein name： **Annexin D4**

Peptide sequences: **R.STHFFIEDER.S; R.FENALVLWAMHPWER.D; R.LLHEALTLVPQSYR.V; R.VILEIACTR.S; R.KAYHSLYDHSIEEDVAIHVTGSER.K; K.AYHSLYDHSIEEDVAIHVTGSER.K; K.LLVALVSAYR.Y; K.ETAECLCNPHAYFSK.V; K.EIKEEYNSLYGVPLTQK.I; K.DLLLALMTR.D**

PFF Mascot score: **[523]**

Sequence coverage %: **[36]**

Matched peptides No.: **[10]**

Calculated Mr: **39535**

Calculated *p*I: **7.72**

Annotated PFF **spectra:**

Ions score is -10*Log(P), where P is the probability that the observed match is a random event.
Individual ions scores > 31 indicate identity or extensive homology (p<0.05).
Protein scores are derived from ions scores as a non-probabilistic basis for ranking protein hits.

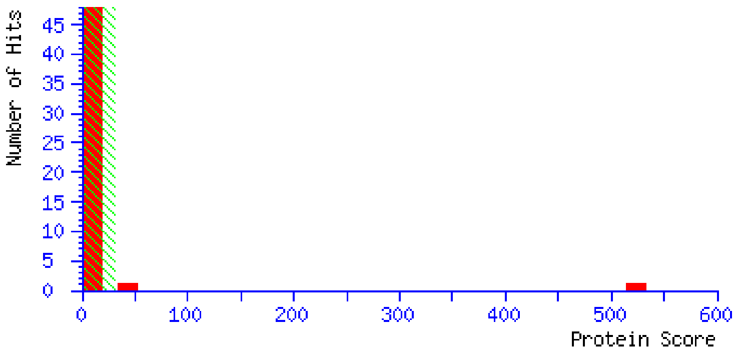

Matched peptide sequences: shown in **Bold Red**

```
  1 MTIITFLFYK YSSISFSLLQ NTKKKTWLIF KNWKLSPKLS QDLELNEKSL
 51 ISILGKSDPA RRTTFRQRST HFFIEDERSF ERWDDHRINL LRLEFVRFEN
101 ALVLWAMHPW ERDARLLHEA LTLVPQSYRV ILEIACTRSS EELLGARKAY
151 HSLYDHSIEE DVAIHVTGSE RKLLVALVSA YRYEGPKVSE DAAKSEAKLI
201 AKAIKNGDKK NPIDDDEVIR ILTTRSKPHL KAIYKHYKEV SGKNINEDIE
251 AADFILKETA ECLCNPHAYF SKVVDEAIRN DADHNTKKAL TRVIVTRADV
301 DLKEIKEEYN SLYGVPLTQK IDDNANGNYK DLLLALMTRD
```

Matched peptide information:

Start - End	Observed	Mr(expt)	Mr(calc)	ppm	Miss	Sequence
69 - 78	1280.5591	1279.5518	1279.5833	-25	0	R.STHFFIEDER.S (Ions score 32)
98 - 112	1898.8768	1897.8695	1897.9297	-32	0	R.FENALVLWAMHPWER.D (Ions score 67)
116 - 129	1639.8740	1638.8667	1638.9093	-26	0	R.LLHEALTLVPQSYR.V (Ions score 114)
130 - 138	1074.5660	1073.5587	1073.5903	-29	0	R.VILEIACTR.S (Ions score 43)
148 - 171	2756.2407	2755.2334	2755.3201	-31	1	R.KAYHSLYDHSIEEDVAIHVTGSER.K (Ions score 75)
149 - 171	2628.1426	2627.1353	2627.2252	-34	0	K.AYHSLYDHSIEEDVAIHVTGSER.K (Ions score 68)
173 - 182	1104.6500	1103.6427	1103.6703	-25	0	K.LLVALVSAYR.Y (Ions score 51)
258 - 272	1826.7563	1825.7490	1825.7763	-15	0	K.ETAECLCNPHAYFSK.V (Ions score 12)
304 - 320	2010.9761	2009.9688	2010.0309	-31	1	K.EIKEEYNSLYGVPLTQK.I (Ions score 27)
331 - 339	1045.5753	1044.5680	1044.6001	-31	0	K.DLLLALMTR.D (Ions score 36)

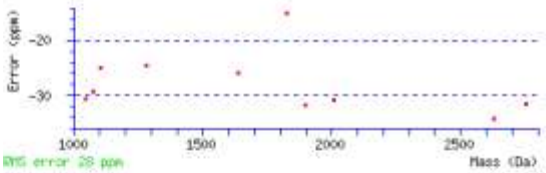

Spot No.: **64**

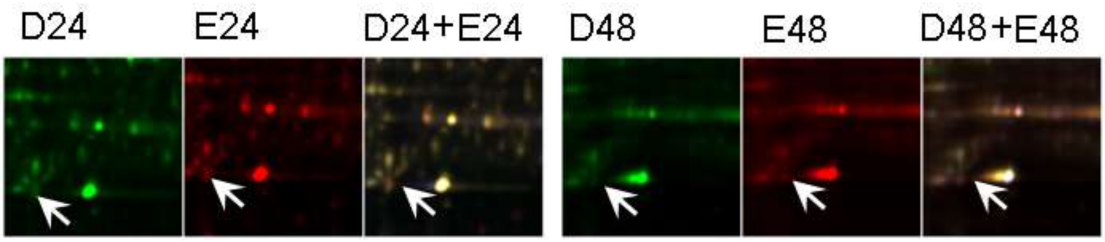

Accession No.: **scaffold0823_158984.mRNA1**

Plant species: ***Hevea brasiliensis***

Protein name：**Isoflavone reductase-like protein**

Peptide sequences: **K.NLGVTILPGDLYDHESLVK.A; K.RFFPSEFGNDVDHVHAVEPAK.S; R.FFPSEFGNDVDHVHAVEPAK.S; R.AIEAAGIPYTYVPSNFFASLIIR.I**

PFF Mascot score: **[380]**

Sequence coverage %: **[20]**

Matched peptides No.: **[4]**

Calculated Mr: **33240**

Calculated *p*I: **5.50**

Annotated PFF spectra:

Ions score is -10*Log(P), where P is the probability that the observed match is a random event.
Individual ions scores > 30 indicate identity or extensive homology (p<0.05).
Protein scores are derived from ions scores as a non-probabilistic basis for ranking protein hits.

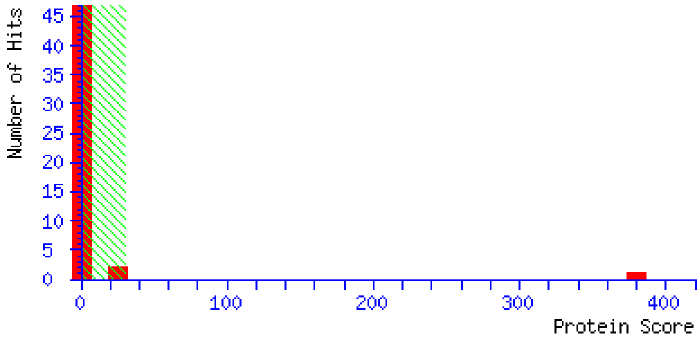

Matched peptide sequences: shown in **Bold Red**

```
  1 MADKSKILIV GGTGYFGKFV VEASARAGHP TFALARESTV SDPVKGKIIE
 51 NFKNLGVTIL PGDLYDHESL VKAIKQVDVV ISTVGALQVA DQTKIIAAIN
101 EAGNIKRFFP SEFGNDVDHV HAVEPAKSAF ETKAQIRRAI EAAGIPYTYV
151 PSNFFASLII RILLQPGSNK VTIQGDGNVK AVFNKEDDIA TYTIKAVDDP
201 RTLNKTLLIK PPNNVYTFNE LVALWEKKLG KILEKTYVPE EQLLKDIQAA
251 PIPFNVGLAI NHSVFIKGDQ TNFEIEPSWG VEASELYPDV KYSTVDELLD
301 LFV
```

Bold red residues: 54–72 (NLGVTIL PGDLYDHESL VK), 107–127 (RFFP SEFGNDVDHV HAVEPAK), 139–161 (AI EAAGIPYTYV PSNFFASLII R)

Matched peptide information:

Start - End	Observed	Mr(expt)	Mr(calc)	ppm	Miss	Sequence
54 - 72	2082.9958	2081.9885	2082.0997	-53	0	K.NLGVTILPGDLYDHESLVK.A (Ions score 126)
107 - 127	2398.0317	2397.0244	2397.1502	-52	1	K.RFFPSEFGNDVDHVHAVEPAK.S (Ions score 49)
108 - 127	2241.9399	2240.9326	2241.0491	-52	0	R.FFPSEFGNDVDHVHAVEPAK.S (Ions score 99)
139 - 161	2513.2048	2512.1975	2512.3366	-55	0	R.AIEAAGIPYTYVPSNFFASLIIR.I (Ions score 106)

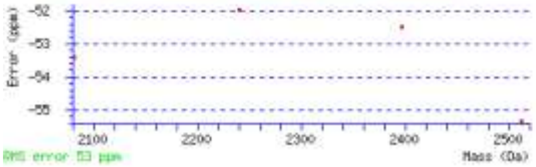

Spot No.: **65**

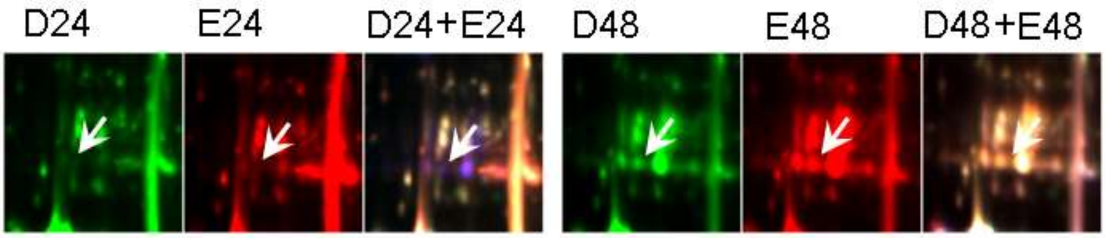

Accession No.: **scaffold0677_448742.mRNA1**

Plant species: ***Hevea brasiliensis***

Protein name：**Proteasome subunit alpha type-5**

Peptide sequences: **R.GVNTFSPEGR.L; R.LFQVEYAIEAIK.L; R.FSYGEPMTVESTTQALCDLALR.F; K.AIGSGSEGADSSLQEQYNKDLTLQEAETIALSILK.Q; K.DLTLQEAETIALSILK.Q; K.VAPTYHLYTPAEVETVISR.L; K.VAPTYHLYTPAEVETVISRL.-**

PFF Mascot score: **[609]** Sequence coverage %: **[41]**

Matched peptides No.: **[7]**

Calculated Mr: **26165** Calculated *p*I: **4.70**

Annotated PFF **spectra:**

Ions score is -10*Log(P), where P is the probability that the observed match is a random event.
Individual ions scores > 31 indicate identity or extensive homology (p<0.05).
Protein scores are derived from ions scores as a non-probabilistic basis for ranking protein hits.

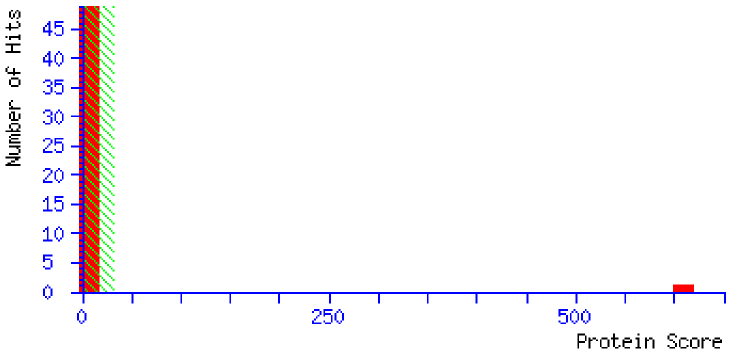

Matched peptide sequences: shown in Bold Red

```
  1 MFLTRTEYDR GVNTFSPEGR LFQVEYAIEA IKLGSTAIGL KTKEGVVLAV
 51 EKRITSPLLE PSSVEKVMEI DEHIGCAMSG LIADARTLVE HARVETQNHR
101 FSYGEPMTVE STTQALCDLA LRFGEGDEES MSRPFGVSLL IAGHDENGPS
151 LYYTDPSGTF WQCNAKAIGS GSEGADSSLQ EQYNKDLTLQ EAETIALSIL
201 KQVMEEKVTP NNVDIAKVAP TYHLYTPAEV ETVISRL
```

Bold red segments: 1 MFLTRTEYDR **GVNTFSPEGR LFQVEYAIEA IK**LGSTAIGL KTKEGVVLAV; 101 **FSYGEPMTVE STTQALCDLA LR**FGEGDEES MSRPFGVSLL IAGHDENGPS; 151 LYYTDPSGTF WQCNAK**AIGS GSEGADSSLQ EQYNKDLTLQ EAETIALSIL**; 201 **K**QVMEEKVTP NNVDIAK**VAP TYHLYTPAEV ETVISRL**

Matched peptide information:

Start - End	Observed	Mr(expt)	Mr(calc)	ppm	Miss	Sequence
11 - 20	1063.4688	1062.4615	1062.5094	-45	0	R.GVNTFSPEGR.L (Ions score 31)
21 - 32	1423.7102	1422.7029	1422.7758	-51	0	R.LFQVEYAIEAIK.L (Ions score 94)
101 - 122	2489.0378	2488.0305	2488.1614	-53	0	R.FSYGEPMTVESTTQALCDLALR.F (Ions score 146)
167 - 201	3679.6880	3678.6807	3678.8475	-45	1	K.AIGSGSEGADSSLQEQYNKDLTLQEAETIALSILK.Q (Ions score 14)
186 - 201	1757.8966	1756.8893	1756.9822	-53	0	K.DLTLQEAETIALSILK.Q (Ions score 82)
218 - 236	2146.0105	2145.0032	2145.1106	-50	0	K.VAPTYHLYTPAEVETVISR.L (Ions score 132)
218 - 237	2259.0830	2258.0757	2258.1947	-53	1	K.VAPTYHLYTPAEVETVISRL.- (Ions score 110)

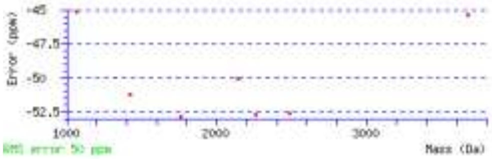

Spot No.: **66**

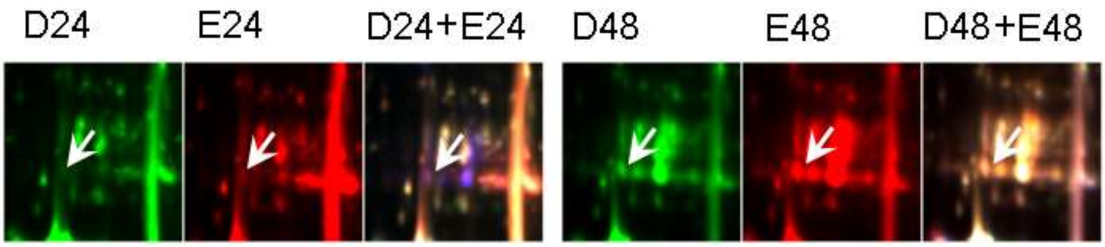

Accession No.: **scaffold1222_60641.mRNA1**

Plant species: ***Hevea brasiliensis***

Protein name： **Small rubber particle protein**

Peptide sequences: **K.TVVTPVYYIPLEAVK.F; K.QVSAQTYSVAQDAPR.I; R.IVLDVASSVFNTGVQEGAK.A; K.AEQYAVITWR.A; R.ALNKLPLVPQVANVVVPTAVYFSEK.Y**

PFF Mascot score: **[532]** Sequence coverage %: **[41]**

Matched peptides No.: **[5]**

Calculated Mr: **22331** Calculated *p*I: **4.80**

Annotated PFF **spectra:**

Ions score is -10*Log(P), where P is the probability that the observed match is a random event.
Individual ions scores > 31 indicate identity or extensive homology (p<0.05).
Protein scores are derived from ions scores as a non-probabilistic basis for ranking protein hits.

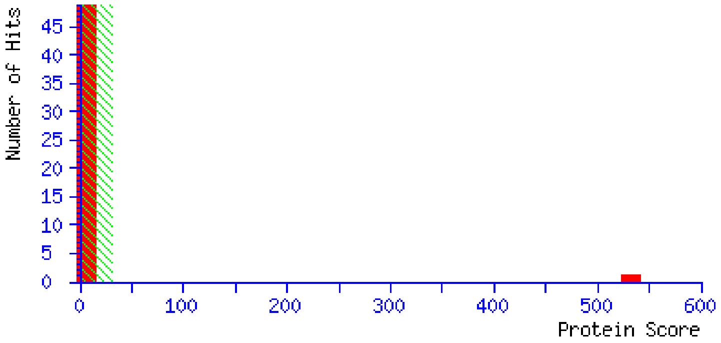

Matched peptide sequences: shown in **Bold Red**

1 MAEEVEEERL KYLDFVRAAG VYAVDSFSTL YLYAKDISGP LKPGVDTIEN
51 VVK**TVVTPVY YIPLEAVK**FV DKTVDVSVTS LDGVVPPVIK **QVSAQTYSVA**
101 **QDAPRIVLDV ASSVFNTGVQ EGAK**ALYANL EPK**AEQYAVI TWRALNKLPL**
151 **VPQVANVVVP TAVYFSEK**YN DVVRGTTEQG YRVSSYLPLL PTEKITKVFG
201 DEAS

Matched peptide information:

Start - End	Observed	Mr(expt)	Mr(calc)	ppm	Miss	Sequence
54 - 68	1691.8862	1690.8789	1690.9546	-45	0	K.TVVTPVYYIPLEAVK.F (Ions score 64)
91 - 105	1620.7314	1619.7241	1619.7903	-41	0	K.QVSAQTYSVAQDAPR.I (Ions score 118)
106 - 124	1933.9448	1932.9375	1933.0157	-40	0	R.IVLDVASSVFNTGVQEGAK.A (Ions score 153)
134 - 143	1236.5956	1235.5883	1235.6299	-34	0	K.AEQYAVITWR.A (Ions score 66)
144 - 168	2696.4307	2695.4234	2695.5313	-40	1	R.ALNKLPLVPQVANVVVPTAVYFSEK.Y (Ions score 110)

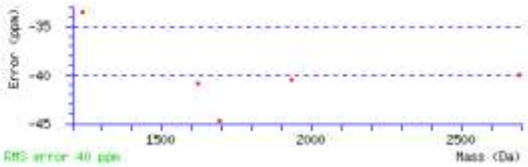

Spot No.: **67**

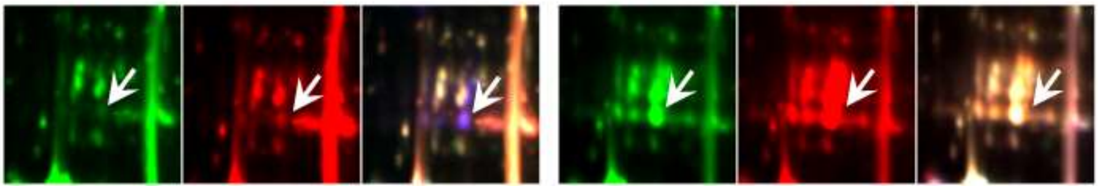

Accession No.: **scaffold0814_47376.mRNA1**

Plant species: ***Hevea brasiliensis***

Protein name: **14-3-3-like protein**

Peptide sequences: **K.LAEQAERYEEMVEFMEK.V; K.TVDVEELTVEER.N; K.ICDGILSLLESHLIPSASSAESK.V; K.SAQDIALADLAPTHPIR.L; K.DSTLIMQLLR.D**

PFF Mascot score: **[396]**

Sequence coverage %: **[29]**

Matched peptides No.: **[5]**

Calculated Mr: **29931**

Calculated *p*I: **4.76**

Annotated PFF **spectra:**

Ions score is -10*Log(P), where P is the probability that the observed match is a random event.
Individual ions scores > 30 indicate identity or extensive homology (p<0.05).
Protein scores are derived from ions scores as a non-probabilistic basis for ranking protein hits.

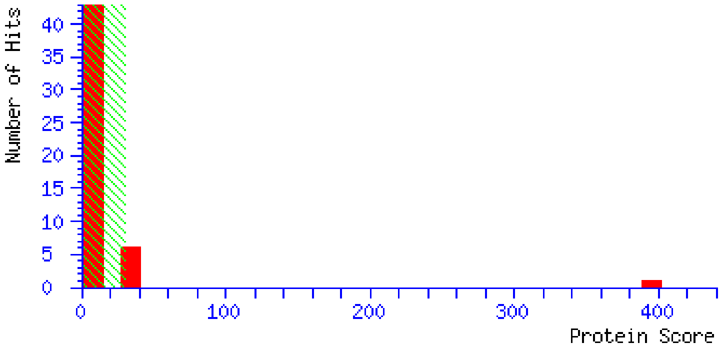

Matched peptide sequences: shown in Bold Red

1 MSPTESSREE NVYMAK**LAEQ AERYEEMVEF MEK**VAK**TVDV EELTVEER**NL
51 LSVAYKNVIG ARRASWRIIS SIEQKEESRG NEDHVTIIKE YRGKIESELS
101 K**ICDGILSLL ESHLIPSASS AESK**VFYLKM KGDYHRYLAE FKTAGERKEA
151 AESTLLAYK**S AQDIALADLA PTHPIR**LGLA LNFSVFYYEI LNSPDRACNL
201 AKQAFDEAIS ELDTLGEESY K**DSTLIMQLL R**DNLTLWTSD ITDEAGDEIK
251 EASKRESGEG QPQQ

Matched peptide information:

Start - End	Observed	Mr(expt)	Mr(calc)	ppm	Miss	Sequence
17 - 33	2131.9172	2130.9099	2130.9601	-24	1	K.LAEQAERYEEMVEFMEK.V (Ions score 41)
37 - 48	1418.6754	1417.6681	1417.6936	-18	0	K.TVDVEELTVEER.N (Ions score 101)
102 - 124	2427.1836	2426.1763	2426.2363	-25	0	K.ICDGILSLLESHLIPSASSAESK.V (Ions score 66)
160 - 176	1788.9161	1787.9088	1787.9530	-25	0	K.SAQDIALADLAPTHPIR.L (Ions score 109)
222 - 231	1189.6407	1188.6334	1188.6536	-17	0	K.DSTLIMQLLR.D (Ions score 80)

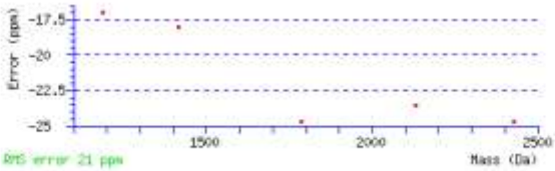

Spot No.: **68**

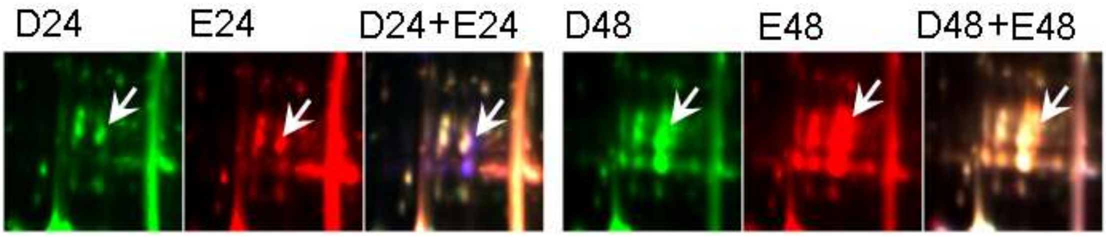

Accession No.: **scaffold0814_47376.mRNA1**

Plant species: ***Hevea brasiliensis***

Protein name: **14-3-3-like protein**

Peptide sequences: **M.SPTESSREENVYMAK.L; K.LAEQAERYEEMVEFMEK.V; K.TVDVEELTVEER.N; K.ICDGILSLLESHLIPSASSAESK.V; K.SAQDIALADLAPTHPIR.L; R.LGLALNFSVFYYEILNSPDR.A; K.QAFDEAISELDTLGEESYKDSTLIMQLLR.D; K.DSTLIMQLLR.D**

PFF Mascot score: **[821]** Sequence coverage %: **[50]**

Matched peptides No.: **[8]**

Calculated Mr: **29931** Calculated *p*I: **4.76**

Annotated PFF **spectra:**

Ions score is -10*Log(P), where P is the probability that the observed match is a random event.
Individual ions scores > 30 indicate identity or extensive homology (p<0.05).
Protein scores are derived from ions scores as a non-probabilistic basis for ranking protein hits.

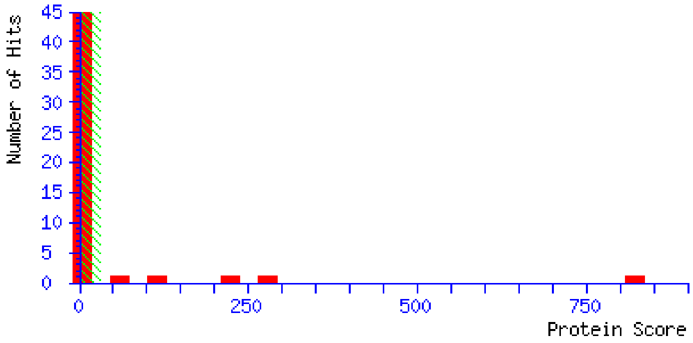

Matched peptide sequences: shown in Bold Red

```
  1 MSPTESSREE NVYMAKLAEQ AERYEEMVEF MEKVAKTVDV EELTVEERNL
 51 LSVAYKNVIG ARRASWRIIS SIEQKEESRG NEDHVTIIKE YRGKIESELS
101 KICDGILSLL ESHLIPSASS AESKVFYLKM KGDYHRYLAE FKTAGERKEA
151 AESTLLAYKS AQDIALADLA PTHPIRLGLA LNFSVFYYEI LNSPDRACNL
201 AKQAFDEAIS ELDTLGEESY KDSTLIMQLL RDNLTLWTSD ITDEAGDEIK
251 EASKRESGEG QPQQ
```

Matched peptide information:

Start - End	Observed	Mr(expt)	Mr(calc)	ppm	Miss	Sequence
2 - 16	1727.7111	1726.7038	1726.7832	-46	1	M.SPTESSREENVYMAK.L (Ions score 89)
17 - 33	2131.8774	2130.8701	2130.9601	-42	1	K.LAEQAERYEEMVEFMEK.V (Ions score 70)
37 - 48	1418.6472	1417.6399	1417.6936	-38	0	K.TVDVEELTVEER.N (Ions score 110)
102 - 124	2427.1436	2426.1363	2426.2363	-41	0	K.ICDGILSLLESHLIPSASSAESK.V (Ions score 90)
160 - 176	1788.8860	1787.8787	1787.9530	-42	0	K.SAQDIALADLAPTHPIR.L (Ions score 132)
177 - 196	2331.0972	2330.0899	2330.1947	-45	0	R.LGLALNFSVFYYEILNSPDR.A (Ions score 70)
203 - 231	3315.4958	3314.4885	3314.6229	-40	1	K.QAFDEAISELDTLGEESYKDSTLIMQLLR.D (Ions score 174)
222 - 231	1189.6123	1188.6050	1188.6536	-41	0	K.DSTLIMQLLR.D (Ions score 88)

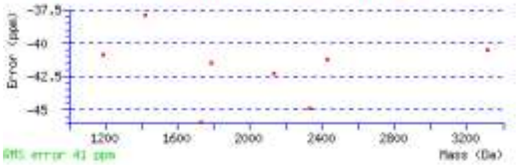

Spot No.: 69

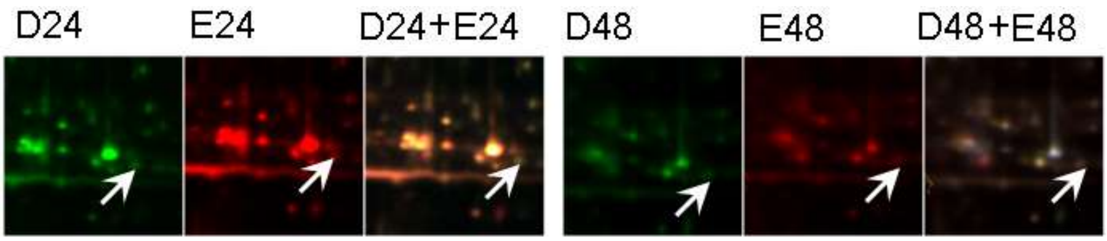

Accession No.: scaffold0127_20309.mRNA1

Plant species: *Hevea brasiliensis*

Protein name：Proteasome subunit beta type-6

Peptide sequences: R.TSTGMYVANR.A; K.ITQLTDNVYVCR.S; R.SGSAADSQIVSDYVR.Y; R.YFLHQHTIQLGQPATVK.V; K.NMLETGLIVGGWDKYEGGK.I; R.TVIINSEGVTR.N; K.LPLWHEELEPHNSLLDIINASGPEPMHM.-

PFF Mascot score: [663]   Sequence coverage %: [48]

Matched peptides No.: [7]

Calculated Mr: 24974   Calculated pI: 5.47

Annotated PFF **spectra:**

Ions score is -10*Log(P), where P is the probability that the observed match is a random event.
Individual ions scores > 30 indicate identity or extensive homology (p<0.05).
Protein scores are derived from ions scores as a non-probabilistic basis for ranking protein hits.

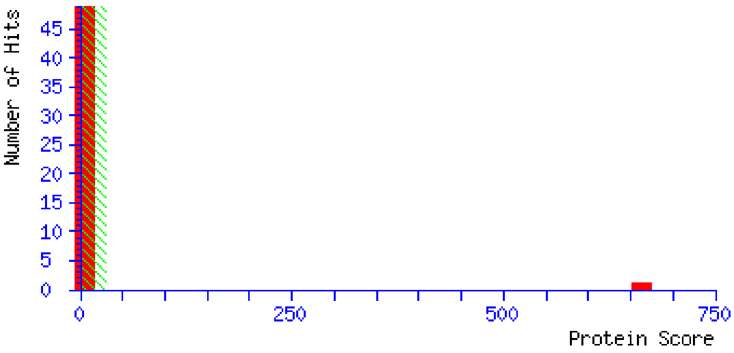

Matched peptide sequences: shown in **Bold Red**

```
  1 MDLKAPHSMG TTIIGVTYDG GVVLGADSRT STGMYVANRA SDKITQLTDN
 51 VYVCRSGSAA DSQIVSDYVR YFLHQHTIQL GQPATVKVAA NLVRLLSYNN
101 KNMLETGLIV GGWDKYEGGK IYGVPLGGTI IEQPFAIGGS GSSYLYGFFD
151 QAWKEGMTKD EAEQLVVKAV SLAMARDGAS GGVVRTVIIN SEGVTRNFYP
201 GDKLPLWHEE LEPHNSLLDI INASGPEPMH M
```

Matched peptide information:

Start - End	Observed	Mr(expt)	Mr(calc)	ppm	Miss	Sequence
30 - 39	1099.4885	1098.4812	1098.5128	-29	0	R.TSTGMYVANR.A (Ions score 77)
44 - 55	1481.7014	1480.6941	1480.7344	-27	0	K.ITQLTDNVYVCR.S (Ions score 113)
56 - 70	1554.7001	1553.6928	1553.7322	-25	0	R.SGSAADSQIVSDYVR.Y (Ions score 131)
71 - 87	1980.9983	1979.9910	1980.0581	-34	0	R.YFLHQHTIQLGQPATVK.V (Ions score 96)
102 - 120	2066.9509	2065.9436	2066.0143	-34	1	K.NMLETGLIVGGWDKYEGGK.I (Ions score 111)
186 - 196	1188.6257	1187.6184	1187.6510	-27	0	R.TVIINSEGVTR.N (Ions score 92)
204 - 231	3219.4785	3218.4712	3218.5528	-25	0	K.LPLWHEELEPHNSLLDIINASGPEPMHM.- (Ions score 42)

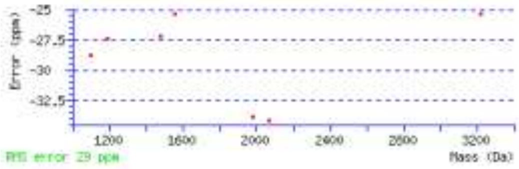

Spot No.: **70**

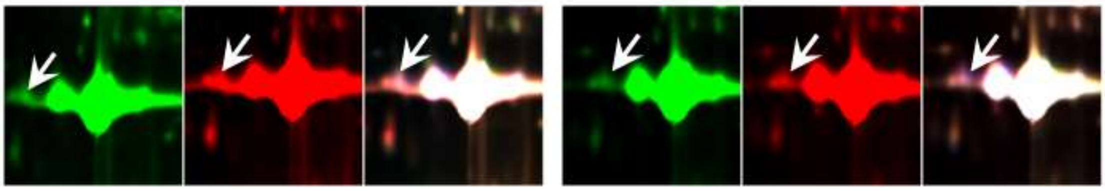

Accession No.: **scaffold1222_60641.mRNA1**

Plant species: ***Hevea brasiliensis***

Protein name： **Small rubber particle protein**

Peptide sequences: **K.DISGPLKPGVDTIENVVK.T; K.TVVTPVYYIPLEAVK.F; K.FVDKTVDVSVTSLDGVVPPVIK.Q; K.TVDVSVTSLDGVVPPVIK.Q; K.QVSAQTYSVAQDAPR.I; R.IVLDVASSVFNTGVQEGAK.A; K.ALYANLEPK.A; K.AEQYAVITWR.A; R.ALNKLPLVPQVANVVVPTAVYFSEK.Y; R.VSSYLPLLPTEK.I**

PFF Mascot score: **[830]**

Sequence coverage %: **[71]**

Matched peptides No.: **[10]**

Calculated Mr: **22331`**

Calculated *p*I: **4.80**

Annotated PFF **spectra:**

Ions score is -10*Log(P), where P is the probability that the observed match is a random event.
Individual ions scores > 31 indicate identity or extensive homology (p<0.05).
Protein scores are derived from ions scores as a non-probabilistic basis for ranking protein hits.

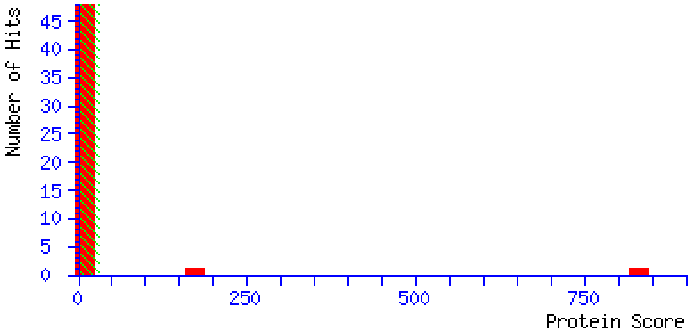

Matched peptide sequences: shown in Bold Red

```
  1 MAEEVEEERL KYLDFVRAAG VYAVDSFSTL YLYAKDISGP LKPGVDTIEN
 51 VVKTVVTPVY YIPLEAVKFV DKTVDVSVTS LDGVVPPVIK QVSAQTYSVA
101 QDAPRIVLDV ASSVFNTGVQ EGAKALYANL EPKAEQYAVI TWRALNKLPL
151 VPQVANVVVP TAVYFSEKYN DVVRGTTEQG YRVSSYLPLL PTEKITKVFG
201 DEAS
```

Matched peptide information:

Start - End	Observed	Mr(expt)	Mr(calc)	ppm	Miss	Sequence
36 - 53	1880.9950	1879.9877	1880.0255	-20	1	K.DISGPLKPGVDTIENVVK.T (Ions score 136)
54 - 68	1691.9015	1690.8942	1690.9546	-36	0	K.TVVTPVYYIPLEAVK.F (Ions score 76)
69 - 90	2314.2390	2313.2317	2313.2832	-22	1	K.FVDKTVDVSVTSLDGVVPPVIK.Q (Ions score 84)
73 - 90	1824.9690	1823.9617	1824.0245	-34	0	K.TVDVSVTSLDGVVPPVIK.Q (Ions score 34)
91 - 105	1620.7704	1619.7631	1619.7903	-17	0	K.QVSAQTYSVAQDAPR.I (Ions score 158)
106 - 124	1933.9744	1932.9671	1933.0157	-25	0	R.IVLDVASSVFNTGVQEGAK.A (Ions score 153)
125 - 133	1018.5144	1017.5071	1017.5495	-42	0	K.ALYANLEPK.A (Ions score 25)
134 - 143	1236.6156	1235.6083	1235.6299	-17	0	K.AEQYAVITWR.A (Ions score 70)
144 - 168	2696.4995	2695.4922	2695.5313	-14	1	R.ALNKLPLVPQVANVVVPTAVYFSEK.Y (Ions score 24)
183 - 194	1346.7030	1345.6957	1345.7493	-40	0	R.VSSYLPLLPTEK.I (Ions score 71)

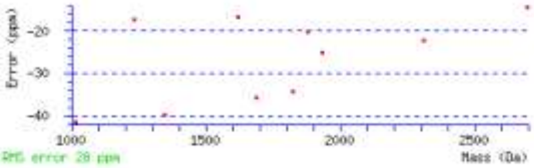

Spot No.: **71**

D24 E24 D24+E24 D48 E48 D48+E48

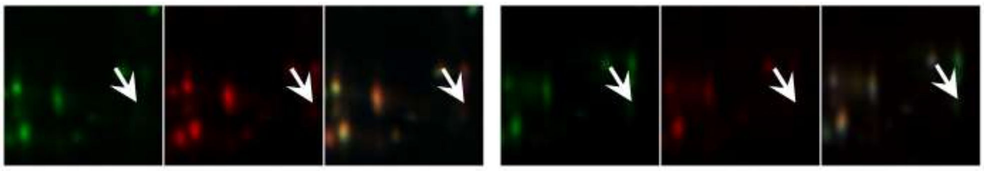

Accession No.: **scaffold0344_256841.mRNA1**

Plant species: ***Hevea brasiliensis***

Protein name： **18.5 kDa class I heat shock protein**

Peptide sequences: **K.DFPFPSFASSSSLFPR.E; K.ETPEAHVFK.A; R.VLQISGER.H; K.EDKNDTWHR.V**

PFF Mascot score: **[221]** Sequence coverage %: **[26]**

Matched peptides No.: **[4]**

Calculated Mr: **18064** Calculated *p*I: **5.97**

Annotated PFF **spectra:**

Ions score is -10*Log(P), where P is the probability that the observed match is a random event.
Individual ions scores > 31 indicate identity or extensive homology (p<0.05).
Protein scores are derived from ions scores as a non-probabilistic basis for ranking protein hits.

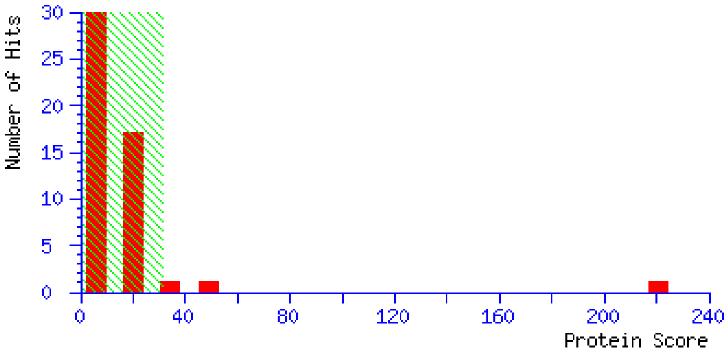

Matched peptide sequences: shown in **Bold Red**

```
  1 MSLTPFSGNR RSSIFDPFSL DIWDPFKDFP FPSFASSSSL FPRENSAFVS
 51 TRIDWKETPE AHVFKADLPG LRKEEVKVEI EDDRVLQISG ERHVEKEDKN
101 DTWHRVERSS GKFSRSFRLP ENTKMDQIKA SMENGVLTVT VPKAEVKKPD
151 VKAIEISG
```

Matched peptide information:

Start - End	Observed	Mr(expt)	Mr(calc)	ppm	Miss	Sequence	
28 - 43	1788.8492	1787.8419	1787.8519	-6	0	K.DFPFPSFASSSSLFPR.E	(Ions score 70)
57 - 65	1057.5239	1056.5166	1056.5240	-7	0	K.ETPEAHVFK.A	(Ions score 30)
85 - 92	901.5137	900.5065	900.5029	4	0	R.VLQISGER.H	(Ions score 49)
97 - 105	1200.5371	1199.5298	1199.5319	-2	1	K.EDKNDTWHR.V	(Ions score 72)

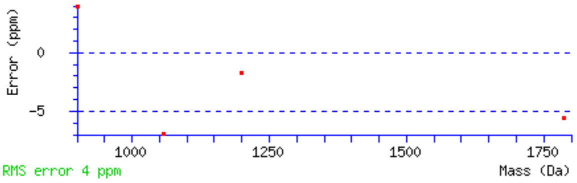

Spot No.: **72**

D24 E24 D24+E24 D48 E48 D48+E48

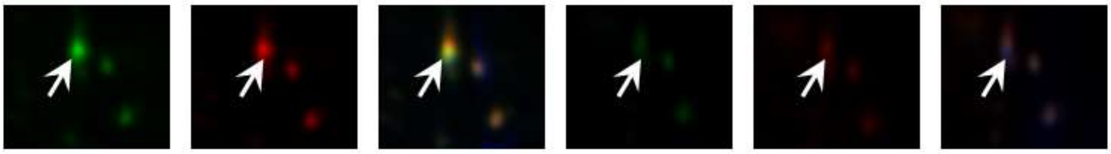

Accession No.: **scaffold0026_327123.mRNA1**

Plant species: ***Hevea brasiliensis***

Protein name： **Chlorophyllase type 0**

Peptide sequences: **K.EIEFAAEVGNWLLSGLQSVLPEK.V; R.GGNIAFALALGYSK.T; K.ISALVGLDPVGR.V**

PFF Mascot score: **[316]** Sequence coverage %: **[14]**

Matched peptides No.: **[3]**

Calculated Mr: **37785** Calculated *p*I: **6.30**

Annotated PFF **spectra:**

Ions score is -10*Log(P), where P is the probability that the observed match is a random event.
Individual ions scores > 30 indicate identity or extensive homology (p<0.05).
Protein scores are derived from ions scores as a non-probabilistic basis for ranking protein hits.

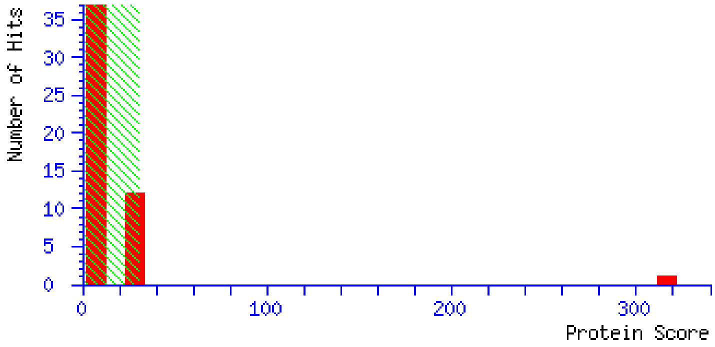

Matched peptide sequences: shown in Bold Red

```
  1 MLVTLLVILL ASALEAKPQF PTVVLLETKP VQDILDVFVT GSFPTKSIDV
 51 KKSNPASPPK PLLIVSPITD GTYPVFMFLH GTCLENYFYS NLLPHIASHG
101 FIVVAPQVYS CINWLIPKLP IRESKEIEFA AEVGNWLLSG LQSVLPEKVT
151 WDQDKLALGG HNRGGNIAFA LALGYSKTPL EVKISALVGL DPVGRVSTDP
201 KILTNVPHSF NLSIPVTVIG TGLGNESVCG VVGLACAPNY MNHVKFYNKC
251 KAPASHFVTT DYGHMDMLDD NPTGILAIIA NSICKNSKDP RDQMRRTVGG
301 LIVAFLKAYF QADSGDFMTI LNEPSVAPAK LDPVQFKEEQ NHAQV
```

Bold red residues: 126–148 (EIEFA AEVGNWLLSG LQSVLPEK), 164–177 (GGNIAFA LALGYSK), 184–195 (ISALVGL DPVGR).

Matched peptide information:

Start - End	Observed	Mr(expt)	Mr(calc)	ppm	Miss	Sequence
126 - 148	2529.2341	2528.2268	2528.3162	-35	0	K.EIEFAAEVGNWLLSGLQSVLPEK.V (Ions score 125)
164 - 177	1381.6929	1380.6856	1380.7401	-39	0	R.GGNIAFALALGYSK.T (Ions score 87)
184 - 195	1196.6589	1195.6516	1195.6925	-34	0	K.ISALVGLDPVGR.V (Ions score 105)

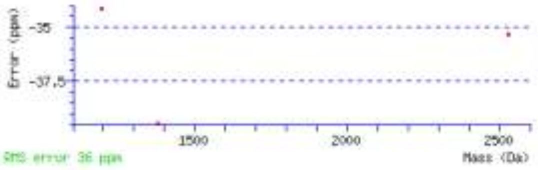

Spot No.: **73**

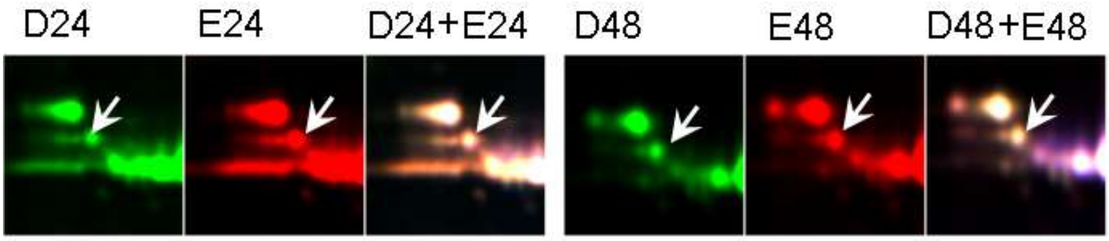

Accession No.: **scaffold1222_89338.mRNA1**

Plant species: ***Hevea brasiliensis***

Protein name: **Small rubber particle protein**

Peptide sequences: **K.YLEFVQATTDNAVTALSNIYLYAK.D; K.DNSGPLKPGVETIEGVAK.T; R.AVDASFTTLQNIVPSVLK.Q; K.QLPTQACDTSVK.E**

PFF Mascot score: **[381]**

Sequence coverage %: **[61]**

Matched peptides No.: **[4]**

Calculated Mr: **12769**

Calculated pI: **4.61**

Annotated PFF **spectra:**

Ions score is -10*Log(P), where P is the probability that the observed match is a random event.
Individual ions scores > 31 indicate identity or extensive homology ($p<0.05$).
Protein scores are derived from ions scores as a non-probabilistic basis for ranking protein hits.

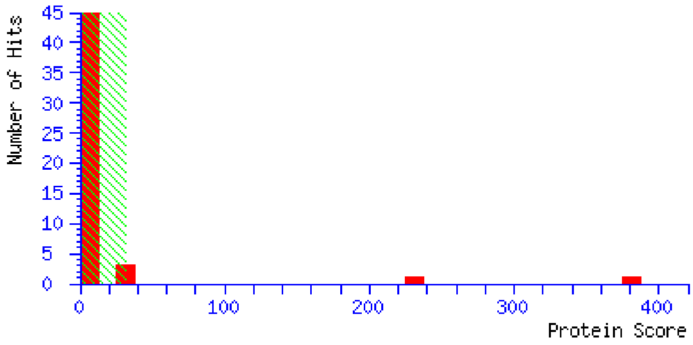

Matched peptide sequences: shown in **Bold Red**

```
  1 MAEGKENENF QQEANEQEEK LKYLEFVQAT TDNAVTALSN IYLYAKDNSG
 51 PLKPGVETIE GVAKTVVIPA SKIPTEAIKF ADRAVDASFT TLQNIVPSVL
101 KQLPTQACDT SVKESAE
```

Matched peptide information:

Start - End	Observed	Mr(expt)	Mr(calc)	ppm	Miss	Sequence
23 - 46	2708.3408	2707.3335	2707.3745	-15	0	K.YLEFVQATTDNAVTALSNIYLYAK.D (Ions score 27)
47 - 64	1810.9176	1809.9103	1809.9472	-20	1	K.DNSGPLKPGVETIEGVAK.T (Ions score 152)
84 - 101	1903.0002	1901.9929	1902.0462	-28	0	R.AVDASFTTLQNIVPSVLK.Q (Ions score 110)
102 - 113	1347.6136	1346.6063	1346.6500	-32	0	K.QLPTQACDTSVK.E (Ions score 93)

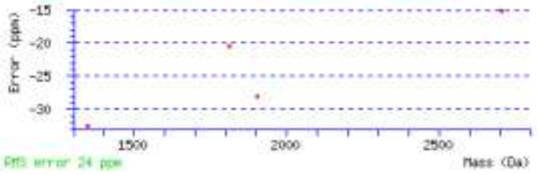

Spot No.: 74

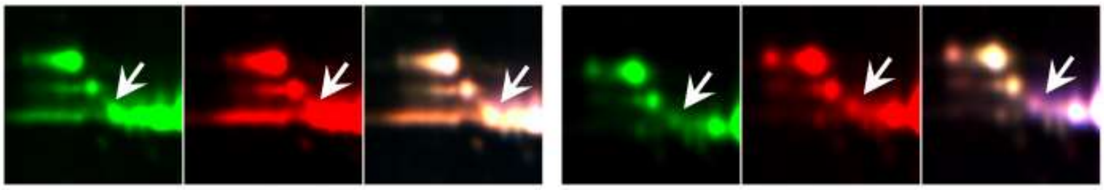

Accession No.: **scaffold1222_136753.mRNA1**

Plant species: ***Hevea brasiliensis***

Protein name：**Rubber elongation factor protein**

Peptide sequences: **K.DKSGPLQPGVDIIEGPVK.N; K.NVAVPLYNR.F; K.FVDSTVVASVTIIDR.S; K.DASIQVVSAIR.A; R.SLASSLPGQTK.I**

PFF Mascot score: **[451]** Sequence coverage %: **[46]**

Matched peptides No.: **[5]**

Calculated Mr: **14713** Calculated *p*I: **5.04**

Annotated PFF **spectra:**

Ions score is -10*Log(P), where P is the probability that the observed match is a random event.
Individual ions scores > 31 indicate identity or extensive homology (p<0.05).
Protein scores are derived from ions scores as a non-probabilistic basis for ranking protein hits.

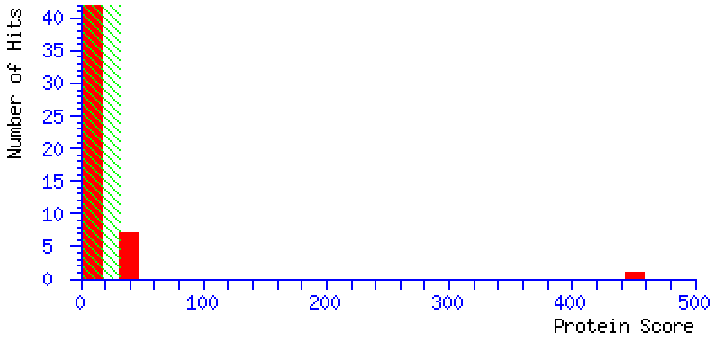

Matched peptide sequences: shown in Bold Red

```
  1 MAEDEDNQQG QGEGLKYLGF VQDAATYAVT TFSNVYLFAK DKSGPLQPGV
 51 DIIEGPVKNV AVPLYNRFSY IPNGALKFVD STVVASVTII DRSLPPIVKD
101 ASIQVVSAIR AAPEAARSLA SSLPGQTKIL AKVFYGEN
```

Matched peptide information:

Start - End	Observed	Mr(expt)	Mr(calc)	ppm	Miss	Sequence
41 - 58	1849.0270	1848.0197	1847.9993	11	1	K.DKSGPLQPGVDIIEGPVK.N (Ions score 129)
59 - 67	1045.5854	1044.5781	1044.5716	6	0	K.NVAVPLYNR.F (Ions score 39)
78 - 92	1621.8971	1620.8898	1620.8723	11	0	K.FVDSTVVASVTIIDR.S (Ions score 143)
100 - 110	1158.6569	1157.6496	1157.6404	8	0	K.DASIQVVSAIR.A (Ions score 103)
118 - 128	1088.5940	1087.5867	1087.5873	-1	0	R.SLASSLPGQTK.I (Ions score 38)

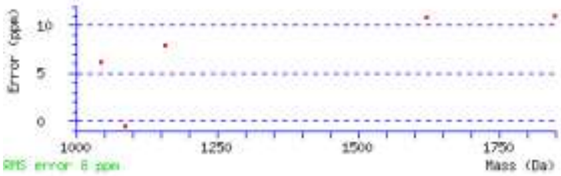

Spot No.: 75

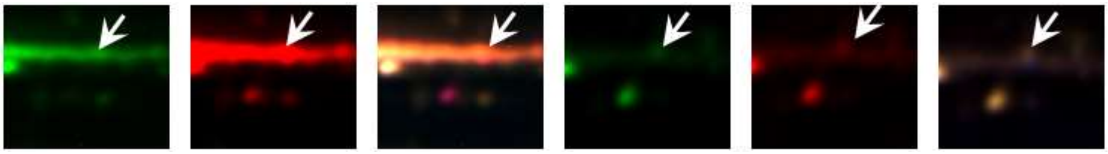

Accession No.: **scaffold0155_515853.mRNA1**

Plant species: ***Hevea brasiliensis***

Protein name: **Pro-hevein**

Peptide sequences: **K.YGWTAFCGPVGAHGQPSCGK.C; R.IVDQCSNGGLDLDVNVFR.Q; R.QLDTDGKGYER.G**

PFF Mascot score: **[221]** Sequence coverage %: **[23]**

Matched peptides No.: **[3]**

Calculated Mr: **23042** Calculated *p*I: **8.15**

Annotated PFF **spectra:**

Ions score is -10*Log(P), where P is the probability that the observed match is a random event.
Individual ions scores > 31 indicate identity or extensive homology (p<0.05).
Protein scores are derived from ions scores as a non-probabilistic basis for ranking protein hits.

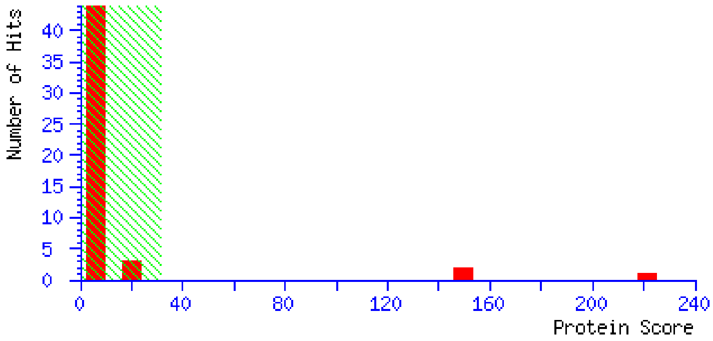

Matched peptide sequences: shown in Bold Red

```
  1 MGRVMNIFIV VLLCLTGVAI AEQCGRQAGG KLCPNNLCCS QYGWCGSSDD
 51 YCSPSKNCQS NCKGGGGGGG GGGGSASNVL ATYHLYNPQQ HGWDLNAVSA
101 YCSTWDANKP YSWRSKYGWT AFCGPVGAHG QPSCGKCLSV TNTGTGAKTT
151 VRIVDQCSNG GLDLDVNVFR QLDTDGKGYE RGHLTVNYQF VNCGDSFNPL
201 FSIMKSSVIN
```

Bold red: 117–136 (YGWT AFCGPVGAHG QPSCGK), 153–181 (IVDQCSNG GLDLDVNVFR QLDTDGKGYE R)

Matched peptide information:

Start - End	Observed	Mr(expt)	Mr(calc)	ppm	Miss	Sequence	
117 - 136	2136.8484	2135.8411	2135.9306	-42	0	K.YGWTAFCGPVGAHGQPSCGK.C	(Ions score 86)
153 - 170	2020.8995	2019.8922	2019.9684	-38	0	R.IVDQCSNGGLDLDVNVFR.Q	(Ions score 109)
171 - 181	1281.5601	1280.5528	1280.5997	-37	1	R.QLDTDGKGYER.G	(Ions score 25)

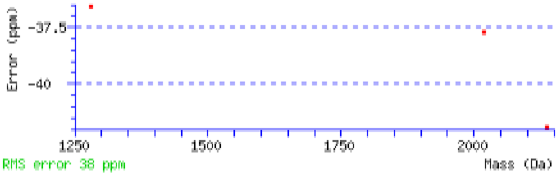

Spot No.: 76

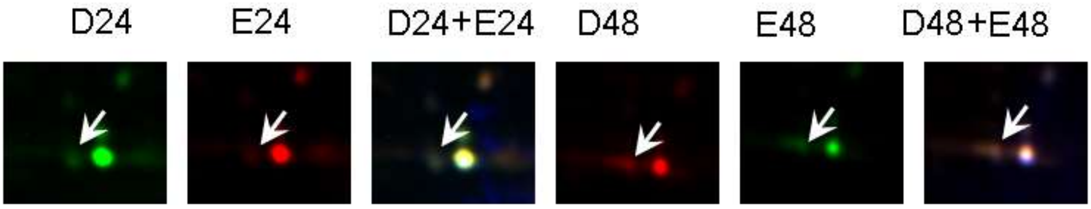

Accession No.: **scaffold0155_515853.mRNA1**

Plant species: ***Hevea brasiliensis***

Protein name： **Pro-hevein**

Peptide sequences: **K.YGWTAFCGPVGAHGQPSCGK.C; R.IVDQCSNGGLDLDVNVFR.Q**

PFF Mascot score: **[149]** Sequence coverage %: **[18]**

Matched peptides No.: **[2]**

Calculated Mr: **23042** Calculated *p*I: **8.15**

Annotated PFF **spectra:**

Ions score is -10*Log(P), where P is the probability that the observed match is a random event.
Individual ions scores > 31 indicate identity or extensive homology (p<0.05).
Protein scores are derived from ions scores as a non-probabilistic basis for ranking protein hits.

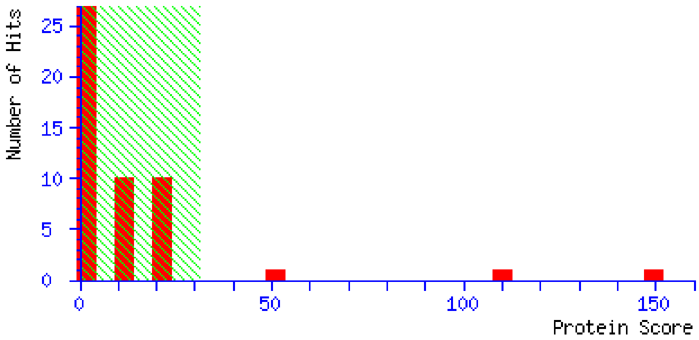

Matched peptide sequences: shown in **Bold Red**

```
  1 MGRVMNIFIV VLLCLTGVAI AEQCGRQAGG KLCPNNLCCS QYGWCGSSDD
 51 YCSPSKNCQS NCKGGGGGGG GGGGSASNVL ATYHLYNPQQ HGWDLNAVSA
101 YCSTWDANKP YSWRSKYGWT AFCGPVGAHG QPSCGKCLSV TNTGTGAKTT
151 VRIVDQCSNG GLDLDVNVFR QLDTDGKGYE RGHLTVNYQF VNCGDSFNPL
201 FSIMKSSVIN
```

Bold red (matched) residues: 117–136 (YGWT AFCGPVGAHG QPSCGK) and 153–170 (IVDQCSNG GLDLDVNVFR).

Matched peptide information:

Start - End	Observed	Mr(expt)	Mr(calc)	ppm	Miss	Sequence	
117 - 136	2136.9202	2135.9129	2135.9306	-8	0	K.YGWTAFCGPVGAHGQPSCGK.C	(Ions score 61)
153 - 170	2020.9714	2019.9641	2019.9684	-2	0	R.IVDQCSNGGLDLDVNVFR.Q	(Ions score 88)

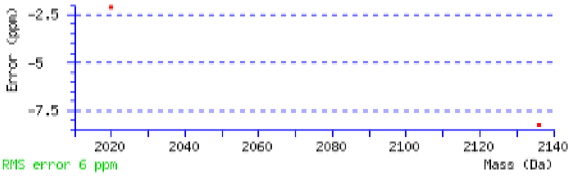

Spot No.: 77

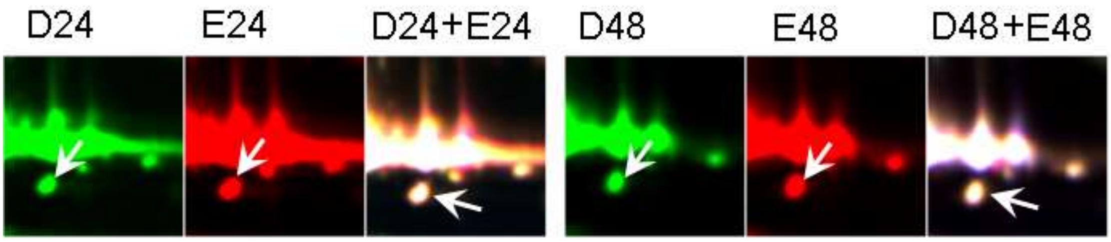

Accession No.: **scaffold0014_190374.mRNA1**

Plant species: ***Hevea brasiliensis***

Protein name：**Cysteine proteinase inhibitor**

Peptide sequences: **K.EVEGSANSVEINSLAR.Y; R.YAVDDYNQK.Q; K.QNALLEFK.K; K.QQVVAGTIYYITLEVTDGGQK.K; K.QQVVAGTIYYITLEVTDGGQKK.V; K.VWEKPWLNFK.E**

PFF Mascot score: **[667]** Sequence coverage %: **[64]**

Matched peptides No.: **[6]**

Calculated Mr: **11236** Calculated *p*I: **5.45**

Annotated PFF **spectra:**

Ions score is -10*Log(P), where P is the probability that the observed match is a random event.
Individual ions scores > 31 indicate identity or extensive homology (p<0.05).
Protein scores are derived from ions scores as a non-probabilistic basis for ranking protein hits.

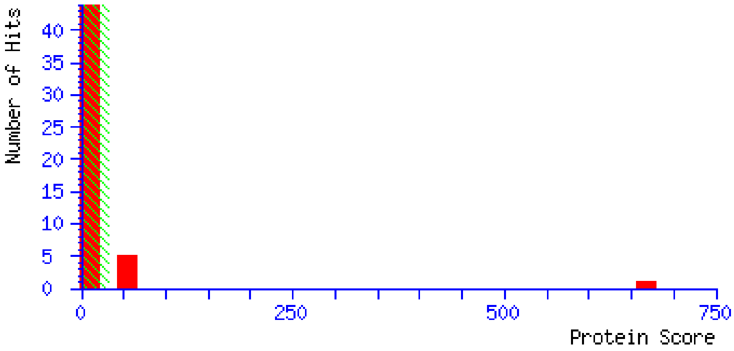

Matched peptide sequences: shown in Bold Red

1 MAKLGGVK**EV EGSANSVEIN SLARYAVDDY NQKQNALLEF K**KVVNAK**QQV**
51 **VAGTIYYITL EVTDGGQKK**V YEAK**VWEKPW LNFK**EVQEFK LIGDAPSDST
101 A

Matched peptide information:

Start - End	Observed	Mr(expt)	Mr(calc)	ppm	Miss	Sequence
9 - 24	1674.8320	1673.8247	1673.8220	2	0	K.EVEGSANSVEINSLAR.Y (Ions score 166)
25 - 33	1115.4976	1114.4903	1114.4931	-2	0	R.YAVDDYNQK.Q (Ions score 63)
34 - 41	962.5294	961.5221	961.5233	-1	0	K.QNALLEFK.K (Ions score 76)
48 - 68	2283.1851	2282.1778	2282.1794	-1	0	K.QQVVAGTIYYITLEVTDGGQK.K (Ions score 160)
48 - 69	2411.2842	2410.2769	2410.2744	1	1	K.QQVVAGTIYYITLEVTDGGQKK.V (Ions score 140)
75 - 84	1346.7258	1345.7185	1345.7183	0	1	K.VWEKPWLNFK.E (Ions score 62)

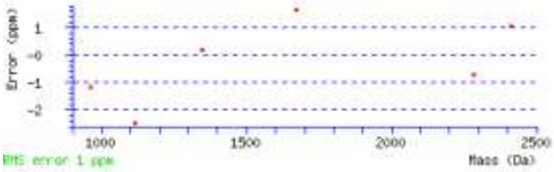

Spot No.: **78**

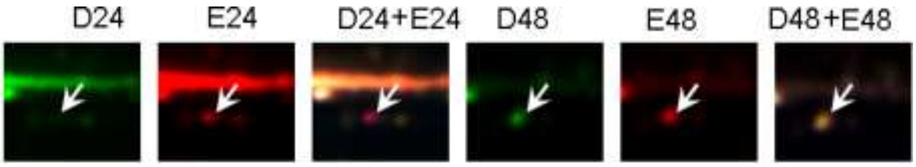

Accession No.: **scaffold0824_253044.mRNA1**

Plant species: ***Hevea brasiliensis***

Protein name： **Nuclear transport factor 2**

Peptide sequences: **K.AFVEHYYTTFDANR.A; R.AGLANLYQEGSMLTFEGQK.I; K.FSQMFHLIPTPQGSFYVLNDIFR.L**

PFF Mascot score: **[252]** Sequence coverage %: **[45]**

Matched peptides No.: **[3]**

Calculated Mr: **13696** Calculated *p*I: **5.68**

Annotated PFF **spectra:**

Ions score is -10*Log(P), where P is the probability that the observed match is a random event.
Individual ions scores > 30 indicate identity or extensive homology (p<0.05).
Protein scores are derived from ions scores as a non-probabilistic basis for ranking protein hits.

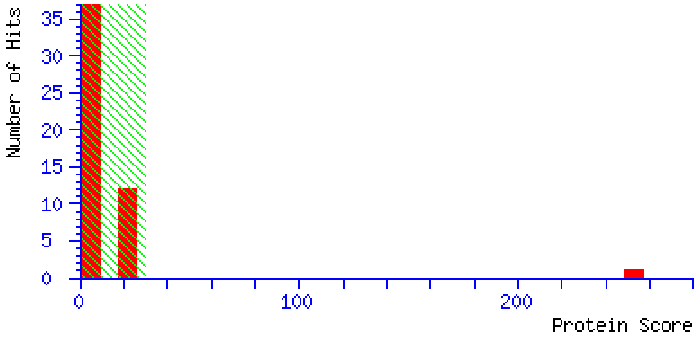

Matched peptide sequences: shown in **Bold Red**

```
  1 MEPDSVAKAF VEHYYTTFDA NRAGLANLYQ EGSMLTFEGQ KIQGAQNIVA
 51 KLISLPFQQC QHSITTVDCQ PSGPAGGMLV FVSGNLQLTG EQHALKFSQM
101 FHLIPTPQGS FYVLNDIFRL NYA
```

Matched peptide information:

Start - End	Observed	Mr(expt)	Mr(calc)	ppm	Miss	Sequence
9 - 22	1733.7362	1732.7289	1732.7845	-32	0	K.AFVEHYYTTFDANR.A (Ions score 99)
23 - 41	2056.9285	2055.9212	2055.9935	-35	0	R.AGLANLYQEGSMLTFEGQK.I (Ions score 90)
97 - 119	2757.2979	2756.2906	2756.3785	-32	0	K.FSQMFHLIPTPQGSFYVLNDIFR.L (Ions score 63)

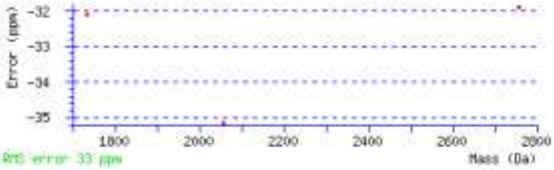

Spot No.: **79**

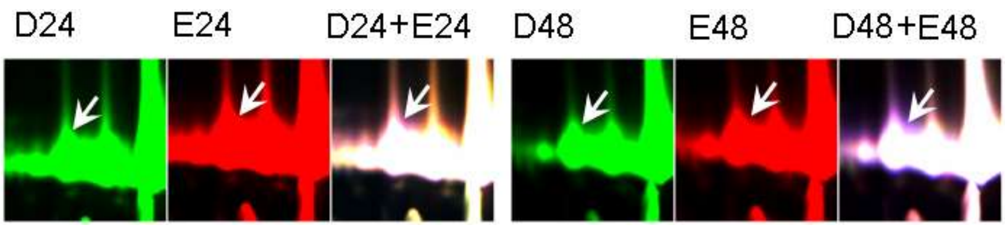

Accession No.: **scaffold1222_136753.mRNA1**

Plant species: ***Hevea brasiliensis***

Protein name: **Rubber elongation factor protein**

Peptide sequences: **K.DKSGPLQPGVDIIEGPVK.N; K.SGPLQPGVDIIEGPVKNVAVPLYNR.F; K.NVAVPLYNR.F; K.FVDSTVVASVTIIDR.S; K.DASIQVVSAIR.A**

PFF Mascot score: **[372]** Sequence coverage %: **[38]**

Matched peptides No.: **[5]**

Calculated Mr: **14713** Calculated p*I*: **5.04**

Annotated PFF **spectra:**

Ions score is -10*Log(P), where P is the probability that the observed match is a random event.
Individual ions scores > 31 indicate identity or extensive homology (p<0.05).
Protein scores are derived from ions scores as a non-probabilistic basis for ranking protein hits.

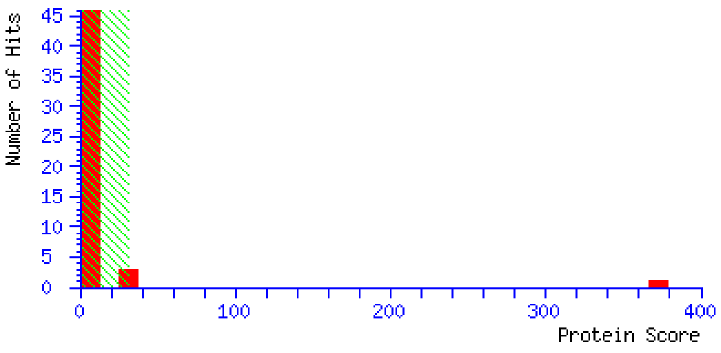

Matched peptide sequences: shown in Bold Red

```
  1 MAEDEDNQQG QGEGLKYLGF VQDAATYAVT TFSNVYLFAK DKSGPLQPGV
 51 DIIEGPVKNV AVPLYNRFSY IPNGALKFVD STVVASVTII DRSLPPIVKD
101 ASIQVVSAIR AAPEAARSLA SSLPGQTKIL AKVFYGEN
```

Matched peptide information:

Start - End	Observed	Mr(expt)	Mr(calc)	ppm	Miss	Sequence
41 - 58	1849.0927	1848.0854	1847.9993	47	1	K.DKSGPLQPGVDIIEGPVK.N (Ions score 70)
43 - 67	2632.5703	2631.5630	2631.4384	47	1	K.SGPLQPGVDIIEGPVKNVAVPLYNR.F (Ions score 35)
59 - 67	1045.6227	1044.6154	1044.5716	42	0	K.NVAVPLYNR.F (Ions score 60)
78 - 92	1621.9495	1620.9422	1620.8723	43	0	K.FVDSTVVASVTIIDR.S (Ions score 132)
100 - 110	1158.6958	1157.6885	1157.6404	42	0	K.DASIQVVSAIR.A (Ions score 75)

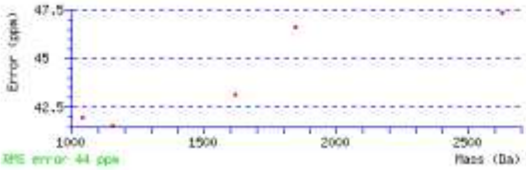

Supplement: Supplementary file 1 [file ijms-20-05082-s001.zip › ijms-597831-SI/ijms-597831-supplementary new 20191010/Figure S2 OK MS identification of DEPs on 2-D DIGE gels of small rubber particles.pdf]
