# Supplementary material for: Proteomic Landscape Has Revealed Small Rubber Particles Are Crucial Rubber Biosynthetic Machines for Ethylene-Stimulation in Natural Rubber Production
Source: Int J Mol Sci. 2019 Oct 14;20(20):5082. doi: 10.3390/ijms20205082 (PMC6829444; doi:10.3390/ijms20205082)
Supplement: Supplementary file 1 [file ijms-20-05082-s001.zip › ijms-597831-SI/ijms-597831-supplementary new 20191010/Figure S3 OK-1009 Phosphopeptides of phosphorylation amino acid sites in SRPP and REF.pdf]

Figure S3: Phosphopeptides and phosphorylation amino acid sites in different REF and SRPP isoforms

Protein name: REF138

Location of the six spots identified as REF138 by MS from 2-D DIGE gels:

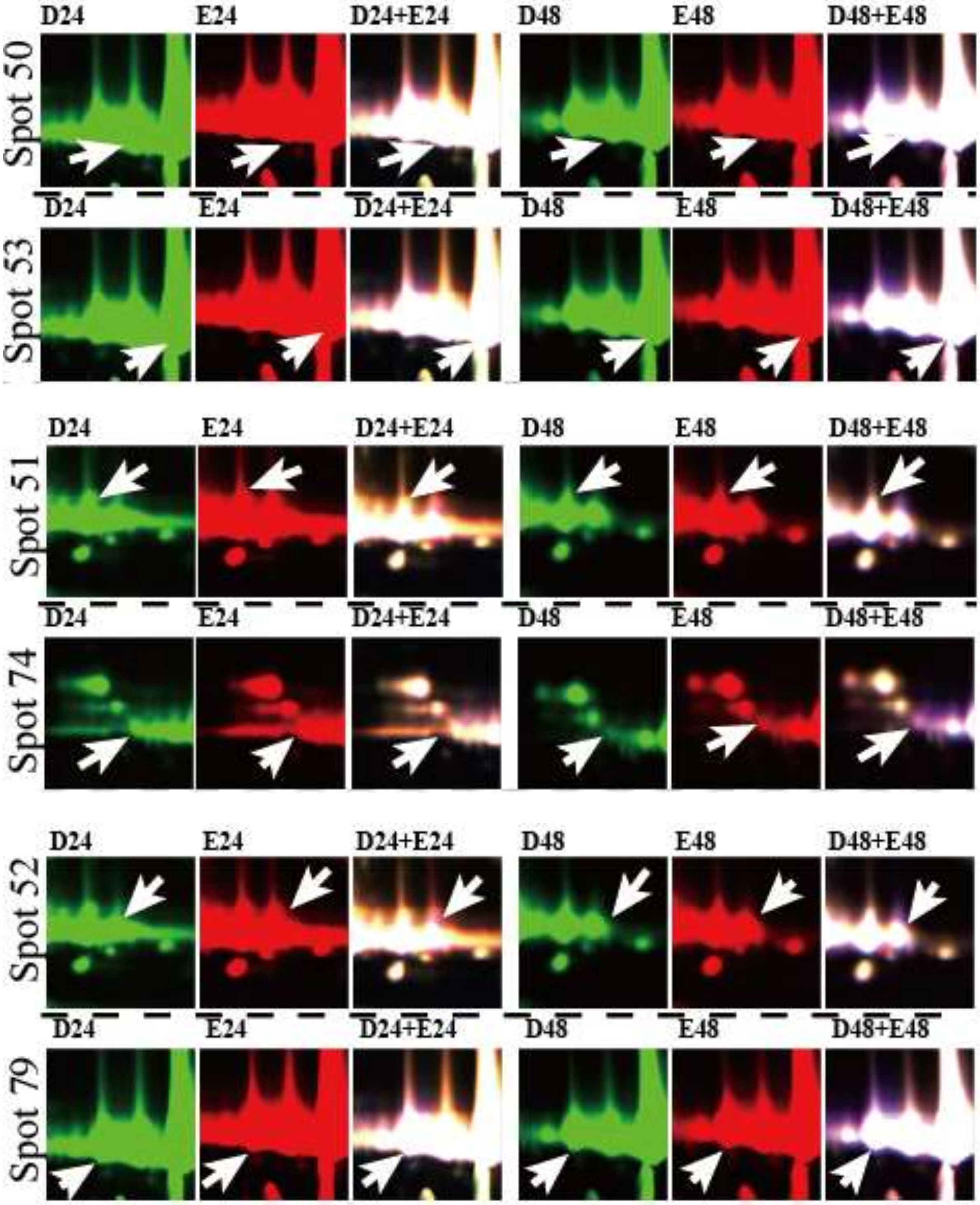

Detail information for phosphorylation of amino acid sites in REF138:

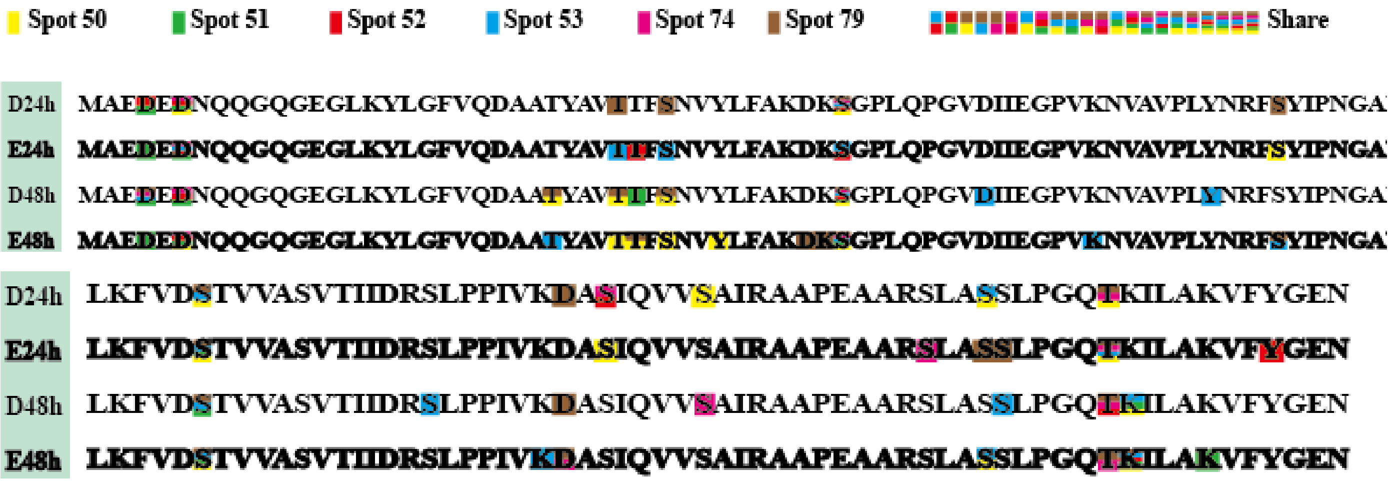

Predicted 3-D structure and phosphorylated amino acids in different REF138 isoforms:

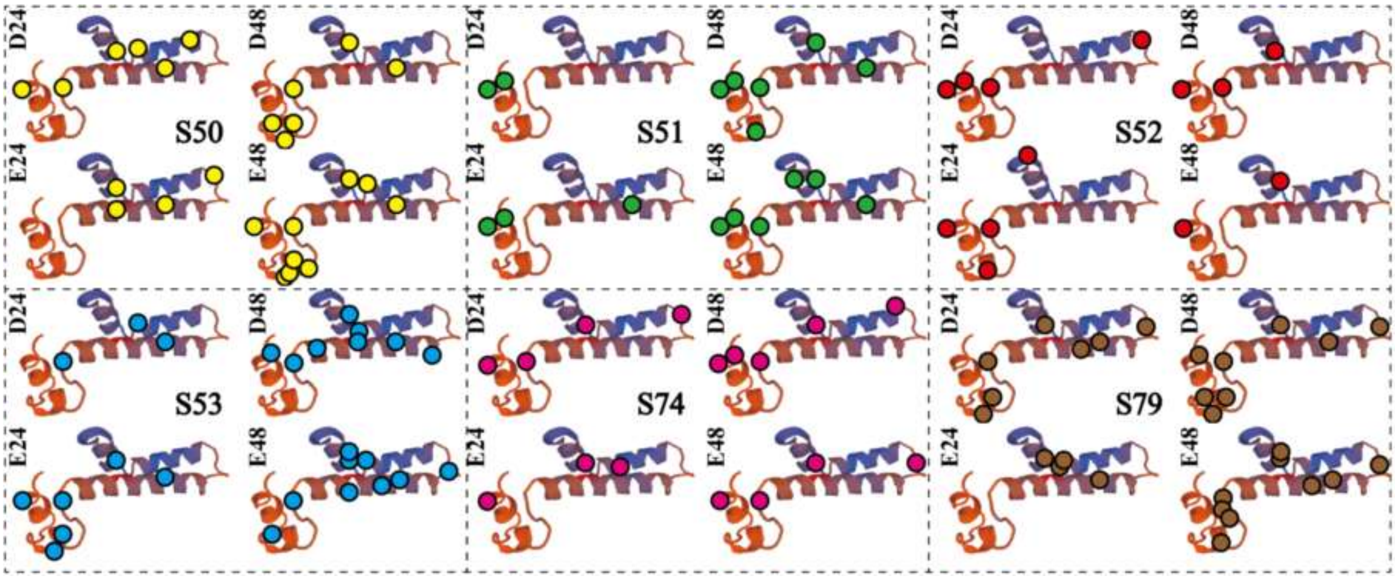

Spot No. 50

Changed pattern on DIGE gel:

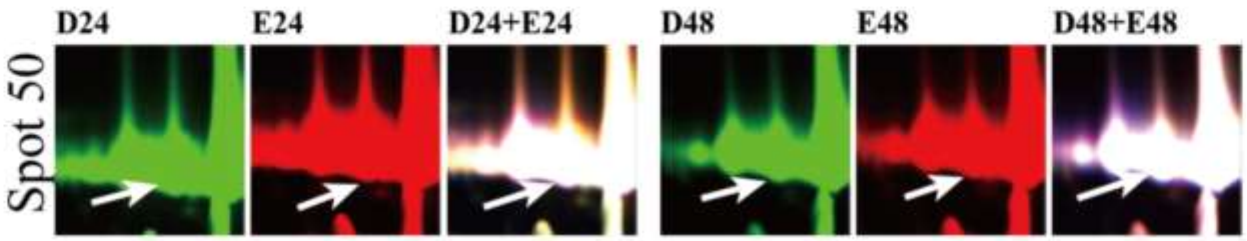

Location in rubber genome: scaffold1222_136753.mRNA1

Protein name: Rubber elongation factor protein/REF138

Predicted 3-D structure and phosphorylated amino acids:

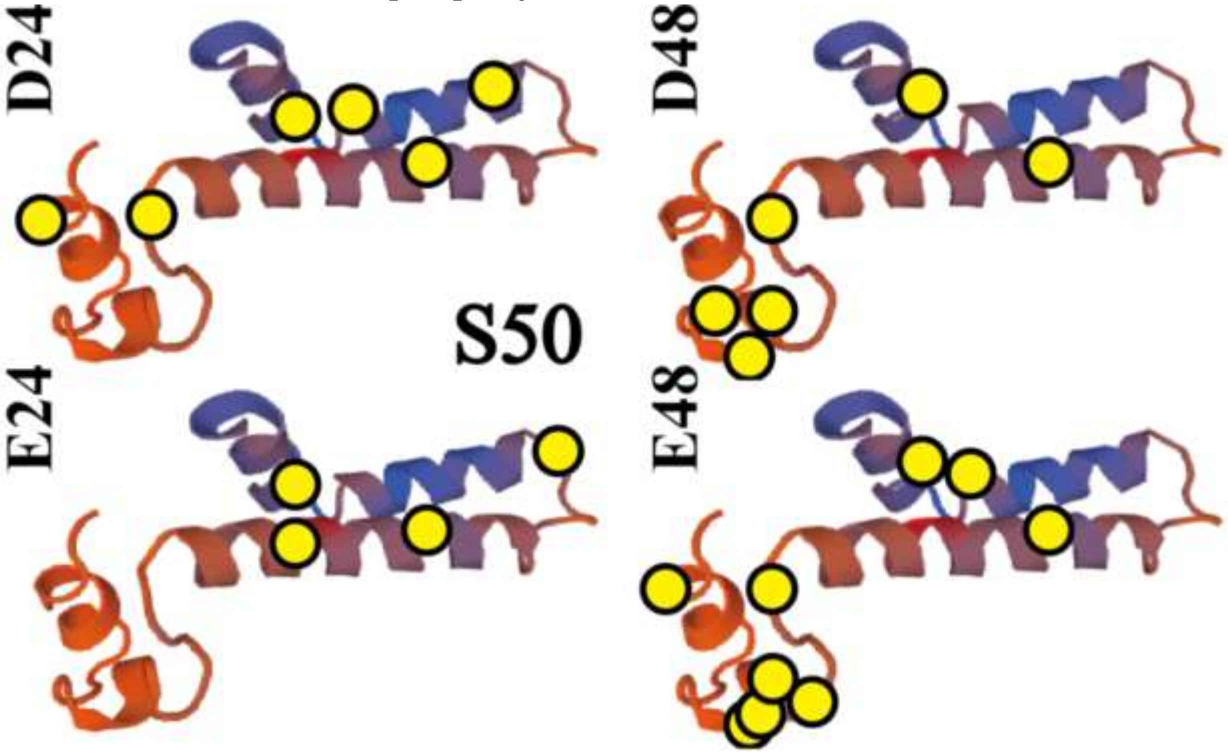

Detail information for phosphorylation of amino acid sites:

D24: MAEDEDNQQGQGEGLKYLGFVQDAATYAVTTFSNVYLFAKDKSGPLQPGVDIIEGPVKNVAVPLYNRFSYIPNGA
E24: MAEDEDNQQGQGEGLKYLGFVQDAATYAVTTFSNVYLFAKDKSGPLQPGVDIIEGPVKNVAVPLYNRFSYIPNGA
D48: MAEDEDNQQGQGEGLKYLGFVQDAATYAVTTFSNVYLFAKDKSGPLQPGVDIIEGPVKNVAVPLYNRFSYIPNGA
E48: MAEDEDNQQGQGEGLKYLGFVQDAATYAVTTFSNVYLFAKDKSGPLQPGVDIIEGPVKNVAVPLYNRFSYIPNGA

D24: LKFVDSTVVASVTIIDRSLPPIVKDASIQVVSAIRAAPEAARSLASSLPGQTKILAKVFYGEN
E24: LKFVDSTVVASVTIIDRSLPPIVKDASIQVVSAIRAAPEAARSLASSLPGQTKILAKVFYGEN
D48: LKFVDSTVVASVTIIDRSLPPIVKDASIQVVSAIRAAPEAARSLASSLPGQTKILAKVFYGEN
E48: LKFVDSTVVASVTIIDRSLPPIVKDASIQVVSAIRAAPEAARSLASSLPGQTKILAKVFYGEN

Spot No. 51

Changed pattern on DIGE gel:

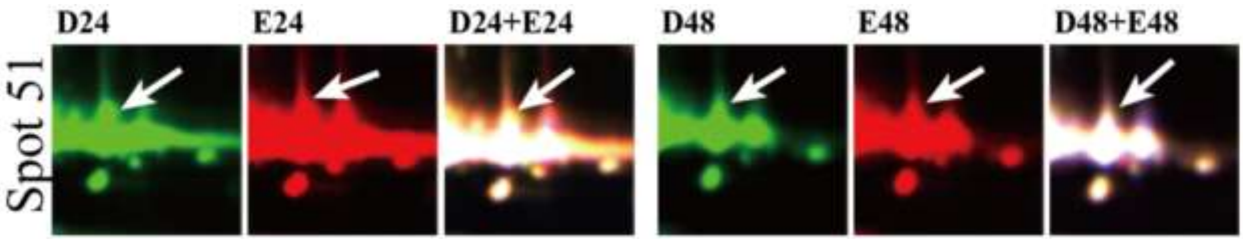

Location in rubber genome: scaffold1222_136753.mRNA1

Protein name: Rubber elongation factor protein/REF138

Predicted 3-D structure and phosphorylated amino acids:

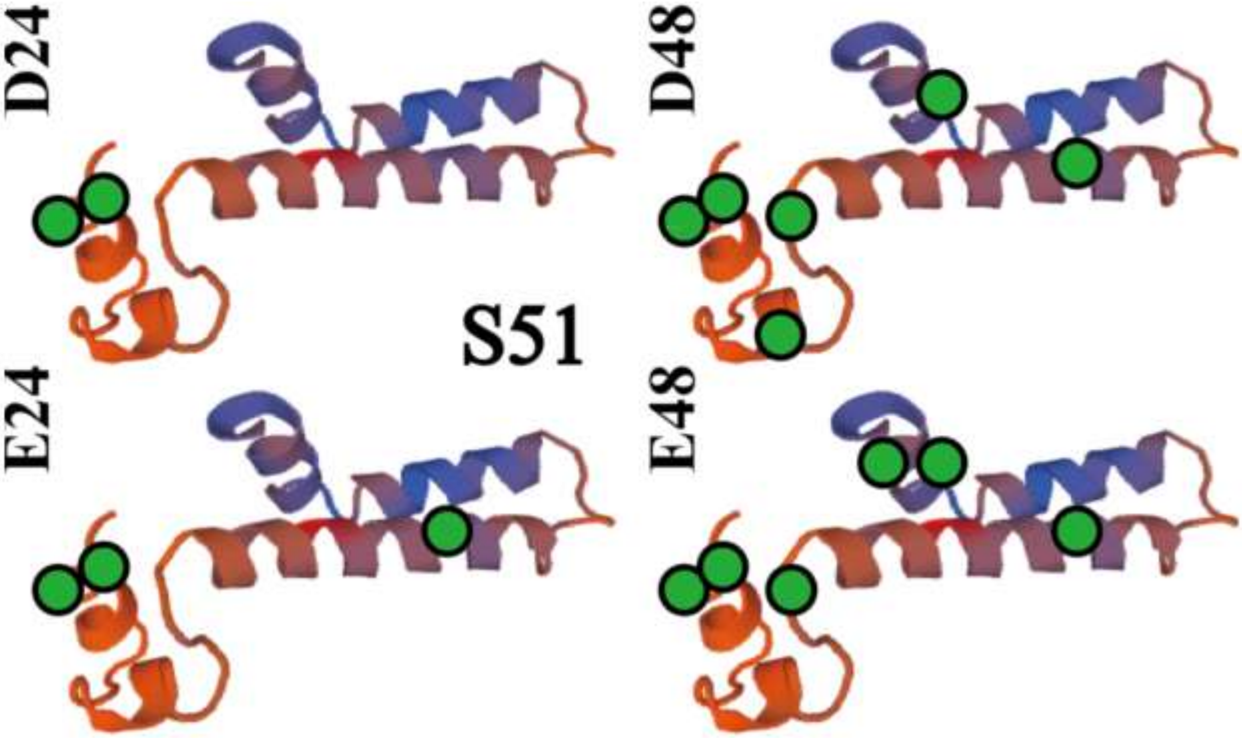

Detail information for phosphorylation of amino acid sites:

D24: MAEDEDNQQGQGEGLKYLGFVQDAATYAVTTFSNVYLFAKDKSGPLQPGVDIIEGPVKNVAVPLYNRFSYIPNGA
E24: MAEDEDNQQGQGEGLKYLGFVQDAATYAVTTFSNVYLFAKDKSGPLQPGVDIIEGPVKNVAVPLYNRFSYIPNGA
D48: MAEDEDNQQGQGEGLKYLGFVQDAATYAVTTFSNVYLFAKDKSGPLQPGVDIIEGPVKNVAVPLYNRFSYIPNGA
E48: MAEDEDNQQGQGEGLKYLGFVQDAATYAVTTFSNVYLFAKDKSGPLQPGVDIIEGPVKNVAVPLYNRFSYIPNGA

D24: LKFVDSTVVASVTIIDRSLPPIVKDASIQVVSAIRAAPEAARSLASSLPGQTKILAKVFYGEN
E24: LKFVDSTVVASVTIIDRSLPPIVKDASIQVVSAIRAAPEAARSLASSLPGQTKILAKVFYGEN
D48: LKFVDSTVVASVTIIDRSLPPIVKDASIQVVSAIRAAPEAARSLASSLPGQTKILAKVFYGEN
E48: LKFVDSTVVASVTIIDRSLPPIVKDASIQVVSAIRAAPEAARSLASSLPGQTKILAKVFYGEN

Spot No. 52

Changed pattern on DIGE gel:

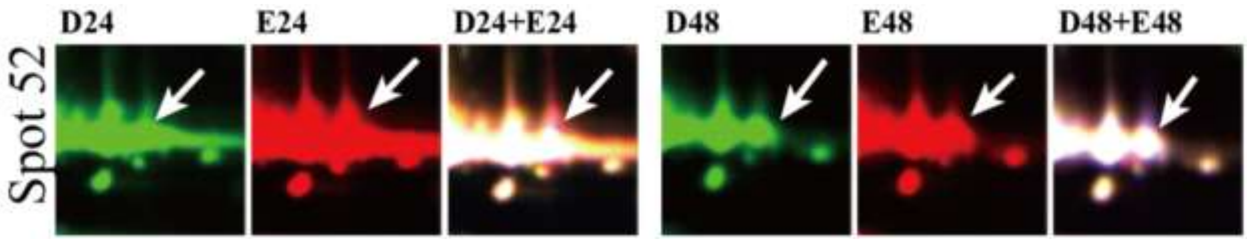

Location in rubber genome: scaffold1222_136753.mRNA1

Protein name: Rubber elongation factor protein/REF138

Predicted 3-D structure and phosphorylated amino acids:

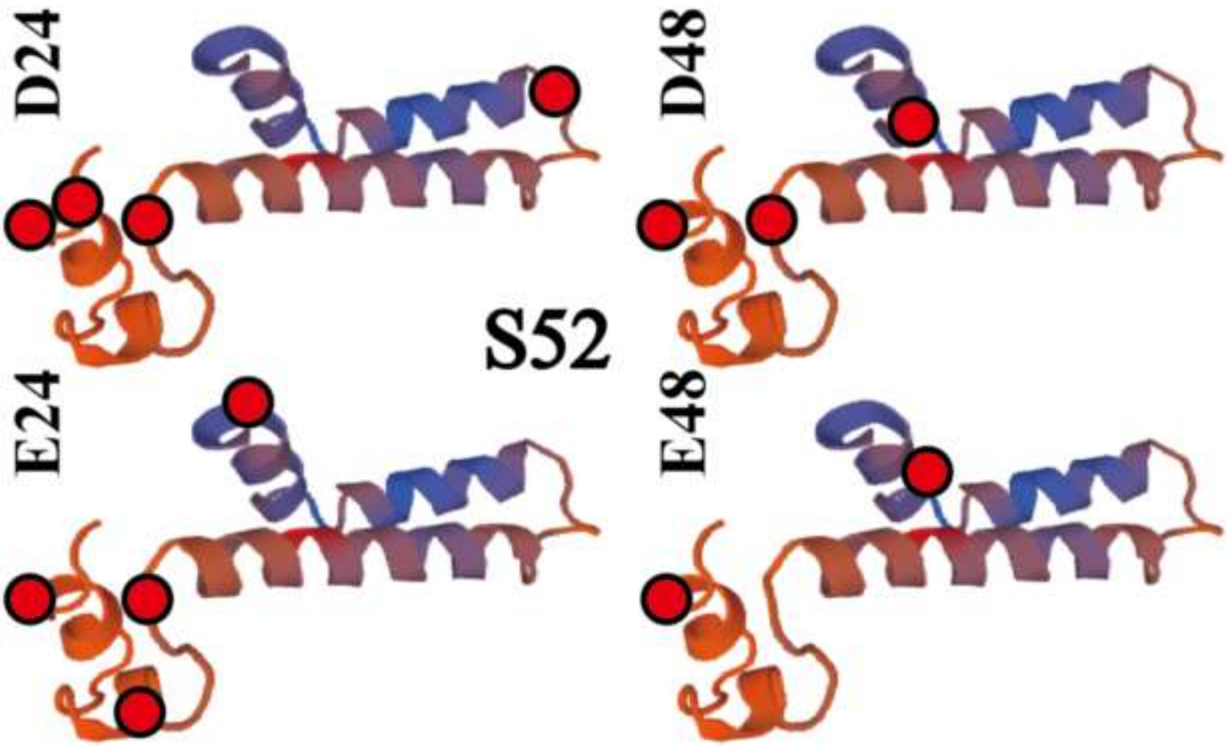

Detail information for phosphorylation of amino acid sites:

D24: MAEDEDNQQGQGEGLKYLGFVQDAATYAVTTFSNVYLFAKDKSGPLQPGVDIIEGPVKNVAVPLYNRFSYIPNGA
E24: MAEDEDNQQGQGEGLKYLGFVQDAATYAVTTFSNVYLFAKDKSGPLQPGVDIIEGPVKNVAVPLYNRFSYIPNGA
D48: MAEDEDNQQGQGEGLKYLGFVQDAATYAVTTFSNVYLFAKDKSGPLQPGVDIIEGPVKNVAVPLYNRFSYIPNGA
E48: MAEDEDNQQGQGEGLKYLGFVQDAATYAVTTFSNVYLFAKDKSGPLQPGVDIIEGPVKNVAVPLYNRFSYIPNGA

D24: LKFVDSTVVASVTIIDRSLPPIVKDASIQVVSAIRAAPEAARSLASSLPGQTKILAKVFYGEN
E24: LKFVDSTVVASVTIIDRSLPPIVKDASIQVVSAIRAAPEAARSLASSLPGQTKILAKVFYGEN
D48: LKFVDSTVVASVTIIDRSLPPIVKDASIQVVSAIRAAPEAARSLASSLPGQTKILAKVFYGEN
E48: LKFVDSTVVASVTIIDRSLPPIVKDASIQVVSAIRAAPEAARSLASSLPGQTKILAKVFYGEN

Spot No. 53

Changed pattern on DIGE gel:

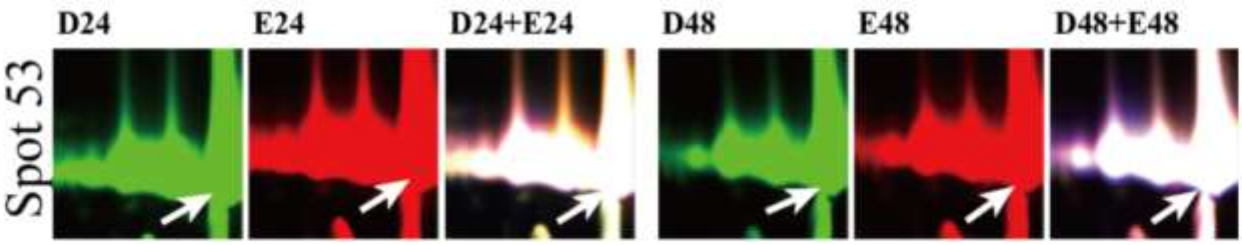

Location in rubber genome: scaffold1222_136753.mRNA1

Protein name: Rubber elongation factor protein/REF138

Predicted 3-D structure and phosphorylated amino acids:

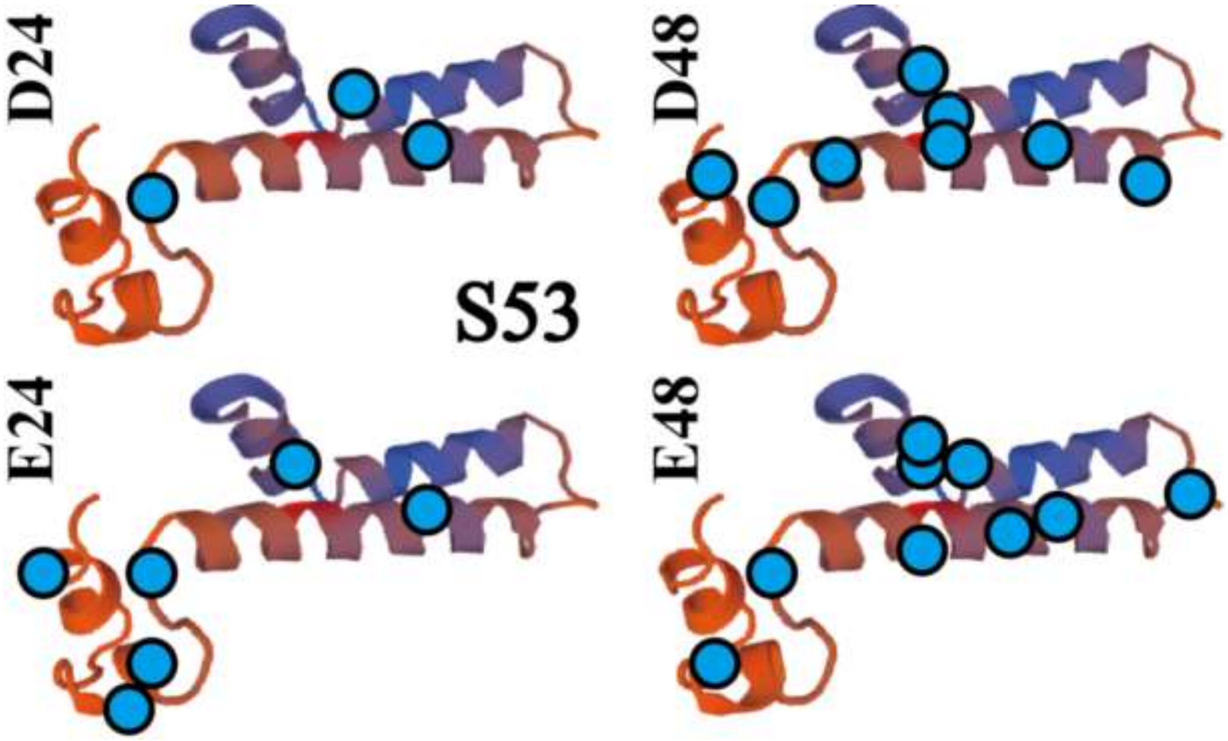

Detail information for phosphorylation of amino acid sites:

D24: MAEDEDNQQGQGEGLKYLGFVQDAATYAVTTFSNVYLFAKDKSGPLQPGVDIIEGPVKNVAVPLYNRFSYIPNGA
E24: MAEDEDNQQGQGEGLKYLGFVQDAATYAVTTFSNVYLFAKDKSGPLQPGVDIIEGPVKNVAVPLYNRFSYIPNGA
D48: MAEDEDNQQGQGEGLKYLGFVQDAATYAVTTFSNVYLFAKDKSGPLQPGVDIIEGPVKNVAVPLYNRFSYIPNGA
E48: MAEDEDNQQGQGEGLKYLGFVQDAATYAVTTFSNVYLFAKDKSGPLQPGVDIIEGPVKNVAVPLYNRFSYIPNGA

D24: LKFVDSTVVASVTIIDRSLPPIVKDASIQVVSAIRAAPEAARSLASSLPGQTKILAKVFYGEN
E24: LKFVDSTVVASVTIIDRSLPPIVKDASIQVVSAIRAAPEAARSLASSLPGQTKILAKVFYGEN
D48: LKFVDSTVVASVTIIDRSLPPIVKDASIQVVSAIRAAPEAARSLASSLPGQTKILAKVFYGEN
E48: LKFVDSTVVASVTIIDRSLPPIVKDASIQVVSAIRAAPEAARSLASSLPGQTKILAKVFYGEN

Spot No. 74
Changed pattern on DIGE gel:

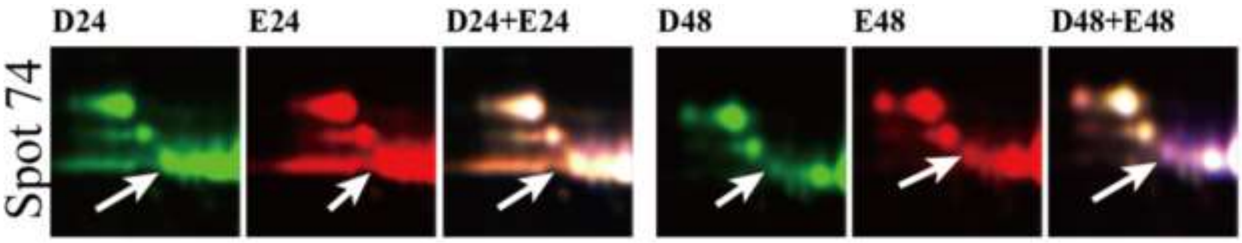

Location in rubber genome: scaffold1222_136753.mRNA1
Protein name: Rubber elongation factor protein/REF138
Predicted 3-D structure and phosphorylated amino acids:

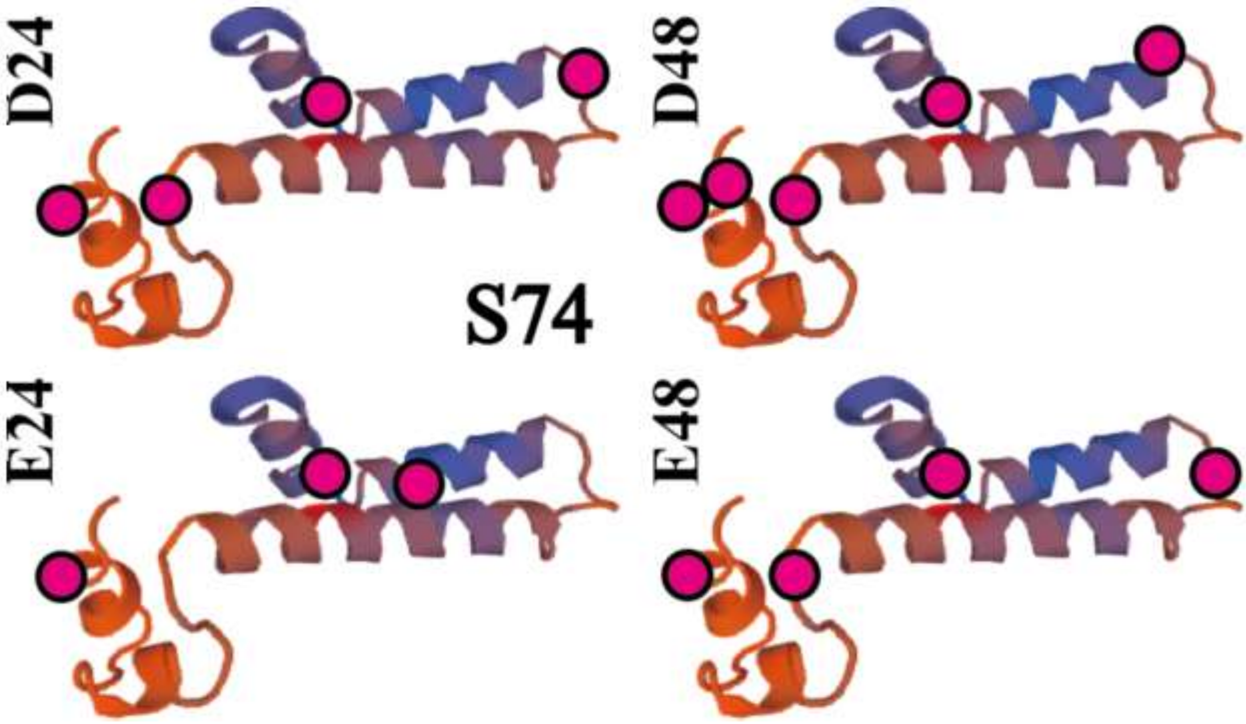

Detail information for phosphorylation of amino acid sites:

D24: MAEDEDNQQGQGEGLKYLGFVQDAATYAVTTFSNVYLFAKDKSGPLQPGVDIIEGPVKNVAVPLYNRFSYIPNGA
E24: MAEDEDNQQGQGEGLKYLGFVQDAATYAVTTFSNVYLFAKDKSGPLQPGVDIIEGPVKNVAVPLYNRFSYIPNGA
D48: MAEDEDNQQGQGEGLKYLGFVQDAATYAVTTFSNVYLFAKDKSGPLQPGVDIIEGPVKNVAVPLYNRFSYIPNGA
E48: MAEDEDNQQGQGEGLKYLGFVQDAATYAVTTFSNVYLFAKDKSGPLQPGVDIIEGPVKNVAVPLYNRFSYIPNGA

D24: LKFVDSTVVASVTIIDRSLPPIVKDASIQVVSAIRAAPEAARSLASSLPGQTKILAKVFYGEN
E24: LKFVDSTVVASVTIIDRSLPPIVKDASIQVVSAIRAAPEAARSLASSLPGQTKILAKVFYGEN
D48: LKFVDSTVVASVTIIDRSLPPIVKDASIQVVSAIRAAPEAARSLASSLPGQTKILAKVFYGEN
E48: LKFVDSTVVASVTIIDRSLPPIVKDASIQVVSAIRAAPEAARSLASSLPGQTKILAKVFYGEN

Spot No. 79

Changed pattern on DIGE gel:

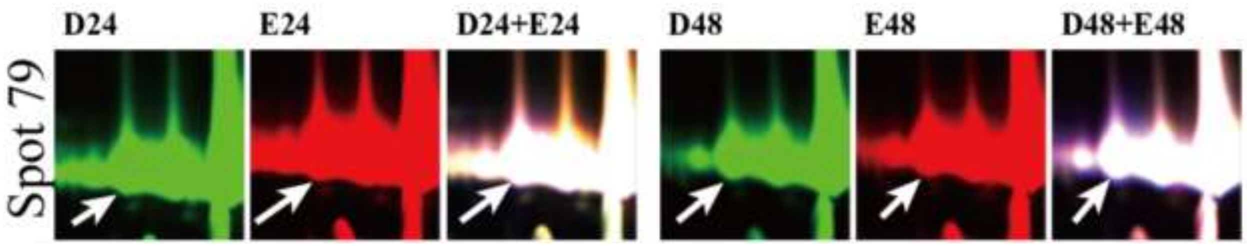

Location in rubber genome: scaffold1222_136753.mRNA1

Protein name: Rubber elongation factor protein/REF138

Predicted 3-D structure and phosphorylated amino acids:

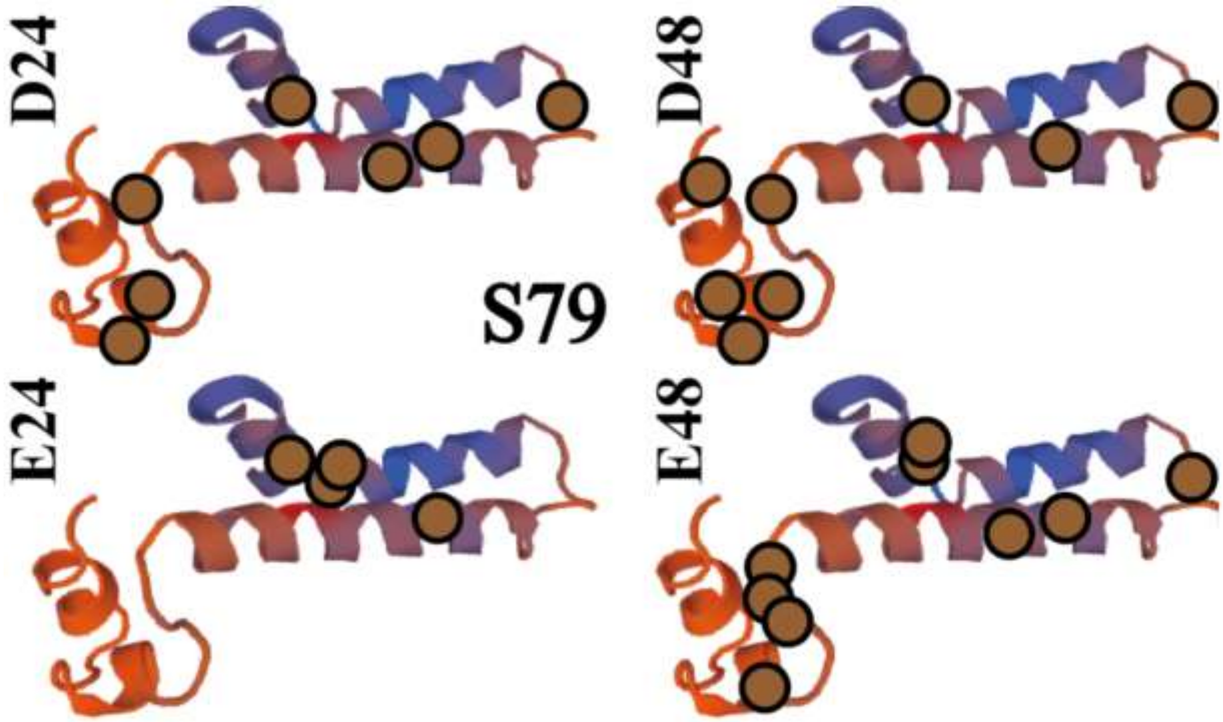

Detail information for phosphorylation of amino acid sites:

D24： MAEDEDNQQGQGEGLKYLGFVQDAATYAV**T**TF**S**NVYLFAKDK**S**GPLQPGVDIIEGPVKNVAVPLYNRF**S**YIPNGA
E24： MAEDEDNQQGQGEGLKYLGFVQDAATYAVTTFSNVYLFAKDKSGPLQPGVDIIEGPVKNVAVPLYNRFSYIPNGA
D48： MAE**D**EDNQQGQGEGLKYLGFVQDAA**T**YAV**T**TF**S**NVYLFAKDK**S**GPLQPGVDIIEGPVKNVAVPLYNRFSYIPNGA
E48： MAEDEDNQQGQGEGLKYLGFVQDAATYAVT**T**FSNVYLFAK**DKS**GPLQPGVDIIEGPVKNVAVPLYNRF**S**YIPNGA

D24： LKFVD**S**TVVASVTIIDRSLPPIVK**D**ASIQVVSAIRAAPEAARSLASSLPGQ**T**KILAKVFYGEN
E24： LKFVD**S**TVVASVTIIDRSLPPIVKDASIQVVSAIRAAPEAARSLA**SS**LPGQ**T**KILAKVFYGEN
D48： LKFVD**S**TVVASVTIIDRSLPPIVK**D**ASIQVVSAIRAAPEAARSLASSLPGQ**T**KILAKVFYGEN
E48： LKFVD**S**TVVASVTIIDRSLPPIVK**D**ASIQVVSAIRAAPEAARSLASSLPGQ**TK**ILAKVFYGEN

Protein name: REF175

Location of the two spots identified as REF175 by MS from 2-D DIGE gels:

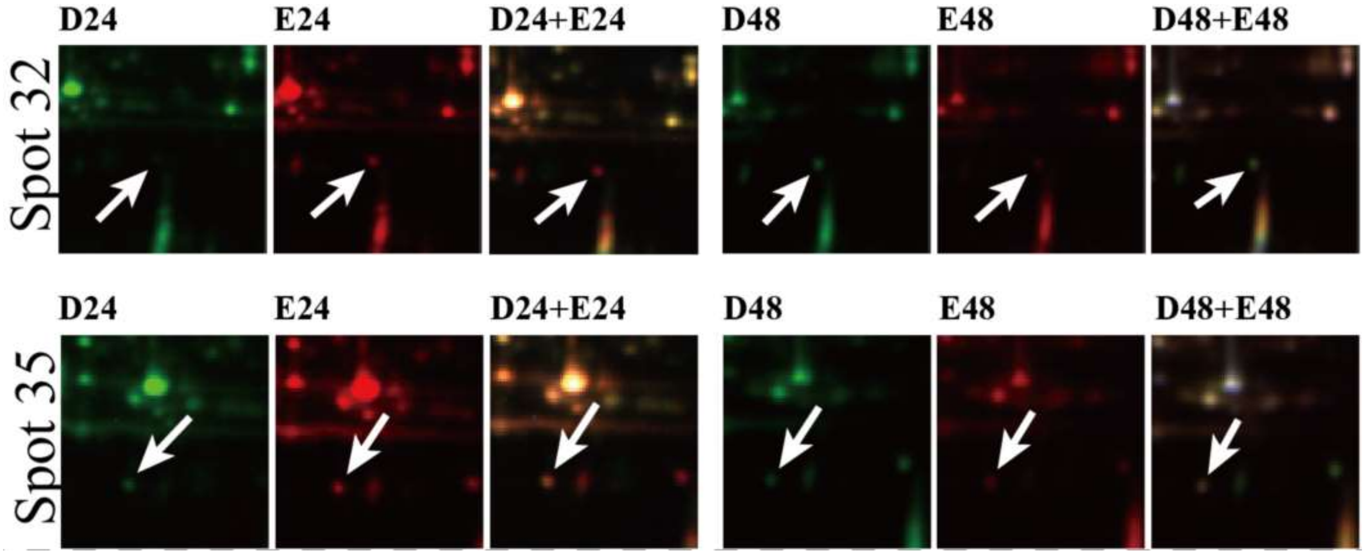

Detail information for phosphorylation of amino ac id sites in REF175:

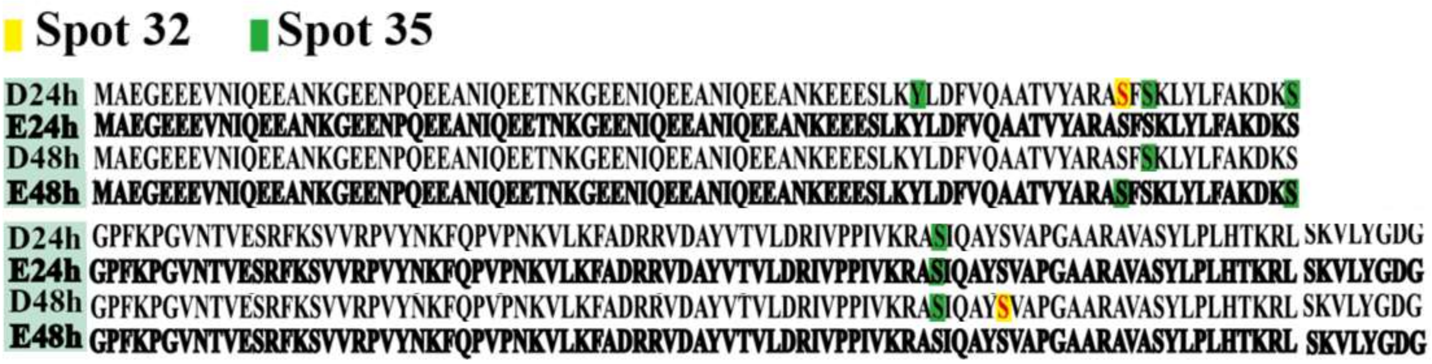

Predicted 3-D structure and phosphorylated amino acids in different REF175 isoforms:

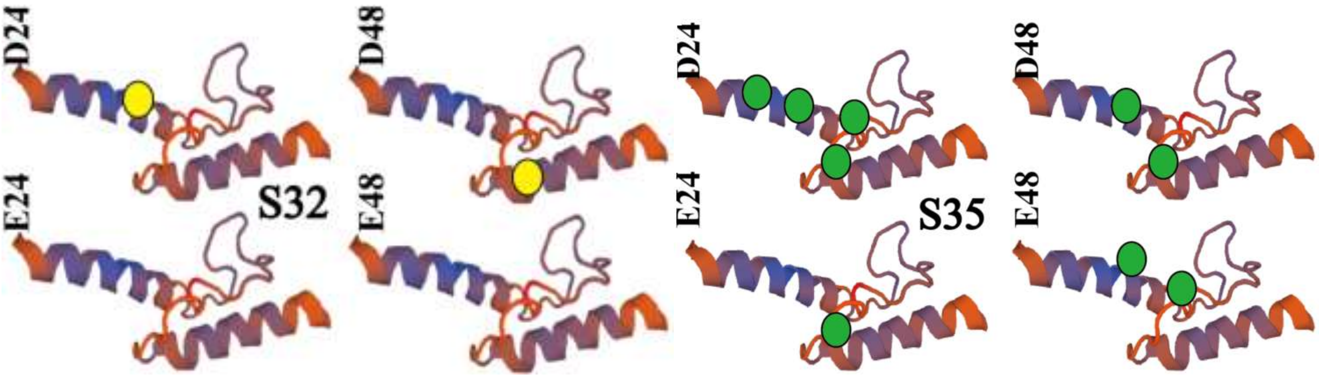

Spot No. 32

Changed pattern on DIGE gel:

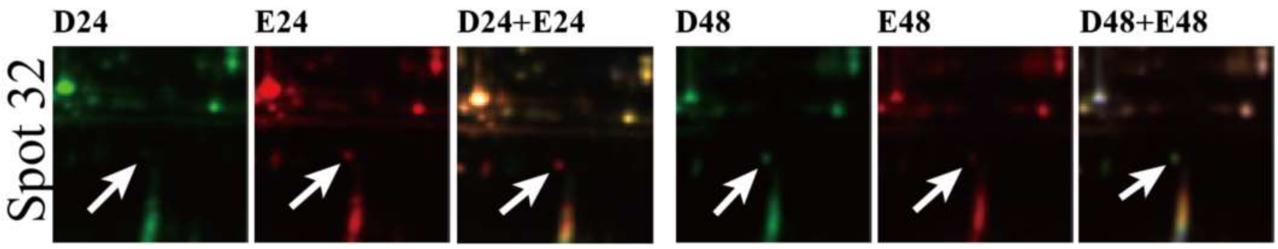

Location in rubber genome: scaffold1222_100110.mRNA1

Protein name: Rubber elongation factor protein/REF175

Predicted 3-D structure and phosphorylated amino acids:

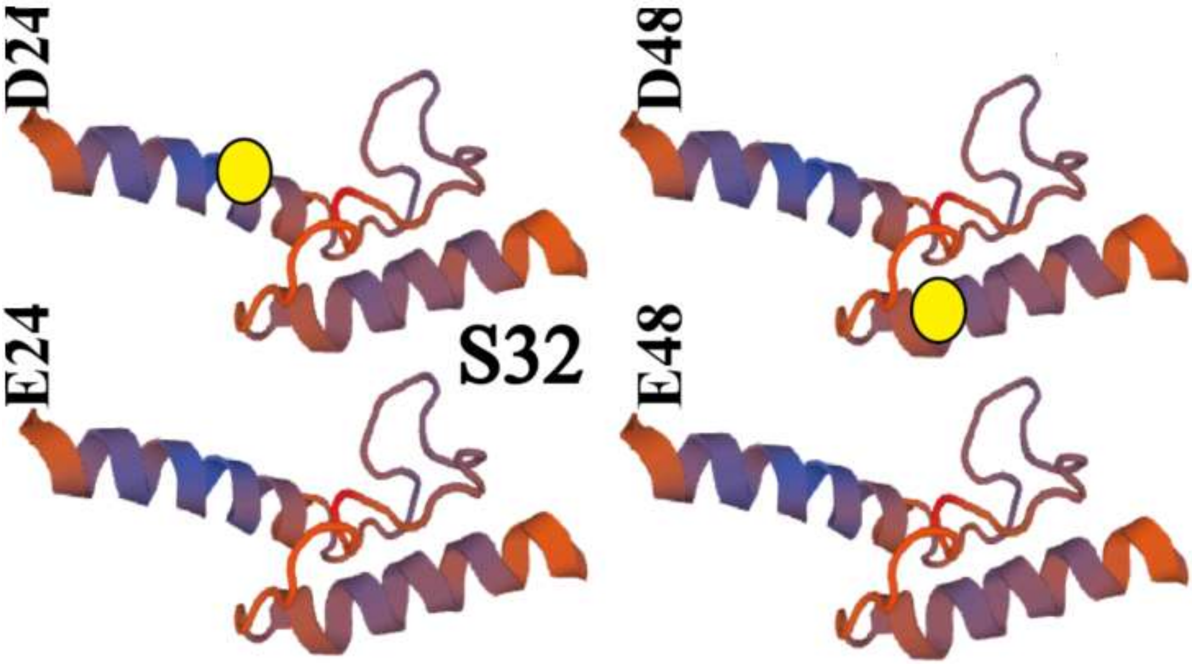

Detail information for phosphorylation of amino acid sites:

D24: MAEGEEEVNIQEEANKGEENPQEEANIQEETNKGEENIQEEANIQEEANKEEESLKYLDFVQAATVYARASFSKL
E24: MAEGEEEVNIQEEANKGEENPQEEANIQEETNKGEENIQEEANIQEEANKEEESLKYLDFVQAATVYARASFSKL
D48: MAEGEEEVNIQEEANKGEENPQEEANIQEETNKGEENIQEEANIQEEANKEEESLKYLDFVQAATVYARASFSKL
E48: MAEGEEEVNIQEEANKGEENPQEEANIQEETNKGEENIQEEANIQEEANKEEESLKYLDFVQAATVYARASFSKL

D24: YLFAKDKSGPFKPGVNTVESRFKSVVRPVYNKFQPVPNKVLKFADRRVDAYVTVLDRIVPPIVKRASIQAYSVAP
E24: YLFAKDKSGPFKPGVNTVESRFKSVVRPVYNKFQPVPNKVLKFADRRVDAYVTVLDRIVPPIVKRASIQAYSVAP
D48: YLFAKDKSGPFKPGVNTVESRFKSVVRPVYNKFQPVPNKVLKFADRRVDAYVTVLDRIVPPIVKRASIQAYSVAP
E48: YLFAKDKSGPFKPGVNTVESRFKSVVRPVYNKFQPVPNKVLKFADRRVDAYVTVLDRIVPPIVKRASIQAYSVAP

D24: GAARAVASYLPLHTKRLSKVLYGDG
E24: GAARAVASYLPLHTKRLSKVLYGDG
D48: GAARAVASYLPLHTKRLSKVLYGDG
E48: GAARAVASYLPLHTKRLSKVLYGDG

Spot No. 35

Changed pattern on DIGE gel:

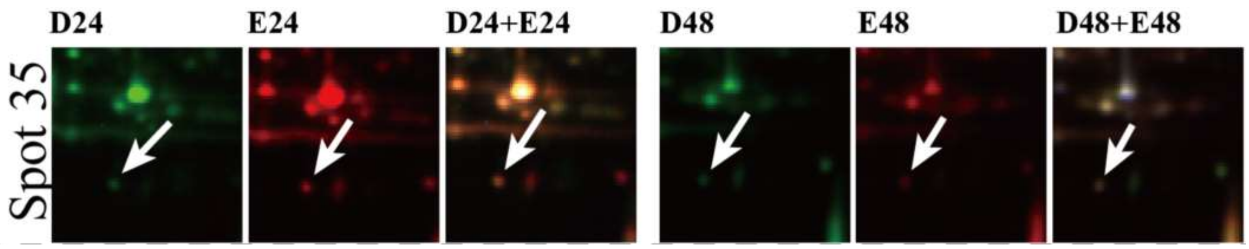

Location in rubber genome: scaffold1222_100110.mRNA1

Protein name: Rubber elongation factor protein/REF175

Predicted 3-D structure and phosphorylated amino acids:

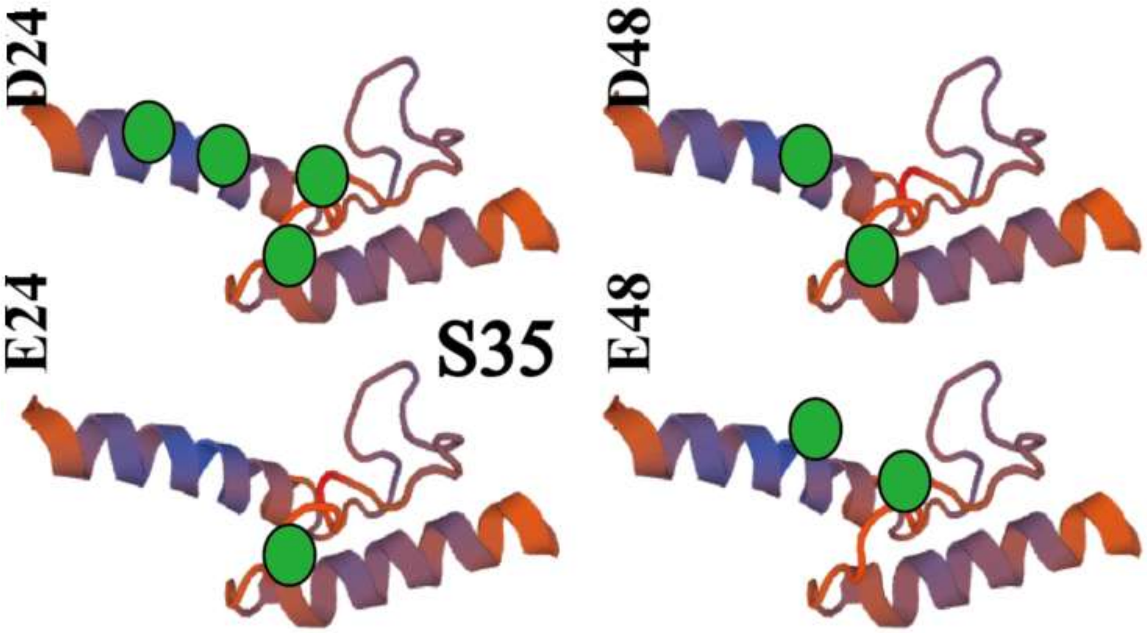

Detail information for phosphorylation of amino acid sites:

D24：MAEGEEEVNIQEEANKGEENPQEEANIQEETNKGEENIQEEANIQEEANKEEESLKYLDFVQAATVYARASFSKL
E24：MAEGEEEVNIQEEANKGEENPQEEANIQEETNKGEENIQEEANIQEEANKEEESLKYLDFVQAATVYARASFSKL
D48：MAEGEEEVNIQEEANKGEENPQEEANIQEETNKGEENIQEEANIQEEANKEEESLKYLDFVQAATVYARASFSKL
E48：MAEGEEEVNIQEEANKGEENPQEEANIQEETNKGEENIQEEANIQEEANKEEESLKYLDFVQAATVYARASFSKL

D24：YLFAKDKSGPFKPGVNTVESRFKSVVRPVYNKFQPVPNKVLKFADRRVDAYVTVLDRIVPPIVKRASIQAYSVAP
E24：YLFAKDKSGPFKPGVNTVESRFKSVVRPVYNKFQPVPNKVLKFADRRVDAYVTVLDRIVPPIVKRASIQAYSVAP
D48：YLFAKDKSGPFKPGVNTVESRFKSVVRPVYNKFQPVPNKVLKFADRRVDAYVTVLDRIVPPIVKRASIQAYSVAP
E48：YLFAKDKSGPFKPGVNTVESRFKSVVRPVYNKFQPVPNKVLKFADRRVDAYVTVLDRIVPPIVKRASIQAYSVAP

D24：GAARAVASYLPLHTKRLSKVLYGDG
E24：GAARAVASYLPLHTKRLSKVLYGDG
D48：GAARAVASYLPLHTKRLSKVLYGDG
E48：GAARAVASYLPLHTKRLSKVLYGDG

Protein name: REF258

Location of the five spots identified as REF258 by MS from 2-D DIGE gels:

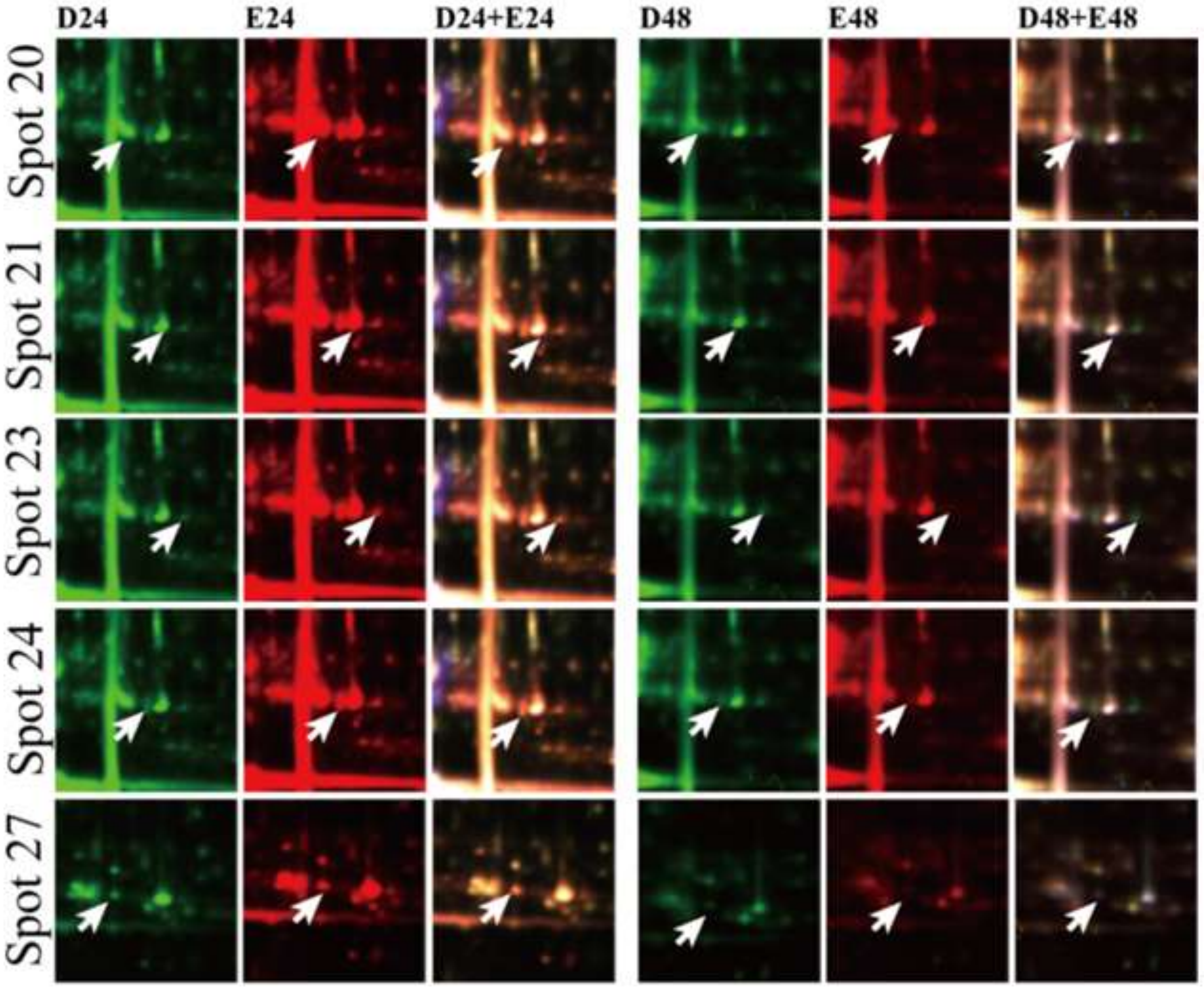

Detail information for phosphorylation of amino ac id sites in REF258:

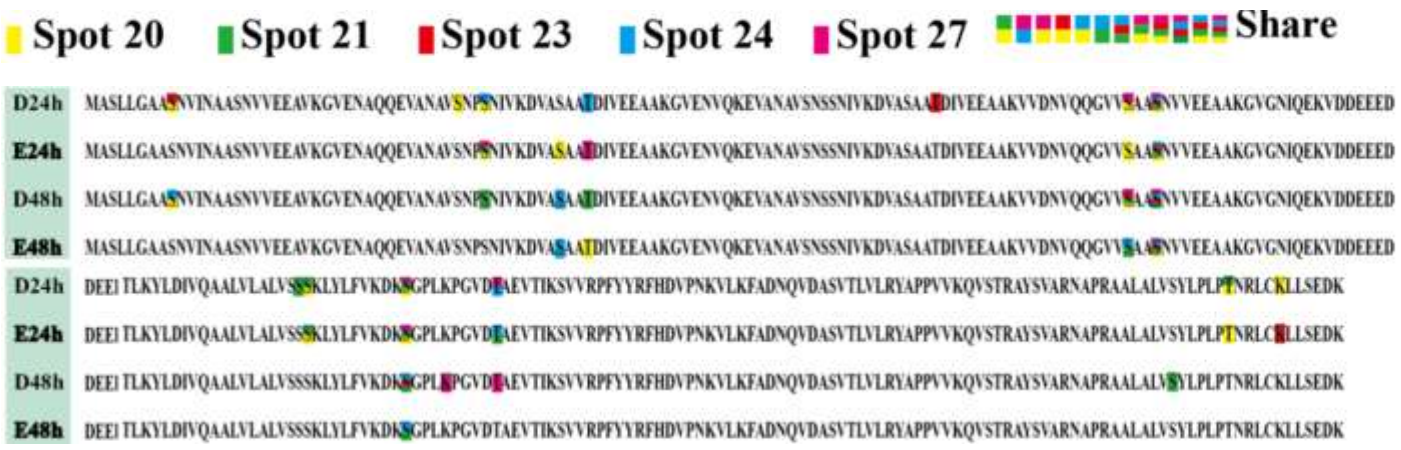

Predicted 3-D structure and phosphorylated amino acids in different REF258 isoforms:

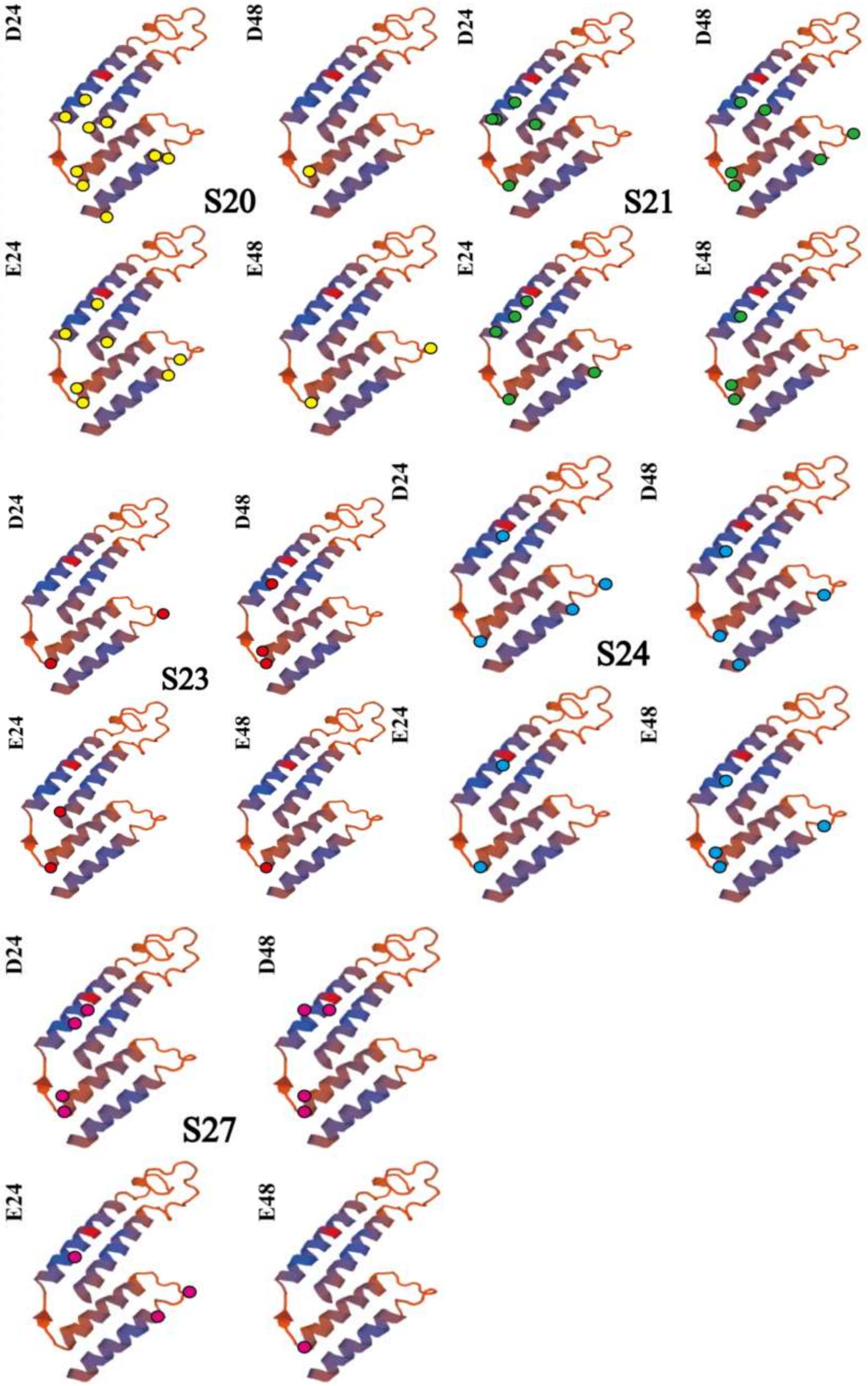

Spot No. 20

Changed pattern on DIGE gel:

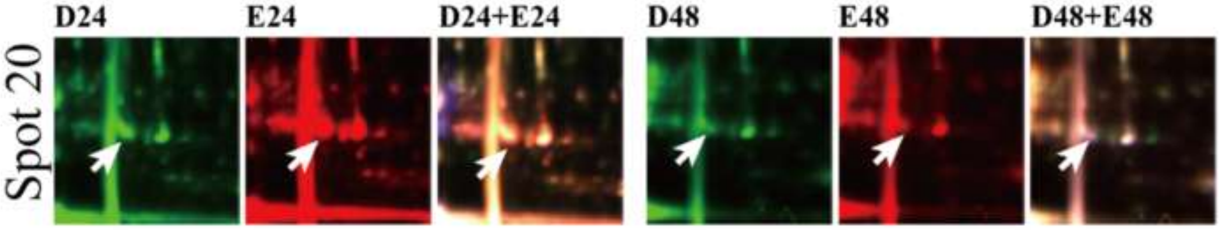

Location in rubber genome: scaffold1222_175215.mRNA1

Protein name: Rubber elongation factor protein/REF258

Predicted 3-D structure and phosphorylated amino acids:

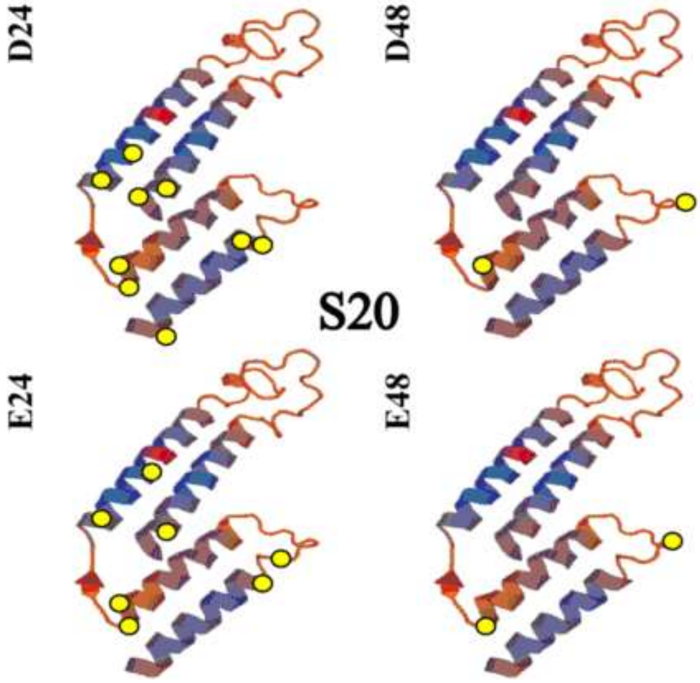

Detail information for phosphorylation of amino acid sites:

D24: MASLLGAASNVINAASNVVEEAVKGVENAQQEVANAVSNPSNIVKDVASAATDIVEEAAKGVENVQKEVANAVSN
E24: MASLLGAASNVINAASNVVEEAVKGVENAQQEVANAVSNPSNIVKDVASAATDIVEEAAKGVENVQKEVANAVSN
D48: MASLLGAASNVINAASNVVEEAVKGVENAQQEVANAVSNPSNIVKDVASAATDIVEEAAKGVENVQKEVANAVSN
E48: MASLLGAASNVINAASNVVEEAVKGVENAQQEVANAVSNPSNIVKDVASAATDIVEEAAKGVENVQKEVANAVSN

D24: SSNIVKDVASAATDIVEEAAKVVDNVQQGVVSAASNVVEEAAKGVGNIQEKVDDEEEDTLKYLDIVQAALVLALV
E24: SSNIVKDVASAATDIVEEAAKVVDNVQQGVVSAASNVVEEAAKGVGNIQEKVDDEEEDTLKYLDIVQAALVLALV
D48: SSNIVKDVASAATDIVEEAAKVVDNVQQGVVSAASNVVEEAAKGVGNIQEKVDDEEEDTLKYLDIVQAALVLALV
E48: SSNIVKDVASAATDIVEEAAKVVDNVQQGVVSAASNVVEEAAKGVGNIQEKVDDEEEDTLKYLDIVQAALVLALV

D24: SSSKLYLFVKDKSGPLKPGVDTAEVTIKSVVRPFYYRFHDVPNKVLKFADNQVDASVTLVLRYAPPVVKQVSTRA
E24: SSSKLYLFVKDKSGPLKPGVDTAEVTIKSVVRPFYYRFHDVPNKVLKFADNQVDASVTLVLRYAPPVVKQVSTRA
D48: SSSKLYLFVKDKSGPLKPGVDTAEVTIKSVVRPFYYRFHDVPNKVLKFADNQVDASVTLVLRYAPPVVKQVSTRA
E48: SSSKLYLFVKDKSGPLKPGVDTAEVTIKSVVRPFYYRFHDVPNKVLKFADNQVDASVTLVLRYAPPVVKQVSTRA

D24: YSVARNAPRAALALVSYLPLPTNRLCKLLSEDK
E24: YSVARNAPRAALALVSYLPLPTNRLCKLLSEDK
D48: YSVARNAPRAALALVSYLPLPTNRLCKLLSEDK
E48: YSVARNAPRAALALVSYLPLPTNRLCKLLSEDK

Spot No. 21

Changed pattern on DIGE gel:

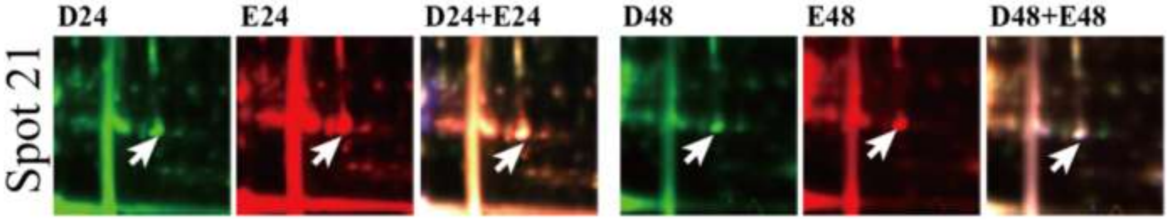

Location in rubber genome: scaffold1222_175215.mRNA1

Protein name: Rubber elongation factor protein/REF258

Predicted 3-D structure and phosphorylated amino acids:

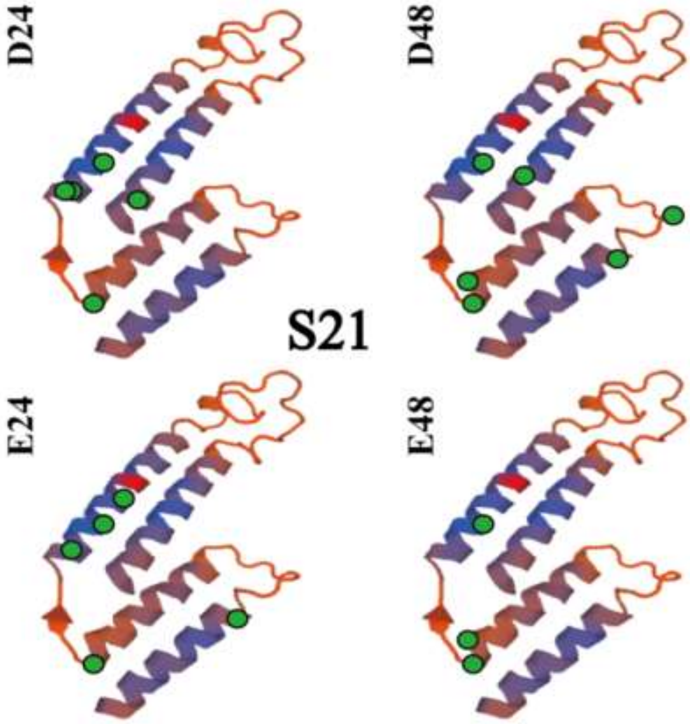

Detail information for phosphorylation of amino acid sites:

D24: MASLLGAASNVINAASNVVEEAVKGVENAQQEVANAVSNPSNIVKDVASAATDIVEEAAKGVENVQKEVANAVSN
E24: MASLLGAASNVINAASNVVEEAVKGVENAQQEVANAVSNPSNIVKDVASAATDIVEEAAKGVENVQKEVANAVSN
D48: MASLLGAASNVINAASNVVEEAVKGVENAQQEVANAVSNPSNIVKDVASAATDIVEEAAKGVENVQKEVANAVSN
E48: MASLLGAASNVINAASNVVEEAVKGVENAQQEVANAVSNPSNIVKDVASAATDIVEEAAKGVENVQKEVANAVSN
D24: SSNIVKDVASAATDIVEEAAKVVDNVQQGVVSAASNVVEEAAKGVGNIQEKVDDEEEDTLKYLDIVQAALVLALV
E24: SSNIVKDVASAATDIVEEAAKVVDNVQQGVVSAASNVVEEAAKGVGNIQEKVDDEEEDTLKYLDIVQAALVLALV
D48: SSNIVKDVASAATDIVEEAAKVVDNVQQGVVSAASNVVEEAAKGVGNIQEKVDDEEEDTLKYLDIVQAALVLALV
E48: SSNIVKDVASAATDIVEEAAKVVDNVQQGVVSAASNVVEEAAKGVGNIQEKVDDEEEDTLKYLDIVQAALVLALV
D24: SSSKLYLFVKDKSGPLKPGVDTAEVTIKSVVRPFYYRFHDVPNKVLKFADNQVDASVTLVLRYAPPVVKQVSTRA
E24: SSSKLYLFVKDKSGPLKPGVDTAEVTIKSVVRPFYYRFHDVPNKVLKFADNQVDASVTLVLRYAPPVVKQVSTRA
D48: SSSKLYLFVKDKSGPLKPGVDTAEVTIKSVVRPFYYRFHDVPNKVLKFADNQVDASVTLVLRYAPPVVKQVSTRA
E48: SSSKLYLFVKDKSGPLKPGVDTAEVTIKSVVRPFYYRFHDVPNKVLKFADNQVDASVTLVLRYAPPVVKQVSTRA
D24: YSVARNAPRAALALVSYLPLPTNRLCKLLSEDK
E24: YSVARNAPRAALALVSYLPLPTNRLCKLLSEDK
D48: YSVARNAPRAALALVSYLPLPTNRLCKLLSEDK
E48: YSVARNAPRAALALVSYLPLPTNRLCKLLSEDK

Spot No. 23

Changed pattern on DIGE gel:

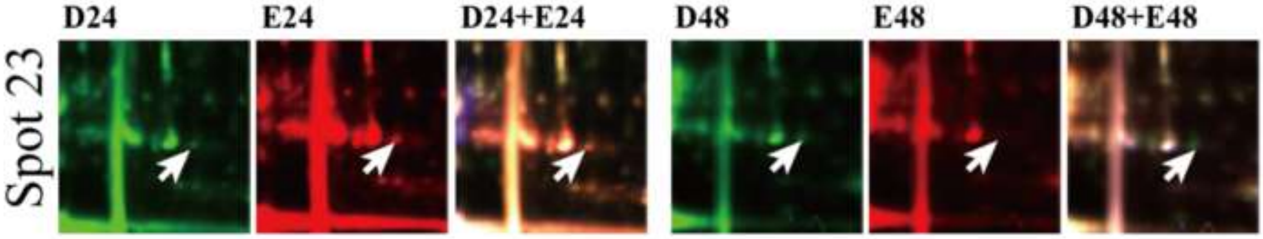

Location in rubber genome: scaffold1222_175215.mRNA1

Protein name: Rubber elongation factor protein/REF258

Predicted 3-D structure and phosphorylated amino acids:

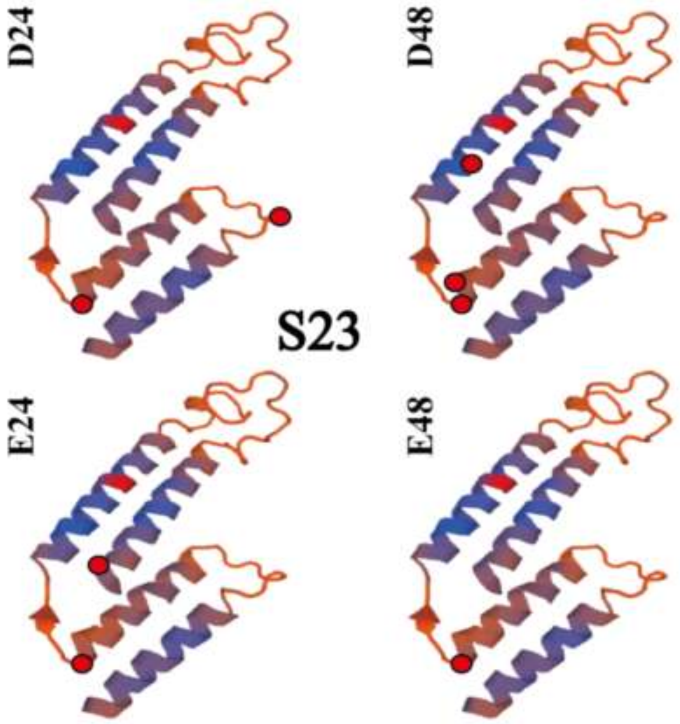

Detail information for phosphorylation of amino acid sites:

D24: MASLLGAA**S**NVINAASNVVEEAVKGVENAQQEVANAVSNPSNIVKDVASAA**T**DIVEEAAKGVENVQKEVANAVSN
E24: MASLLGAASNVINAASNVVEEAVKGVENAQQEVANAVSNPSNIVKDVASAATDIVEEAAKGVENVQKEVANAVSN
D48: MASLLGAASNVINAASNVVEEAVKGVENAQQEVANAVSNPSNIVKDVASAATDIVEEAAKGVENVQKEVANAVSN
E48: MASLLGAASNVINAASNVVEEAVKGVENAQQEVANAVSNPSNIVKDVASAATDIVEEAAKGVENVQKEVANAVSN
D24: SSNIVKDVASAATDIVEEAAKVVDNVQQGVVSAA**S**NVVEEAAKGVGNIQEKVDDEEEDTLKYLDIVQAALVLALV
E24: SSNIVKDVASAATDIVEEAAKVVDNVQQGVVSAA**S**NVVEEAAKGVGNIQEKVDDEEEDTLKYLDIVQAALVLALV
D48: SSNIVKDVASAATDIVEEAAKVVDNVQQGVV**S**AA**S**NVVEEAAKGVGNIQEKVDDEEEDTLKYLDIVQAALVLALV
E48: SSNIVKDVASAATDIVEEAAKVVDNVQQGVVSAA**S**NVVEEAAKGVGNIQEKVDDEEEDTLKYLDIVQAALVLALV
D24: SSSKLYLFVKDKSGPLKPGVDTAEVTIKSVVRPFYYRFHDVPNKVLKFADNQVDASVTLVLRYAPPVVKQVSTRA
E24: SSSKLYLFVKDKSGPLKPGVDTAEVTIKSVVRPFYYRFHDVPNKVLKFADNQVDASVTLVLRYAPPVVKQVSTRA
D48: SSSKLYLFVKDK**S**GPLKPGVDTAEVTIKSVVRPFYYRFHDVPNKVLKFADNQVDASVTLVLRYAPPVVKQVSTRA
E48: SSSKLYLFVKDKSGPLKPGVDTAEVTIKSVVRPFYYRFHDVPNKVLKFADNQVDASVTLVLRYAPPVVKQVSTRA
D24: YSVARNAPRAALALVSYLPLPTNRLCKLLSEDK
E24: YSVARNAPRAALALVSYLPLPTNRLC**K**LLSEDK
D48: YSVARNAPRAALALVSYLPLPTNRLCKLLSEDK
E48: YSVARNAPRAALALVSYLPLPTNRLCKLLSEDK

Spot No. 24

Changed pattern on DIGE gel:

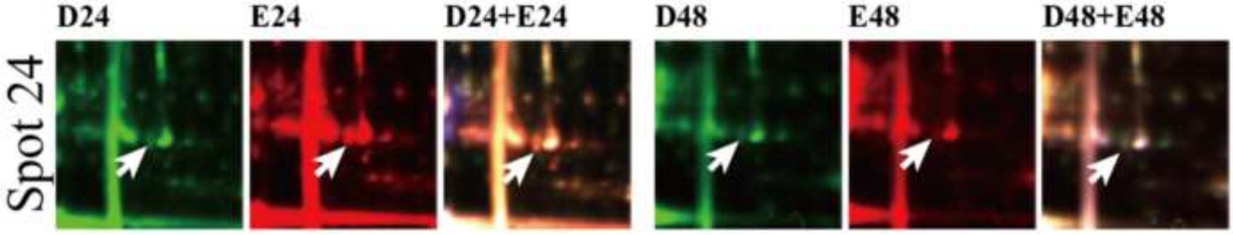

Location in rubber genome: scaffold1222_175215.mRNA1

Protein name: Rubber elongation factor protein/REF258

Predicted 3-D structure and phosphorylated amino acids:

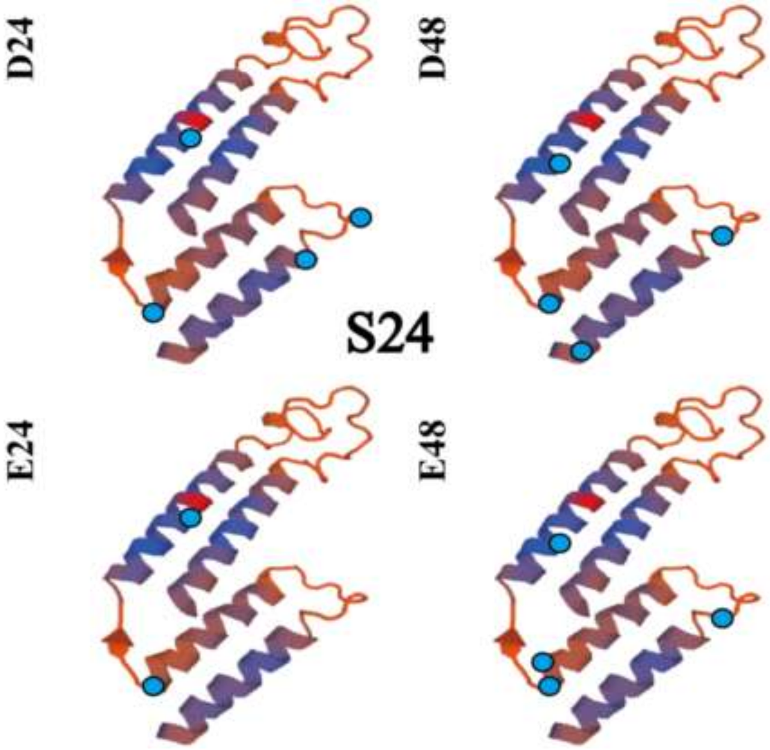

Detail information for phosphorylation of amino acid sites:

D24： MASLLGAASNVINAASNVVEEAVKGVENAQQEVANAVSNP**S**NIVKDVASAA**T**DIVEEAAKGVENVQKEVANAVSN
E24： MASLLGAASNVINAASNVVEEAVKGVENAQQEVANAVSNPSNIVKDVASAATDIVEEAAKGVENVQKEVANAVSN
D48： MASLLGAA**S**NVINAASNVVEEAVKGVENAQQEVANAVSNPSNIVKDVA**S**AATDIVEEAAKGVENVQKEVANAVSN
E48： MASLLGAASNVINAASNVVEEAVKGVENAQQEVANAVSNPSNIVKDVA**S**AATDIVEEAAKGVENVQKEVANAVSN
D24： SSNIVKDVASAATDIVEEAAKVVDNVQQGVVSAA**S**NVVEEAAKGVGNIQEKVDDEEEDTLKYLDIVQAALVLALV
E24： SSNIVKDVASAATDIVEEAAKVVDNVQQGVVSAA**S**NVVEEAAKGVGNIQEKVDDEEEDTLKYLDIVQAALVLALV
D48： SSNIVKDVASAATDIVEEAAKVVDNVQQGVVSAA**S**NVVEEAAKGVGNIQEKVDDEEEDTLKYLDIVQAALVLALV
E48： SSNIVKDVASAATDIVEEAAKVVDNVQQGVV**S**AA**S**NVVEEAAKGVGNIQEKVDDEEEDTLKYLDIVQAALVLALV
D24： SSSKLYLFVKDKSGPLKPGVD**T**AEVTIKSVVRPFYYRFHDVPNKVLKFADNQVDASVTLVLRYAPPVVKQVSTRA
E24： SSSKLYLFVKDKSGPLKPGVD**T**AEVTIKSVVRPFYYRFHDVPNKVLKFADNQVDASVTLVLRYAPPVVKQVSTRA
D48： SSSKLYLFVKDK**S**GPLKPGVDTAEVTIKSVVRPFYYRFHDVPNKVLKFADNQVDASVTLVLRYAPPVVKQVSTRA
E48： SSSKLYLFVKDK**S**GPLKPGVDTAEVTIKSVVRPFYYRFHDVPNKVLKFADNQVDASVTLVLRYAPPVVKQVSTRA
D24： YSVARNAPRAALALVSYLPLPTNRLCKLLSEDK
E24： YSVARNAPRAALALVSYLPLPTNRLCKLLSEDK
D48： YSVARNAPRAALALVSYLPLPTNRLCKLLSEDK
E48： YSVARNAPRAALALVSYLPLPTNRLCKLLSEDK

Spot No. 27

Changed pattern on DIGE gel:

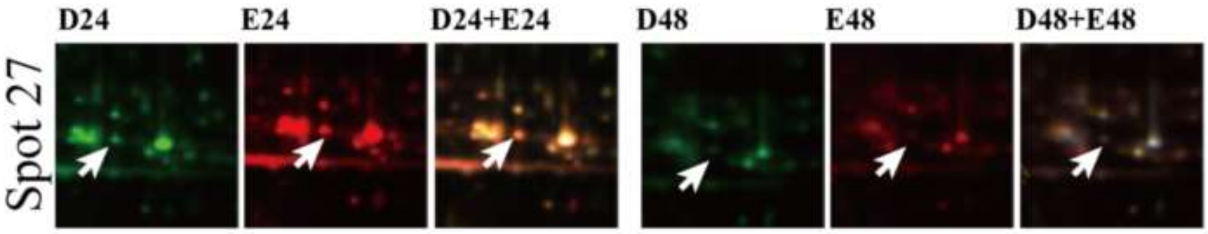

Location in rubber genome: scaffold1222_175215.mRNA1

Protein name: Rubber elongation factor protein/REF258

Predicted 3-D structure and phosphorylated amino acids:

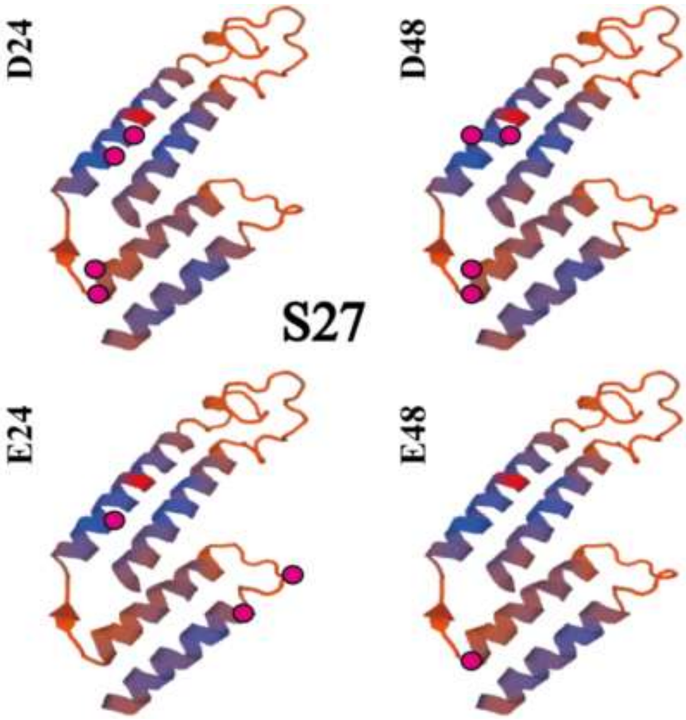

Detail information for phosphorylation of amino acid sites:

D24: MASLLGAASNVINAASNVVEEAVKGVENAQQEVANAVSNPSNIVKDVASAATDIVEEAAKGVENVQKEVANAVSN
E24: MASLLGAASNVINAASNVVEEAVKGVENAQQEVANAVSNPSNIVKDVASAATDIVEEAAKGVENVQKEVANAVSN
D48: MASLLGAASNVINAASNVVEEAVKGVENAQQEVANAVSNPSNIVKDVASAATDIVEEAAKGVENVQKEVANAVSN
E48: MASLLGAASNVINAASNVVEEAVKGVENAQQEVANAVSNPSNIVKDVASAATDIVEEAAKGVENVQKEVANAVSN
D24: SSNIVKDVASAATDIVEEAAKVVDNVQQGVVSAASNVVEEAAKGVGNIQEKVDDEEEDTLKYLDIVQAALVLALV
E24: SSNIVKDVASAATDIVEEAAKVVDNVQQGVVSAASNVVEEAAKGVGNIQEKVDDEEEDTLKYLDIVQAALVLALV
D48: SSNIVKDVASAATDIVEEAAKVVDNVQQGVVSAASNVVEEAAKGVGNIQEKVDDEEEDTLKYLDIVQAALVLALV
E48: SSNIVKDVASAATDIVEEAAKVVDNVQQGVVSAASNVVEEAAKGVGNIQEKVDDEEEDTLKYLDIVQAALVLALV
D24: SSSKLYLFVKDKSGPLKPGVDTAEVTIKSVVRPFYYRFHDVPNKVLKFADNQVDASVTLVLRYAPPVVKQVSTRA
E24: SSSKLYLFVKDKSGPLKPGVDTAEVTIKSVVRPFYYRFHDVPNKVLKFADNQVDASVTLVLRYAPPVVKQVSTRA
D48: SSSKLYLFVKDKSGPLKPGVDTAEVTIKSVVRPFYYRFHDVPNKVLKFADNQVDASVTLVLRYAPPVVKQVSTRA
E48: SSSKLYLFVKDKSGPLKPGVDTAEVTIKSVVRPFYYRFHDVPNKVLKFADNQVDASVTLVLRYAPPVVKQVSTRA
D24: YSVARNAPRAALALVSYLPLPTNRLCKLLSEDK
E24: YSVARNAPRAALALVSYLPLPTNRLCKLLSEDK
D48: YSVARNAPRAALALVSYLPLPTNRLCKLLSEDK
E48: YSVARNAPRAALALVSYLPLPTNRLCKLLSEDK

Protein name: SRPP204

Location of the three spots identified as SRPP204 by MS from 2-D DIGE gels:

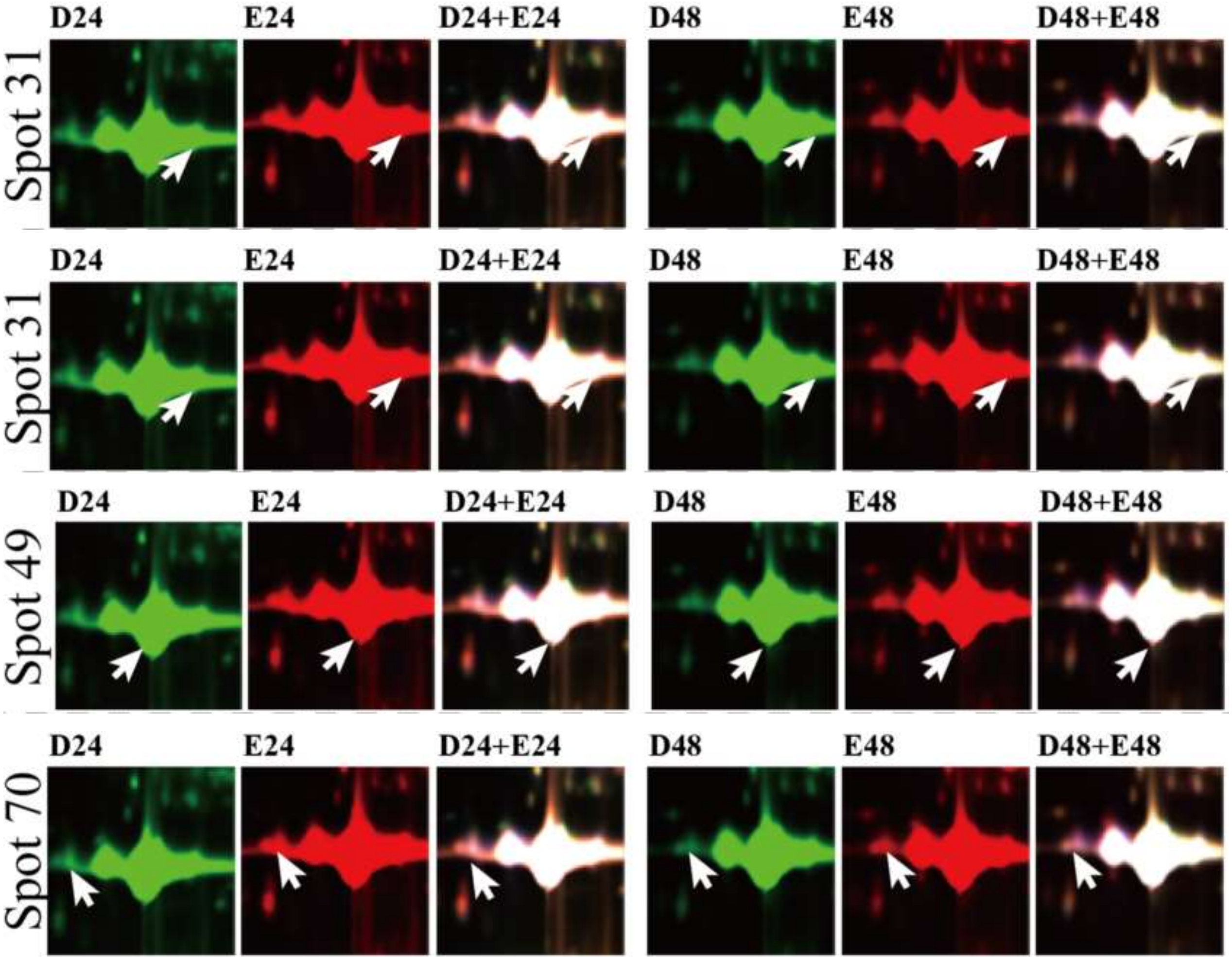

Detail information for phosphorylation of amino ac id sites in SRPP204：

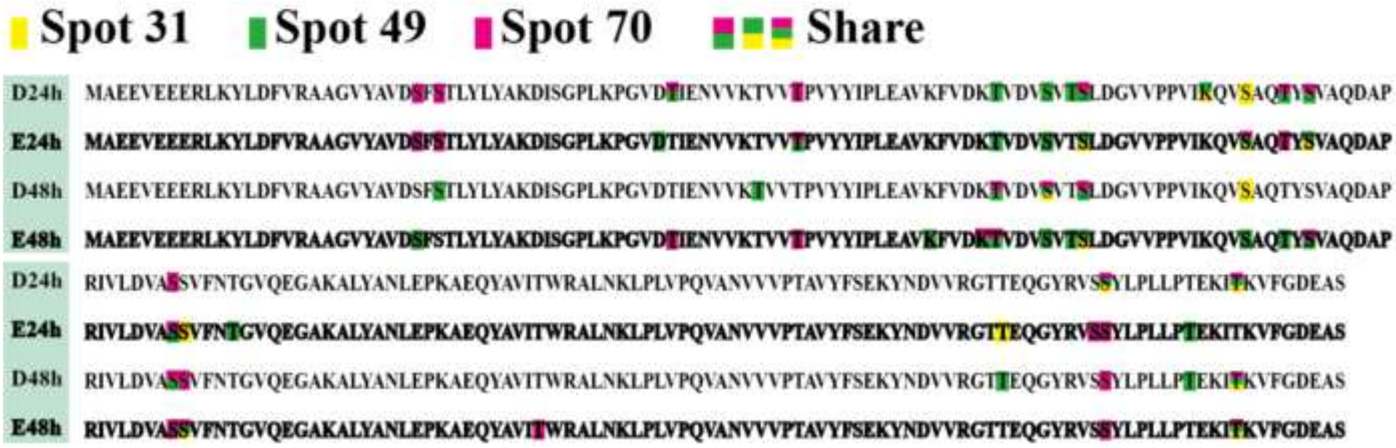

Predicted 3-D structure and phosphorylated amino acids in different SRPP204 isoforms:

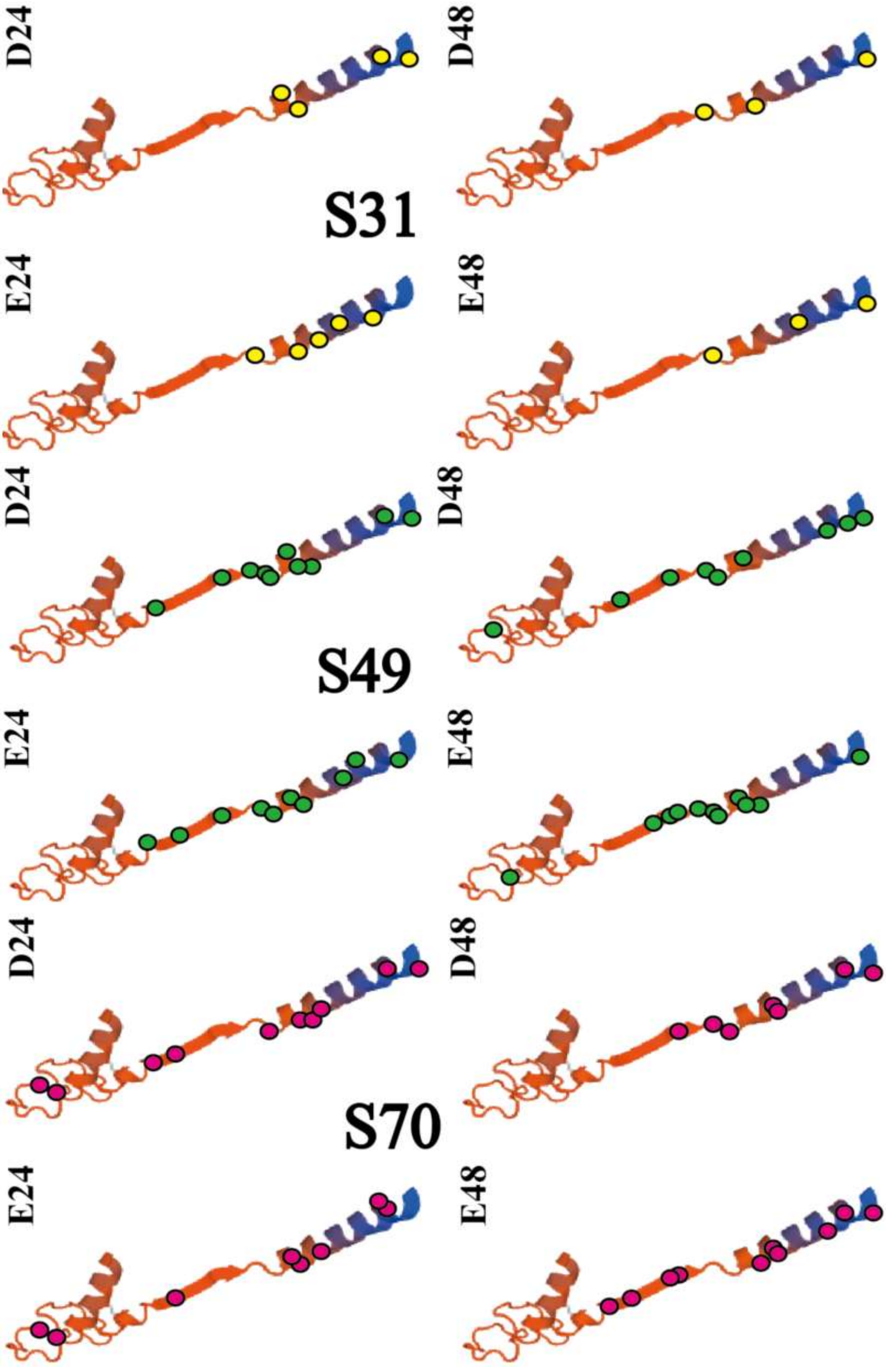

Spot No. 31

Changed pattern on DIGE gel:

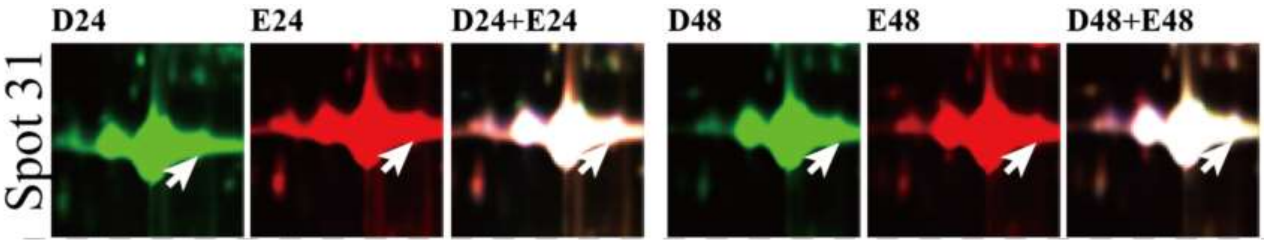

Location in rubber genome: scaffold1222_60641.mRNA1

Protein name: Small rubber particle protein/SRPP204

Predicted 3-D structure and phosphorylated amino acids:

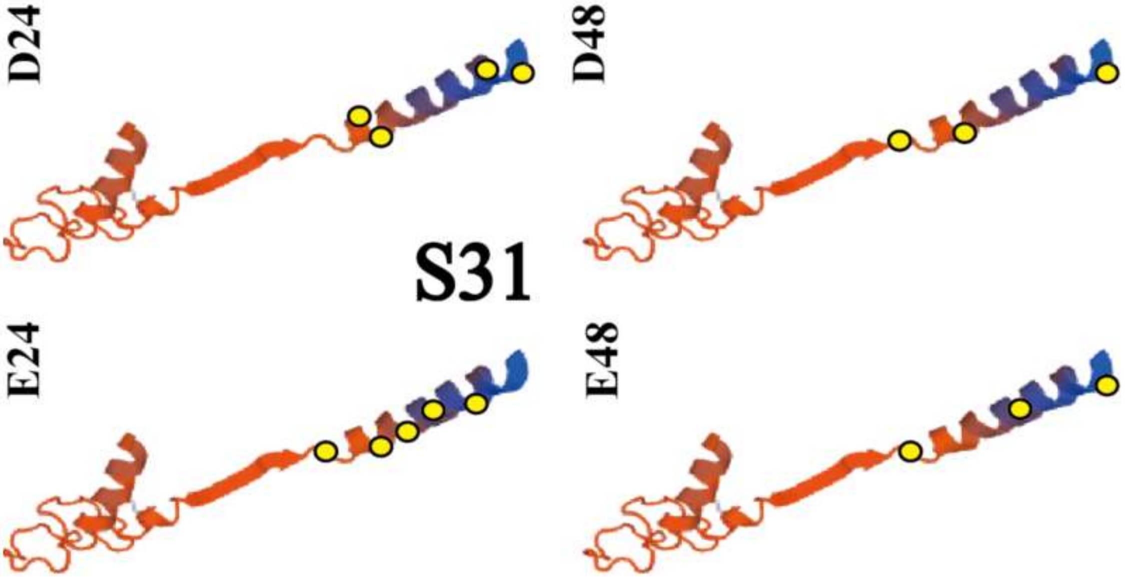

Detail information for phosphorylation of amino acid sites:

D24: MAEEVEEERLKYLDFVRAAGVYAVDSFSTLYLYAKDISGPLKPGVDTIENVVKTVVTPVYYIPLEAVKFVDKTVD
E24: MAEEVEEERLKYLDFVRAAGVYAVDSFSTLYLYAKDISGPLKPGVDTIENVVKTVVTPVYYIPLEAVKFVDKTVD
D48: MAEEVEEERLKYLDFVRAAGVYAVDSFSTLYLYAKDISGPLKPGVDTIENVVKTVVTPVYYIPLEAVKFVDKTVD
E48: MAEEVEEERLKYLDFVRAAGVYAVDSFSTLYLYAKDISGPLKPGVDTIENVVKTVVTPVYYIPLEAVKFVDKTVD

D24: VSVTSLDGVVPPVIKQVSAQTYSVAQDAPRIVLDVASSVFNTGVQEGAKALYANLEPKAEQYAVITWRALNKLPL
E24: VSVTSLDGVVPPVIKQVSAQTYSVAQDAPRIVLDVASSVFNTGVQEGAKALYANLEPKAEQYAVITWRALNKLPL
D48: VSVTSLDGVVPPVIKQVSAQTYSVAQDAPRIVLDVASSVFNTGVQEGAKALYANLEPKAEQYAVITWRALNKLPL
E48: VSVTSLDGVVPPVIKQVSAQTYSVAQDAPRIVLDVASSVFNTGVQEGAKALYANLEPKAEQYAVITWRALNKLPL

D24: VPQVANVVVPTAVYFSEKYNDVVRGTTEQGYRVSSYLPLLPTEKITKVFGDEAS
E24: VPQVANVVVPTAVYFSEKYNDVVRGTTEQGYRVSSYLPLLPTEKITKVFGDEAS
D48: VPQVANVVVPTAVYFSEKYNDVVRGTTEQGYRVSSYLPLLPTEKITKVFGDEAS
E48: VPQVANVVVPTAVYFSEKYNDVVRGTTEQGYRVSSYLPLLPTEKITKVFGDEAS

Spot No. 49

Changed pattern on DIGE gel:

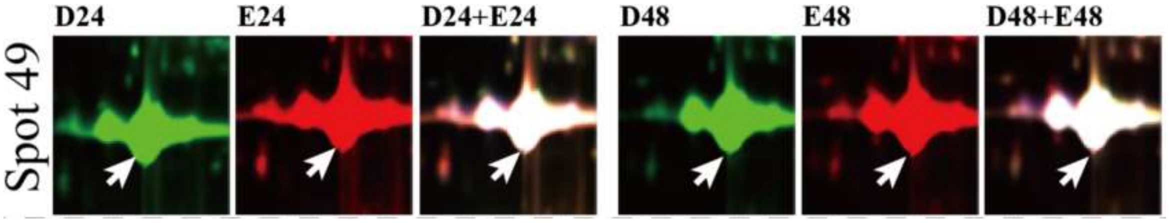

Location in rubber genome: scaffold1222_60641.mRNA1

Protein name: Small rubber particle protein/SRPP204

Predicted 3-D structure and phosphorylated amino acids:

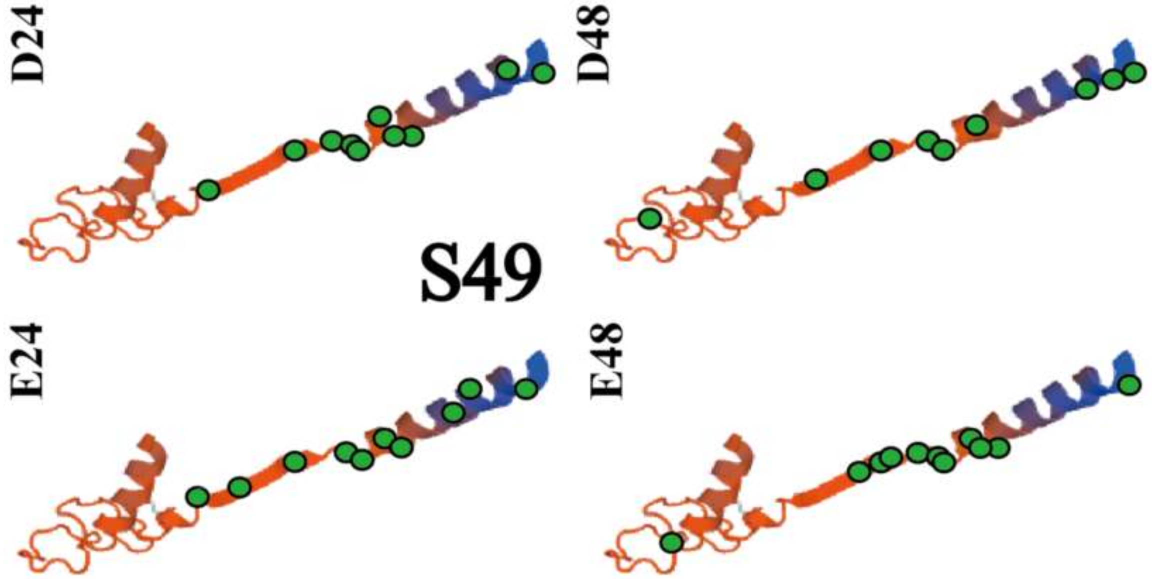

Detail information for phosphorylation of amino acid sites:

D24: MAEEVEEERLKYLDFVRAAGVYAVDSFSTLYLYAKDISGPLKPGVDTIENVVKTVVTPVYYIPLEAVKFVDKTVD
E24: MAEEVEEERLKYLDFVRAAGVYAVDSFSTLYLYAKDISGPLKPGVDTIENVVKTVVTPVYYIPLEAVKFVDKTVD
D48: MAEEVEEERLKYLDFVRAAGVYAVDSFSTLYLYAKDISGPLKPGVDTIENVVKTVVTPVYYIPLEAVKFVDKTVD
E48: MAEEVEEERLKYLDFVRAAGVYAVDSFSTLYLYAKDISGPLKPGVDTIENVVKTVVTPVYYIPLEAVKFVDKTVD

D24: VSVTSLDGVVPPVIKQVSAQTYSVAQDAPRIVLDVASSVFNTGVQEGAKALYANLEPKAEQYAVITWRALNKLPL
E24: VSVTSLDGVVPPVIKQVSAQTYSVAQDAPRIVLDVASSVFNTGVQEGAKALYANLEPKAEQYAVITWRALNKLPL
D48: VSVTSLDGVVPPVIKQVSAQTYSVAQDAPRIVLDVASSVFNTGVQEGAKALYANLEPKAEQYAVITWRALNKLPL
E48: VSVTSLDGVVPPVIKQVSAQTYSVAQDAPRIVLDVASSVFNTGVQEGAKALYANLEPKAEQYAVITWRALNKLPL

D24: VPQVANVVVPTAVYFSEKYNDVVRGTTEQGYRVSSYLPLLPTEKITKVFGDEAS
E24: VPQVANVVVPTAVYFSEKYNDVVRGTTEQGYRVSSYLPLLPTEKITKVFGDEAS
D48: VPQVANVVVPTAVYFSEKYNDVVRGTTEQGYRVSSYLPLLPTEKITKVFGDEAS
E48: VPQVANVVVPTAVYFSEKYNDVVRGTTEQGYRVSSYLPLLPTEKITKVFGDEAS

Spot No. 70

Changed pattern on DIGE gel:

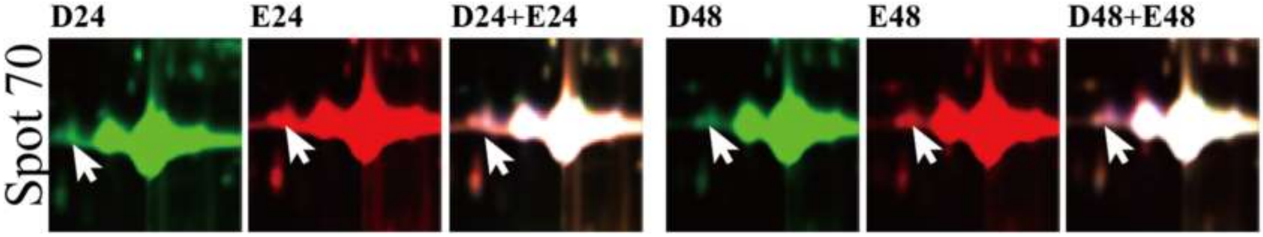

Location in rubber genome: scaffold1222_60641.mRNA1

Protein name: Small rubber particle protein/SRPP204

Predicted 3-D structure and phosphorylated amino acids:

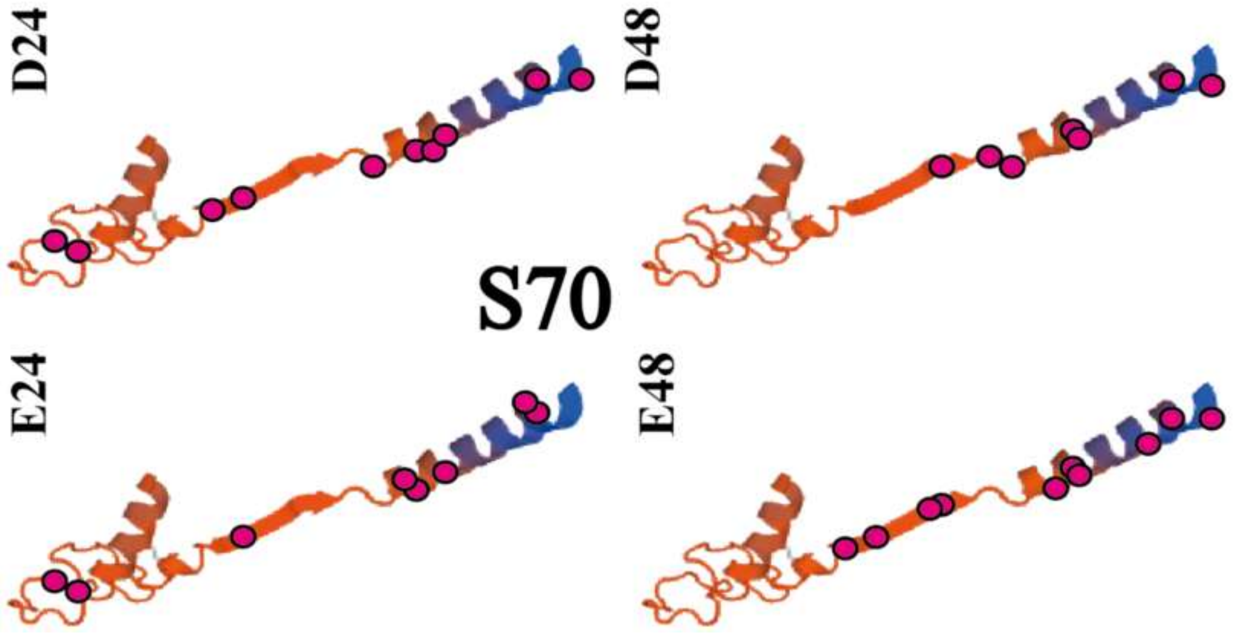

Detail information for phosphorylation of amino acid sites:

D24：MAEEVEEERLKYLDFVRAAGVYYAVDSFSTLYLYAKDISGPLKPGVDTIENVVKTVVTPVYYIPLEAVKFVDKTVD
E24：MAEEVEEERLKYLDFVRAAGVYYAVDSFSTLYLYAKDISGPLKPGVDTIENVVKTVVTPVYYIPLEAVKFVDKTVD
D48：MAEEVEEERLKYLDFVRAAGVYYAVDSFSTLYLYAKDISGPLKPGVDTIENVVKTVVTPVYYIPLEAVKFVDKTVD
E48：MAEEVEEERLKYLDFVRAAGVYYAVDSFSTLYLYAKDISGPLKPGVDTIENVVKTVVTPVYYIPLEAVKFVDKTVD

D24：VSVTSLDGVVPPVIKQVSAQTYSVAQDAPRIVLDVASSVFNTGVQEGAKALYANLEPKAEQYAVITWRALNKLPL
E24：VSVTSLDGVVPPVIKQVSAQTYSVAQDAPRIVLDVASSVFNTGVQEGAKALYANLEPKAEQYAVITWRALNKLPL
D48：VSVTSLDGVVPPVIKQVSAQTYSVAQDAPRIVLDVASSVFNTGVQEGAKALYANLEPKAEQYAVITWRALNKLPL
E48：VSVTSLDGVVPPVIKQVSAQTYSVAQDAPRIVLDVASSVFNTGVQEGAKALYANLEPKAEQYAVITWRALNKLPL

D24：VPQVANVVVPTAVYFSEKYNDVVRGTTEQGYRVSSYLPLLPTEKITKVFGDEAS
E24：VPQVANVVVPTAVYFSEKYNDVVRGTTEQGYRVSSYLPLLPTEKITKVFGDEAS
D48：VPQVANVVVPTAVYFSEKYNDVVRGTTEQGYRVSSYLPLLPTEKITKVFGDEAS
E48：VPQVANVVVPTAVYFSEKYNDVVRGTTEQGYRVSSYLPLLPTEKITKVFGDEAS
