# Supplementary figures and images for: Proteomic Landscape Has Revealed Small Rubber Particles Are Crucial Rubber Biosynthetic Machines for Ethylene-Stimulation in Natural Rubber Production
Source: Int J Mol Sci. 2019 Oct 14;20(20):5082. doi: 10.3390/ijms20205082 (PMC6829444; doi:10.3390/ijms20205082)

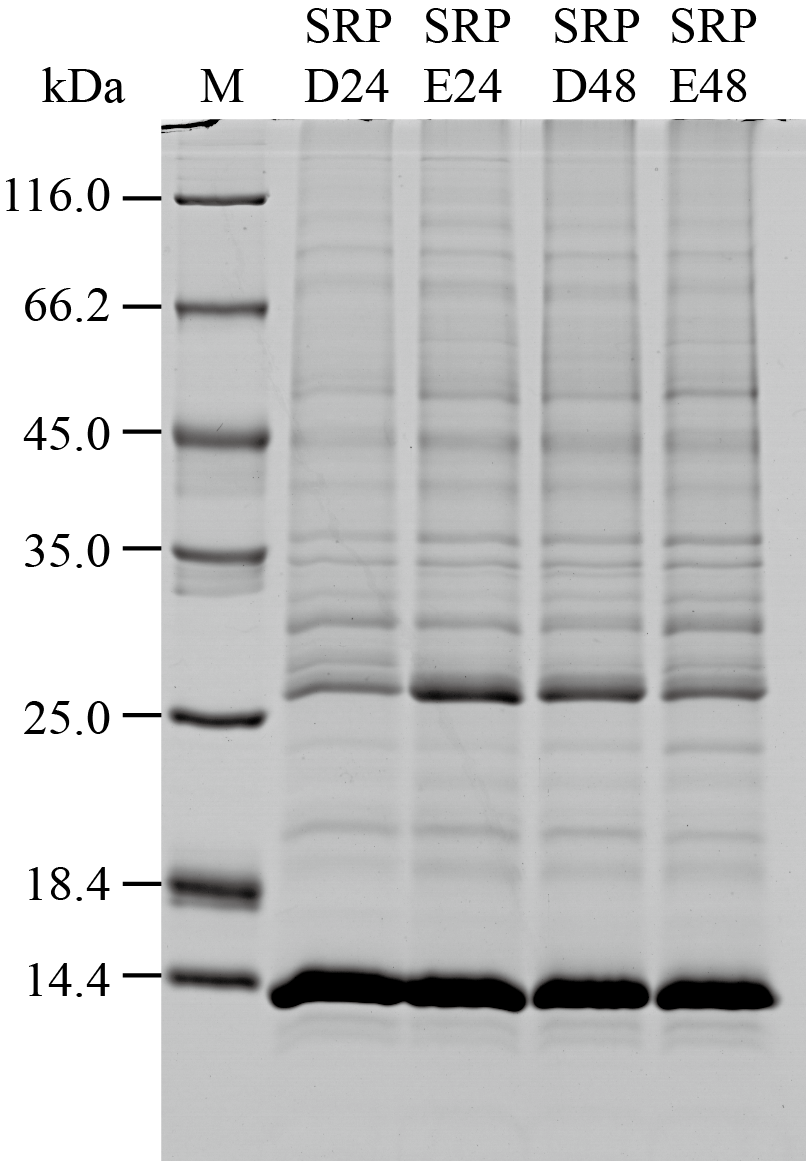

Supplement: Supplementary file 1 [file ijms-20-05082-s001.zip › ijms-597831-SI/ijms-597831-supplementary new 20191010/Figure S1 OK SDS-PAGE analysis of proteins in different small rubber particles.tif]
